# Identification of candidate genes for *LepR1* resistance against *Leptosphaeria maculans* in *Brassica napus*

**DOI:** 10.3389/fpls.2023.1051994

**Published:** 2023-02-14

**Authors:** Aldrin Y. Cantila, William J.W. Thomas, Nur Shuhadah Mohd Saad, Anita A. Severn-Ellis, Robyn Anderson, Philipp E. Bayer, David Edwards, Angela P. Van de Wouw, Jacqueline Batley

**Affiliations:** ^1^ School of Biological Sciences, The University of Western Australia, Crawley, WA, Australia; ^2^ School of BioSciences, University of Melbourne, Parkville, VIC, Australia

**Keywords:** association mapping, blackleg resistance, canola, gene sequencing, *LepR1* resistant-specific marker

## Abstract

Utilising resistance (*R*) genes, such as *LepR1*, against *Leptosphaeria maculans*, the causal agent of blackleg in canola (*Brassica napus*), could help manage the disease in the field and increase crop yield. Here we present a genome wide association study (GWAS) in *B. napus* to identify *LepR1* candidate genes. Disease phenotyping of 104 *B. napus* genotypes revealed 30 resistant and 74 susceptible lines. Whole genome re-sequencing of these cultivars yielded over 3 million high quality single nucleotide polymorphisms (SNPs). GWAS in mixed linear model (MLM) revealed a total of 2,166 significant SNPs associated with *LepR1* resistance. Of these SNPs, 2108 (97%) were found on chromosome A02 of *B. napus* cv. Darmor bzh v9 with a delineated *LepR1*_mlm1 QTL at 15.11-26.08 Mb. In *LepR1*_mlm1, there are 30 resistance gene analogs (RGAs) (13 nucleotide-binding site-leucine rich repeats (NLRs), 12 receptor-like kinases (RLKs), and 5 transmembrane-coiled-coil (TM-CCs)). Sequence analysis of alleles in resistant and susceptible lines was undertaken to identify candidate genes. This research provides insights into blackleg resistance in *B. napus* and assists identification of the functional *LepR1* blackleg resistance gene.

## Introduction

*Brassica napus* L. (canola, oilseed rape) is one of the most important crops for edible oil, food and industrial purposes (Şensöz et al., 2000; Abbadi and Leckband, 2011). The breeding of canola cultivars has focused on oil quality, seed yield and resistance to pests and diseases (Potter et al., 2016; Salisbury et al., 2016). One of the major diseases affecting canola production is blackleg, caused by the fungal pathogen *Leptosphaeria maculans.* Blackleg can reduce seed yield by at least 50% (Gugel and Petrie, 1992; West et al., 2001; Van de Wouw et al., 2010; Potter et al., 2016; Salisbury et al., 2016) and also affect oil quality (Fikere et al., 2020a). Blackleg disease is difficult to manage, even with fungicide application and recently there has been identification of fungicide tolerant *L. maculans* isolates (Kutcher et al., 2010; Van de Wouw et al., 2017; Van de Wouw et al., 2021). Thus, utilising resistant cultivars is recognised as an effective and environmentally safe approach in managing *L. maculans* and minimising yield loss.

There are two types of resistance against *L. maculans* in *B. napus;* qualitative and quantitative resistance (Delourme et al., 2006). Qualitative resistance involves an interaction between a resistance (*R)* gene and an avirulence (*Avr*) gene (Flor, 1971). These *R* genes, also termed major genes, are highly heritable and manifest effectively from the cotyledon stage through to the adult stage (Raman et al., 2012; Raman et al., 2012). On the other hand, quantitative resistance, considered to be governed by multiple minor genes, provides partial resistance to individual isolates and is poorly understood both phenotypically and genotypically (Huang et al., 2009). Both types of blackleg resistance are important in canola and many commercial cultivars have both qualitative and quantitative resistance.

To determine the position of an *R* gene in the genome, genetic mapping of genotypic variants is frequently undertaken. Two of the most common approaches for genetic mapping are association mapping, or genome wide association studies (GWAS), and biparental linkage mapping. GWAS is an alternative approach to traditional mapping of biparental populations and has been widely used in *B. napus* (Raman et al., 2014; Zhang et al., 2015; Gacek et al., 2016; Raman et al., 2016; Wu et al., 2016; Fu et al., 2020; Hu et al., 2022). The detection of quantitative trait loci (QTL) through GWAS depends on the level of linkage disequilibrium (LD) between functional loci and markers, which most GWAS models can statistically and easily determine. Generalized linear models (GLM) and mixed linear models (MLM) are the most common GWAS models used in plant studies, as these models are flexible and can detect multiple genotypic variants. Of the two models, MLM simultaneously incorporates kinship and principal component analyses (PCA) to address cryptic relatedness in tested materials (Yu et al., 2006).

Currently 16 qualitative blackleg *R* genes have been mapped in *Brassica* species, however only *LepR3, Rlm2*, *Rlm4*, *Rlm7* and *Rlm9* have been cloned to date (Larkan et al., 2013; Larkan et al., 2015; Larkan et al., 2020; Haddadi et al., 2022). *LepR3* and *Rlm2* are alleles of the same gene, a resistance gene analog (RGA) encoding a receptor-like protein (RLP), while *Rlm4*, *Rlm7* and *Rlm9* are also allelic variants of an RGA encoding wall-associated kinases (WAKs) (Larkan et al., 2020; Haddadi et al., 2022). Other blackleg RGAs have also been cloned in *Arabidopsis thaliana*; *RLM1a*, *RLM1b* and *RLM3* which encode nucleotide-binding site (NBS)-leucine rich repeats (LRR) (Staal et al., 2006; Staal et al., 2008). One of the blackleg *R* genes yet to be cloned is *LepR1*, which was originally introgressed from *Brassica rapa* L. subsp. *sylvestris* into *B. napus* and has been mapped on chromosome A02 of *B. napus* (Yu et al., 2005; Larkan et al., 2016; Dolatabadian et al., 2019; Fikere et al., 2020b). This study used whole genome re-sequencing (WGRS) data from 104 Australian *B. napus* genotypes, screened with blackleg isolates containing *AvrLep1* to identify a precise location of the QTL harbouring *LepR1* and to identify candidate genes within this region. Lastly, sequence analysis was used to identify candidate *LepR1* genes in *B. napus*.

## Materials and methods

### Blackleg isolates and disease phenotyping

A set of 14 well-characterised differential *L. maculans* isolates were used to genotype *B. napus* genotypes for the presence or absence of resistance gene *LepR1*. All isolates and their genotypes (**Table S1**) were maintained on 10% V8 media. Pycnidiospores (10^-5^) of each *L. maculans* isolate were dropped onto cotyledons of wounded ten-day-old seedlings grown in the glasshouse at 22°C. Disease symptoms were scored at 14 days post-inoculation on a 0-9 scale, as previously described (Marcroft et al., 2012). For each isolate x cultivar interaction, eight replicate plants were inoculated, each with four wounds per plant. Average pathogenicity scores of <5.0 were considered avirulent, whilst average scores of >5.0 were considered virulent.

### DNA extraction and whole genome re-sequencing

A total of 104 Australian *B. napus* genotypes (89 commercial cultivars and 15 breeding lines) were used in this study (**Table S2**). The genotypes were grown in a glasshouse at 18°C during the day and 13°C at night in a 12-hour photoperiod cycle for two weeks. Approximately 100 mg of young, healthy leaf material was collected, snap-frozen in liquid nitrogen and finely ground using a Geno-Grinder 2010 (SPEX SamplePrep, Mettuchen, Germany). DNA was extracted using the DNeasy Plant Kit (Qiagen^©^, Hilden, Germany) according to the manufacturer’s instructions. Total DNA was quantified using a Qubit 3.0 Fluorometer and the Qubit dsDNA BR Assay kit (Invitrogen, Carlsbad, United States), while DNA quality was assessed using the LabChip GX Touch 24 (PerkinElmer, Waltham, United States). WGRS of the cultivars was carried out using an Illumina NovaSeq™ 6000 Sequencing System by GeneWiz (Brookes Life Sciences, Guangzhou, China), which generated approximately ~6 Gb of 150 bp paired-end sequencing data per cultivar.

### SNP calling and filtering

From the WGRS data, raw FASTQ files were trimmed of adapter sequences and low quality bases using Trimmomatic v0.39 (Bolger et al., 2014) with a maximum mismatch score of 2, a palindrome clip score threshold of 30 and a clip score threshold of 10. Low quality bases with a Phred+ 33 score of < 3 were trimmed from both the start and end of each read. Sliding window trimming was carried out using a 4-base wide window to remove bases with an average quality per base of <15. All the reads with fewer than 36 bases, as well as unpaired reads paired after trimming, were discarded. To verify adapter and read quality trimming, untrimmed and trimmed reads were analysed using FastQC (Andrews, 2010) followed by MultiQC (Ewels et al., 2016), which were compared afterwards.

Trimmed paired reads were then aligned to *B. napus* genome cv. Darmor bzh v9 (Bayer et al., 2020), using Bowtie2 v2.4.1 (Langmead and Salzberg, 2012) with settings end-to-end, sensitive,-I 0 and -X 1000. Alignments were subsequently converted to bam format and sorted using samtools v.1.10 (Li et al., 2009; https://github.com/samtools/bcftools). Duplicate reads were removed using samtools markdup. SNPs for each individual were called using bcftools v.1.10 (Li et al., 2009) functions mpileup (-q 10 and –Q 20). The vcf files were indexed using tabix (Li, 2011) while the SNPs were merged per chromosome using bcftools.

Merged variants were further filtered using VCFtools v0.1.16 (Danecek et al., 2011). Indels and multiallelic SNPs were omitted (–remove-indels –max-alleles 2 –min-alleles 2). Individuals with > 0.9 missing genotypes were removed before the filtering of SNPs. Genotypes with a depth of < 5 (–minDP 5) were set to missing to minimise the rate of heterozygous alleles incorrectly called as homozygous alleles due to insufficient read depth. SNPs displaying a minor allele frequency (MAF) of < 0.05 (–MAF 0.05) or when genotypes were not present in > 80% of all individuals (–max-missing 0.8) were also discarded. The filtered SNP data was then used as genotype data for GWAS.

### Association analysis

The GWAS results, along with quantile-quantile (QQ) plot in mixed linear model (MLM) with PCA (3 principal components) + Kinship matrix for family relatedness estimates (K) (Yu et al., 2006), were computed using the GAPIT3 R package (Wang and Zhang, 2021). An additional GWAS model; Fixed and random model Circulating Probability Unification (FarmCPU) (Liu et al., 2016), computed through GAPIT3 R package was used to determine consistent significant SNPs across two models. From the GWAS result, CMplot R package (Yin et al., 2021) was used to visualise circular Manhattan and SNP density plots. MAF and adjusted P-value following a false discovery rate (FDR)-controlling procedure (Benjamini and Hochberg, 1995), additional criteria to select SNPs associated to the trait, were also identified using GAPIT3. GAPIT was also used to show the heterozygosity information and LD, represented by R^2^ which measures two biallelic markers (Hill and Robertson, 1968). The phenotypic variation explained by a SNP marker (PVE) was taken using the following formula: PVE = [(R2 with SNP model value- R2 without SNP model value) x 100] (Sun et al., 2010). Figure plotting of the SNP statistics was also done in GAPIT3 while the other SNP statistics were computed in TASSEL 5.0 (Bradbury et al., 2007).

### Current and previous QTL dissection

Putative QTL were derived from the significant SNPs identified through MLM GWAS. A QTL was defined either as a 100 kb upstream and downstream region of each significant SNP (Anderson et al., 2020) or a region with two significant SNPs located ≤ 15 Mb from each other on the same chromosome (Martínez-Montes et al., 2018), also integrating a 100 kb region upstream and downstream from the SNP. For QTL comparison, the previous QTL (chromosome A02) based on *LepR1* introgressed lines and structural variants, derived from the results of *L. maculans* containing *AvrLep1* screened at the cotyledon stage (Larkan et al., 2016; Dolatabadian et al., 2019), and based on adult plant survival rate and average internal infection of the stem at maturity stage (Fikere et al., 2020b) (considered as the QTL outlier) were physically mapped to *B. napus* cv Darmor bzh v9 using MapChart 2.32 for comparison (Voorrips, 2002). Then, the number of genes and RGAs in the derived QTL intervals were identified for comparison.

The RGAugury pipeline (Li et al., 2016) was used to predict RGAs, along with the classes, for example NLR, RLK, RLP, and TM-CC (Transmembrane (TM)- Coiled-Coil (CC)) and subclasses including NBS, CNL (CC-NBS-LRR), CN (CC-NBS), NL (NBS-LRR), TNL (Toll/Interleukin-1 Receptor (TIR)-NBS-LRR), TN (TIR-NBS), TX (TIR with unknown domains), in Darmor bzh v9 (Bayer et al., 2020), then the RGAs within each QTL region were classified. The SNPs of the *LepR1* candidate genes (current QTL in A02) were visualised in Geneious R10 (Kearse et al., 2012) and identified as either within a non-coding or coding (CDS) region, non-synonymous or synonymous if it was within the coding region, and non-sense or missense variant if it was a non-synonymous SNP.

### Molecular analysis

The *LepR1* candidates which had at least 2 SNPs in CDS regions were chosen as the most likely candidates. We also included two flanking RGAs (for comparison) nearest to the *LepR1* QTL identified in this study. The marker development was done in two stages. First, markers flanking each candidate were designed using the Primer3 function in Geneious R10 (Kearse et al., 2012) and the markers were tested for allele detection of the resistant and susceptible materials. Then, the amplicons showing segregation between resistant and susceptible lines were purified for sequencing. MiSeq sequencing was done using an Illumina MiSeq platform at the Australian Genome Research Facility (AGRF), Perth, Australia. MiSeq reads were quality-trimmed using Trimmomatic (Bolger et al., 2014) and assembled using Spades version 3.6.0 (Bankevich et al., 2012). The markers showing sequence variation between resistant and susceptible materials were used to design a resistant-specific marker.

## Results

### Phenotyping

Out of 104 *B. napus* cultivars, 30 showed a resistance response to *L. maculans* isolates containing *AvrLep1*, indicating the presence of *LepR1*, while 74 showed a susceptible response, indicating they do not harbour *LepR1* (**Table S2**).

### WGRS SNP analysis

The WGRS data of 104 *B. napus* genotypes produced a total of 3,235,008 high quality SNPs that were used in the GWAS (**Figure S1**). The average number of heterozygous SNPs was 427,970 SNPs per *B. napus* genotype (7.77% of the 3,235,008 high quality SNPs) (**Figure S1**, **Table S3**). LD also showed a slower decay rate (**Figure S2**) over the previous *B. napus* LD findings obtained using less dense markers (845 RFLP, 89 and 451 SSR, 8,502 and 251,575 GBS SNP markers) (Bus et al., 2011; Xiao et al., 2012; Horvath et al., 2020; Rahman et al., 2022).

### SNPs, QTL and RGAs associated with *LepR1* resistance

**Figure 1:**
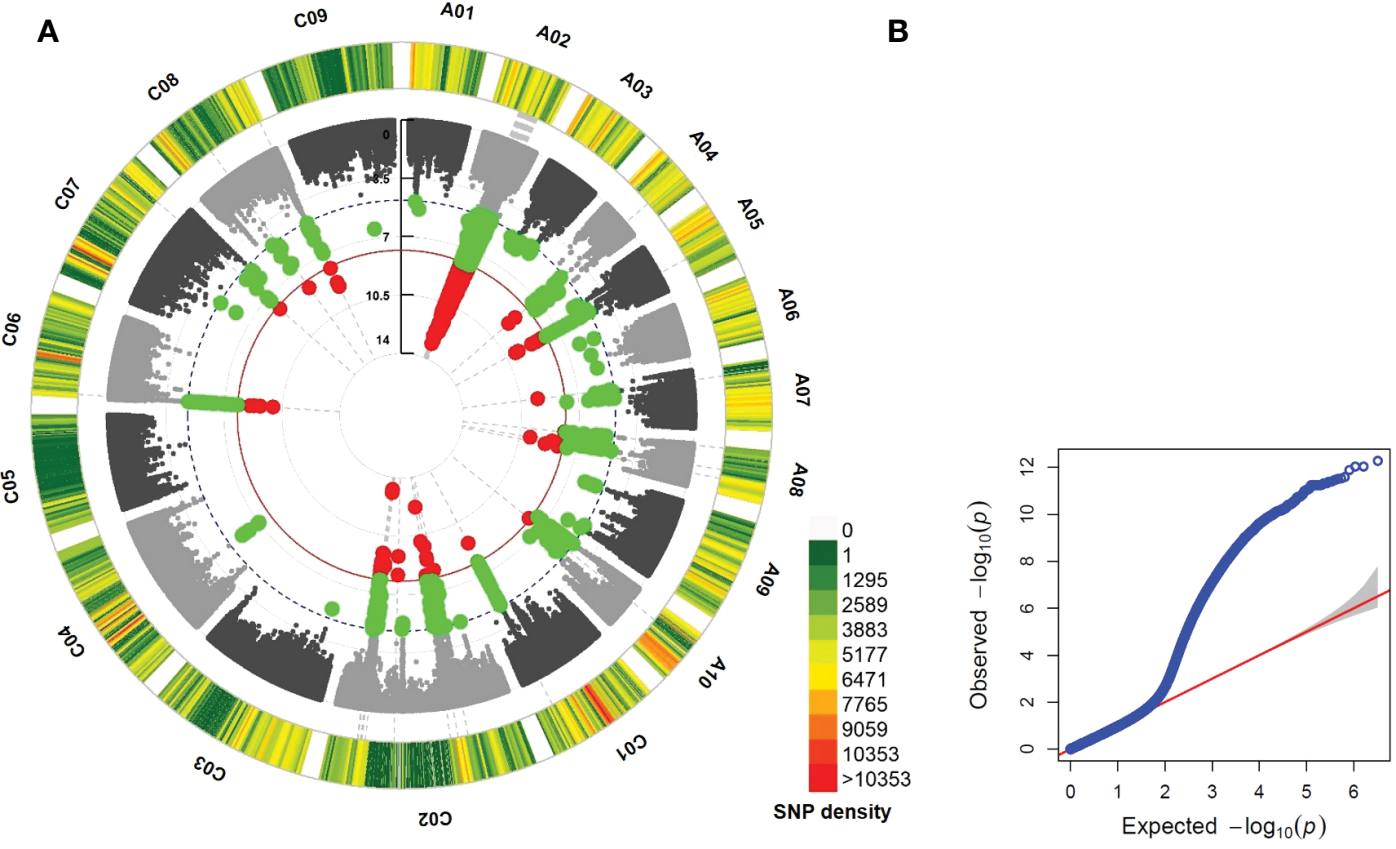
Circular Manhattan **(A)** and quantile-quantile (QQ) plots **(B)** showing the significant single nucleotide polymorphisms (SNPs) above the significance threshold line (red dot), 1.58 E-08, in mixed linear model (MLM) of genome wide association study in *Brassica napus* cv. Darmor bzh v9. Green dot refers to SNPs below the cut-off for significance having at least E-05.

**Figure 2:**
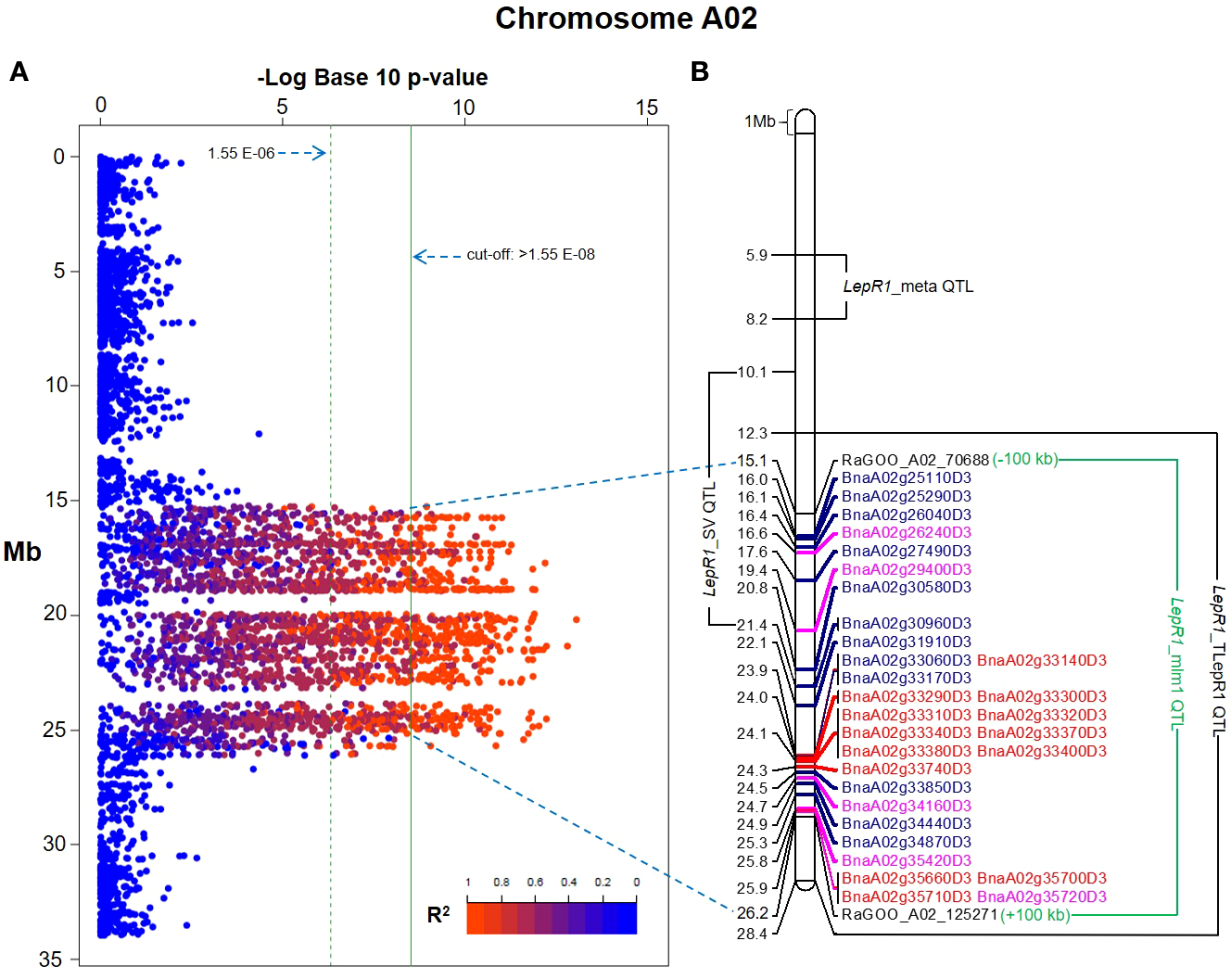
Mixed linear model (MLM)- genome-wide association study (GWAS) Manhattan plot in chromosome A02 of *Brassica napus* cv. Darmor bzh v9 showing SNPs and their linkage disequilibrium estimates in R^2^ **(A)**. Physical map of the flanking significant SNPs, RaGOO_A02_70688 and _125271, delineating the current quantitative trait loci (QTL) along its resistance gene analogs (RGAs) (red font=nucleotide binding site leucine rich repeats, blue font=receptor-like protein kinase, pink font=transmembrane-coiled coil) and previous QTL associated to *LepR1* blackleg resistance in *Brassica napus* **(B)**.

In MLM GWAS integrated with PCA + Kinship results (**Figures S3**-**4**), a total of 2,166 significant SNPs associated with *LepR1* resistance were identified across 10 chromosomes (2,108 SNPs on A02, 26 SNPs on C02, 7 SNPs each on A05 and C06, 6 SNPs on A08, 5 SNPs on C08, 3 SNPs on A07, 2 SNPs each on A04 and C01, and a SNP in C07) at the 1.58 E-08 cut-off value, implemented with Bonferroni correction and FDR (**Figures 1**–**2**; **Tables S4**). Significant SNPs had a range of 11.7 – 23.14% PVE (**Table S4**). The additional FarmCPU GWAS detected 7 significant SNPs but only one SNP (RaGOO_A02_103419) was found significant in the two different GWAS models (**Table S5**, **Figure S5**).

From the significant SNPs in MLM GWAS, we derived 15 QTL (containing 3,055 genes in total), however only 9 of them contained RGAs (**Table 1**, **S6**) and were considered further. These 9 QTL were located on chromosomes A02, C01 and C02, harbouring 62 RGAs in total (28 NLRs (12 TXs, 9 TNLs, 5 NLs, 4 NBS, 2 CNLs and 2 CN), 24 RLKs, 14 TM-CCs, and 3 RLPs) (**Tables 1**, **2**; **S7**)

**Table 1:** Delineation of the *LepR1*_mlm1 quantitative trait loci (QTL) including number of genes and the resistance gene analogs (RGAs) content using the mixed linear model (MLM) genome-wide association study (GWAS) and its significant single nucleotide polymorphism (SNP) associated with *LepR1* blackleg resistance in *Brassica napus*.

QTL name		*LepR1*_mlm1
**Chromosome (Position (Mb))**		A02 (15.11-26.18)
**Significant SNPs delineating the QTL**		RaGOO_A02_70688 and 125271 (flanking SNPs)
**Total # of SNPs ≤ E-05 (# of SNPs ≤ 1.58 E-08) in the QTL**		15331 SNPs (2108 SNPs)
**Total SNPs within the QTL**		54584 SNPs
**Genetic information within the QTL intervals**	**Total RGAs**	30
	**Total genes**	1235

**Table 2:** List of resistance gene analogs (RGA) and their single nucleotide polymorphism (SNP) information within *LepR1*_mlm1 on chromosome A02 of *Brassica napus* cv. Darmor bzh v9 identified using mixed linear (MLM) genome wide association study (GWAS).

Gene	RGA class	SNP					
		Total	Non-CDS	CDS	S^@^	M^@^	N^@^
BnaA02g25110D3	RLK	12	0	12	4	8	0
BnaA02g25290D3	RLK	45	14	31	9	20	2
BnaA02g26040D3	RLK	16	8	8	4	4	0
BnaA02g26240D3	TM-CC	0	0	0	0	0	0
BnaA02g27490D3	RLK	14	9	5	5	0	0
BnaA02g29400D3	TM-CC	1	0	1	1	0	0
BnaA02g30580D3	RLK	3	0	3	0	2	1
BnaA02g30960D3	RLK	1	0	1	1	0	0
BnaA02g31910D3	RLK	11	0	11	5	6	0
BnaA02g33060D3	RLK	0	0	0	0	0	0
BnaA02g33140D3	TX*	0	0	0	0	0	0
BnaA02g33170D3	RLK	4	4	0	0	0	0
BnaA02g33290D3	TX*	6	2	4	4	0	0
BnaA02g33300D3	TNL*	29	28	1	0	1	0
BnaA02g33310D3	TNL*	21	10	11	11	0	0
BnaA02g33320D3	TN*	14	4	10	9	1	0
BnaA02g33340D3	TNL*	0	0	0	0	0	0
BnaA02g33370D3	TX*	0	0	0	0	0	0
BnaA02g33380D3	TNL*	0	0	0	0	0	0
BnaA02g33400D3	TN*	0	0	0	0	0	0
BnaA02g33740D3	CN*	1	0	1	1	0	0
BnaA02g33850D3	RLK	3	1	2	0	2	0
BnaA02g34160D3	TM-CC	19	8	11	4	6	1
BnaA02g34440D3	RLK	12	8	4	4	0	0
BnaA02g34870D3	RLK	0	0	0	0	0	0
BnaA02g35420D3	TM-CC	34	32	2	0	2	0
BnaA02g35660D3	CNL*	0	0	0	0	0	0
BnaA02g35700D3	CNL*	0	0	0	0	0	0
BnaA02g35710D3	NL*	0	0	0	0	0	0
BnaA02g35720D3	TM-CC	0	0	0	0	0	0

*, belongs to nucleotide binding site-leucine rich repeats main class; CDS,coding region; S,synonymous; M, missense; N, nonsense; ^@^, most common allele in the SNP.

### A02 as the chromosome for *LepR1* in *B. napus*

This study showed 97% (2,108 out of 2,167 SNPs) of the total significant SNPs associated with *LepR1* blackleg resistance were found in chromosome A02 in *B. napus* cv. Darmor bzh v9 (**Figure 1**; **Table S3**). The delineated QTL on A02 (*LepR1*_mlm1) was 11.07 Mb in length (15.11-26.18 Mb) (**Figure 2**). Analysis of LD revealed closely associated SNPs within the QTL interval with a R^2^ ranging between 0.802-0.923 (**Figure 2**; **Table S4**).

The QTL in other studies were based on the *LepR1* introgression line (cv. Topas), mapped in genome cv. Darmor bzh v4.1 (Larkan et al., 2016), the structural variants of *LepR1* candidates mapped in cv. Darmor bzh v8.1 (Dolatabadian et al., 2019), and meta-analysis of blackleg resistance mapped in cv. Darmor bzh v4.1 (Fikere et al., 2020b), which were named in this study as *LepR1*_TLepR1, *LepR1*_SV, and *LepR1*_meta, respectively (**Figure 2**; **Table 3**). In Darmor bzh v9, *LepR1*_TLepR1, *LepR1*_SV, and *LepR1*_meta were found at 12.27-28.44 Mb totalling 16.17 Mb size, 10.09-21.4 Mb totalling 11.31 Mb size, and 5.91-8.22 Mb totalling 2.31 Mb size in A02, respectively (**Figure 2**). It can be noted that *LepR1*_mlm1 is shorter compared to the sizes of the *LepR1*_TLepR1 and *LepR1*_SV, but larger than the QTL outlier, *LepR1*_meta. Between the positions of these QTL, *LepR1*_mlm1 was nearer to *LepR1*_TLepR1 and *LepR1*_SV than *LepR1*_meta (**Figure 2**). *LepR1*_mlm1 contained 1235 genes (30 RGAs), *LepR1*_TLepR1, *LepR1*_SV, and *LepR1*_meta contained 1915 genes (46 RGAs), 344 genes (8 RGAs), and 1434 genes (20 RGAs), respectively (**Tables 1**, **3**). Of the previous QTL, only *LepR1*_TLepR1 and *LepR1*_SV had an overlapping or co-localised position with *LepR1*_mlm1 (**Figure 2**). Eight out of 30 RGAs in *LepR1*_mlm1 (RLKs BnaA02g25110D3, BnaA02g25290D3, BnaA02g26040D3, BnaA02g27490D3, BnaA02g30580D3, and BnaA02g30960D3, TM-CCs BnaA02g26240D3 and BnaA02g29400D3) were also within the intervals of the *LepR1*_TLepR1 and *LepR1*_SV (**Figure 2**).

**Table 3:** Alignment (basic local alignment sequence tool or BLAST) of the flanking genes from previous *LepR1* quantitative trait loci (QTL) in chromosome A02 of *Brassica napus* to Darmor bzh v9.

Flanking genes in previous *LepR1* QTLs (designated QTL name in this study)	Closest genomic Feature	Position	Perc ID	Score	Information within the QTL intervals	
					Total RGAs	Total genes
BnaA02g15610.1D2^a^ (upstream gene of *LepR1*_SV QTL)	BnaA02g16670D3	10089392	100%	2977	20	1434
BnaA02g23050.1D2^a^ (downstream gene of *LepR1*_SV QTL)	BnaA02g31000D3	21404096	99.9%	9694		
BnaA02g09140D^b^ (upstream gene of *LepR1*_meta QTL)	BnaA02g10850D3	5911085	100%	8685	8	344
BnaA02g12340D^b^ (downstream gene of *LepR1*_meta QTL)	BnaA02g14280D3	8221378	100%	3530		
BnaA02g16780D^c^ (upstream gene of *LepR1*_TLepR1 QTL)	BnaA02g20040D3	12269388	99.9%	13350	46	1915
BnaA02g27650D^c^ (downstream gene of *LepR1*_TLepR1 QTL)	BnaA02g39180D3	28437140	100%	1067		

Source: ^a^(Dolatabadian et al., 2019), ^b^(Fikere et al., 2020b), ^c^(Larkan et al., 2016). All BLAST results obtained E-values of “0”and 100% sequence coverage.

Eighteen (60%) out of the 30 RGAs underlying *LepR1*_mlm1 contained SNPs (246 SNPs in total), with 17 having SNPs in the CDS region (CDS-SNPs) (118 CDS-SNPs in total) (**Table 2**). BnaA02g25290D3 (RLK) contained the most CDS-SNPs, 31. The next highest number of CDS-SNPs in a *LepR1* candidate gene were 12 in BnaA02g25110D3 (RLK), 11 in BnaA02g31910D3 (RLK), BnaA02g33310D3 (RLK) and BnaA02g34160D3 (TM-CC), 10 in BnaA02g33320D3 (NLR) and 8 in BnaA02g26040D3 (RLK) (**Table 2**). Among the candidate genes, only BnaA02g25290D3, BnaA02g30580D3 (RLK), and BnaA02g34160D3 contained nonsense SNPs. For instance, BnaA02g25290D3 had the “T” allele of RaGOO_A02_75077 (16,131,870 bp in A02) and RaGOO_A02_75078 (16,131,906 bp in A02) was identified as a STOP CODON, “TGA” (**Table S7**). The positions of RaGOO_A02_75077 and RaGOO_A02_75078 correspond to the 16,131,870-16,131,872 bp and 16,131,906-16,906,908 bp with a three-nucleotide “AGA” (Arginine) in reference to the Darmor bzh v9 (**Table S8**). BnaA02g25290D3 also contained the highest number of possible missense SNP alleles, 20, followed by 8 in BnaA02g25110D3 and 6 to both BnaA02g31910D3 and BnaA02g34160D3 (**Table 2**).

### Molecular analysis in *LepR1* resistant and susceptible materials

Only 13 *LepR1* candidates (BnaA02g25110D3, BnaA02g25290D3, BnaA02g26040D3, BnaA02g27490D3, BnaA02g30580D3, BnaA02g31910D3, BnaA02g33290D3, BnaA02g33310D3, BnaA02g33320D3, BnaA02g34160D3, BnaA02g34440D3, BnaA02g33850D3, and BnaA02g35420D3) had at least 4 SNPs in their CDS regions between resistant and susceptible lines (**Table 2**). Among the 13 selected *LepR1* candidates and the 2 flanking RGAs, only BnaA02g33310D3, BnaA02g33850D3, BnaA02g34440D3, and the flanking RGA BnaA02g25030D3 were reproducibly amplified (**Table S9**), which were sent for sequencing analysis.

BnaA02g33310D3 and BnaA02g25030D3 had a sequence difference between the resistant and susceptible alleles, while BnaA02g33850D3 and BnaA02g34440D3 had similar sequences throughout the tested materials. SNP differences among the alleles of the resistant cultivars was also observed in BnaA02g25030D3. In all, BnaA02g33310D3 had 92 SNP differences between the resistant and susceptible *B. napus* cultivars (**Figure S5**). There were also two large deletions in positions 697 to 706 bp and 2836 to 2854 bp observed in the resistant allele, while there was one large deletion in position 2881 and 2894 bp in the susceptible allele (**Figure S5**). For the resistant-specific marker developed based on the sequencing result (**Table S10**), 22 out of 25 *LepR1* resistant cultivars had a band while no band was detected in the 31 susceptible cultivars (**Figure S6**).

## Discussion

*Leptosphaeria maculans* isolates containing specific *Avr* gene profiles are widely used either to verify known blackleg *R* genes or discover novel blackleg *R* genes in *B. napus* (Balesdent et al., 2005; Van de Wouw et al., 2009; Marcroft et al., 2012; Jiquel et al., 2021). However, a mild or inconsistent hypersensitive response in *B. napus* cultivars can be observed due to different genomic backgrounds (Rouxel et al., 2003; Balesdent et al., 2005; Larkan et al., 2016). Nevertheless, *LepR1* is a single dominant gene and its interaction to *AvrLep1* interaction in *B. napus* follows a gene-for-gene relationship (Yu et al., 2002; Yu et al., 2005). Thus, the phenotype data from *L. maculans* isolates containing *AvrLep1* confirmed the presence of the *R* gene in the resistant cultivars and the absence of the *R* gene in the susceptible cultivars in this study.

The use of WGRS in crop studies is advantageous because it can identify a large number of SNPs that are densely dispersed throughout the entire genome, therefore improving the ability for GWAS to identify functional QTL. The genotype data used here comprised over 3 million SNP markers, which has a better coverage and was more dense compared to low coverage markers such as RFLPs, SSRs, the *Brassica* Infinium 60K SNP array, and imputed SNPs, that were used to detect previous *LepR1* QTL in *B. napus* (Yu et al., 2005; Yu et al., 2012; Larkan et al., 2016; Fikere et al., 2020b). In previous studies which had defined positions of the *LepR1* QTL, they used previous versions (with different quality and assembly size) of Darmor bzh genomes (v4.1 or v8.1). For instance, Darmor bzh v9 has a total size of 1,043.4 Mb compared to the 850.3 Mb of Darmor bzh v4.1 (Bayer et al., 2020).

GWAS is commonly used to reveal genetic mechanisms underlying disease resistance as it integrates historical recombination of the different cultivars and thus, uncovers significant associations between genotype and phenotype. Among the GWAS models, MLM is widely regarded as the most common model in analysing marker trait associations in crop research. Kinship and PCA results were integrated into the MLM to overcome any bias from genetic ancestry (relatedness) (Price et al., 2006; Yu et al., 2006; VanRaden, 2008).

*LepR1*_meta was based on adult plant survival rate and average internal infection of the stem at maturity stage paired with imputed SNPs, a methodology that would identify quantitative instead of qualitative blackleg resistance in *B. napus* (Raman et al., 2018; Fikere et al., 2020b). This is likely the reason why *LepR1*_meta was located 6.89 Mb away from *LepR1*_mlm1 of this study. Our *LepR1*_mlm1 corresponds to qualitative blackleg resistance. Similarly, *LepR1*_TLepR1 was a result from *L. maculans* containing *AvrLep1* screened at the cotyledon stage of the *LepR1* introgressed lines (Larkan et al., 2016). *LepR1*_TLepR1 was also used as the basis to define structural variants of *LepR1* in *LepR1*_SV (Dolatabadian et al., 2019). Taken together, our QTL was consistent with previous *LepR1* QTL based on gene-for-gene screening which were all mapped to A02 (Larkan et al., 2016; Dolatabadian et al., 2019; Fikere et al., 2020b).

The few significant SNP associations on C01 and C02 (having RGAs in the QTL) could possibly be due the incorrect mapping of DNA sequences containing notable SNPs that occurred during the genome assembly process in *B. napus* as SNPs were commonly observed in a genome due to their abundance. Another reason is due to homology between chromosomes, as C02 is homeologous to A02 in *B. napus* (Parkin et al., 2005). Another reason could be that some genes might have a role (for example as a helper or decoy gene) in the gene-for-gene interaction between *LepR1* and *AvrLep1*. RNA sequencing in previous studies has revealed several significantly activated genes in the *LepR1-AvrLep1* interaction on chromosomes C01 and C02 (Becker et al., 2017; Haddadi et al., 2019), indicating the possible roles of other genes in the *LepR1-AvrLep1* interaction in *B. napus*. Recent findings in the *B. napus-L. maculans* pathosystem indicate that the simple gene-for-gene interaction may indeed be more complicated than once thought (Cantila et al., 2021). Functional gene studies revealed that *B. napus mitogen-activated protein (MAP) kinase 9 (BnMPK9)*, BnaCnng11720D or C05p48150.1_BnaDar on C05 (Rousseau-Gueutin et al., 2020), is an essential partner to the gene-for-gene mediated resistance of blackleg *R* gene *Rlm1* on A07 and pathogen effector *AvrLm1* (Ma et al., 2018). Furthermore, *Suppresor of BAK1-interacting receptor like kinase 1 (SOBIR1)* genes on A03 and C03 (BnaA03g14760D and BnaC03g17800D) are also required for the blackleg resistance of the alleles *LepR3* and *Rlm2* (BnaA10g20720D) of A10 to effectors *AvrLm1* and *AvrLm2*, respectively (Larkan et al., 2015; Ma et al., 2018).

RGAs with CDS-SNPs are promising candidates for the *LepR1* gene. The CDS-SNPs of these RGAs could alter amino acids (aa) of the predicted proteins explaining the difference between susceptible and resistant phenotypes. Among the SNP types, a nonsense SNP leads to a premature stoppage of the amino acids, potentially changing the gene expression among cultivars with different genotypes. Although this study did not examine gene expression between cultivars with different *LepR1* phenotypes, previous studies have shown that RLKs and NLRs were significantly expressed in *LepR1* resistant cultivars (Becker et al., 2017; Ferdous et al., 2020).

Among the candidates, only BnaA02g33310D3 showed interesting SNP variation, including large deletions, between *LepR1* resistant and susceptible materials, indicating the gene may be considered further as a candidate gene. Large deleted sequences were also found in functionally characterised (mutated) genes *RPM1* against *Pseudomonas syringae* (Grant et al., 1998) and *LepR3/Rlm2* against *L. maculans* (Larkan et al., 2013; Larkan et al., 2015) compared to its respective wild gene. Also, SNP variation has been the basis to develop specific markers with blackleg resistance (Van de Wouw et al., 2022). This study’s resistant-specific marker for BnaA02g33310D3 also signified the gene as a candidate in *B. napus* and will be useful for MAS. On the other hand, the other candidates which did not have successful band detection can still be considered candidates for the *LepR1* but they require further analysis.

## Conclusion

This study has narrowed down the *LepR1* resistance in *B. napus* by comparing the current QTL to the previous *LepR1* QTLs. While all RGAs in *LepR1*_mlm1 are promising candidates, those co-localised within the intervals in previous *LepR1* QTLs aligned in Darmor bzh v9 and having CDS-SNPs, especially with nonsense SNPs, should be prioritised for validation. In addition, RGAs in the QTLs identified on chromosomes other than A02 in this study can be tested for the possible roles of accessory, helper or decoy genes in *LepR1* gene-for-gene resistance in *B. napus.* Our identified RGAs especially BnaA02g33310D3 are a useful resource to identify the functional *LepR1* gene in *B. napus*.

*LepR1* can also be an significant source of blackleg resistance for the cultivars in Australia, which has virulent *L. maculans* population causing the breakdown of the cultivars containing *R* gene like *LepR3* and ineffectiveness of *R* genes like *Rlm1* and *Rlm3* (Li et al., 2005; Van de Wouw et al., 2014; Van de Wouw et al., 2021). Other studies have also shown that cultivars with *LepR1* are more effective than those with *R* genes *Rlm1, Rlm3, Rlm4*, and *LepR3* (Alnajar et al., 2022; Rashid et al., 2022).

## Data availability statement

The datasets presented in this study can be found in online repositories. The names of the repository/repositories and accession number(s) can be found below: NCBI accession PRJNA885991.

## Author contributions

AC and JB conceptualized the paper. AC wrote the original draft along with formal analyses. WT, PB, DE, and AV helped improve the paper by suggesting additional ideas and by thorough revision/editing. AC and NS planted the *B. napus* materials including DNA extraction. AV provided the phenotype data. AS-E, NS and RA provided bioinformatics support by calling SNPs on the reference *B. napus* cv. Darmor-bzh v9 genome and statistical codes. AC designed the primers/markers and prepared the materials for sequencing analysis. JB supervised, reviewed, and suggested revisions to the paper. All authors contributed to the article and approved the submitted version.
