# Supplementary material for: Identification of candidate genes for LepR1 resistance against Leptosphaeria maculans in Brassica napus
Source: Front Plant Sci. 2023 Feb 14;14:1051994. doi: 10.3389/fpls.2023.1051994 (PMC9971972; doi:10.3389/fpls.2023.1051994)
Supplement: Supplementary file 1 [file DataSheet_1.docx]

Supplementary Materials

**Tables**

**Table S1.** *Leptosphaeria maculans* isolates used in this study to determine the presence or absence of *Brassica napus LepR1* resistance gene.

| **Isolate** | ***AvrLep1* genotype** |
| --- | --- |
| D2 | *AvrLep1* |
| D3 | *AvrLep1* |
| D13 | *avrLep1* |
| D14 | *AvrLep1* |
| D16 | *avrLep1* |
| D17 | *avrLep1* |
| D18 | *AvrLep1* |
| D19 | *AvrLep1* |
| D20 | *avrLep1* |
| D21 | *avrLep1* |
| D22 | *avrLep1* |
| D23 | *avrLep1* |
| D24 | *AvrLep1* |
| D25 | *AvrLep1* |

| **Table S2**. List of cultivars used in this study along their cultivar type information and phenotypic result screened to blackleg isolates containing *AvrLep1* . | | |
| --- | --- | --- |
| **Cultivars** | **Cultivar type** | **Phenotypic result based on blackleg isolates containing *AvrLep1* gene** |
| Hyola 350TT, Hyola 404RR, Hyola 410XX, Hyola 444TT, Hyola 450, Hyola 50, Hyola 506 RR, Hyola 525 RT, Hyola 530 XT, Hyola 550 TT, Hyola 559 TT, Hyola 600 RR, Hyola 650, Hyola 725 RT, HyTTec Trident, HyTTec Trophy, NCH16G342 (HyTTec Trifecta), NEX9080 TT, NL1015 (Monola 420TT), NT0218 (ATR Flathead), Nuseed GT42, Nuseed GT53, Nuseed Quartz, Pioneer 44T02TT, Pioneer 45T03TT, & SF SparkTT | Commercial cultivars | 1 |
| ABL1, ABL2, ABL3, & ABL4 | Advanced breeding lines | 1 |
| ATR Bonito, ATR Mako, ATR Wahoo, Archer, Banker CL, BASF 3000TR, Carbine, DG 408RR, DG 460RR, DG 560TT, DG 670TT, Edimax CL, Hyola 474CL, Hyola 540 XC, Hyola 575 CL, Hyola 577 CL, Hyola 580 CT, Hyola 970, IH 30, IH 51RR, IH 52, InVigor R3520, InVigor R4022P, InVigor R5520P,  InVigor T3510, InVigor T4510, Monola 515TT, Monola G11, Nuseed Diamond, Nuseed GT41, Nuseed GT50, Phoenix CL, Pioneer 43Y23RR, Pioneer 43Y29RR, Pioneer 43Y92CL, Pioneer 44Y24RR, Pioneer 44Y27RR, Pioneer 44Y89CL, Pioneer 44Y90CL, Pioneer 45T01TT, Pioneer 45Y25RR, Pioneer 45Y88CL, Pioneer 45Y91CL, Pioneer 45Y93CL, Saintly CL, SF Ignite TT, SFR65-028TT, SF TurbineTT, Victory V3002, Victory V5003RR, Victory V7001CL, Victory V7002CL, Victory V75.03CL, BLN3347, Crusher TT, DG 1902TT (DG MurrayTT), DG 1903TT (DG BidgeeTT), Monola 416TT,  Mustang, Pioneer 43C80CL, Pioneer Sturt, SF Brazzil, Surpass 501TT, Surpass 603CL, Thunder TT, & Yetna | Commercial cultivars | 0 |
| ABL5, ABL6, ABL7, ABL8, ABL9, ABL10, ABL11, & ABL12 | Advanced breeding lines | 0 |
| ABL=Advanced breeding line | | |

**Table S3**. Single nucleotide polymorphism (SNPs) statistics computed by TASSEL 5.0.

| **Stat Type** | **Value** |
| --- | --- |
| Number of Taxa (*Brassica napus* genotypes) | 104 |
| Number of Sites (SNPs) | 3235008 |
| Sites x Taxa | 336440000 |
| Number Not Missing | 321404000 |
| Proportion Not Missing | 0.955308525 |
| Number Missing | 15037695 |
| Proportion Missing | 0.044696513 |
| Number Gametes | 672883000 |
| Gametes Not Missing | 642801000 |
| Proportion Gametes Not Missing | 0.955293862 |
| Gametes Missing | 30075100 |
| Proportion Gametes Missing | 0.044695883 |
| Number Heterozygous | 44508900 |
| Proportion Heterozygous | 0.132293723 |
| Average Heterozygous SNPs per B. napus genotype | 427970 |

**Table S4**. Genome wide association study (GWAS) in mixed linear model showing the 2166 significant SNPs above the GWAS threshold 1.58E-08 associated to *LepR1* blackleg resistance in *Brassica napus* cv. Darmor bzh v9.

| **SNP** | **Chromosome** | **Position** | **P-value** | **Minor allele frequency** | **Number of**  **observation** | **R^2^ Model without**  **SNP** | **R^2^ Model with SNP** | **FDR Adjusted**  **P-values** | **PVE (%)** |
| --- | --- | --- | --- | --- | --- | --- | --- | --- | --- |
| RaGOO_A02_97627 | A02 | 20185942 | 8.70E-14 | 0.135 | 104 | 0.692 | 0.923 | 9.34E-08 | 23.1 |
| RaGOO_A02_103419 | A02 | 21340791 | 1.54E-13 | 0.135 | 104 | 0.692 | 0.917 | 1.24E-07 | 22.5 |
| RaGOO_A02_82450 | A02 | 17229004 | 2.58E-13 | 0.135 | 104 | 0.692 | 0.912 | 1.58E-07 | 22.0 |
| RaGOO_A02_103420 | A02 | 21340792 | 5.77E-13 | 0.139 | 104 | 0.692 | 0.904 | 1.58E-07 | 21.2 |
| RaGOO_A02_115795 | A02 | 24517935 | 5.80E-13 | 0.168 | 104 | 0.692 | 0.904 | 1.58E-07 | 21.2 |
| RaGOO_A02_85672 | A02 | 17718872 | 6.29E-13 | 0.183 | 104 | 0.692 | 0.903 | 1.58E-07 | 21.1 |
| RaGOO_A02_103842 | A02 | 21497050 | 7.07E-13 | 0.183 | 104 | 0.692 | 0.901 | 1.58E-07 | 20.9 |
| RaGOO_A02_103849 | A02 | 21497330 | 8.98E-13 | 0.183 | 104 | 0.692 | 0.899 | 1.58E-07 | 20.7 |
| RaGOO_A02_117926 | A02 | 24824385 | 9.29E-13 | 0.163 | 104 | 0.692 | 0.899 | 1.58E-07 | 20.7 |
| RaGOO_A02_103843 | A02 | 21497087 | 1.03E-12 | 0.178 | 104 | 0.692 | 0.898 | 1.58E-07 | 20.6 |
| RaGOO_A02_91910 | A02 | 18882301 | 1.18E-12 | 0.178 | 104 | 0.692 | 0.896 | 1.58E-07 | 20.4 |
| RaGOO_A02_95992 | A02 | 19942378 | 1.24E-12 | 0.178 | 104 | 0.692 | 0.896 | 1.58E-07 | 20.4 |
| RaGOO_A02_109713 | A02 | 22662292 | 1.34E-12 | 0.168 | 104 | 0.692 | 0.895 | 1.58E-07 | 20.3 |
| RaGOO_A02_104320 | A02 | 21564486 | 1.36E-12 | 0.163 | 104 | 0.692 | 0.895 | 1.58E-07 | 20.3 |
| RaGOO_A02_86929 | A02 | 17983213 | 1.37E-12 | 0.173 | 104 | 0.692 | 0.895 | 1.58E-07 | 20.3 |
| RaGOO_A02_97237 | A02 | 20133436 | 1.37E-12 | 0.173 | 104 | 0.692 | 0.895 | 1.58E-07 | 20.3 |
| RaGOO_A02_103847 | A02 | 21497227 | 1.37E-12 | 0.173 | 104 | 0.692 | 0.895 | 1.58E-07 | 20.3 |
| RaGOO_A02_106128 | A02 | 21928708 | 1.37E-12 | 0.173 | 104 | 0.692 | 0.895 | 1.58E-07 | 20.3 |
| RaGOO_A02_118198 | A02 | 24863222 | 1.37E-12 | 0.173 | 104 | 0.692 | 0.895 | 1.58E-07 | 20.3 |
| RaGOO_A02_91694 | A02 | 18861285 | 1.37E-12 | 0.173 | 104 | 0.692 | 0.895 | 1.58E-07 | 20.3 |
| RaGOO_A02_91724 | A02 | 18863362 | 1.37E-12 | 0.173 | 104 | 0.692 | 0.895 | 1.58E-07 | 20.3 |
| RaGOO_A02_97096 | A02 | 20105213 | 1.37E-12 | 0.173 | 104 | 0.692 | 0.895 | 1.58E-07 | 20.3 |
| RaGOO_A02_97242 | A02 | 20133773 | 1.37E-12 | 0.173 | 104 | 0.692 | 0.895 | 1.58E-07 | 20.3 |
| RaGOO_A02_103844 | A02 | 21497116 | 1.37E-12 | 0.173 | 104 | 0.692 | 0.895 | 1.58E-07 | 20.3 |
| RaGOO_A02_113912 | A02 | 24235501 | 1.50E-12 | 0.173 | 104 | 0.692 | 0.894 | 1.63E-07 | 20.2 |
| RaGOO_A02_98122 | A02 | 20273271 | 1.52E-12 | 0.163 | 104 | 0.692 | 0.894 | 1.63E-07 | 20.2 |
| RaGOO_A02_115690 | A02 | 24501139 | 1.76E-12 | 0.173 | 104 | 0.692 | 0.892 | 1.70E-07 | 20.0 |
| RaGOO_A02_101913 | A02 | 21040501 | 1.81E-12 | 0.168 | 104 | 0.692 | 0.892 | 1.70E-07 | 20.0 |

| RaGOO_A02_119577 | A02 | 25152714 | 1.81E-12 | 0.168 | 104 | 0.692 | 0.892 | 1.70E-07 | 20.0 |
| --- | --- | --- | --- | --- | --- | --- | --- | --- | --- |
| RaGOO_A02_106107 | A02 | 21926166 | 1.81E-12 | 0.168 | 104 | 0.692 | 0.892 | 1.70E-07 | 20.0 |
| RaGOO_A02_99642 | A02 | 20567750 | 1.84E-12 | 0.163 | 104 | 0.692 | 0.892 | 1.70E-07 | 20.0 |
| RaGOO_A02_91695 | A02 | 18861317 | 2.08E-12 | 0.168 | 104 | 0.692 | 0.891 | 1.86E-07 | 19.9 |
| RaGOO_A02_104321 | A02 | 21564532 | 2.37E-12 | 0.178 | 104 | 0.692 | 0.889 | 2.02E-07 | 19.7 |
| RaGOO_A02_103845 | A02 | 21497176 | 2.38E-12 | 0.168 | 104 | 0.692 | 0.889 | 2.02E-07 | 19.7 |
| RaGOO_A02_120961 | A02 | 25417257 | 2.45E-12 | 0.168 | 104 | 0.692 | 0.889 | 2.03E-07 | 19.7 |
| RaGOO_A02_110239 | A02 | 22784579 | 2.75E-12 | 0.163 | 104 | 0.692 | 0.888 | 2.21E-07 | 19.6 |
| RaGOO_A02_110978 | A02 | 22913810 | 2.97E-12 | 0.178 | 104 | 0.692 | 0.887 | 2.34E-07 | 19.5 |
| RaGOO_A02_97638 | A02 | 20186621 | 4.23E-12 | 0.163 | 104 | 0.692 | 0.884 | 3.24E-07 | 19.1 |
| RaGOO_A02_99861 | A02 | 20625774 | 4.48E-12 | 0.178 | 104 | 0.692 | 0.883 | 3.35E-07 | 19.1 |
| RaGOO_A02_91650 | A02 | 18858809 | 5.31E-12 | 0.188 | 104 | 0.692 | 0.881 | 3.81E-07 | 18.9 |
| RaGOO_A02_80262 | A02 | 16929432 | 5.32E-12 | 0.163 | 104 | 0.692 | 0.881 | 3.81E-07 | 18.9 |
| RaGOO_A02_111112 | A02 | 22934119 | 5.51E-12 | 0.159 | 104 | 0.692 | 0.881 | 3.83E-07 | 18.9 |
| RaGOO_A02_97241 | A02 | 20133637 | 5.59E-12 | 0.178 | 104 | 0.692 | 0.881 | 3.83E-07 | 18.9 |
| RaGOO_A02_103846 | A02 | 21497197 | 5.77E-12 | 0.178 | 104 | 0.692 | 0.880 | 3.87E-07 | 18.8 |
| RaGOO_A02_100696 | A02 | 20856231 | 6.10E-12 | 0.183 | 104 | 0.692 | 0.880 | 3.96E-07 | 18.8 |
| RaGOO_A02_97238 | A02 | 20133437 | 6.15E-12 | 0.178 | 104 | 0.692 | 0.880 | 3.96E-07 | 18.8 |
| RaGOO_A02_82308 | A02 | 17220415 | 6.38E-12 | 0.183 | 104 | 0.692 | 0.879 | 4.03E-07 | 18.7 |
| RaGOO_A02_91913 | A02 | 18882464 | 6.72E-12 | 0.173 | 104 | 0.692 | 0.879 | 4.16E-07 | 18.7 |
| RaGOO_A02_91487 | A02 | 18846419 | 6.99E-12 | 0.178 | 104 | 0.692 | 0.879 | 4.18E-07 | 18.7 |
| RaGOO_A02_101381 | A02 | 20972005 | 7.01E-12 | 0.159 | 104 | 0.692 | 0.879 | 4.18E-07 | 18.7 |
| RaGOO_A02_89561 | A02 | 18529296 | 8.11E-12 | 0.178 | 104 | 0.692 | 0.877 | 4.64E-07 | 18.5 |
| RaGOO_A02_91862 | A02 | 18877955 | 8.11E-12 | 0.178 | 104 | 0.692 | 0.877 | 4.64E-07 | 18.5 |
| RaGOO_A02_98124 | A02 | 20273610 | 8.21E-12 | 0.178 | 104 | 0.692 | 0.877 | 4.64E-07 | 18.5 |
| RaGOO_A02_89559 | A02 | 18529056 | 8.62E-12 | 0.178 | 104 | 0.692 | 0.877 | 4.79E-07 | 18.4 |
| RaGOO_A02_89524 | A02 | 18524019 | 8.82E-12 | 0.173 | 104 | 0.692 | 0.876 | 4.81E-07 | 18.4 |
| RaGOO_A02_79874 | A02 | 16884196 | 9.00E-12 | 0.173 | 104 | 0.692 | 0.876 | 4.82E-07 | 18.4 |
| RaGOO_A02_99277 | A02 | 20505036 | 9.12E-12 | 0.173 | 104 | 0.692 | 0.876 | 4.82E-07 | 18.4 |
| RaGOO_A02_91726 | A02 | 18863470 | 9.43E-12 | 0.178 | 104 | 0.692 | 0.876 | 4.89E-07 | 18.4 |
| RaGOO_A02_111074 | A02 | 22919892 | 9.57E-12 | 0.178 | 104 | 0.692 | 0.875 | 4.89E-07 | 18.3 |
| RaGOO_A02_73180 | A02 | 15739617 | 9.73E-12 | 0.168 | 104 | 0.692 | 0.875 | 4.90E-07 | 18.3 |
| RaGOO_A02_92083 | A02 | 18898818 | 1.06E-11 | 0.168 | 104 | 0.692 | 0.875 | 5.08E-07 | 18.3 |
| RaGOO_A02_119574 | A02 | 25152555 | 1.06E-11 | 0.178 | 104 | 0.692 | 0.874 | 5.08E-07 | 18.2 |
| RaGOO_A02_97628 | A02 | 20186115 | 1.07E-11 | 0.178 | 104 | 0.692 | 0.874 | 5.08E-07 | 18.2 |
| RaGOO_A02_104310 | A02 | 21563992 | 1.07E-11 | 0.178 | 104 | 0.692 | 0.874 | 5.08E-07 | 18.2 |
| RaGOO_A02_80166 | A02 | 16919417 | 1.14E-11 | 0.173 | 104 | 0.692 | 0.874 | 5.24E-07 | 18.2 |
| RaGOO_A02_119573 | A02 | 25152533 | 1.14E-11 | 0.173 | 104 | 0.692 | 0.874 | 5.24E-07 | 18.2 |
| RaGOO_A02_103416 | A02 | 21340466 | 1.23E-11 | 0.183 | 104 | 0.692 | 0.873 | 5.31E-07 | 18.1 |
| RaGOO_A02_97637 | A02 | 20186619 | 1.26E-11 | 0.168 | 104 | 0.692 | 0.873 | 5.31E-07 | 18.1 |
| RaGOO_A02_97065 | A02 | 20101951 | 1.26E-11 | 0.178 | 104 | 0.692 | 0.873 | 5.31E-07 | 18.1 |
| RaGOO_A02_91540 | A02 | 18851948 | 1.26E-11 | 0.178 | 104 | 0.692 | 0.873 | 5.31E-07 | 18.1 |
| RaGOO_A02_91541 | A02 | 18851960 | 1.26E-11 | 0.178 | 104 | 0.692 | 0.873 | 5.31E-07 | 18.1 |
| RaGOO_A02_113743 | A02 | 24203466 | 1.27E-11 | 0.173 | 104 | 0.692 | 0.873 | 5.31E-07 | 18.1 |
| RaGOO_A02_110669 | A02 | 22846296 | 1.28E-11 | 0.183 | 104 | 0.692 | 0.873 | 5.31E-07 | 18.1 |
| RaGOO_A02_100886 | A02 | 20895302 | 1.31E-11 | 0.168 | 104 | 0.692 | 0.872 | 5.31E-07 | 18.0 |
| RaGOO_A02_101103 | A02 | 20940069 | 1.32E-11 | 0.183 | 104 | 0.692 | 0.872 | 5.31E-07 | 18.0 |
| RaGOO_A02_91908 | A02 | 18882041 | 1.38E-11 | 0.183 | 104 | 0.692 | 0.872 | 5.31E-07 | 18.0 |
| RaGOO_A02_97626 | A02 | 20185763 | 1.38E-11 | 0.183 | 104 | 0.692 | 0.872 | 5.31E-07 | 18.0 |
| RaGOO_A02_91821 | A02 | 18875412 | 1.39E-11 | 0.178 | 104 | 0.692 | 0.872 | 5.31E-07 | 18.0 |
| RaGOO_A02_97244 | A02 | 20133805 | 1.39E-11 | 0.178 | 104 | 0.692 | 0.872 | 5.31E-07 | 18.0 |
| RaGOO_A02_97243 | A02 | 20133798 | 1.39E-11 | 0.178 | 104 | 0.692 | 0.872 | 5.31E-07 | 18.0 |
| RaGOO_A02_123178 | A02 | 25686196 | 1.41E-11 | 0.154 | 104 | 0.692 | 0.872 | 5.33E-07 | 18.0 |
| RaGOO_A02_80092 | A02 | 16912979 | 1.46E-11 | 0.168 | 104 | 0.692 | 0.871 | 5.47E-07 | 17.9 |
| RaGOO_A02_73276 | A02 | 15757760 | 1.49E-11 | 0.163 | 104 | 0.692 | 0.871 | 5.52E-07 | 17.9 |
| RaGOO_A02_99750 | A02 | 20594697 | 1.61E-11 | 0.173 | 104 | 0.692 | 0.870 | 5.83E-07 | 17.8 |
| RaGOO_A02_98003 | A02 | 20234969 | 1.62E-11 | 0.163 | 104 | 0.692 | 0.870 | 5.83E-07 | 17.8 |
| RaGOO_A02_102951 | A02 | 21208889 | 1.74E-11 | 0.173 | 104 | 0.692 | 0.870 | 5.83E-07 | 17.8 |
| RaGOO_A02_103848 | A02 | 21497230 | 1.78E-11 | 0.168 | 104 | 0.692 | 0.870 | 5.83E-07 | 17.8 |
| RaGOO_A02_106127 | A02 | 21928704 | 1.78E-11 | 0.168 | 104 | 0.692 | 0.870 | 5.83E-07 | 17.8 |
| RaGOO_A02_75263 | A02 | 16171931 | 1.78E-11 | 0.168 | 104 | 0.692 | 0.870 | 5.83E-07 | 17.8 |
| RaGOO_A02_96457 | A02 | 20006748 | 1.90E-11 | 0.168 | 104 | 0.692 | 0.869 | 5.83E-07 | 17.7 |
| RaGOO_A02_99885 | A02 | 20629894 | 1.93E-11 | 0.163 | 104 | 0.692 | 0.869 | 5.83E-07 | 17.7 |
| RaGOO_A02_84478 | A02 | 17504529 | 1.93E-11 | 0.163 | 104 | 0.692 | 0.869 | 5.83E-07 | 17.7 |
| RaGOO_A02_82256 | A02 | 17218230 | 1.93E-11 | 0.173 | 104 | 0.692 | 0.869 | 5.83E-07 | 17.7 |
| RaGOO_A02_82258 | A02 | 17218286 | 1.93E-11 | 0.173 | 104 | 0.692 | 0.869 | 5.83E-07 | 17.7 |
| RaGOO_A02_82259 | A02 | 17218288 | 1.93E-11 | 0.173 | 104 | 0.692 | 0.869 | 5.83E-07 | 17.7 |
| RaGOO_A02_79953 | A02 | 16893714 | 1.93E-11 | 0.183 | 104 | 0.692 | 0.869 | 5.83E-07 | 17.7 |
| RaGOO_A02_80162 | A02 | 16919213 | 1.95E-11 | 0.178 | 104 | 0.692 | 0.869 | 5.83E-07 | 17.7 |
| RaGOO_A02_85725 | A02 | 17724609 | 1.97E-11 | 0.168 | 104 | 0.692 | 0.869 | 5.83E-07 | 17.7 |
| RaGOO_A02_97138 | A02 | 20116695 | 1.97E-11 | 0.168 | 104 | 0.692 | 0.869 | 5.83E-07 | 17.7 |
| RaGOO_A02_91962 | A02 | 18886462 | 1.97E-11 | 0.168 | 104 | 0.692 | 0.869 | 5.83E-07 | 17.7 |
| RaGOO_A02_91483 | A02 | 18846204 | 1.97E-11 | 0.163 | 104 | 0.692 | 0.869 | 5.83E-07 | 17.7 |
| RaGOO_A02_103435 | A02 | 21342066 | 2.00E-11 | 0.192 | 104 | 0.692 | 0.868 | 5.83E-07 | 17.6 |
| RaGOO_A02_92296 | A02 | 18918712 | 2.02E-11 | 0.168 | 104 | 0.692 | 0.868 | 5.83E-07 | 17.6 |
| RaGOO_A02_113113 | A02 | 24036424 | 2.02E-11 | 0.168 | 104 | 0.692 | 0.868 | 5.83E-07 | 17.6 |
| RaGOO_A02_108582 | A02 | 22423528 | 2.03E-11 | 0.168 | 104 | 0.692 | 0.868 | 5.83E-07 | 17.6 |
| RaGOO_A02_105764 | A02 | 21883517 | 2.04E-11 | 0.168 | 104 | 0.692 | 0.868 | 5.83E-07 | 17.6 |
| RaGOO_A02_99040 | A02 | 20476170 | 2.04E-11 | 0.168 | 104 | 0.692 | 0.868 | 5.83E-07 | 17.6 |
| RaGOO_A02_105791 | A02 | 21886248 | 2.06E-11 | 0.163 | 104 | 0.692 | 0.868 | 5.83E-07 | 17.6 |
| RaGOO_A02_115088 | A02 | 24420543 | 2.08E-11 | 0.168 | 104 | 0.692 | 0.868 | 5.83E-07 | 17.6 |
| RaGOO_A02_115780 | A02 | 24516476 | 2.10E-11 | 0.159 | 104 | 0.692 | 0.868 | 5.83E-07 | 17.6 |
| RaGOO_A02_91630 | A02 | 18856877 | 2.13E-11 | 0.178 | 104 | 0.692 | 0.868 | 5.83E-07 | 17.6 |
| RaGOO_A02_91629 | A02 | 18856876 | 2.13E-11 | 0.178 | 104 | 0.692 | 0.868 | 5.83E-07 | 17.6 |
| RaGOO_A02_97235 | A02 | 20133305 | 2.21E-11 | 0.163 | 104 | 0.692 | 0.867 | 5.83E-07 | 17.5 |
| RaGOO_A02_118425 | A02 | 24909434 | 2.21E-11 | 0.163 | 104 | 0.692 | 0.867 | 5.83E-07 | 17.5 |
| RaGOO_A02_110827 | A02 | 22886219 | 2.23E-11 | 0.163 | 104 | 0.692 | 0.867 | 5.83E-07 | 17.5 |
| RaGOO_A02_97616 | A02 | 20185150 | 2.24E-11 | 0.188 | 104 | 0.692 | 0.867 | 5.83E-07 | 17.5 |
| RaGOO_A02_102558 | A02 | 21140775 | 2.31E-11 | 0.163 | 104 | 0.692 | 0.867 | 5.83E-07 | 17.5 |
| RaGOO_A02_108574 | A02 | 22422887 | 2.31E-11 | 0.163 | 104 | 0.692 | 0.867 | 5.83E-07 | 17.5 |
| RaGOO_A02_91699 | A02 | 18861506 | 2.31E-11 | 0.178 | 104 | 0.692 | 0.867 | 5.83E-07 | 17.5 |

| RaGOO_A02_99148 | A02 | 20485450 | 2.31E-11 | 0.159 | 104 | 0.692 | 0.867 | 5.83E-07 | 17.5 |
| --- | --- | --- | --- | --- | --- | --- | --- | --- | --- |
| RaGOO_A02_86676 | A02 | 17929523 | 2.32E-11 | 0.159 | 104 | 0.692 | 0.867 | 5.83E-07 | 17.5 |
| RaGOO_A02_79780 | A02 | 16873305 | 2.34E-11 | 0.163 | 104 | 0.692 | 0.867 | 5.83E-07 | 17.5 |
| RaGOO_A02_79782 | A02 | 16873319 | 2.34E-11 | 0.163 | 104 | 0.692 | 0.867 | 5.83E-07 | 17.5 |
| RaGOO_A02_73191 | A02 | 15740523 | 2.34E-11 | 0.163 | 104 | 0.692 | 0.867 | 5.83E-07 | 17.5 |
| RaGOO_A02_91331 | A02 | 18818375 | 2.35E-11 | 0.159 | 104 | 0.692 | 0.867 | 5.83E-07 | 17.5 |
| RaGOO_A02_91332 | A02 | 18818379 | 2.35E-11 | 0.159 | 104 | 0.692 | 0.867 | 5.83E-07 | 17.5 |
| RaGOO_A02_109712 | A02 | 22662283 | 2.42E-11 | 0.173 | 104 | 0.692 | 0.867 | 5.94E-07 | 17.5 |
| RaGOO_A02_73120 | A02 | 15729023 | 2.51E-11 | 0.163 | 104 | 0.692 | 0.866 | 5.96E-07 | 17.4 |
| RaGOO_A02_96053 | A02 | 19952925 | 2.51E-11 | 0.163 | 104 | 0.692 | 0.866 | 5.96E-07 | 17.4 |
| RaGOO_A02_104315 | A02 | 21564213 | 2.53E-11 | 0.163 | 104 | 0.692 | 0.866 | 5.96E-07 | 17.4 |
| RaGOO_A02_104314 | A02 | 21564193 | 2.53E-11 | 0.163 | 104 | 0.692 | 0.866 | 5.96E-07 | 17.4 |
| RaGOO_A02_112994 | A02 | 24016570 | 2.53E-11 | 0.163 | 104 | 0.692 | 0.866 | 5.96E-07 | 17.4 |
| RaGOO_A02_118222 | A02 | 24865702 | 2.54E-11 | 0.188 | 104 | 0.692 | 0.866 | 5.96E-07 | 17.4 |
| RaGOO_A02_102674 | A02 | 21150817 | 2.55E-11 | 0.168 | 104 | 0.692 | 0.866 | 5.96E-07 | 17.4 |
| RaGOO_A02_98989 | A02 | 20472714 | 2.61E-11 | 0.173 | 104 | 0.692 | 0.866 | 6.00E-07 | 17.4 |
| RaGOO_A02_82386 | A02 | 17225152 | 2.61E-11 | 0.159 | 104 | 0.692 | 0.866 | 6.00E-07 | 17.4 |
| RaGOO_A02_104312 | A02 | 21564059 | 2.64E-11 | 0.168 | 104 | 0.692 | 0.866 | 6.00E-07 | 17.4 |
| RaGOO_A02_91461 | A02 | 18845303 | 2.64E-11 | 0.168 | 104 | 0.692 | 0.866 | 6.00E-07 | 17.4 |
| RaGOO_A02_97095 | A02 | 20105154 | 2.77E-11 | 0.173 | 104 | 0.692 | 0.865 | 6.23E-07 | 17.3 |
| RaGOO_A02_119578 | A02 | 25152719 | 2.80E-11 | 0.163 | 104 | 0.692 | 0.865 | 6.24E-07 | 17.3 |
| RaGOO_A02_119576 | A02 | 25152658 | 2.86E-11 | 0.163 | 104 | 0.692 | 0.865 | 6.24E-07 | 17.3 |
| RaGOO_A02_91800 | A02 | 18873990 | 2.87E-11 | 0.168 | 104 | 0.692 | 0.865 | 6.24E-07 | 17.3 |
| RaGOO_A02_102677 | A02 | 21150929 | 2.88E-11 | 0.173 | 104 | 0.692 | 0.865 | 6.24E-07 | 17.3 |
| RaGOO_A02_91595 | A02 | 18855530 | 2.91E-11 | 0.188 | 104 | 0.692 | 0.865 | 6.24E-07 | 17.3 |
| RaGOO_A02_101384 | A02 | 20972382 | 2.92E-11 | 0.163 | 104 | 0.692 | 0.865 | 6.24E-07 | 17.3 |
| RaGOO_A02_101958 | A02 | 21046438 | 2.94E-11 | 0.183 | 104 | 0.692 | 0.865 | 6.24E-07 | 17.3 |
| RaGOO_A02_113899 | A02 | 24234828 | 2.95E-11 | 0.163 | 104 | 0.692 | 0.865 | 6.24E-07 | 17.3 |
| RaGOO_A02_113900 | A02 | 24234832 | 2.95E-11 | 0.163 | 104 | 0.692 | 0.865 | 6.24E-07 | 17.3 |
| RaGOO_A02_79781 | A02 | 16873318 | 2.97E-11 | 0.173 | 104 | 0.692 | 0.865 | 6.24E-07 | 17.3 |
| RaGOO_A02_117684 | A02 | 24797824 | 3.03E-11 | 0.173 | 104 | 0.692 | 0.864 | 6.32E-07 | 17.2 |
| RaGOO_A02_113880 | A02 | 24234206 | 3.04E-11 | 0.154 | 104 | 0.692 | 0.864 | 6.32E-07 | 17.2 |
| RaGOO_A02_91799 | A02 | 18873988 | 3.06E-11 | 0.163 | 104 | 0.692 | 0.864 | 6.32E-07 | 17.2 |
| RaGOO_A02_101912 | A02 | 21040471 | 3.09E-11 | 0.173 | 104 | 0.692 | 0.864 | 6.33E-07 | 17.2 |
| RaGOO_A02_91623 | A02 | 18856728 | 3.14E-11 | 0.178 | 104 | 0.692 | 0.864 | 6.40E-07 | 17.2 |
| RaGOO_A02_106286 | A02 | 21954092 | 3.16E-11 | 0.159 | 104 | 0.692 | 0.864 | 6.40E-07 | 17.2 |
| RaGOO_A02_91462 | A02 | 18845357 | 3.36E-11 | 0.163 | 104 | 0.692 | 0.863 | 6.72E-07 | 17.1 |
| RaGOO_A02_119575 | A02 | 25152612 | 3.36E-11 | 0.163 | 104 | 0.692 | 0.863 | 6.72E-07 | 17.1 |
| RaGOO_A02_108583 | A02 | 22423615 | 3.39E-11 | 0.168 | 104 | 0.692 | 0.863 | 6.72E-07 | 17.1 |
| RaGOO_A02_101215 | A02 | 20950680 | 3.41E-11 | 0.154 | 104 | 0.692 | 0.863 | 6.72E-07 | 17.1 |
| RaGOO_A02_73177 | A02 | 15739447 | 3.42E-11 | 0.159 | 104 | 0.692 | 0.863 | 6.72E-07 | 17.1 |
| RaGOO_A02_111231 | A02 | 22948223 | 3.58E-11 | 0.163 | 104 | 0.692 | 0.863 | 6.99E-07 | 17.1 |
| RaGOO_A02_91634 | A02 | 18857021 | 3.60E-11 | 0.192 | 104 | 0.692 | 0.863 | 6.99E-07 | 17.1 |
| RaGOO_A02_118230 | A02 | 24866177 | 3.66E-11 | 0.183 | 104 | 0.692 | 0.863 | 6.99E-07 | 17.1 |
| RaGOO_A02_108797 | A02 | 22463573 | 3.68E-11 | 0.168 | 104 | 0.692 | 0.863 | 6.99E-07 | 17.1 |
| RaGOO_A02_113742 | A02 | 24203448 | 3.68E-11 | 0.168 | 104 | 0.692 | 0.863 | 6.99E-07 | 17.1 |
| RaGOO_A02_100219 | A02 | 20723642 | 3.69E-11 | 0.168 | 104 | 0.692 | 0.863 | 6.99E-07 | 17.1 |
| RaGOO_A02_84484 | A02 | 17505538 | 3.79E-11 | 0.159 | 104 | 0.692 | 0.862 | 7.03E-07 | 17.0 |
| RaGOO_A02_118423 | A02 | 24909290 | 3.87E-11 | 0.173 | 104 | 0.692 | 0.862 | 7.03E-07 | 17.0 |
| RaGOO_A02_91606 | A02 | 18856290 | 3.90E-11 | 0.173 | 104 | 0.692 | 0.862 | 7.03E-07 | 17.0 |
| RaGOO_A02_91608 | A02 | 18856306 | 3.90E-11 | 0.173 | 104 | 0.692 | 0.862 | 7.03E-07 | 17.0 |
| RaGOO_A02_91609 | A02 | 18856325 | 3.90E-11 | 0.173 | 104 | 0.692 | 0.862 | 7.03E-07 | 17.0 |
| RaGOO_A02_82257 | A02 | 17218238 | 3.93E-11 | 0.178 | 104 | 0.692 | 0.862 | 7.03E-07 | 17.0 |
| RaGOO_A02_117725 | A02 | 24801883 | 3.93E-11 | 0.159 | 104 | 0.692 | 0.862 | 7.03E-07 | 17.0 |
| RaGOO_A02_118660 | A02 | 24966110 | 3.99E-11 | 0.144 | 104 | 0.692 | 0.862 | 7.03E-07 | 17.0 |
| RaGOO_A02_96544 | A02 | 20025014 | 4.01E-11 | 0.168 | 104 | 0.692 | 0.862 | 7.03E-07 | 17.0 |
| RaGOO_A02_102998 | A02 | 21218990 | 4.01E-11 | 0.168 | 104 | 0.692 | 0.862 | 7.03E-07 | 17.0 |
| RaGOO_A02_111073 | A02 | 22919790 | 4.01E-11 | 0.168 | 104 | 0.692 | 0.862 | 7.03E-07 | 17.0 |
| RaGOO_A02_116087 | A02 | 24548042 | 4.01E-11 | 0.168 | 104 | 0.692 | 0.862 | 7.03E-07 | 17.0 |
| RaGOO_A02_96543 | A02 | 20024975 | 4.01E-11 | 0.168 | 104 | 0.692 | 0.862 | 7.03E-07 | 17.0 |
| RaGOO_A02_102637 | A02 | 21147044 | 4.02E-11 | 0.178 | 104 | 0.692 | 0.862 | 7.03E-07 | 17.0 |
| RaGOO_A02_99639 | A02 | 20566513 | 4.06E-11 | 0.163 | 104 | 0.692 | 0.862 | 7.06E-07 | 17.0 |
| RaGOO_A02_118224 | A02 | 24865779 | 4.08E-11 | 0.173 | 104 | 0.692 | 0.862 | 7.06E-07 | 17.0 |
| RaGOO_A02_107233 | A02 | 22141917 | 4.11E-11 | 0.135 | 104 | 0.692 | 0.862 | 7.08E-07 | 17.0 |
| RaGOO_A02_116354 | A02 | 24571880 | 4.15E-11 | 0.168 | 104 | 0.692 | 0.861 | 7.11E-07 | 16.9 |
| RaGOO_A02_91460 | A02 | 18845119 | 4.20E-11 | 0.178 | 104 | 0.692 | 0.861 | 7.11E-07 | 16.9 |
| RaGOO_A02_107939 | A02 | 22201442 | 4.22E-11 | 0.120 | 104 | 0.692 | 0.861 | 7.11E-07 | 16.9 |
| RaGOO_A02_102569 | A02 | 21141235 | 4.24E-11 | 0.178 | 104 | 0.692 | 0.861 | 7.11E-07 | 16.9 |
| RaGOO_A02_102972 | A02 | 21216340 | 4.24E-11 | 0.178 | 104 | 0.692 | 0.861 | 7.11E-07 | 16.9 |
| RaGOO_A02_102955 | A02 | 21209184 | 4.26E-11 | 0.178 | 104 | 0.692 | 0.861 | 7.12E-07 | 16.9 |
| RaGOO_A02_102571 | A02 | 21141246 | 4.30E-11 | 0.168 | 104 | 0.692 | 0.861 | 7.14E-07 | 16.9 |
| RaGOO_A02_92297 | A02 | 18918755 | 4.39E-11 | 0.173 | 104 | 0.692 | 0.861 | 7.19E-07 | 16.9 |
| RaGOO_A02_91482 | A02 | 18846152 | 4.40E-11 | 0.168 | 104 | 0.692 | 0.861 | 7.19E-07 | 16.9 |
| RaGOO_A02_102331 | A02 | 21109762 | 4.40E-11 | 0.168 | 104 | 0.692 | 0.861 | 7.19E-07 | 16.9 |
| RaGOO_A02_99045 | A02 | 20476539 | 4.45E-11 | 0.163 | 104 | 0.692 | 0.861 | 7.20E-07 | 16.9 |
| RaGOO_A02_110125 | A02 | 22769558 | 4.46E-11 | 0.178 | 104 | 0.692 | 0.861 | 7.20E-07 | 16.9 |
| RaGOO_A02_98054 | A02 | 20256438 | 4.51E-11 | 0.163 | 104 | 0.692 | 0.861 | 7.20E-07 | 16.9 |
| RaGOO_A02_100462 | A02 | 20812810 | 4.53E-11 | 0.154 | 104 | 0.692 | 0.861 | 7.20E-07 | 16.9 |
| RaGOO_A02_117477 | A02 | 24745919 | 4.53E-11 | 0.163 | 104 | 0.692 | 0.861 | 7.20E-07 | 16.9 |
| RaGOO_A02_115843 | A02 | 24521311 | 4.54E-11 | 0.178 | 104 | 0.692 | 0.861 | 7.20E-07 | 16.9 |
| RaGOO_A02_102602 | A02 | 21143437 | 4.63E-11 | 0.163 | 104 | 0.692 | 0.860 | 7.26E-07 | 16.8 |
| RaGOO_A02_114204 | A02 | 24296812 | 4.68E-11 | 0.168 | 104 | 0.692 | 0.860 | 7.26E-07 | 16.8 |
| RaGOO_A02_91748 | A02 | 18865502 | 4.75E-11 | 0.173 | 104 | 0.692 | 0.860 | 7.26E-07 | 16.8 |
| RaGOO_A02_96589 | A02 | 20032673 | 4.78E-11 | 0.168 | 104 | 0.692 | 0.860 | 7.26E-07 | 16.8 |
| RaGOO_A02_99038 | A02 | 20476057 | 4.78E-11 | 0.168 | 104 | 0.692 | 0.860 | 7.26E-07 | 16.8 |
| RaGOO_A02_103037 | A02 | 21223038 | 4.78E-11 | 0.168 | 104 | 0.692 | 0.860 | 7.26E-07 | 16.8 |
| RaGOO_A02_89684 | A02 | 18546841 | 4.83E-11 | 0.163 | 104 | 0.692 | 0.860 | 7.26E-07 | 16.8 |
| RaGOO_A02_77714 | A02 | 16565144 | 4.84E-11 | 0.168 | 104 | 0.692 | 0.860 | 7.26E-07 | 16.8 |
| RaGOO_A02_77715 | A02 | 16565145 | 4.84E-11 | 0.168 | 104 | 0.692 | 0.860 | 7.26E-07 | 16.8 |
| RaGOO_A02_97245 | A02 | 20133996 | 4.89E-11 | 0.183 | 104 | 0.692 | 0.860 | 7.26E-07 | 16.8 |
| RaGOO_A02_90239 | A02 | 18616031 | 4.90E-11 | 0.168 | 104 | 0.692 | 0.860 | 7.26E-07 | 16.8 |

| RaGOO_A02_101383 | A02 | 20972184 | 4.92E-11 | 0.168 | 104 | 0.692 | 0.860 | 7.26E-07 | 16.8 |
| --- | --- | --- | --- | --- | --- | --- | --- | --- | --- |
| RaGOO_A02_99020 | A02 | 20475262 | 4.94E-11 | 0.173 | 104 | 0.692 | 0.860 | 7.26E-07 | 16.8 |
| RaGOO_A02_101055 | A02 | 20936116 | 4.96E-11 | 0.168 | 104 | 0.692 | 0.860 | 7.26E-07 | 16.8 |
| RaGOO_A02_103030 | A02 | 21222524 | 4.96E-11 | 0.168 | 104 | 0.692 | 0.860 | 7.26E-07 | 16.8 |
| RaGOO_A02_100283 | A02 | 20756678 | 4.98E-11 | 0.168 | 104 | 0.692 | 0.860 | 7.26E-07 | 16.8 |
| RaGOO_A02_91864 | A02 | 18878089 | 5.04E-11 | 0.192 | 104 | 0.692 | 0.860 | 7.26E-07 | 16.8 |
| RaGOO_A02_91965 | A02 | 18886508 | 5.09E-11 | 0.197 | 104 | 0.692 | 0.860 | 7.26E-07 | 16.8 |
| RaGOO_A02_102595 | A02 | 21142947 | 5.11E-11 | 0.173 | 104 | 0.692 | 0.860 | 7.26E-07 | 16.7 |
| RaGOO_A02_77694 | A02 | 16564154 | 5.12E-11 | 0.183 | 104 | 0.692 | 0.859 | 7.26E-07 | 16.7 |
| RaGOO_A02_111603 | A02 | 23167309 | 5.12E-11 | 0.183 | 104 | 0.692 | 0.859 | 7.26E-07 | 16.7 |
| RaGOO_A02_80220 | A02 | 16925210 | 5.15E-11 | 0.163 | 104 | 0.692 | 0.859 | 7.26E-07 | 16.7 |
| RaGOO_A02_100844 | A02 | 20892108 | 5.15E-11 | 0.163 | 104 | 0.692 | 0.859 | 7.26E-07 | 16.7 |
| RaGOO_A02_86928 | A02 | 17983168 | 5.16E-11 | 0.163 | 104 | 0.692 | 0.859 | 7.26E-07 | 16.7 |
| RaGOO_A02_86927 | A02 | 17983158 | 5.16E-11 | 0.163 | 104 | 0.692 | 0.859 | 7.26E-07 | 16.7 |
| RaGOO_A02_97273 | A02 | 20136839 | 5.16E-11 | 0.163 | 104 | 0.692 | 0.859 | 7.26E-07 | 16.7 |
| RaGOO_A02_102851 | A02 | 21170801 | 5.22E-11 | 0.159 | 104 | 0.692 | 0.859 | 7.26E-07 | 16.7 |
| RaGOO_A02_91904 | A02 | 18881921 | 5.27E-11 | 0.168 | 104 | 0.692 | 0.859 | 7.26E-07 | 16.7 |
| RaGOO_A02_115849 | A02 | 24521912 | 5.27E-11 | 0.168 | 104 | 0.692 | 0.859 | 7.26E-07 | 16.7 |
| RaGOO_A02_78928 | A02 | 16767793 | 5.27E-11 | 0.168 | 104 | 0.692 | 0.859 | 7.26E-07 | 16.7 |
| RaGOO_A02_99873 | A02 | 20627125 | 5.33E-11 | 0.178 | 104 | 0.692 | 0.859 | 7.26E-07 | 16.7 |
| RaGOO_A02_99874 | A02 | 20627127 | 5.33E-11 | 0.178 | 104 | 0.692 | 0.859 | 7.26E-07 | 16.7 |
| RaGOO_A02_113910 | A02 | 24235341 | 5.34E-11 | 0.163 | 104 | 0.692 | 0.859 | 7.26E-07 | 16.7 |
| RaGOO_A02_107942 | A02 | 22201541 | 5.35E-11 | 0.139 | 104 | 0.692 | 0.859 | 7.26E-07 | 16.7 |
| RaGOO_A02_114625 | A02 | 24365979 | 5.37E-11 | 0.173 | 104 | 0.692 | 0.859 | 7.26E-07 | 16.7 |
| RaGOO_A02_91488 | A02 | 18846438 | 5.48E-11 | 0.183 | 104 | 0.692 | 0.859 | 7.35E-07 | 16.7 |
| RaGOO_A02_91489 | A02 | 18846486 | 5.48E-11 | 0.183 | 104 | 0.692 | 0.859 | 7.35E-07 | 16.7 |
| RaGOO_A02_102572 | A02 | 21141354 | 5.56E-11 | 0.173 | 104 | 0.692 | 0.859 | 7.43E-07 | 16.7 |
| RaGOO_A02_91503 | A02 | 18848401 | 5.62E-11 | 0.178 | 104 | 0.692 | 0.859 | 7.46E-07 | 16.7 |
| RaGOO_A02_98005 | A02 | 20235034 | 5.63E-11 | 0.159 | 104 | 0.692 | 0.859 | 7.46E-07 | 16.7 |
| RaGOO_A02_96054 | A02 | 19952997 | 5.81E-11 | 0.163 | 104 | 0.692 | 0.858 | 7.66E-07 | 16.6 |
| RaGOO_A02_73168 | A02 | 15738544 | 5.89E-11 | 0.154 | 104 | 0.692 | 0.858 | 7.74E-07 | 16.6 |
| RaGOO_A02_80157 | A02 | 16918945 | 5.96E-11 | 0.159 | 104 | 0.692 | 0.858 | 7.78E-07 | 16.6 |
| RaGOO_A02_115837 | A02 | 24520793 | 5.98E-11 | 0.163 | 104 | 0.692 | 0.858 | 7.78E-07 | 16.6 |
| RaGOO_A02_101058 | A02 | 20936180 | 6.00E-11 | 0.168 | 104 | 0.692 | 0.858 | 7.78E-07 | 16.6 |
| RaGOO_A02_115089 | A02 | 24420547 | 6.02E-11 | 0.192 | 104 | 0.692 | 0.858 | 7.78E-07 | 16.6 |
| RaGOO_A02_91663 | A02 | 18859805 | 6.13E-11 | 0.178 | 104 | 0.692 | 0.858 | 7.90E-07 | 16.6 |
| RaGOO_A02_91749 | A02 | 18865538 | 6.26E-11 | 0.173 | 104 | 0.692 | 0.858 | 8.02E-07 | 16.6 |
| RaGOO_A02_91619 | A02 | 18856683 | 6.30E-11 | 0.178 | 104 | 0.692 | 0.858 | 8.02E-07 | 16.6 |
| RaGOO_A02_91622 | A02 | 18856711 | 6.30E-11 | 0.178 | 104 | 0.692 | 0.858 | 8.02E-07 | 16.6 |
| RaGOO_A02_91827 | A02 | 18875484 | 6.36E-11 | 0.178 | 104 | 0.692 | 0.857 | 8.03E-07 | 16.5 |
| RaGOO_A02_113904 | A02 | 24235011 | 6.36E-11 | 0.168 | 104 | 0.692 | 0.857 | 8.03E-07 | 16.5 |
| RaGOO_A02_89131 | A02 | 18423209 | 6.41E-11 | 0.163 | 104 | 0.692 | 0.857 | 8.07E-07 | 16.5 |
| RaGOO_A02_119586 | A02 | 25153168 | 6.47E-11 | 0.183 | 104 | 0.692 | 0.857 | 8.10E-07 | 16.5 |
| RaGOO_A02_110238 | A02 | 22784536 | 6.54E-11 | 0.159 | 104 | 0.692 | 0.857 | 8.16E-07 | 16.5 |
| RaGOO_A02_91914 | A02 | 18882501 | 6.60E-11 | 0.183 | 104 | 0.692 | 0.857 | 8.21E-07 | 16.5 |
| RaGOO_A02_105725 | A02 | 21880215 | 6.64E-11 | 0.173 | 104 | 0.692 | 0.857 | 8.22E-07 | 16.5 |
| RaGOO_A02_91661 | A02 | 18859758 | 6.68E-11 | 0.183 | 104 | 0.692 | 0.857 | 8.23E-07 | 16.5 |
| RaGOO_A02_78872 | A02 | 16760968 | 6.72E-11 | 0.163 | 104 | 0.692 | 0.857 | 8.23E-07 | 16.5 |
| RaGOO_A02_115327 | A02 | 24450170 | 6.75E-11 | 0.173 | 104 | 0.692 | 0.857 | 8.23E-07 | 16.5 |
| RaGOO_A02_101060 | A02 | 20936204 | 6.76E-11 | 0.168 | 104 | 0.692 | 0.857 | 8.23E-07 | 16.5 |
| RaGOO_A02_102575 | A02 | 21141549 | 6.77E-11 | 0.168 | 104 | 0.692 | 0.857 | 8.23E-07 | 16.5 |
| RaGOO_A02_102942 | A02 | 21207991 | 6.87E-11 | 0.183 | 104 | 0.692 | 0.857 | 8.29E-07 | 16.5 |
| RaGOO_A02_80167 | A02 | 16919450 | 6.93E-11 | 0.163 | 104 | 0.692 | 0.857 | 8.29E-07 | 16.5 |
| RaGOO_A02_91216 | A02 | 18765988 | 6.93E-11 | 0.163 | 104 | 0.692 | 0.857 | 8.29E-07 | 16.5 |
| RaGOO_A02_115800 | A02 | 24518270 | 6.93E-11 | 0.163 | 104 | 0.692 | 0.857 | 8.29E-07 | 16.5 |
| RaGOO_A02_100399 | A02 | 20795505 | 7.09E-11 | 0.159 | 104 | 0.692 | 0.856 | 8.46E-07 | 16.4 |
| RaGOO_A02_79726 | A02 | 16868296 | 7.22E-11 | 0.183 | 104 | 0.692 | 0.856 | 8.58E-07 | 16.4 |
| RaGOO_A02_105948 | A02 | 21910533 | 7.25E-11 | 0.163 | 104 | 0.692 | 0.856 | 8.59E-07 | 16.4 |
| RaGOO_A02_97239 | A02 | 20133461 | 7.32E-11 | 0.188 | 104 | 0.692 | 0.856 | 8.63E-07 | 16.4 |
| RaGOO_A02_77196 | A02 | 16449241 | 7.37E-11 | 0.168 | 104 | 0.692 | 0.856 | 8.63E-07 | 16.4 |
| RaGOO_A02_84486 | A02 | 17505643 | 7.37E-11 | 0.163 | 104 | 0.692 | 0.856 | 8.63E-07 | 16.4 |
| RaGOO_A02_83497 | A02 | 17346029 | 7.42E-11 | 0.159 | 104 | 0.692 | 0.856 | 8.65E-07 | 16.4 |
| RaGOO_A02_112860 | A02 | 23989087 | 7.44E-11 | 0.159 | 104 | 0.692 | 0.856 | 8.65E-07 | 16.4 |
| RaGOO_A02_114138 | A02 | 24270088 | 7.50E-11 | 0.154 | 104 | 0.692 | 0.856 | 8.68E-07 | 16.4 |
| RaGOO_A02_82380 | A02 | 17224794 | 7.57E-11 | 0.159 | 104 | 0.692 | 0.856 | 8.71E-07 | 16.4 |
| RaGOO_A02_89434 | A02 | 18512631 | 7.62E-11 | 0.173 | 104 | 0.692 | 0.856 | 8.71E-07 | 16.4 |
| RaGOO_A02_91664 | A02 | 18859815 | 7.62E-11 | 0.178 | 104 | 0.692 | 0.856 | 8.71E-07 | 16.4 |
| RaGOO_A02_114490 | A02 | 24331841 | 7.63E-11 | 0.163 | 104 | 0.692 | 0.856 | 8.71E-07 | 16.4 |
| RaGOO_A02_102950 | A02 | 21208853 | 7.68E-11 | 0.178 | 104 | 0.692 | 0.856 | 8.74E-07 | 16.4 |
| RaGOO_A02_80263 | A02 | 16929442 | 7.96E-11 | 0.159 | 104 | 0.692 | 0.855 | 9.02E-07 | 16.3 |
| RaGOO_A02_118229 | A02 | 24866015 | 8.04E-11 | 0.178 | 104 | 0.692 | 0.855 | 9.09E-07 | 16.3 |
| RaGOO_A02_109704 | A02 | 22661591 | 8.10E-11 | 0.168 | 104 | 0.692 | 0.855 | 9.12E-07 | 16.3 |
| RaGOO_A02_114637 | A02 | 24366274 | 8.28E-11 | 0.173 | 104 | 0.692 | 0.855 | 9.24E-07 | 16.3 |
| RaGOO_A02_105245 | A02 | 21798690 | 8.29E-11 | 0.168 | 104 | 0.692 | 0.855 | 9.24E-07 | 16.3 |
| RaGOO_A02_101214 | A02 | 20950595 | 8.38E-11 | 0.154 | 104 | 0.692 | 0.855 | 9.24E-07 | 16.3 |
| RaGOO_A02_118197 | A02 | 24863120 | 8.46E-11 | 0.178 | 104 | 0.692 | 0.855 | 9.24E-07 | 16.3 |
| RaGOO_A02_96619 | A02 | 20036243 | 8.46E-11 | 0.173 | 104 | 0.692 | 0.855 | 9.24E-07 | 16.3 |
| RaGOO_A02_71744 | A02 | 15428829 | 8.47E-11 | 0.144 | 104 | 0.692 | 0.855 | 9.24E-07 | 16.3 |
| RaGOO_A02_71745 | A02 | 15428833 | 8.47E-11 | 0.144 | 104 | 0.692 | 0.855 | 9.24E-07 | 16.3 |
| RaGOO_A02_110823 | A02 | 22885159 | 8.48E-11 | 0.183 | 104 | 0.692 | 0.855 | 9.24E-07 | 16.3 |
| RaGOO_A02_100826 | A02 | 20890795 | 8.50E-11 | 0.173 | 104 | 0.692 | 0.855 | 9.24E-07 | 16.3 |
| RaGOO_A02_105626 | A02 | 21863772 | 8.52E-11 | 0.173 | 104 | 0.692 | 0.855 | 9.24E-07 | 16.3 |
| RaGOO_A02_73056 | A02 | 15722978 | 8.69E-11 | 0.159 | 104 | 0.692 | 0.855 | 9.35E-07 | 16.3 |
| RaGOO_A02_73511 | A02 | 15792967 | 8.71E-11 | 0.163 | 104 | 0.692 | 0.855 | 9.35E-07 | 16.3 |
| RaGOO_A02_103436 | A02 | 21342141 | 8.71E-11 | 0.197 | 104 | 0.692 | 0.855 | 9.35E-07 | 16.3 |
| RaGOO_A02_72816 | A02 | 15654718 | 8.77E-11 | 0.163 | 104 | 0.692 | 0.854 | 9.35E-07 | 16.2 |
| RaGOO_A02_97317 | A02 | 20142717 | 8.80E-11 | 0.159 | 104 | 0.692 | 0.854 | 9.35E-07 | 16.2 |
| RaGOO_A02_83433 | A02 | 17330760 | 8.84E-11 | 0.159 | 104 | 0.692 | 0.854 | 9.35E-07 | 16.2 |
| RaGOO_A02_110237 | A02 | 22784518 | 8.89E-11 | 0.159 | 104 | 0.692 | 0.854 | 9.35E-07 | 16.2 |
| RaGOO_A02_111228 | A02 | 22948051 | 8.90E-11 | 0.173 | 104 | 0.692 | 0.854 | 9.35E-07 | 16.2 |
| RaGOO_A02_91486 | A02 | 18846417 | 8.90E-11 | 0.183 | 104 | 0.692 | 0.854 | 9.35E-07 | 16.2 |

| RaGOO_A02_83499 | A02 | 17346047 | 8.91E-11 | 0.159 | 104 | 0.692 | 0.854 | 9.35E-07 | 16.2 |
| --- | --- | --- | --- | --- | --- | --- | --- | --- | --- |
| RaGOO_A02_100827 | A02 | 20890819 | 8.97E-11 | 0.178 | 104 | 0.692 | 0.854 | 9.38E-07 | 16.2 |
| RaGOO_A02_113115 | A02 | 24036496 | 9.08E-11 | 0.173 | 104 | 0.692 | 0.854 | 9.46E-07 | 16.2 |
| RaGOO_A02_102874 | A02 | 21173799 | 9.15E-11 | 0.168 | 104 | 0.692 | 0.854 | 9.51E-07 | 16.2 |
| RaGOO_A02_104319 | A02 | 21564336 | 9.33E-11 | 0.154 | 104 | 0.692 | 0.854 | 9.66E-07 | 16.2 |
| RaGOO_A02_73277 | A02 | 15757830 | 9.44E-11 | 0.159 | 104 | 0.692 | 0.854 | 9.74E-07 | 16.2 |
| RaGOO_A02_104311 | A02 | 21563996 | 9.49E-11 | 0.173 | 104 | 0.692 | 0.854 | 9.74E-07 | 16.2 |
| RaGOO_A02_100695 | A02 | 20856117 | 9.50E-11 | 0.178 | 104 | 0.692 | 0.854 | 9.74E-07 | 16.2 |
| RaGOO_A02_97021 | A02 | 20092620 | 9.66E-11 | 0.159 | 104 | 0.692 | 0.854 | 9.88E-07 | 16.2 |
| RaGOO_A02_113911 | A02 | 24235415 | 9.77E-11 | 0.178 | 104 | 0.692 | 0.853 | 9.94E-07 | 16.1 |
| RaGOO_A02_89563 | A02 | 18530591 | 9.79E-11 | 0.178 | 104 | 0.692 | 0.853 | 9.94E-07 | 16.1 |
| RaGOO_A02_105792 | A02 | 21886320 | 9.86E-11 | 0.168 | 104 | 0.692 | 0.853 | 9.95E-07 | 16.1 |
| RaGOO_A02_99042 | A02 | 20476267 | 9.93E-11 | 0.183 | 104 | 0.692 | 0.853 | 9.95E-07 | 16.1 |
| RaGOO_A02_102959 | A02 | 21209388 | 9.93E-11 | 0.183 | 104 | 0.692 | 0.853 | 9.95E-07 | 16.1 |
| RaGOO_A02_102970 | A02 | 21216256 | 9.94E-11 | 0.188 | 104 | 0.692 | 0.853 | 9.95E-07 | 16.1 |
| RaGOO_A02_104146 | A02 | 21540640 | 9.97E-11 | 0.183 | 104 | 0.692 | 0.853 | 9.95E-07 | 16.1 |
| RaGOO_A02_113114 | A02 | 24036429 | 9.98E-11 | 0.173 | 104 | 0.692 | 0.853 | 9.95E-07 | 16.1 |
| RaGOO_A02_114496 | A02 | 24332364 | 1.01E-10 | 0.188 | 104 | 0.692 | 0.853 | 9.97E-07 | 16.1 |
| RaGOO_A02_100823 | A02 | 20890697 | 1.01E-10 | 0.168 | 104 | 0.692 | 0.853 | 9.97E-07 | 16.1 |
| RaGOO_A02_108256 | A02 | 22378823 | 1.02E-10 | 0.168 | 104 | 0.692 | 0.853 | 1.00E-06 | 16.1 |
| RaGOO_A02_114622 | A02 | 24365712 | 1.03E-10 | 0.168 | 104 | 0.692 | 0.853 | 1.02E-06 | 16.1 |
| RaGOO_A02_98028 | A02 | 20253865 | 1.04E-10 | 0.163 | 104 | 0.692 | 0.853 | 1.02E-06 | 16.1 |
| RaGOO_A02_89273 | A02 | 18467297 | 1.05E-10 | 0.154 | 104 | 0.692 | 0.853 | 1.03E-06 | 16.1 |
| RaGOO_A02_99822 | A02 | 20611669 | 1.07E-10 | 0.163 | 104 | 0.692 | 0.853 | 1.04E-06 | 16.1 |
| RaGOO_A02_106288 | A02 | 21954112 | 1.07E-10 | 0.159 | 104 | 0.692 | 0.853 | 1.05E-06 | 16.1 |
| RaGOO_A02_91648 | A02 | 18858562 | 1.09E-10 | 0.188 | 104 | 0.692 | 0.852 | 1.06E-06 | 16.0 |
| RaGOO_A02_85146 | A02 | 17633258 | 1.12E-10 | 0.183 | 104 | 0.692 | 0.852 | 1.08E-06 | 16.0 |
| RaGOO_A02_91912 | A02 | 18882436 | 1.14E-10 | 0.178 | 104 | 0.692 | 0.852 | 1.09E-06 | 16.0 |
| RaGOO_A02_111601 | A02 | 23167168 | 1.14E-10 | 0.178 | 104 | 0.692 | 0.852 | 1.09E-06 | 16.0 |
| RaGOO_A02_91656 | A02 | 18859529 | 1.15E-10 | 0.192 | 104 | 0.692 | 0.852 | 1.10E-06 | 16.0 |
| RaGOO_A02_100686 | A02 | 20855098 | 1.15E-10 | 0.183 | 104 | 0.692 | 0.852 | 1.10E-06 | 16.0 |
| RaGOO_A02_102661 | A02 | 21148933 | 1.16E-10 | 0.173 | 104 | 0.692 | 0.852 | 1.11E-06 | 16.0 |
| RaGOO_A02_110784 | A02 | 22880700 | 1.18E-10 | 0.154 | 104 | 0.692 | 0.852 | 1.11E-06 | 16.0 |
| RaGOO_A02_90335 | A02 | 18639092 | 1.20E-10 | 0.173 | 104 | 0.692 | 0.852 | 1.13E-06 | 16.0 |
| RaGOO_A02_110222 | A02 | 22781894 | 1.21E-10 | 0.163 | 104 | 0.692 | 0.852 | 1.14E-06 | 16.0 |
| RaGOO_A02_97314 | A02 | 20142535 | 1.21E-10 | 0.163 | 104 | 0.692 | 0.851 | 1.14E-06 | 15.9 |
| RaGOO_A02_102671 | A02 | 21150631 | 1.22E-10 | 0.159 | 104 | 0.692 | 0.851 | 1.15E-06 | 15.9 |
| RaGOO_A02_109670 | A02 | 22658268 | 1.25E-10 | 0.163 | 104 | 0.692 | 0.851 | 1.17E-06 | 15.9 |
| RaGOO_A02_115836 | A02 | 24520779 | 1.26E-10 | 0.163 | 104 | 0.692 | 0.851 | 1.17E-06 | 15.9 |
| RaGOO_A02_91597 | A02 | 18855649 | 1.26E-10 | 0.183 | 104 | 0.692 | 0.851 | 1.17E-06 | 15.9 |
| RaGOO_A02_91598 | A02 | 18855650 | 1.26E-10 | 0.183 | 104 | 0.692 | 0.851 | 1.17E-06 | 15.9 |
| RaGOO_A02_91612 | A02 | 18856471 | 1.27E-10 | 0.163 | 104 | 0.692 | 0.851 | 1.17E-06 | 15.9 |
| RaGOO_A02_91911 | A02 | 18882411 | 1.27E-10 | 0.192 | 104 | 0.692 | 0.851 | 1.17E-06 | 15.9 |
| RaGOO_A02_111604 | A02 | 23167334 | 1.27E-10 | 0.183 | 104 | 0.692 | 0.851 | 1.17E-06 | 15.9 |
| RaGOO_A02_80138 | A02 | 16917456 | 1.28E-10 | 0.159 | 104 | 0.692 | 0.851 | 1.17E-06 | 15.9 |
| RaGOO_A02_96458 | A02 | 20006752 | 1.29E-10 | 0.173 | 104 | 0.692 | 0.851 | 1.17E-06 | 15.9 |
| RaGOO_A02_100101 | A02 | 20691763 | 1.30E-10 | 0.159 | 104 | 0.692 | 0.851 | 1.17E-06 | 15.9 |
| RaGOO_A02_91601 | A02 | 18855867 | 1.31E-10 | 0.163 | 104 | 0.692 | 0.851 | 1.18E-06 | 15.9 |
| RaGOO_A02_91647 | A02 | 18858555 | 1.33E-10 | 0.188 | 104 | 0.692 | 0.851 | 1.20E-06 | 15.9 |
| RaGOO_A02_91964 | A02 | 18886503 | 1.35E-10 | 0.202 | 104 | 0.692 | 0.851 | 1.21E-06 | 15.9 |
| RaGOO_A02_100825 | A02 | 20890773 | 1.36E-10 | 0.163 | 104 | 0.692 | 0.850 | 1.22E-06 | 15.8 |
| RaGOO_A02_99625 | A02 | 20562270 | 1.37E-10 | 0.149 | 104 | 0.692 | 0.850 | 1.22E-06 | 15.8 |
| RaGOO_A02_97618 | A02 | 20185254 | 1.37E-10 | 0.188 | 104 | 0.692 | 0.850 | 1.22E-06 | 15.8 |
| RaGOO_A02_117731 | A02 | 24802307 | 1.39E-10 | 0.168 | 104 | 0.692 | 0.850 | 1.23E-06 | 15.8 |
| RaGOO_A02_91820 | A02 | 18875288 | 1.39E-10 | 0.168 | 104 | 0.692 | 0.850 | 1.23E-06 | 15.8 |
| RaGOO_A02_84695 | A02 | 17531067 | 1.45E-10 | 0.163 | 104 | 0.692 | 0.850 | 1.28E-06 | 15.8 |
| RaGOO_A02_82274 | A02 | 17218812 | 1.45E-10 | 0.163 | 104 | 0.692 | 0.850 | 1.28E-06 | 15.8 |
| RaGOO_A02_76676 | A02 | 16327287 | 1.46E-10 | 0.125 | 104 | 0.692 | 0.850 | 1.28E-06 | 15.8 |
| RaGOO_A02_97629 | A02 | 20186160 | 1.47E-10 | 0.115 | 104 | 0.692 | 0.850 | 1.28E-06 | 15.8 |
| RaGOO_A02_92017 | A02 | 18892583 | 1.47E-10 | 0.163 | 104 | 0.692 | 0.850 | 1.28E-06 | 15.8 |
| RaGOO_A02_92018 | A02 | 18892587 | 1.47E-10 | 0.163 | 104 | 0.692 | 0.850 | 1.28E-06 | 15.8 |
| RaGOO_A02_73192 | A02 | 15740561 | 1.48E-10 | 0.159 | 104 | 0.692 | 0.850 | 1.28E-06 | 15.8 |
| RaGOO_A02_73193 | A02 | 15740563 | 1.48E-10 | 0.159 | 104 | 0.692 | 0.850 | 1.28E-06 | 15.8 |
| RaGOO_A02_79875 | A02 | 16884251 | 1.48E-10 | 0.149 | 104 | 0.692 | 0.850 | 1.28E-06 | 15.8 |
| RaGOO_A02_73908 | A02 | 15927226 | 1.49E-10 | 0.168 | 104 | 0.692 | 0.850 | 1.28E-06 | 15.8 |
| RaGOO_A02_110214 | A02 | 22780599 | 1.49E-10 | 0.154 | 104 | 0.692 | 0.850 | 1.28E-06 | 15.8 |
| RaGOO_A02_97635 | A02 | 20186540 | 1.50E-10 | 0.183 | 104 | 0.692 | 0.850 | 1.28E-06 | 15.8 |
| RaGOO_A02_97634 | A02 | 20186536 | 1.50E-10 | 0.183 | 104 | 0.692 | 0.850 | 1.28E-06 | 15.8 |
| RaGOO_A02_72946 | A02 | 15706488 | 1.51E-10 | 0.178 | 104 | 0.692 | 0.849 | 1.28E-06 | 15.7 |
| RaGOO_A02_80161 | A02 | 16919174 | 1.51E-10 | 0.163 | 104 | 0.692 | 0.849 | 1.28E-06 | 15.7 |
| RaGOO_A02_101548 | A02 | 20991223 | 1.51E-10 | 0.178 | 104 | 0.692 | 0.849 | 1.28E-06 | 15.7 |
| RaGOO_A02_119579 | A02 | 25152852 | 1.51E-10 | 0.168 | 104 | 0.692 | 0.849 | 1.28E-06 | 15.7 |
| RaGOO_A02_91646 | A02 | 18858317 | 1.51E-10 | 0.197 | 104 | 0.692 | 0.849 | 1.28E-06 | 15.7 |
| RaGOO_A02_91320 | A02 | 18817597 | 1.53E-10 | 0.159 | 104 | 0.692 | 0.849 | 1.28E-06 | 15.7 |
| RaGOO_A02_91321 | A02 | 18817598 | 1.53E-10 | 0.159 | 104 | 0.692 | 0.849 | 1.28E-06 | 15.7 |
| RaGOO_A02_91819 | A02 | 18875222 | 1.55E-10 | 0.173 | 104 | 0.692 | 0.849 | 1.30E-06 | 15.7 |
| RaGOO_A02_105861 | A02 | 21896851 | 1.57E-10 | 0.163 | 104 | 0.692 | 0.849 | 1.31E-06 | 15.7 |
| RaGOO_A02_98939 | A02 | 20467063 | 1.63E-10 | 0.168 | 104 | 0.692 | 0.849 | 1.36E-06 | 15.7 |
| RaGOO_A02_73149 | A02 | 15737217 | 1.64E-10 | 0.159 | 104 | 0.692 | 0.849 | 1.36E-06 | 15.7 |
| RaGOO_A02_105443 | A02 | 21823875 | 1.66E-10 | 0.163 | 104 | 0.692 | 0.849 | 1.37E-06 | 15.7 |
| RaGOO_A02_98982 | A02 | 20471876 | 1.70E-10 | 0.149 | 104 | 0.692 | 0.848 | 1.39E-06 | 15.6 |
| RaGOO_A02_98983 | A02 | 20471877 | 1.70E-10 | 0.149 | 104 | 0.692 | 0.848 | 1.39E-06 | 15.6 |
| RaGOO_A02_98984 | A02 | 20471885 | 1.70E-10 | 0.149 | 104 | 0.692 | 0.848 | 1.39E-06 | 15.6 |
| RaGOO_A02_114150 | A02 | 24271983 | 1.76E-10 | 0.163 | 104 | 0.692 | 0.848 | 1.43E-06 | 15.6 |
| RaGOO_A02_106129 | A02 | 21928883 | 1.76E-10 | 0.163 | 104 | 0.692 | 0.848 | 1.43E-06 | 15.6 |
| RaGOO_A02_119580 | A02 | 25152853 | 1.76E-10 | 0.163 | 104 | 0.692 | 0.848 | 1.43E-06 | 15.6 |
| RaGOO_A02_119581 | A02 | 25152869 | 1.76E-10 | 0.163 | 104 | 0.692 | 0.848 | 1.43E-06 | 15.6 |
| RaGOO_A02_114636 | A02 | 24366242 | 1.77E-10 | 0.173 | 104 | 0.692 | 0.848 | 1.43E-06 | 15.6 |
| RaGOO_A02_82378 | A02 | 17224755 | 1.78E-10 | 0.159 | 104 | 0.692 | 0.848 | 1.43E-06 | 15.6 |
| RaGOO_A02_97625 | A02 | 20185613 | 1.78E-10 | 0.125 | 104 | 0.692 | 0.848 | 1.43E-06 | 15.6 |
| RaGOO_A02_91463 | A02 | 18845373 | 1.78E-10 | 0.168 | 104 | 0.692 | 0.848 | 1.43E-06 | 15.6 |

| RaGOO_A02_100221 | A02 | 20724699 | 1.79E-10 | 0.173 | 104 | 0.692 | 0.848 | 1.43E-06 | 15.6 |
| --- | --- | --- | --- | --- | --- | --- | --- | --- | --- |
| RaGOO_A02_75352 | A02 | 16179089 | 1.79E-10 | 0.168 | 104 | 0.692 | 0.848 | 1.43E-06 | 15.6 |
| RaGOO_A02_91600 | A02 | 18855821 | 1.80E-10 | 0.173 | 104 | 0.692 | 0.848 | 1.44E-06 | 15.6 |
| RaGOO_A02_108573 | A02 | 22422876 | 1.81E-10 | 0.159 | 104 | 0.692 | 0.848 | 1.44E-06 | 15.6 |
| RaGOO_A02_97963 | A02 | 20231360 | 1.82E-10 | 0.163 | 104 | 0.692 | 0.848 | 1.44E-06 | 15.6 |
| RaGOO_A02_118237 | A02 | 24866846 | 1.84E-10 | 0.192 | 104 | 0.692 | 0.848 | 1.46E-06 | 15.6 |
| RaGOO_A02_100603 | A02 | 20843204 | 1.86E-10 | 0.159 | 104 | 0.692 | 0.848 | 1.47E-06 | 15.6 |
| RaGOO_A02_91909 | A02 | 18882082 | 1.88E-10 | 0.188 | 104 | 0.692 | 0.847 | 1.48E-06 | 15.5 |
| RaGOO_A02_91720 | A02 | 18863201 | 1.89E-10 | 0.183 | 104 | 0.692 | 0.847 | 1.48E-06 | 15.5 |
| RaGOO_A02_112993 | A02 | 24016548 | 1.89E-10 | 0.159 | 104 | 0.692 | 0.847 | 1.48E-06 | 15.5 |
| RaGOO_A02_90711 | A02 | 18688469 | 1.90E-10 | 0.163 | 104 | 0.692 | 0.847 | 1.48E-06 | 15.5 |
| RaGOO_A02_101061 | A02 | 20936287 | 1.91E-10 | 0.173 | 104 | 0.692 | 0.847 | 1.49E-06 | 15.5 |
| RaGOO_A02_119585 | A02 | 25153128 | 1.91E-10 | 0.173 | 104 | 0.692 | 0.847 | 1.49E-06 | 15.5 |
| RaGOO_A02_98048 | A02 | 20256062 | 1.92E-10 | 0.173 | 104 | 0.692 | 0.847 | 1.49E-06 | 15.5 |
| RaGOO_A02_84694 | A02 | 17531047 | 1.93E-10 | 0.163 | 104 | 0.692 | 0.847 | 1.49E-06 | 15.5 |
| RaGOO_A02_115500 | A02 | 24474348 | 1.94E-10 | 0.159 | 104 | 0.692 | 0.847 | 1.49E-06 | 15.5 |
| RaGOO_A02_80230 | A02 | 16926044 | 1.94E-10 | 0.163 | 104 | 0.692 | 0.847 | 1.49E-06 | 15.5 |
| RaGOO_A02_103226 | A02 | 21295114 | 1.95E-10 | 0.178 | 104 | 0.692 | 0.847 | 1.50E-06 | 15.5 |
| RaGOO_A02_91907 | A02 | 18881965 | 1.95E-10 | 0.188 | 104 | 0.692 | 0.847 | 1.50E-06 | 15.5 |
| RaGOO_A02_99862 | A02 | 20625811 | 1.99E-10 | 0.163 | 104 | 0.692 | 0.847 | 1.52E-06 | 15.5 |
| RaGOO_A02_91490 | A02 | 18846610 | 2.00E-10 | 0.173 | 104 | 0.692 | 0.847 | 1.52E-06 | 15.5 |
| RaGOO_A02_118951 | A02 | 24997747 | 2.01E-10 | 0.139 | 104 | 0.692 | 0.847 | 1.52E-06 | 15.5 |
| RaGOO_A02_100195 | A02 | 20714251 | 2.01E-10 | 0.159 | 104 | 0.692 | 0.847 | 1.52E-06 | 15.5 |
| RaGOO_A02_114998 | A02 | 24410069 | 2.04E-10 | 0.183 | 104 | 0.692 | 0.847 | 1.54E-06 | 15.5 |
| RaGOO_A02_98168 | A02 | 20278362 | 2.05E-10 | 0.163 | 104 | 0.692 | 0.847 | 1.55E-06 | 15.5 |
| RaGOO_A02_105923 | A02 | 21907980 | 2.07E-10 | 0.154 | 104 | 0.692 | 0.847 | 1.55E-06 | 15.5 |
| RaGOO_A02_103885 | A02 | 21500947 | 2.07E-10 | 0.163 | 104 | 0.692 | 0.847 | 1.55E-06 | 15.5 |
| RaGOO_A02_100284 | A02 | 20756701 | 2.08E-10 | 0.159 | 104 | 0.692 | 0.847 | 1.56E-06 | 15.5 |
| RaGOO_A02_118223 | A02 | 24865732 | 2.09E-10 | 0.183 | 104 | 0.692 | 0.846 | 1.56E-06 | 15.4 |
| RaGOO_A02_114193 | A02 | 24294446 | 2.09E-10 | 0.173 | 104 | 0.692 | 0.846 | 1.56E-06 | 15.4 |
| RaGOO_A02_85147 | A02 | 17633287 | 2.11E-10 | 0.168 | 104 | 0.692 | 0.846 | 1.57E-06 | 15.4 |
| RaGOO_A02_117717 | A02 | 24801254 | 2.11E-10 | 0.173 | 104 | 0.692 | 0.846 | 1.57E-06 | 15.4 |
| RaGOO_A02_83434 | A02 | 17330764 | 2.12E-10 | 0.163 | 104 | 0.692 | 0.846 | 1.57E-06 | 15.4 |
| RaGOO_A02_91481 | A02 | 18846073 | 2.12E-10 | 0.163 | 104 | 0.692 | 0.846 | 1.57E-06 | 15.4 |
| RaGOO_A02_91967 | A02 | 18886544 | 2.13E-10 | 0.192 | 104 | 0.692 | 0.846 | 1.57E-06 | 15.4 |
| RaGOO_A02_107644 | A02 | 22175166 | 2.13E-10 | 0.120 | 104 | 0.692 | 0.846 | 1.57E-06 | 15.4 |
| RaGOO_A02_82373 | A02 | 17224340 | 2.14E-10 | 0.159 | 104 | 0.692 | 0.846 | 1.57E-06 | 15.4 |
| RaGOO_A02_73321 | A02 | 15764053 | 2.15E-10 | 0.159 | 104 | 0.692 | 0.846 | 1.57E-06 | 15.4 |
| RaGOO_A02_110761 | A02 | 22878675 | 2.15E-10 | 0.125 | 104 | 0.692 | 0.846 | 1.57E-06 | 15.4 |
| RaGOO_A02_110764 | A02 | 22878710 | 2.15E-10 | 0.125 | 104 | 0.692 | 0.846 | 1.57E-06 | 15.4 |
| RaGOO_A02_73220 | A02 | 15743296 | 2.17E-10 | 0.168 | 104 | 0.692 | 0.846 | 1.57E-06 | 15.4 |
| RaGOO_A02_108796 | A02 | 22463557 | 2.21E-10 | 0.163 | 104 | 0.692 | 0.846 | 1.60E-06 | 15.4 |
| RaGOO_A02_120066 | A02 | 25235436 | 2.22E-10 | 0.173 | 104 | 0.692 | 0.846 | 1.60E-06 | 15.4 |
| RaGOO_A02_120067 | A02 | 25235467 | 2.22E-10 | 0.173 | 104 | 0.692 | 0.846 | 1.60E-06 | 15.4 |
| RaGOO_A02_79779 | A02 | 16873146 | 2.25E-10 | 0.178 | 104 | 0.692 | 0.846 | 1.62E-06 | 15.4 |
| RaGOO_A02_114639 | A02 | 24366349 | 2.26E-10 | 0.168 | 104 | 0.692 | 0.846 | 1.62E-06 | 15.4 |
| RaGOO_A02_104317 | A02 | 21564313 | 2.28E-10 | 0.163 | 104 | 0.692 | 0.846 | 1.63E-06 | 15.4 |
| RaGOO_A02_75264 | A02 | 16171946 | 2.28E-10 | 0.163 | 104 | 0.692 | 0.846 | 1.63E-06 | 15.4 |
| RaGOO_A02_103544 | A02 | 21377922 | 2.28E-10 | 0.163 | 104 | 0.692 | 0.846 | 1.63E-06 | 15.4 |
| RaGOO_A02_110630 | A02 | 22842116 | 2.29E-10 | 0.159 | 104 | 0.692 | 0.846 | 1.63E-06 | 15.4 |
| RaGOO_A02_118219 | A02 | 24865547 | 2.30E-10 | 0.163 | 104 | 0.692 | 0.846 | 1.64E-06 | 15.4 |
| RaGOO_A02_90339 | A02 | 18639285 | 2.32E-10 | 0.178 | 104 | 0.692 | 0.846 | 1.64E-06 | 15.4 |
| RaGOO_A02_108464 | A02 | 22395364 | 2.32E-10 | 0.163 | 104 | 0.692 | 0.846 | 1.64E-06 | 15.4 |
| RaGOO_A02_117718 | A02 | 24801255 | 2.33E-10 | 0.168 | 104 | 0.692 | 0.846 | 1.64E-06 | 15.3 |
| RaGOO_A02_82387 | A02 | 17225186 | 2.34E-10 | 0.159 | 104 | 0.692 | 0.845 | 1.65E-06 | 15.3 |
| RaGOO_A02_116305 | A02 | 24568689 | 2.35E-10 | 0.183 | 104 | 0.692 | 0.845 | 1.65E-06 | 15.3 |
| RaGOO_A02_100354 | A02 | 20783040 | 2.35E-10 | 0.163 | 104 | 0.692 | 0.845 | 1.65E-06 | 15.3 |
| RaGOO_A02_89560 | A02 | 18529278 | 2.36E-10 | 0.173 | 104 | 0.692 | 0.845 | 1.65E-06 | 15.3 |
| RaGOO_A02_79614 | A02 | 16857222 | 2.37E-10 | 0.159 | 104 | 0.692 | 0.845 | 1.66E-06 | 15.3 |
| RaGOO_A02_125271 | A02 | 26081033 | 2.39E-10 | 0.159 | 104 | 0.692 | 0.845 | 1.66E-06 | 15.3 |
| RaGOO_A02_109652 | A02 | 22656840 | 2.39E-10 | 0.159 | 104 | 0.692 | 0.845 | 1.66E-06 | 15.3 |
| RaGOO_A02_109506 | A02 | 22634860 | 2.39E-10 | 0.168 | 104 | 0.692 | 0.845 | 1.66E-06 | 15.3 |
| RaGOO_A02_103036 | A02 | 21222919 | 2.39E-10 | 0.159 | 104 | 0.692 | 0.845 | 1.66E-06 | 15.3 |
| RaGOO_A02_96588 | A02 | 20032519 | 2.40E-10 | 0.173 | 104 | 0.692 | 0.845 | 1.66E-06 | 15.3 |
| RaGOO_A02_85812 | A02 | 17735829 | 2.41E-10 | 0.178 | 104 | 0.692 | 0.845 | 1.66E-06 | 15.3 |
| RaGOO_A02_102278 | A02 | 21103743 | 2.41E-10 | 0.159 | 104 | 0.692 | 0.845 | 1.66E-06 | 15.3 |
| RaGOO_A02_100783 | A02 | 20869343 | 2.44E-10 | 0.168 | 104 | 0.692 | 0.845 | 1.67E-06 | 15.3 |
| RaGOO_A02_88543 | A02 | 18261195 | 2.45E-10 | 0.168 | 104 | 0.692 | 0.845 | 1.68E-06 | 15.3 |
| RaGOO_A02_110236 | A02 | 22784444 | 2.45E-10 | 0.173 | 104 | 0.692 | 0.845 | 1.68E-06 | 15.3 |
| RaGOO_A02_118220 | A02 | 24865600 | 2.48E-10 | 0.163 | 104 | 0.692 | 0.845 | 1.69E-06 | 15.3 |
| RaGOO_A02_77348 | A02 | 16486045 | 2.49E-10 | 0.168 | 104 | 0.692 | 0.845 | 1.69E-06 | 15.3 |
| RaGOO_A02_109998 | A02 | 22751690 | 2.50E-10 | 0.163 | 104 | 0.692 | 0.845 | 1.70E-06 | 15.3 |
| RaGOO_A02_89130 | A02 | 18423187 | 2.52E-10 | 0.173 | 104 | 0.692 | 0.845 | 1.71E-06 | 15.3 |
| RaGOO_A02_116069 | A02 | 24547080 | 2.53E-10 | 0.159 | 104 | 0.692 | 0.845 | 1.71E-06 | 15.3 |
| RaGOO_A02_104316 | A02 | 21564234 | 2.54E-10 | 0.159 | 104 | 0.692 | 0.845 | 1.71E-06 | 15.3 |
| RaGOO_A02_73055 | A02 | 15722940 | 2.55E-10 | 0.159 | 104 | 0.692 | 0.845 | 1.71E-06 | 15.3 |
| RaGOO_A02_107800 | A02 | 22194258 | 2.55E-10 | 0.144 | 104 | 0.692 | 0.845 | 1.71E-06 | 15.3 |
| RaGOO_A02_118231 | A02 | 24866210 | 2.55E-10 | 0.178 | 104 | 0.692 | 0.845 | 1.71E-06 | 15.3 |
| RaGOO_A02_96620 | A02 | 20036252 | 2.57E-10 | 0.178 | 104 | 0.692 | 0.845 | 1.72E-06 | 15.3 |
| RaGOO_A02_100344 | A02 | 20780673 | 2.60E-10 | 0.168 | 104 | 0.692 | 0.845 | 1.73E-06 | 15.3 |
| RaGOO_A02_82255 | A02 | 17218189 | 2.60E-10 | 0.163 | 104 | 0.692 | 0.844 | 1.74E-06 | 15.2 |
| RaGOO_A02_113274 | A02 | 24060368 | 2.62E-10 | 0.163 | 104 | 0.692 | 0.844 | 1.74E-06 | 15.2 |
| RaGOO_A02_86922 | A02 | 17982862 | 2.62E-10 | 0.168 | 104 | 0.692 | 0.844 | 1.74E-06 | 15.2 |
| RaGOO_A02_75977 | A02 | 16243536 | 2.62E-10 | 0.188 | 104 | 0.692 | 0.844 | 1.74E-06 | 15.2 |
| RaGOO_A02_111488 | A02 | 23135657 | 2.63E-10 | 0.163 | 104 | 0.692 | 0.844 | 1.74E-06 | 15.2 |
| RaGOO_A02_80098 | A02 | 16913417 | 2.63E-10 | 0.168 | 104 | 0.692 | 0.844 | 1.74E-06 | 15.2 |
| RaGOO_A02_100887 | A02 | 20895483 | 2.64E-10 | 0.159 | 104 | 0.692 | 0.844 | 1.74E-06 | 15.2 |
| RaGOO_A02_84692 | A02 | 17531026 | 2.66E-10 | 0.173 | 104 | 0.692 | 0.844 | 1.74E-06 | 15.2 |
| RaGOO_A02_105606 | A02 | 21853040 | 2.66E-10 | 0.159 | 104 | 0.692 | 0.844 | 1.74E-06 | 15.2 |
| RaGOO_A02_75266 | A02 | 16171974 | 2.69E-10 | 0.163 | 104 | 0.692 | 0.844 | 1.75E-06 | 15.2 |
| RaGOO_A02_75267 | A02 | 16171978 | 2.69E-10 | 0.163 | 104 | 0.692 | 0.844 | 1.75E-06 | 15.2 |

| RaGOO_A02_91825 | A02 | 18875452 | 2.69E-10 | 0.173 | 104 | 0.692 | 0.844 | 1.75E-06 | 15.2 |
| --- | --- | --- | --- | --- | --- | --- | --- | --- | --- |
| RaGOO_A02_91824 | A02 | 18875451 | 2.69E-10 | 0.173 | 104 | 0.692 | 0.844 | 1.75E-06 | 15.2 |
| RaGOO_A02_97307 | A02 | 20141944 | 2.70E-10 | 0.163 | 104 | 0.692 | 0.844 | 1.75E-06 | 15.2 |
| RaGOO_A02_117733 | A02 | 24802359 | 2.70E-10 | 0.159 | 104 | 0.692 | 0.844 | 1.75E-06 | 15.2 |
| RaGOO_A02_115693 | A02 | 24501600 | 2.70E-10 | 0.168 | 104 | 0.692 | 0.844 | 1.75E-06 | 15.2 |
| RaGOO_A02_82325 | A02 | 17221529 | 2.72E-10 | 0.159 | 104 | 0.692 | 0.844 | 1.75E-06 | 15.2 |
| RaGOO_A02_100870 | A02 | 20893948 | 2.72E-10 | 0.173 | 104 | 0.692 | 0.844 | 1.75E-06 | 15.2 |
| RaGOO_A02_90240 | A02 | 18616068 | 2.73E-10 | 0.178 | 104 | 0.692 | 0.844 | 1.76E-06 | 15.2 |
| RaGOO_A02_101382 | A02 | 20972095 | 2.74E-10 | 0.149 | 104 | 0.692 | 0.844 | 1.76E-06 | 15.2 |
| RaGOO_A02_105537 | A02 | 21841559 | 2.75E-10 | 0.168 | 104 | 0.692 | 0.844 | 1.76E-06 | 15.2 |
| RaGOO_A02_89371 | A02 | 18493276 | 2.76E-10 | 0.173 | 104 | 0.692 | 0.844 | 1.76E-06 | 15.2 |
| RaGOO_A02_108381 | A02 | 22389395 | 2.78E-10 | 0.168 | 104 | 0.692 | 0.844 | 1.76E-06 | 15.2 |
| RaGOO_A02_97623 | A02 | 20185437 | 2.78E-10 | 0.183 | 104 | 0.692 | 0.844 | 1.76E-06 | 15.2 |
| RaGOO_A02_110901 | A02 | 22902021 | 2.78E-10 | 0.163 | 104 | 0.692 | 0.844 | 1.76E-06 | 15.2 |
| RaGOO_A02_87929 | A02 | 18155986 | 2.79E-10 | 0.163 | 104 | 0.692 | 0.844 | 1.76E-06 | 15.2 |
| RaGOO_A02_111051 | A02 | 22918194 | 2.79E-10 | 0.163 | 104 | 0.692 | 0.844 | 1.76E-06 | 15.2 |
| RaGOO_A02_109650 | A02 | 22656425 | 2.81E-10 | 0.168 | 104 | 0.692 | 0.844 | 1.77E-06 | 15.2 |
| RaGOO_A02_73208 | A02 | 15741717 | 2.82E-10 | 0.154 | 104 | 0.692 | 0.844 | 1.77E-06 | 15.2 |
| RaGOO_A02_85774 | A02 | 17731591 | 2.82E-10 | 0.159 | 104 | 0.692 | 0.844 | 1.77E-06 | 15.2 |
| RaGOO_A02_115694 | A02 | 24501706 | 2.83E-10 | 0.163 | 104 | 0.692 | 0.844 | 1.78E-06 | 15.2 |
| RaGOO_A02_97624 | A02 | 20185452 | 2.86E-10 | 0.173 | 104 | 0.692 | 0.844 | 1.78E-06 | 15.2 |
| RaGOO_A02_97240 | A02 | 20133605 | 2.86E-10 | 0.188 | 104 | 0.692 | 0.844 | 1.78E-06 | 15.2 |
| RaGOO_A02_98868 | A02 | 20454456 | 2.86E-10 | 0.178 | 104 | 0.692 | 0.844 | 1.78E-06 | 15.2 |
| RaGOO_A02_91983 | A02 | 18888545 | 2.86E-10 | 0.154 | 104 | 0.692 | 0.844 | 1.78E-06 | 15.2 |
| RaGOO_A02_110123 | A02 | 22769368 | 2.88E-10 | 0.173 | 104 | 0.692 | 0.844 | 1.79E-06 | 15.2 |
| RaGOO_A02_91631 | A02 | 18856959 | 2.92E-10 | 0.183 | 104 | 0.692 | 0.843 | 1.81E-06 | 15.1 |
| RaGOO_A02_89762 | A02 | 18556173 | 2.95E-10 | 0.163 | 104 | 0.692 | 0.843 | 1.83E-06 | 15.1 |
| RaGOO_A02_83498 | A02 | 17346041 | 2.96E-10 | 0.154 | 104 | 0.692 | 0.843 | 1.83E-06 | 15.1 |
| RaGOO_A02_91984 | A02 | 18888822 | 2.97E-10 | 0.168 | 104 | 0.692 | 0.843 | 1.83E-06 | 15.1 |
| RaGOO_A02_116355 | A02 | 24571952 | 2.98E-10 | 0.168 | 104 | 0.692 | 0.843 | 1.83E-06 | 15.1 |
| RaGOO_A02_91980 | A02 | 18888172 | 2.99E-10 | 0.168 | 104 | 0.692 | 0.843 | 1.83E-06 | 15.1 |
| RaGOO_A02_105414 | A02 | 21818756 | 2.99E-10 | 0.168 | 104 | 0.692 | 0.843 | 1.83E-06 | 15.1 |
| RaGOO_A02_88141 | A02 | 18197390 | 3.01E-10 | 0.163 | 104 | 0.692 | 0.843 | 1.84E-06 | 15.1 |
| RaGOO_A02_114499 | A02 | 24332392 | 3.02E-10 | 0.183 | 104 | 0.692 | 0.843 | 1.84E-06 | 15.1 |
| RaGOO_A02_108925 | A02 | 22544388 | 3.03E-10 | 0.159 | 104 | 0.692 | 0.843 | 1.85E-06 | 15.1 |
| RaGOO_A02_79778 | A02 | 16873128 | 3.09E-10 | 0.168 | 104 | 0.692 | 0.843 | 1.88E-06 | 15.1 |
| RaGOO_A02_106467 | A02 | 21973491 | 3.11E-10 | 0.149 | 104 | 0.692 | 0.843 | 1.89E-06 | 15.1 |
| RaGOO_A02_113902 | A02 | 24234970 | 3.12E-10 | 0.163 | 104 | 0.692 | 0.843 | 1.89E-06 | 15.1 |
| RaGOO_A02_113903 | A02 | 24234981 | 3.12E-10 | 0.163 | 104 | 0.692 | 0.843 | 1.89E-06 | 15.1 |
| RaGOO_A02_97804 | A02 | 20217149 | 3.12E-10 | 0.163 | 104 | 0.692 | 0.843 | 1.89E-06 | 15.1 |
| RaGOO_A02_105860 | A02 | 21896840 | 3.14E-10 | 0.168 | 104 | 0.692 | 0.843 | 1.89E-06 | 15.1 |
| RaGOO_A02_103222 | A02 | 21294821 | 3.16E-10 | 0.163 | 104 | 0.692 | 0.843 | 1.90E-06 | 15.1 |
| RaGOO_A02_103224 | A02 | 21294928 | 3.16E-10 | 0.163 | 104 | 0.692 | 0.843 | 1.90E-06 | 15.1 |
| RaGOO_A02_98004 | A02 | 20235030 | 3.18E-10 | 0.163 | 104 | 0.692 | 0.843 | 1.90E-06 | 15.1 |
| RaGOO_A02_110904 | A02 | 22902273 | 3.18E-10 | 0.163 | 104 | 0.692 | 0.843 | 1.90E-06 | 15.1 |
| RaGOO_A02_109090 | A02 | 22572403 | 3.22E-10 | 0.163 | 104 | 0.692 | 0.843 | 1.90E-06 | 15.1 |
| RaGOO_A02_109091 | A02 | 22572404 | 3.22E-10 | 0.163 | 104 | 0.692 | 0.843 | 1.90E-06 | 15.1 |
| RaGOO_A02_99097 | A02 | 20480549 | 3.23E-10 | 0.159 | 104 | 0.692 | 0.843 | 1.90E-06 | 15.1 |
| RaGOO_A02_114623 | A02 | 24365810 | 3.23E-10 | 0.159 | 104 | 0.692 | 0.843 | 1.90E-06 | 15.1 |
| RaGOO_A02_118424 | A02 | 24909365 | 3.23E-10 | 0.159 | 104 | 0.692 | 0.843 | 1.90E-06 | 15.1 |
| RaGOO_A02_109932 | A02 | 22688178 | 3.23E-10 | 0.159 | 104 | 0.692 | 0.843 | 1.90E-06 | 15.1 |
| RaGOO_A02_99626 | A02 | 20562453 | 3.24E-10 | 0.159 | 104 | 0.692 | 0.843 | 1.90E-06 | 15.1 |
| RaGOO_A02_100767 | A02 | 20868024 | 3.24E-10 | 0.159 | 104 | 0.692 | 0.843 | 1.90E-06 | 15.1 |
| RaGOO_A02_100768 | A02 | 20868027 | 3.24E-10 | 0.159 | 104 | 0.692 | 0.843 | 1.90E-06 | 15.1 |
| RaGOO_A02_100133 | A02 | 20698296 | 3.24E-10 | 0.168 | 104 | 0.692 | 0.843 | 1.90E-06 | 15.1 |
| RaGOO_A02_109933 | A02 | 22688218 | 3.25E-10 | 0.159 | 104 | 0.692 | 0.842 | 1.91E-06 | 15.0 |
| RaGOO_A02_107894 | A02 | 22200144 | 3.27E-10 | 0.115 | 104 | 0.692 | 0.842 | 1.92E-06 | 15.0 |
| RaGOO_A02_103839 | A02 | 21496911 | 3.30E-10 | 0.163 | 104 | 0.692 | 0.842 | 1.93E-06 | 15.0 |
| RaGOO_A02_103840 | A02 | 21496934 | 3.30E-10 | 0.163 | 104 | 0.692 | 0.842 | 1.93E-06 | 15.0 |
| RaGOO_A02_91906 | A02 | 18881958 | 3.32E-10 | 0.178 | 104 | 0.692 | 0.842 | 1.94E-06 | 15.0 |
| RaGOO_A02_97914 | A02 | 20227489 | 3.33E-10 | 0.168 | 104 | 0.692 | 0.842 | 1.94E-06 | 15.0 |
| RaGOO_A02_73190 | A02 | 15740516 | 3.34E-10 | 0.159 | 104 | 0.692 | 0.842 | 1.94E-06 | 15.0 |
| RaGOO_A02_107941 | A02 | 22201537 | 3.35E-10 | 0.144 | 104 | 0.692 | 0.842 | 1.94E-06 | 15.0 |
| RaGOO_A02_103850 | A02 | 21497372 | 3.35E-10 | 0.159 | 104 | 0.692 | 0.842 | 1.94E-06 | 15.0 |
| RaGOO_A02_115801 | A02 | 24518336 | 3.36E-10 | 0.168 | 104 | 0.692 | 0.842 | 1.94E-06 | 15.0 |
| RaGOO_A02_110906 | A02 | 22902544 | 3.36E-10 | 0.178 | 104 | 0.692 | 0.842 | 1.94E-06 | 15.0 |
| RaGOO_A02_105402 | A02 | 21817038 | 3.37E-10 | 0.154 | 104 | 0.692 | 0.842 | 1.94E-06 | 15.0 |
| RaGOO_A02_102675 | A02 | 21150823 | 3.38E-10 | 0.163 | 104 | 0.692 | 0.842 | 1.94E-06 | 15.0 |
| RaGOO_A02_105934 | A02 | 21909107 | 3.40E-10 | 0.168 | 104 | 0.692 | 0.842 | 1.95E-06 | 15.0 |
| RaGOO_A02_87433 | A02 | 18075077 | 3.41E-10 | 0.130 | 104 | 0.692 | 0.842 | 1.95E-06 | 15.0 |
| RaGOO_A02_91818 | A02 | 18875195 | 3.41E-10 | 0.178 | 104 | 0.692 | 0.842 | 1.95E-06 | 15.0 |
| RaGOO_A02_100131 | A02 | 20698190 | 3.43E-10 | 0.168 | 104 | 0.692 | 0.842 | 1.95E-06 | 15.0 |
| RaGOO_A02_98055 | A02 | 20256496 | 3.49E-10 | 0.163 | 104 | 0.692 | 0.842 | 1.98E-06 | 15.0 |
| RaGOO_A02_98956 | A02 | 20469626 | 3.50E-10 | 0.168 | 104 | 0.692 | 0.842 | 1.99E-06 | 15.0 |
| RaGOO_A02_92425 | A02 | 18934155 | 3.52E-10 | 0.163 | 104 | 0.692 | 0.842 | 1.99E-06 | 15.0 |
| RaGOO_A02_101840 | A02 | 21032644 | 3.52E-10 | 0.183 | 104 | 0.692 | 0.842 | 1.99E-06 | 15.0 |
| RaGOO_A02_115808 | A02 | 24518913 | 3.53E-10 | 0.159 | 104 | 0.692 | 0.842 | 1.99E-06 | 15.0 |
| RaGOO_A02_102670 | A02 | 21150583 | 3.55E-10 | 0.168 | 104 | 0.692 | 0.842 | 1.99E-06 | 15.0 |
| RaGOO_A02_77057 | A02 | 16423858 | 3.55E-10 | 0.159 | 104 | 0.692 | 0.842 | 1.99E-06 | 15.0 |
| RaGOO_A02_77056 | A02 | 16423851 | 3.55E-10 | 0.159 | 104 | 0.692 | 0.842 | 1.99E-06 | 15.0 |
| RaGOO_A02_118636 | A02 | 24956886 | 3.58E-10 | 0.163 | 104 | 0.692 | 0.842 | 2.01E-06 | 15.0 |
| RaGOO_A02_88144 | A02 | 18197708 | 3.58E-10 | 0.178 | 104 | 0.692 | 0.842 | 2.01E-06 | 15.0 |
| RaGOO_A02_105247 | A02 | 21798842 | 3.59E-10 | 0.163 | 104 | 0.692 | 0.842 | 2.01E-06 | 15.0 |
| RaGOO_A02_82253 | A02 | 17218125 | 3.60E-10 | 0.159 | 104 | 0.692 | 0.842 | 2.01E-06 | 15.0 |
| RaGOO_A02_99095 | A02 | 20480468 | 3.62E-10 | 0.163 | 104 | 0.692 | 0.842 | 2.01E-06 | 14.9 |
| RaGOO_A02_102819 | A02 | 21168078 | 3.63E-10 | 0.159 | 104 | 0.692 | 0.842 | 2.01E-06 | 14.9 |
| RaGOO_A02_91515 | A02 | 18849998 | 3.63E-10 | 0.173 | 104 | 0.692 | 0.841 | 2.01E-06 | 14.9 |
| RaGOO_A02_111052 | A02 | 22918198 | 3.64E-10 | 0.159 | 104 | 0.692 | 0.841 | 2.01E-06 | 14.9 |
| RaGOO_A02_89523 | A02 | 18523931 | 3.64E-10 | 0.163 | 104 | 0.692 | 0.841 | 2.01E-06 | 14.9 |
| RaGOO_A02_117715 | A02 | 24801045 | 3.66E-10 | 0.154 | 104 | 0.692 | 0.841 | 2.02E-06 | 14.9 |
| RaGOO_A02_115090 | A02 | 24420575 | 3.66E-10 | 0.192 | 104 | 0.692 | 0.841 | 2.02E-06 | 14.9 |

| RaGOO_A02_114642 | A02 | 24366409 | 3.66E-10 | 0.163 | 104 | 0.692 | 0.841 | 2.02E-06 | 14.9 |
| --- | --- | --- | --- | --- | --- | --- | --- | --- | --- |
| RaGOO_A02_106469 | A02 | 21973590 | 3.67E-10 | 0.154 | 104 | 0.692 | 0.841 | 2.02E-06 | 14.9 |
| RaGOO_A02_110767 | A02 | 22878905 | 3.71E-10 | 0.178 | 104 | 0.692 | 0.841 | 2.03E-06 | 14.9 |
| RaGOO_A02_115692 | A02 | 24501467 | 3.72E-10 | 0.163 | 104 | 0.692 | 0.841 | 2.03E-06 | 14.9 |
| RaGOO_A02_112518 | A02 | 23949179 | 3.75E-10 | 0.159 | 104 | 0.692 | 0.841 | 2.05E-06 | 14.9 |
| RaGOO_A02_105598 | A02 | 21851741 | 3.75E-10 | 0.173 | 104 | 0.692 | 0.841 | 2.05E-06 | 14.9 |
| RaGOO_A02_97357 | A02 | 20152471 | 3.76E-10 | 0.173 | 104 | 0.692 | 0.841 | 2.05E-06 | 14.9 |
| RaGOO_A02_82300 | A02 | 17220032 | 3.77E-10 | 0.163 | 104 | 0.692 | 0.841 | 2.05E-06 | 14.9 |
| RaGOO_A02_83620 | A02 | 17373905 | 3.78E-10 | 0.168 | 104 | 0.692 | 0.841 | 2.05E-06 | 14.9 |
| RaGOO_A02_101213 | A02 | 20950557 | 3.81E-10 | 0.154 | 104 | 0.692 | 0.841 | 2.06E-06 | 14.9 |
| RaGOO_A02_91701 | A02 | 18861625 | 3.83E-10 | 0.197 | 104 | 0.692 | 0.841 | 2.07E-06 | 14.9 |
| RaGOO_A02_91969 | A02 | 18886772 | 3.84E-10 | 0.183 | 104 | 0.692 | 0.841 | 2.07E-06 | 14.9 |
| RaGOO_A02_115092 | A02 | 24420716 | 3.84E-10 | 0.168 | 104 | 0.692 | 0.841 | 2.07E-06 | 14.9 |
| RaGOO_A02_114618 | A02 | 24365515 | 3.85E-10 | 0.163 | 104 | 0.692 | 0.841 | 2.07E-06 | 14.9 |
| RaGOO_A02_78878 | A02 | 16761313 | 3.86E-10 | 0.168 | 104 | 0.692 | 0.841 | 2.07E-06 | 14.9 |
| RaGOO_A02_115827 | A02 | 24519856 | 3.88E-10 | 0.154 | 104 | 0.692 | 0.841 | 2.08E-06 | 14.9 |
| RaGOO_A02_92081 | A02 | 18898786 | 3.89E-10 | 0.159 | 104 | 0.692 | 0.841 | 2.08E-06 | 14.9 |
| RaGOO_A02_108592 | A02 | 22424395 | 3.90E-10 | 0.168 | 104 | 0.692 | 0.841 | 2.08E-06 | 14.9 |
| RaGOO_A02_90647 | A02 | 18682919 | 3.92E-10 | 0.168 | 104 | 0.692 | 0.841 | 2.09E-06 | 14.9 |
| RaGOO_A02_117726 | A02 | 24802142 | 3.95E-10 | 0.163 | 104 | 0.692 | 0.841 | 2.09E-06 | 14.9 |
| RaGOO_A02_102576 | A02 | 21141605 | 3.95E-10 | 0.183 | 104 | 0.692 | 0.841 | 2.09E-06 | 14.9 |
| RaGOO_A02_89128 | A02 | 18423081 | 3.95E-10 | 0.159 | 104 | 0.692 | 0.841 | 2.09E-06 | 14.9 |
| RaGOO_A02_104174 | A02 | 21546427 | 3.97E-10 | 0.159 | 104 | 0.692 | 0.841 | 2.10E-06 | 14.9 |
| RaGOO_A02_102973 | A02 | 21216455 | 4.02E-10 | 0.188 | 104 | 0.692 | 0.841 | 2.12E-06 | 14.9 |
| RaGOO_A02_79012 | A02 | 16781063 | 4.04E-10 | 0.163 | 104 | 0.692 | 0.841 | 2.13E-06 | 14.9 |
| RaGOO_A02_83619 | A02 | 17373892 | 4.05E-10 | 0.168 | 104 | 0.692 | 0.841 | 2.13E-06 | 14.8 |
| RaGOO_A02_91693 | A02 | 18861211 | 4.10E-10 | 0.173 | 104 | 0.692 | 0.840 | 2.15E-06 | 14.8 |
| RaGOO_A02_90599 | A02 | 18677106 | 4.11E-10 | 0.154 | 104 | 0.692 | 0.840 | 2.15E-06 | 14.8 |
| RaGOO_A02_111626 | A02 | 23170267 | 4.11E-10 | 0.163 | 104 | 0.692 | 0.840 | 2.15E-06 | 14.8 |
| RaGOO_A02_107937 | A02 | 22201418 | 4.12E-10 | 0.130 | 104 | 0.692 | 0.840 | 2.15E-06 | 14.8 |
| RaGOO_A02_118217 | A02 | 24865220 | 4.21E-10 | 0.163 | 104 | 0.692 | 0.840 | 2.20E-06 | 14.8 |
| RaGOO_A02_108557 | A02 | 22421666 | 4.24E-10 | 0.159 | 104 | 0.692 | 0.840 | 2.20E-06 | 14.8 |
| RaGOO_A02_82228 | A02 | 17216809 | 4.24E-10 | 0.159 | 104 | 0.692 | 0.840 | 2.20E-06 | 14.8 |
| RaGOO_A02_82229 | A02 | 17216810 | 4.24E-10 | 0.159 | 104 | 0.692 | 0.840 | 2.20E-06 | 14.8 |
| RaGOO_A02_97641 | A02 | 20186788 | 4.29E-10 | 0.183 | 104 | 0.692 | 0.840 | 2.22E-06 | 14.8 |
| RaGOO_A02_115832 | A02 | 24520182 | 4.30E-10 | 0.159 | 104 | 0.692 | 0.840 | 2.22E-06 | 14.8 |
| RaGOO_A02_109092 | A02 | 22572421 | 4.31E-10 | 0.173 | 104 | 0.692 | 0.840 | 2.23E-06 | 14.8 |
| RaGOO_A02_89942 | A02 | 18581057 | 4.35E-10 | 0.163 | 104 | 0.692 | 0.840 | 2.25E-06 | 14.8 |
| RaGOO_A02_72940 | A02 | 15705386 | 4.36E-10 | 0.159 | 104 | 0.692 | 0.840 | 2.25E-06 | 14.8 |
| RaGOO_A02_97313 | A02 | 20142435 | 4.36E-10 | 0.154 | 104 | 0.692 | 0.840 | 2.25E-06 | 14.8 |
| RaGOO_A02_84709 | A02 | 17531860 | 4.41E-10 | 0.163 | 104 | 0.692 | 0.840 | 2.26E-06 | 14.8 |
| RaGOO_A02_116321 | A02 | 24569885 | 4.41E-10 | 0.163 | 104 | 0.692 | 0.840 | 2.26E-06 | 14.8 |
| RaGOO_A02_99094 | A02 | 20480430 | 4.45E-10 | 0.163 | 104 | 0.692 | 0.840 | 2.28E-06 | 14.8 |
| RaGOO_A02_100390 | A02 | 20792522 | 4.48E-10 | 0.163 | 104 | 0.692 | 0.840 | 2.29E-06 | 14.8 |
| RaGOO_A02_100697 | A02 | 20856259 | 4.50E-10 | 0.183 | 104 | 0.692 | 0.840 | 2.29E-06 | 14.8 |
| RaGOO_A02_100888 | A02 | 20895547 | 4.54E-10 | 0.159 | 104 | 0.692 | 0.839 | 2.31E-06 | 14.7 |
| RaGOO_A02_92428 | A02 | 18934321 | 4.56E-10 | 0.168 | 104 | 0.692 | 0.839 | 2.32E-06 | 14.7 |
| RaGOO_A02_105793 | A02 | 21886353 | 4.57E-10 | 0.159 | 104 | 0.692 | 0.839 | 2.32E-06 | 14.7 |
| RaGOO_A02_83496 | A02 | 17346005 | 4.60E-10 | 0.149 | 104 | 0.692 | 0.839 | 2.33E-06 | 14.7 |
| RaGOO_A02_112964 | A02 | 23998113 | 4.64E-10 | 0.178 | 104 | 0.692 | 0.839 | 2.33E-06 | 14.7 |
| RaGOO_A02_112963 | A02 | 23998092 | 4.64E-10 | 0.178 | 104 | 0.692 | 0.839 | 2.33E-06 | 14.7 |
| RaGOO_A02_102557 | A02 | 21140718 | 4.64E-10 | 0.173 | 104 | 0.692 | 0.839 | 2.33E-06 | 14.7 |
| RaGOO_A02_108613 | A02 | 22425429 | 4.65E-10 | 0.173 | 104 | 0.692 | 0.839 | 2.33E-06 | 14.7 |
| RaGOO_A02_107219 | A02 | 22141422 | 4.65E-10 | 0.125 | 104 | 0.692 | 0.839 | 2.33E-06 | 14.7 |
| RaGOO_A02_115796 | A02 | 24517950 | 4.66E-10 | 0.163 | 104 | 0.692 | 0.839 | 2.33E-06 | 14.7 |
| RaGOO_A02_89352 | A02 | 18484610 | 4.67E-10 | 0.178 | 104 | 0.692 | 0.839 | 2.33E-06 | 14.7 |
| RaGOO_A02_91092 | A02 | 18757560 | 4.68E-10 | 0.168 | 104 | 0.692 | 0.839 | 2.33E-06 | 14.7 |
| RaGOO_A02_108576 | A02 | 22423215 | 4.68E-10 | 0.168 | 104 | 0.692 | 0.839 | 2.33E-06 | 14.7 |
| RaGOO_A02_100882 | A02 | 20895180 | 4.68E-10 | 0.173 | 104 | 0.692 | 0.839 | 2.33E-06 | 14.7 |
| RaGOO_A02_87800 | A02 | 18143671 | 4.69E-10 | 0.188 | 104 | 0.692 | 0.839 | 2.33E-06 | 14.7 |
| RaGOO_A02_105538 | A02 | 21841651 | 4.69E-10 | 0.163 | 104 | 0.692 | 0.839 | 2.33E-06 | 14.7 |
| RaGOO_A02_77695 | A02 | 16564179 | 4.73E-10 | 0.163 | 104 | 0.692 | 0.839 | 2.34E-06 | 14.7 |
| RaGOO_A02_92082 | A02 | 18898793 | 4.74E-10 | 0.168 | 104 | 0.692 | 0.839 | 2.34E-06 | 14.7 |
| RaGOO_A02_80218 | A02 | 16924989 | 4.74E-10 | 0.163 | 104 | 0.692 | 0.839 | 2.34E-06 | 14.7 |
| RaGOO_A02_99650 | A02 | 20570931 | 4.77E-10 | 0.168 | 104 | 0.692 | 0.839 | 2.35E-06 | 14.7 |
| RaGOO_A02_117671 | A02 | 24796417 | 4.79E-10 | 0.202 | 104 | 0.692 | 0.839 | 2.36E-06 | 14.7 |
| RaGOO_A02_117686 | A02 | 24798211 | 4.86E-10 | 0.159 | 104 | 0.692 | 0.839 | 2.39E-06 | 14.7 |
| RaGOO_A02_94533 | A02 | 19289923 | 4.87E-10 | 0.120 | 104 | 0.692 | 0.839 | 2.39E-06 | 14.7 |
| RaGOO_A02_91611 | A02 | 18856466 | 4.87E-10 | 0.168 | 104 | 0.692 | 0.839 | 2.39E-06 | 14.7 |
| RaGOO_A02_82366 | A02 | 17224133 | 4.93E-10 | 0.163 | 104 | 0.692 | 0.839 | 2.41E-06 | 14.7 |
| RaGOO_A02_101874 | A02 | 21036996 | 4.94E-10 | 0.163 | 104 | 0.692 | 0.839 | 2.41E-06 | 14.7 |
| RaGOO_A02_100782 | A02 | 20869287 | 4.94E-10 | 0.163 | 104 | 0.692 | 0.839 | 2.41E-06 | 14.7 |
| RaGOO_A02_88685 | A02 | 18273570 | 4.95E-10 | 0.159 | 104 | 0.692 | 0.839 | 2.41E-06 | 14.7 |
| RaGOO_A02_90340 | A02 | 18639376 | 4.96E-10 | 0.168 | 104 | 0.692 | 0.839 | 2.42E-06 | 14.7 |
| RaGOO_A02_112132 | A02 | 23886154 | 4.97E-10 | 0.154 | 104 | 0.692 | 0.839 | 2.42E-06 | 14.7 |
| RaGOO_A02_80164 | A02 | 16919232 | 4.98E-10 | 0.173 | 104 | 0.692 | 0.839 | 2.42E-06 | 14.7 |
| RaGOO_A02_116063 | A02 | 24546868 | 4.98E-10 | 0.163 | 104 | 0.692 | 0.839 | 2.42E-06 | 14.7 |
| RaGOO_A02_89695 | A02 | 18548244 | 5.00E-10 | 0.159 | 104 | 0.692 | 0.839 | 2.42E-06 | 14.7 |
| RaGOO_A02_97621 | A02 | 20185403 | 5.00E-10 | 0.125 | 104 | 0.692 | 0.839 | 2.42E-06 | 14.7 |
| RaGOO_A02_99058 | A02 | 20478161 | 5.04E-10 | 0.178 | 104 | 0.692 | 0.839 | 2.43E-06 | 14.7 |
| RaGOO_A02_115761 | A02 | 24515078 | 5.08E-10 | 0.168 | 104 | 0.692 | 0.838 | 2.45E-06 | 14.6 |
| RaGOO_A02_110561 | A02 | 22834996 | 5.09E-10 | 0.163 | 104 | 0.692 | 0.838 | 2.45E-06 | 14.6 |
| RaGOO_A02_103555 | A02 | 21382563 | 5.09E-10 | 0.154 | 104 | 0.692 | 0.838 | 2.45E-06 | 14.6 |
| RaGOO_A02_103381 | A02 | 21325098 | 5.12E-10 | 0.173 | 104 | 0.692 | 0.838 | 2.46E-06 | 14.6 |
| RaGOO_A02_80976 | A02 | 16992229 | 5.17E-10 | 0.178 | 104 | 0.692 | 0.838 | 2.48E-06 | 14.6 |
| RaGOO_A02_89686 | A02 | 18546910 | 5.18E-10 | 0.154 | 104 | 0.692 | 0.838 | 2.48E-06 | 14.6 |
| RaGOO_A02_114981 | A02 | 24408746 | 5.20E-10 | 0.173 | 104 | 0.692 | 0.838 | 2.48E-06 | 14.6 |
| RaGOO_A02_99041 | A02 | 20476237 | 5.20E-10 | 0.183 | 104 | 0.692 | 0.838 | 2.48E-06 | 14.6 |
| RaGOO_A02_105433 | A02 | 21821495 | 5.24E-10 | 0.159 | 104 | 0.692 | 0.838 | 2.50E-06 | 14.6 |
| RaGOO_A02_103428 | A02 | 21341829 | 5.27E-10 | 0.130 | 104 | 0.692 | 0.838 | 2.50E-06 | 14.6 |
| RaGOO_A02_120965 | A02 | 25417446 | 5.27E-10 | 0.154 | 104 | 0.692 | 0.838 | 2.50E-06 | 14.6 |

| RaGOO_A02_91894 | A02 | 18880576 | 5.30E-10 | 0.188 | 104 | 0.692 | 0.838 | 2.51E-06 | 14.6 |
| --- | --- | --- | --- | --- | --- | --- | --- | --- | --- |
| RaGOO_A02_91958 | A02 | 18886275 | 5.33E-10 | 0.183 | 104 | 0.692 | 0.838 | 2.52E-06 | 14.6 |
| RaGOO_A02_101875 | A02 | 21037049 | 5.39E-10 | 0.163 | 104 | 0.692 | 0.838 | 2.54E-06 | 14.6 |
| RaGOO_A02_91884 | A02 | 18879556 | 5.39E-10 | 0.183 | 104 | 0.692 | 0.838 | 2.54E-06 | 14.6 |
| RaGOO_A02_91886 | A02 | 18879698 | 5.39E-10 | 0.183 | 104 | 0.692 | 0.838 | 2.54E-06 | 14.6 |
| RaGOO_A02_110241 | A02 | 22784753 | 5.41E-10 | 0.159 | 104 | 0.692 | 0.838 | 2.54E-06 | 14.6 |
| RaGOO_A02_108382 | A02 | 22389408 | 5.42E-10 | 0.159 | 104 | 0.692 | 0.838 | 2.54E-06 | 14.6 |
| RaGOO_A02_100132 | A02 | 20698229 | 5.50E-10 | 0.159 | 104 | 0.692 | 0.838 | 2.58E-06 | 14.6 |
| RaGOO_A02_113322 | A02 | 24075514 | 5.50E-10 | 0.192 | 104 | 0.692 | 0.838 | 2.58E-06 | 14.6 |
| RaGOO_A02_92071 | A02 | 18898339 | 5.54E-10 | 0.168 | 104 | 0.692 | 0.838 | 2.59E-06 | 14.6 |
| RaGOO_A02_75265 | A02 | 16171964 | 5.55E-10 | 0.163 | 104 | 0.692 | 0.838 | 2.59E-06 | 14.6 |
| RaGOO_A02_89685 | A02 | 18546879 | 5.56E-10 | 0.159 | 104 | 0.692 | 0.838 | 2.59E-06 | 14.6 |
| RaGOO_A02_108465 | A02 | 22395411 | 5.56E-10 | 0.159 | 104 | 0.692 | 0.838 | 2.59E-06 | 14.6 |
| RaGOO_A02_89718 | A02 | 18552108 | 5.58E-10 | 0.163 | 104 | 0.692 | 0.838 | 2.59E-06 | 14.6 |
| RaGOO_A02_89720 | A02 | 18552170 | 5.58E-10 | 0.163 | 104 | 0.692 | 0.838 | 2.59E-06 | 14.6 |
| RaGOO_A02_77656 | A02 | 16561328 | 5.60E-10 | 0.154 | 104 | 0.692 | 0.838 | 2.59E-06 | 14.6 |
| RaGOO_A02_100857 | A02 | 20892946 | 5.61E-10 | 0.163 | 104 | 0.692 | 0.838 | 2.59E-06 | 14.6 |
| RaGOO_A02_113750 | A02 | 24203772 | 5.61E-10 | 0.168 | 104 | 0.692 | 0.838 | 2.59E-06 | 14.6 |
| RaGOO_A02_101915 | A02 | 21040671 | 5.61E-10 | 0.168 | 104 | 0.692 | 0.838 | 2.59E-06 | 14.6 |
| RaGOO_A02_102856 | A02 | 21171649 | 5.64E-10 | 0.168 | 104 | 0.692 | 0.838 | 2.60E-06 | 14.6 |
| RaGOO_A02_118214 | A02 | 24865069 | 5.66E-10 | 0.159 | 104 | 0.692 | 0.838 | 2.61E-06 | 14.5 |
| RaGOO_A02_73057 | A02 | 15723004 | 5.69E-10 | 0.154 | 104 | 0.692 | 0.837 | 2.61E-06 | 14.5 |
| RaGOO_A02_89721 | A02 | 18552204 | 5.70E-10 | 0.159 | 104 | 0.692 | 0.837 | 2.61E-06 | 14.5 |
| RaGOO_A02_90235 | A02 | 18615741 | 5.77E-10 | 0.183 | 104 | 0.692 | 0.837 | 2.62E-06 | 14.5 |
| RaGOO_A02_98049 | A02 | 20256201 | 5.77E-10 | 0.163 | 104 | 0.692 | 0.837 | 2.62E-06 | 14.5 |
| RaGOO_A02_110219 | A02 | 22781777 | 5.78E-10 | 0.163 | 104 | 0.692 | 0.837 | 2.62E-06 | 14.5 |
| RaGOO_A02_103029 | A02 | 21222511 | 5.78E-10 | 0.163 | 104 | 0.692 | 0.837 | 2.62E-06 | 14.5 |
| RaGOO_A02_92356 | A02 | 18925927 | 5.81E-10 | 0.168 | 104 | 0.692 | 0.837 | 2.62E-06 | 14.5 |
| RaGOO_A02_90522 | A02 | 18669225 | 5.82E-10 | 0.154 | 104 | 0.692 | 0.837 | 2.62E-06 | 14.5 |
| RaGOO_A02_115422 | A02 | 24467089 | 5.83E-10 | 0.188 | 104 | 0.692 | 0.837 | 2.62E-06 | 14.5 |
| RaGOO_A02_102946 | A02 | 21208441 | 5.83E-10 | 0.192 | 104 | 0.692 | 0.837 | 2.62E-06 | 14.5 |
| RaGOO_A02_89947 | A02 | 18581295 | 5.83E-10 | 0.168 | 104 | 0.692 | 0.837 | 2.62E-06 | 14.5 |
| RaGOO_A02_113275 | A02 | 24060386 | 5.83E-10 | 0.163 | 104 | 0.692 | 0.837 | 2.62E-06 | 14.5 |
| RaGOO_A02_114136 | A02 | 24269642 | 5.84E-10 | 0.178 | 104 | 0.692 | 0.837 | 2.62E-06 | 14.5 |
| RaGOO_A02_108255 | A02 | 22378820 | 5.84E-10 | 0.163 | 104 | 0.692 | 0.837 | 2.62E-06 | 14.5 |
| RaGOO_A02_102066 | A02 | 21069740 | 5.86E-10 | 0.168 | 104 | 0.692 | 0.837 | 2.62E-06 | 14.5 |
| RaGOO_A02_88196 | A02 | 18203099 | 5.87E-10 | 0.173 | 104 | 0.692 | 0.837 | 2.62E-06 | 14.5 |
| RaGOO_A02_88198 | A02 | 18203138 | 5.87E-10 | 0.173 | 104 | 0.692 | 0.837 | 2.62E-06 | 14.5 |
| RaGOO_A02_88197 | A02 | 18203122 | 5.87E-10 | 0.173 | 104 | 0.692 | 0.837 | 2.62E-06 | 14.5 |
| RaGOO_A02_88199 | A02 | 18203152 | 5.87E-10 | 0.173 | 104 | 0.692 | 0.837 | 2.62E-06 | 14.5 |
| RaGOO_A02_97308 | A02 | 20141964 | 5.88E-10 | 0.168 | 104 | 0.692 | 0.837 | 2.62E-06 | 14.5 |
| RaGOO_A02_75311 | A02 | 16173988 | 5.91E-10 | 0.178 | 104 | 0.692 | 0.837 | 2.63E-06 | 14.5 |
| RaGOO_A02_106854 | A02 | 22022074 | 5.91E-10 | 0.159 | 104 | 0.692 | 0.837 | 2.63E-06 | 14.5 |
| RaGOO_A02_82379 | A02 | 17224776 | 5.92E-10 | 0.154 | 104 | 0.692 | 0.837 | 2.63E-06 | 14.5 |
| RaGOO_A02_80140 | A02 | 16917539 | 6.09E-10 | 0.159 | 104 | 0.692 | 0.837 | 2.70E-06 | 14.5 |
| RaGOO_A02_103223 | A02 | 21294921 | 6.10E-10 | 0.168 | 104 | 0.692 | 0.837 | 2.70E-06 | 14.5 |
| RaGOO_A02_115867 | A02 | 24528700 | 6.11E-10 | 0.173 | 104 | 0.692 | 0.837 | 2.70E-06 | 14.5 |
| RaGOO_A02_104249 | A02 | 21554429 | 6.12E-10 | 0.163 | 104 | 0.692 | 0.837 | 2.70E-06 | 14.5 |
| RaGOO_A02_116195 | A02 | 24559628 | 6.13E-10 | 0.173 | 104 | 0.692 | 0.837 | 2.70E-06 | 14.5 |
| RaGOO_A02_73181 | A02 | 15739662 | 6.14E-10 | 0.159 | 104 | 0.692 | 0.837 | 2.70E-06 | 14.5 |
| RaGOO_A02_97070 | A02 | 20102209 | 6.14E-10 | 0.178 | 104 | 0.692 | 0.837 | 2.70E-06 | 14.5 |
| RaGOO_A02_87317 | A02 | 18049638 | 6.15E-10 | 0.159 | 104 | 0.692 | 0.837 | 2.71E-06 | 14.5 |
| RaGOO_A02_73219 | A02 | 15743274 | 6.22E-10 | 0.149 | 104 | 0.692 | 0.837 | 2.73E-06 | 14.5 |
| RaGOO_A02_91613 | A02 | 18856533 | 6.25E-10 | 0.178 | 104 | 0.692 | 0.837 | 2.74E-06 | 14.5 |
| RaGOO_A02_115845 | A02 | 24521602 | 6.27E-10 | 0.168 | 104 | 0.692 | 0.837 | 2.74E-06 | 14.5 |
| RaGOO_A02_110220 | A02 | 22781831 | 6.28E-10 | 0.168 | 104 | 0.692 | 0.837 | 2.74E-06 | 14.5 |
| RaGOO_A02_110221 | A02 | 22781888 | 6.28E-10 | 0.168 | 104 | 0.692 | 0.837 | 2.74E-06 | 14.5 |
| RaGOO_A02_119166 | A02 | 25034865 | 6.28E-10 | 0.159 | 104 | 0.692 | 0.837 | 2.74E-06 | 14.5 |
| RaGOO_A02_108584 | A02 | 22423667 | 6.34E-10 | 0.173 | 104 | 0.692 | 0.836 | 2.76E-06 | 14.4 |
| RaGOO_A02_82249 | A02 | 17217690 | 6.41E-10 | 0.163 | 104 | 0.692 | 0.836 | 2.79E-06 | 14.4 |
| RaGOO_A02_112530 | A02 | 23949636 | 6.42E-10 | 0.163 | 104 | 0.692 | 0.836 | 2.79E-06 | 14.4 |
| RaGOO_A02_99640 | A02 | 20566933 | 6.42E-10 | 0.168 | 104 | 0.692 | 0.836 | 2.79E-06 | 14.4 |
| RaGOO_A02_80160 | A02 | 16919118 | 6.43E-10 | 0.159 | 104 | 0.692 | 0.836 | 2.79E-06 | 14.4 |
| RaGOO_A02_105497 | A02 | 21834524 | 6.45E-10 | 0.163 | 104 | 0.692 | 0.836 | 2.79E-06 | 14.4 |
| RaGOO_A02_118703 | A02 | 24969807 | 6.45E-10 | 0.178 | 104 | 0.692 | 0.836 | 2.79E-06 | 14.4 |
| RaGOO_A02_104318 | A02 | 21564318 | 6.48E-10 | 0.159 | 104 | 0.692 | 0.836 | 2.80E-06 | 14.4 |
| RaGOO_A02_84064 | A02 | 17425320 | 6.53E-10 | 0.159 | 104 | 0.692 | 0.836 | 2.81E-06 | 14.4 |
| RaGOO_A02_87796 | A02 | 18143542 | 6.54E-10 | 0.173 | 104 | 0.692 | 0.836 | 2.81E-06 | 14.4 |
| RaGOO_A02_97806 | A02 | 20217217 | 6.60E-10 | 0.197 | 104 | 0.692 | 0.836 | 2.83E-06 | 14.4 |
| RaGOO_A02_108572 | A02 | 22422788 | 6.60E-10 | 0.154 | 104 | 0.692 | 0.836 | 2.83E-06 | 14.4 |
| RaGOO_A02_100964 | A02 | 20927556 | 6.61E-10 | 0.173 | 104 | 0.692 | 0.836 | 2.83E-06 | 14.4 |
| RaGOO_A02_115848 | A02 | 24521759 | 6.61E-10 | 0.163 | 104 | 0.692 | 0.836 | 2.83E-06 | 14.4 |
| RaGOO_A02_97633 | A02 | 20186521 | 6.64E-10 | 0.188 | 104 | 0.692 | 0.836 | 2.84E-06 | 14.4 |
| RaGOO_A02_78929 | A02 | 16767828 | 6.68E-10 | 0.163 | 104 | 0.692 | 0.836 | 2.85E-06 | 14.4 |
| RaGOO_A02_97259 | A02 | 20136143 | 6.68E-10 | 0.163 | 104 | 0.692 | 0.836 | 2.85E-06 | 14.4 |
| RaGOO_A02_101872 | A02 | 21036956 | 6.73E-10 | 0.163 | 104 | 0.692 | 0.836 | 2.86E-06 | 14.4 |
| RaGOO_A02_73121 | A02 | 15729043 | 6.75E-10 | 0.159 | 104 | 0.692 | 0.836 | 2.87E-06 | 14.4 |
| RaGOO_A02_115768 | A02 | 24515468 | 6.76E-10 | 0.154 | 104 | 0.692 | 0.836 | 2.87E-06 | 14.4 |
| RaGOO_A02_112069 | A02 | 23881608 | 6.80E-10 | 0.144 | 104 | 0.692 | 0.836 | 2.88E-06 | 14.4 |
| RaGOO_A02_101845 | A02 | 21033241 | 6.86E-10 | 0.163 | 104 | 0.692 | 0.836 | 2.90E-06 | 14.4 |
| RaGOO_A02_91751 | A02 | 18865580 | 6.86E-10 | 0.173 | 104 | 0.692 | 0.836 | 2.90E-06 | 14.4 |
| RaGOO_A02_86921 | A02 | 17982805 | 6.91E-10 | 0.163 | 104 | 0.692 | 0.836 | 2.92E-06 | 14.4 |
| RaGOO_A02_116090 | A02 | 24548209 | 6.97E-10 | 0.154 | 104 | 0.692 | 0.836 | 2.94E-06 | 14.4 |
| RaGOO_A02_114479 | A02 | 24331202 | 6.98E-10 | 0.163 | 104 | 0.692 | 0.836 | 2.94E-06 | 14.4 |
| RaGOO_A02_105759 | A02 | 21883314 | 7.01E-10 | 0.163 | 104 | 0.692 | 0.836 | 2.95E-06 | 14.4 |
| RaGOO_A02_116061 | A02 | 24546740 | 7.02E-10 | 0.149 | 104 | 0.692 | 0.836 | 2.95E-06 | 14.4 |
| RaGOO_A02_109508 | A02 | 22635007 | 7.04E-10 | 0.163 | 104 | 0.692 | 0.836 | 2.95E-06 | 14.4 |
| RaGOO_A02_87297 | A02 | 18048385 | 7.04E-10 | 0.168 | 104 | 0.692 | 0.836 | 2.95E-06 | 14.4 |
| RaGOO_A02_91322 | A02 | 18817641 | 7.17E-10 | 0.154 | 104 | 0.692 | 0.835 | 3.00E-06 | 14.3 |
| RaGOO_A02_97727 | A02 | 20199387 | 7.18E-10 | 0.168 | 104 | 0.692 | 0.835 | 3.00E-06 | 14.3 |
| RaGOO_A02_75386 | A02 | 16182875 | 7.20E-10 | 0.183 | 104 | 0.692 | 0.835 | 3.00E-06 | 14.3 |

| RaGOO_A02_113749 | A02 | 24203717 | 7.27E-10 | 0.178 | 104 | 0.692 | 0.835 | 3.03E-06 | 14.3 |
| --- | --- | --- | --- | --- | --- | --- | --- | --- | --- |
| RaGOO_A02_91744 | A02 | 18865376 | 7.29E-10 | 0.188 | 104 | 0.692 | 0.835 | 3.03E-06 | 14.3 |
| RaGOO_A02_73829 | A02 | 15866791 | 7.30E-10 | 0.168 | 104 | 0.692 | 0.835 | 3.03E-06 | 14.3 |
| RaGOO_A02_82341 | A02 | 17222603 | 7.35E-10 | 0.154 | 104 | 0.692 | 0.835 | 3.05E-06 | 14.3 |
| RaGOO_A02_89291 | A02 | 18471357 | 7.37E-10 | 0.159 | 104 | 0.692 | 0.835 | 3.05E-06 | 14.3 |
| RaGOO_A02_104934 | A02 | 21728101 | 7.41E-10 | 0.163 | 104 | 0.692 | 0.835 | 3.06E-06 | 14.3 |
| RaGOO_A02_108972 | A02 | 22549331 | 7.41E-10 | 0.163 | 104 | 0.692 | 0.835 | 3.06E-06 | 14.3 |
| RaGOO_A02_89435 | A02 | 18512700 | 7.44E-10 | 0.173 | 104 | 0.692 | 0.835 | 3.06E-06 | 14.3 |
| RaGOO_A02_89172 | A02 | 18429340 | 7.44E-10 | 0.163 | 104 | 0.692 | 0.835 | 3.06E-06 | 14.3 |
| RaGOO_A02_113905 | A02 | 24235015 | 7.50E-10 | 0.163 | 104 | 0.692 | 0.835 | 3.08E-06 | 14.3 |
| RaGOO_A02_115781 | A02 | 24516689 | 7.51E-10 | 0.173 | 104 | 0.692 | 0.835 | 3.08E-06 | 14.3 |
| RaGOO_A02_100694 | A02 | 20855865 | 7.63E-10 | 0.168 | 104 | 0.692 | 0.835 | 3.13E-06 | 14.3 |
| RaGOO_A02_116287 | A02 | 24567705 | 7.69E-10 | 0.178 | 104 | 0.692 | 0.835 | 3.15E-06 | 14.3 |
| RaGOO_A02_91921 | A02 | 18883036 | 7.69E-10 | 0.173 | 104 | 0.692 | 0.835 | 3.15E-06 | 14.3 |
| RaGOO_A02_116071 | A02 | 24547113 | 7.74E-10 | 0.154 | 104 | 0.692 | 0.835 | 3.16E-06 | 14.3 |
| RaGOO_A02_105432 | A02 | 21821426 | 7.75E-10 | 0.159 | 104 | 0.692 | 0.835 | 3.16E-06 | 14.3 |
| RaGOO_A02_106287 | A02 | 21954111 | 7.77E-10 | 0.154 | 104 | 0.692 | 0.835 | 3.17E-06 | 14.3 |
| RaGOO_A02_110905 | A02 | 22902452 | 7.77E-10 | 0.163 | 104 | 0.692 | 0.835 | 3.17E-06 | 14.3 |
| RaGOO_A02_116193 | A02 | 24559449 | 7.88E-10 | 0.173 | 104 | 0.692 | 0.835 | 3.19E-06 | 14.3 |
| RaGOO_A02_96696 | A02 | 20053050 | 7.90E-10 | 0.120 | 104 | 0.692 | 0.835 | 3.19E-06 | 14.3 |
| RaGOO_A02_110244 | A02 | 22784916 | 7.90E-10 | 0.163 | 104 | 0.692 | 0.835 | 3.19E-06 | 14.3 |
| RaGOO_A02_116320 | A02 | 24569849 | 7.91E-10 | 0.168 | 104 | 0.692 | 0.835 | 3.19E-06 | 14.3 |
| RaGOO_A02_100142 | A02 | 20700383 | 7.92E-10 | 0.163 | 104 | 0.692 | 0.835 | 3.19E-06 | 14.3 |
| RaGOO_A02_86938 | A02 | 17983974 | 8.03E-10 | 0.168 | 104 | 0.692 | 0.834 | 3.24E-06 | 14.2 |
| RaGOO_A02_116059 | A02 | 24546613 | 8.05E-10 | 0.154 | 104 | 0.692 | 0.834 | 3.24E-06 | 14.2 |
| RaGOO_A02_91389 | A02 | 18824110 | 8.07E-10 | 0.159 | 104 | 0.692 | 0.834 | 3.24E-06 | 14.2 |
| RaGOO_A02_83618 | A02 | 17373858 | 8.08E-10 | 0.159 | 104 | 0.692 | 0.834 | 3.24E-06 | 14.2 |
| RaGOO_A02_96545 | A02 | 20025130 | 8.09E-10 | 0.154 | 104 | 0.692 | 0.834 | 3.24E-06 | 14.2 |
| RaGOO_A02_91509 | A02 | 18848985 | 8.10E-10 | 0.173 | 104 | 0.692 | 0.834 | 3.24E-06 | 14.2 |
| RaGOO_A02_92222 | A02 | 18912730 | 8.11E-10 | 0.159 | 104 | 0.692 | 0.834 | 3.24E-06 | 14.2 |
| RaGOO_A02_97645 | A02 | 20186960 | 8.15E-10 | 0.188 | 104 | 0.692 | 0.834 | 3.26E-06 | 14.2 |
| RaGOO_A02_113881 | A02 | 24234366 | 8.19E-10 | 0.168 | 104 | 0.692 | 0.834 | 3.27E-06 | 14.2 |
| RaGOO_A02_91986 | A02 | 18889016 | 8.22E-10 | 0.168 | 104 | 0.692 | 0.834 | 3.28E-06 | 14.2 |
| RaGOO_A02_97913 | A02 | 20227426 | 8.25E-10 | 0.163 | 104 | 0.692 | 0.834 | 3.28E-06 | 14.2 |
| RaGOO_A02_80264 | A02 | 16929471 | 8.26E-10 | 0.163 | 104 | 0.692 | 0.834 | 3.28E-06 | 14.2 |
| RaGOO_A02_118635 | A02 | 24956792 | 8.32E-10 | 0.163 | 104 | 0.692 | 0.834 | 3.30E-06 | 14.2 |
| RaGOO_A02_75397 | A02 | 16183678 | 8.35E-10 | 0.173 | 104 | 0.692 | 0.834 | 3.31E-06 | 14.2 |
| RaGOO_A02_72485 | A02 | 15585257 | 8.41E-10 | 0.144 | 104 | 0.692 | 0.834 | 3.33E-06 | 14.2 |
| RaGOO_A02_102568 | A02 | 21141152 | 8.42E-10 | 0.163 | 104 | 0.692 | 0.834 | 3.33E-06 | 14.2 |
| RaGOO_A02_105794 | A02 | 21886378 | 8.45E-10 | 0.154 | 104 | 0.692 | 0.834 | 3.33E-06 | 14.2 |
| RaGOO_A02_97586 | A02 | 20183119 | 8.48E-10 | 0.159 | 104 | 0.692 | 0.834 | 3.34E-06 | 14.2 |
| RaGOO_A02_72646 | A02 | 15607694 | 8.55E-10 | 0.154 | 104 | 0.692 | 0.834 | 3.37E-06 | 14.2 |
| RaGOO_A02_105726 | A02 | 21880383 | 8.61E-10 | 0.168 | 104 | 0.692 | 0.834 | 3.38E-06 | 14.2 |
| RaGOO_A02_91853 | A02 | 18877386 | 8.61E-10 | 0.173 | 104 | 0.692 | 0.834 | 3.38E-06 | 14.2 |
| RaGOO_A02_75398 | A02 | 16183843 | 8.62E-10 | 0.149 | 104 | 0.692 | 0.834 | 3.38E-06 | 14.2 |
| RaGOO_A02_95993 | A02 | 19942452 | 8.62E-10 | 0.159 | 104 | 0.692 | 0.834 | 3.38E-06 | 14.2 |
| RaGOO_A02_91747 | A02 | 18865473 | 8.67E-10 | 0.178 | 104 | 0.692 | 0.834 | 3.39E-06 | 14.2 |
| RaGOO_A02_79776 | A02 | 16873022 | 8.68E-10 | 0.163 | 104 | 0.692 | 0.834 | 3.39E-06 | 14.2 |
| RaGOO_A02_123251 | A02 | 25693067 | 8.74E-10 | 0.159 | 104 | 0.692 | 0.834 | 3.41E-06 | 14.2 |
| RaGOO_A02_103092 | A02 | 21230094 | 8.79E-10 | 0.183 | 104 | 0.692 | 0.834 | 3.43E-06 | 14.2 |
| RaGOO_A02_82223 | A02 | 17216472 | 8.83E-10 | 0.159 | 104 | 0.692 | 0.834 | 3.43E-06 | 14.2 |
| RaGOO_A02_118701 | A02 | 24969471 | 8.84E-10 | 0.173 | 104 | 0.692 | 0.834 | 3.43E-06 | 14.2 |
| RaGOO_A02_104284 | A02 | 21558960 | 8.86E-10 | 0.159 | 104 | 0.692 | 0.834 | 3.43E-06 | 14.2 |
| RaGOO_A02_91961 | A02 | 18886458 | 8.86E-10 | 0.178 | 104 | 0.692 | 0.834 | 3.43E-06 | 14.2 |
| RaGOO_A02_97139 | A02 | 20116701 | 8.86E-10 | 0.178 | 104 | 0.692 | 0.834 | 3.43E-06 | 14.2 |
| RaGOO_A02_118633 | A02 | 24956726 | 8.87E-10 | 0.159 | 104 | 0.692 | 0.834 | 3.43E-06 | 14.2 |
| RaGOO_A02_73148 | A02 | 15737158 | 8.96E-10 | 0.154 | 104 | 0.692 | 0.833 | 3.46E-06 | 14.1 |
| RaGOO_A02_82365 | A02 | 17224090 | 9.04E-10 | 0.163 | 104 | 0.692 | 0.833 | 3.48E-06 | 14.1 |
| RaGOO_A02_88706 | A02 | 18274831 | 9.04E-10 | 0.163 | 104 | 0.692 | 0.833 | 3.48E-06 | 14.1 |
| RaGOO_A02_80181 | A02 | 16920630 | 9.05E-10 | 0.149 | 104 | 0.692 | 0.833 | 3.48E-06 | 14.1 |
| RaGOO_A02_80182 | A02 | 16920637 | 9.05E-10 | 0.149 | 104 | 0.692 | 0.833 | 3.48E-06 | 14.1 |
| RaGOO_A02_91053 | A02 | 18753959 | 9.05E-10 | 0.178 | 104 | 0.692 | 0.833 | 3.48E-06 | 14.1 |
| RaGOO_A02_108917 | A02 | 22543384 | 9.07E-10 | 0.154 | 104 | 0.692 | 0.833 | 3.48E-06 | 14.1 |
| RaGOO_A02_82254 | A02 | 17218149 | 9.07E-10 | 0.159 | 104 | 0.692 | 0.833 | 3.48E-06 | 14.1 |
| RaGOO_A02_82384 | A02 | 17225010 | 9.12E-10 | 0.168 | 104 | 0.692 | 0.833 | 3.49E-06 | 14.1 |
| RaGOO_A02_114497 | A02 | 24332371 | 9.13E-10 | 0.183 | 104 | 0.692 | 0.833 | 3.49E-06 | 14.1 |
| RaGOO_A02_123601 | A02 | 25724430 | 9.16E-10 | 0.168 | 104 | 0.692 | 0.833 | 3.50E-06 | 14.1 |
| RaGOO_A02_77025 | A02 | 16419345 | 9.16E-10 | 0.178 | 104 | 0.692 | 0.833 | 3.50E-06 | 14.1 |
| RaGOO_A02_118218 | A02 | 24865535 | 9.21E-10 | 0.159 | 104 | 0.692 | 0.833 | 3.51E-06 | 14.1 |
| RaGOO_A02_116311 | A02 | 24569009 | 9.21E-10 | 0.154 | 104 | 0.692 | 0.833 | 3.51E-06 | 14.1 |
| RaGOO_A02_102169 | A02 | 21085309 | 9.29E-10 | 0.154 | 104 | 0.692 | 0.833 | 3.53E-06 | 14.1 |
| RaGOO_A02_110127 | A02 | 22769797 | 9.33E-10 | 0.173 | 104 | 0.692 | 0.833 | 3.54E-06 | 14.1 |
| RaGOO_A02_115773 | A02 | 24515914 | 9.40E-10 | 0.154 | 104 | 0.692 | 0.833 | 3.56E-06 | 14.1 |
| RaGOO_A02_118044 | A02 | 24836197 | 9.40E-10 | 0.178 | 104 | 0.692 | 0.833 | 3.56E-06 | 14.1 |
| RaGOO_A02_75388 | A02 | 16183066 | 9.46E-10 | 0.154 | 104 | 0.692 | 0.833 | 3.58E-06 | 14.1 |
| RaGOO_A02_108298 | A02 | 22381942 | 9.47E-10 | 0.168 | 104 | 0.692 | 0.833 | 3.58E-06 | 14.1 |
| RaGOO_A02_100222 | A02 | 20724827 | 9.48E-10 | 0.168 | 104 | 0.692 | 0.833 | 3.58E-06 | 14.1 |
| RaGOO_A02_89267 | A02 | 18465074 | 9.50E-10 | 0.173 | 104 | 0.692 | 0.833 | 3.58E-06 | 14.1 |
| RaGOO_A02_107565 | A02 | 22172716 | 9.58E-10 | 0.135 | 104 | 0.692 | 0.833 | 3.61E-06 | 14.1 |
| RaGOO_A02_120071 | A02 | 25235568 | 9.58E-10 | 0.173 | 104 | 0.692 | 0.833 | 3.61E-06 | 14.1 |
| RaGOO_A02_91712 | A02 | 18862380 | 9.62E-10 | 0.183 | 104 | 0.692 | 0.833 | 3.62E-06 | 14.1 |
| RaGOO_A02_97300 | A02 | 20140178 | 9.64E-10 | 0.168 | 104 | 0.692 | 0.833 | 3.62E-06 | 14.1 |
| RaGOO_A02_99863 | A02 | 20625939 | 9.65E-10 | 0.183 | 104 | 0.692 | 0.833 | 3.62E-06 | 14.1 |
| RaGOO_A02_91484 | A02 | 18846370 | 9.72E-10 | 0.183 | 104 | 0.692 | 0.833 | 3.64E-06 | 14.1 |
| RaGOO_A02_77001 | A02 | 16416638 | 9.74E-10 | 0.154 | 104 | 0.692 | 0.833 | 3.64E-06 | 14.1 |
| RaGOO_A02_82342 | A02 | 17222659 | 9.76E-10 | 0.154 | 104 | 0.692 | 0.833 | 3.65E-06 | 14.1 |
| RaGOO_A02_91657 | A02 | 18859609 | 9.81E-10 | 0.188 | 104 | 0.692 | 0.833 | 3.66E-06 | 14.1 |
| RaGOO_A02_109671 | A02 | 22658337 | 9.88E-10 | 0.168 | 104 | 0.692 | 0.833 | 3.68E-06 | 14.1 |
| RaGOO_A02_109672 | A02 | 22658357 | 9.88E-10 | 0.168 | 104 | 0.692 | 0.833 | 3.68E-06 | 14.1 |
| RaGOO_A02_122337 | A02 | 25569754 | 9.88E-10 | 0.149 | 104 | 0.692 | 0.833 | 3.68E-06 | 14.1 |
| RaGOO_A02_82294 | A02 | 17219861 | 9.94E-10 | 0.163 | 104 | 0.692 | 0.833 | 3.69E-06 | 14.0 |

| RaGOO_A02_106547 | A02 | 21977715 | 1.00E-09 | 0.168 | 104 | 0.692 | 0.832 | 3.72E-06 | 14.0 |
| --- | --- | --- | --- | --- | --- | --- | --- | --- | --- |
| RaGOO_A02_91649 | A02 | 18858672 | 1.01E-09 | 0.183 | 104 | 0.692 | 0.832 | 3.73E-06 | 14.0 |
| RaGOO_A02_111192 | A02 | 22945264 | 1.01E-09 | 0.159 | 104 | 0.692 | 0.832 | 3.75E-06 | 14.0 |
| RaGOO_A02_96297 | A02 | 19990612 | 1.02E-09 | 0.168 | 104 | 0.692 | 0.832 | 3.78E-06 | 14.0 |
| RaGOO_A02_101756 | A02 | 21021987 | 1.02E-09 | 0.168 | 104 | 0.692 | 0.832 | 3.79E-06 | 14.0 |
| RaGOO_A02_118631 | A02 | 24956469 | 1.03E-09 | 0.159 | 104 | 0.692 | 0.832 | 3.80E-06 | 14.0 |
| RaGOO_A02_84301 | A02 | 17468682 | 1.03E-09 | 0.154 | 104 | 0.692 | 0.832 | 3.80E-06 | 14.0 |
| RaGOO_A02_104447 | A02 | 21583221 | 1.04E-09 | 0.139 | 104 | 0.692 | 0.832 | 3.81E-06 | 14.0 |
| RaGOO_A02_91905 | A02 | 18881940 | 1.04E-09 | 0.183 | 104 | 0.692 | 0.832 | 3.83E-06 | 14.0 |
| RaGOO_A02_102657 | A02 | 21148474 | 1.04E-09 | 0.168 | 104 | 0.692 | 0.832 | 3.83E-06 | 14.0 |
| RaGOO_A02_100194 | A02 | 20713982 | 1.05E-09 | 0.163 | 104 | 0.692 | 0.832 | 3.84E-06 | 14.0 |
| RaGOO_A02_106285 | A02 | 21954035 | 1.05E-09 | 0.159 | 104 | 0.692 | 0.832 | 3.84E-06 | 14.0 |
| RaGOO_A02_73319 | A02 | 15763758 | 1.06E-09 | 0.154 | 104 | 0.692 | 0.832 | 3.87E-06 | 14.0 |
| RaGOO_A02_80165 | A02 | 16919292 | 1.06E-09 | 0.163 | 104 | 0.692 | 0.832 | 3.88E-06 | 14.0 |
| RaGOO_A02_82343 | A02 | 17222688 | 1.06E-09 | 0.154 | 104 | 0.692 | 0.832 | 3.88E-06 | 14.0 |
| RaGOO_A02_103042 | A02 | 21223355 | 1.06E-09 | 0.173 | 104 | 0.692 | 0.832 | 3.88E-06 | 14.0 |
| RaGOO_A02_99872 | A02 | 20627099 | 1.07E-09 | 0.115 | 104 | 0.692 | 0.832 | 3.88E-06 | 14.0 |
| RaGOO_A02_110424 | A02 | 22814506 | 1.07E-09 | 0.163 | 104 | 0.692 | 0.832 | 3.88E-06 | 14.0 |
| RaGOO_A02_114131 | A02 | 24268588 | 1.07E-09 | 0.159 | 104 | 0.692 | 0.832 | 3.88E-06 | 14.0 |
| RaGOO_A02_99631 | A02 | 20564196 | 1.07E-09 | 0.168 | 104 | 0.692 | 0.832 | 3.88E-06 | 14.0 |
| RaGOO_A02_114619 | A02 | 24365526 | 1.07E-09 | 0.173 | 104 | 0.692 | 0.832 | 3.89E-06 | 14.0 |
| RaGOO_A02_99668 | A02 | 20573480 | 1.08E-09 | 0.163 | 104 | 0.692 | 0.832 | 3.89E-06 | 14.0 |
| RaGOO_A02_75310 | A02 | 16173810 | 1.08E-09 | 0.163 | 104 | 0.692 | 0.832 | 3.89E-06 | 14.0 |
| RaGOO_A02_98092 | A02 | 20268656 | 1.08E-09 | 0.163 | 104 | 0.692 | 0.832 | 3.89E-06 | 14.0 |
| RaGOO_A02_73198 | A02 | 15741042 | 1.08E-09 | 0.159 | 104 | 0.692 | 0.832 | 3.89E-06 | 14.0 |
| RaGOO_A02_73145 | A02 | 15737072 | 1.09E-09 | 0.159 | 104 | 0.692 | 0.832 | 3.95E-06 | 14.0 |
| RaGOO_A02_117670 | A02 | 24796365 | 1.10E-09 | 0.197 | 104 | 0.692 | 0.832 | 3.95E-06 | 14.0 |
| RaGOO_A02_117669 | A02 | 24796356 | 1.10E-09 | 0.197 | 104 | 0.692 | 0.832 | 3.95E-06 | 14.0 |
| RaGOO_A02_103295 | A02 | 21300133 | 1.10E-09 | 0.154 | 104 | 0.692 | 0.832 | 3.95E-06 | 14.0 |
| RaGOO_A02_100130 | A02 | 20698142 | 1.11E-09 | 0.163 | 104 | 0.692 | 0.832 | 4.00E-06 | 14.0 |
| RaGOO_A02_85730 | A02 | 17725052 | 1.11E-09 | 0.154 | 104 | 0.692 | 0.832 | 4.00E-06 | 13.9 |
| RaGOO_A02_92207 | A02 | 18911665 | 1.12E-09 | 0.173 | 104 | 0.692 | 0.831 | 4.01E-06 | 13.9 |
| RaGOO_A02_70897 | A02 | 15259189 | 1.13E-09 | 0.163 | 104 | 0.692 | 0.831 | 4.04E-06 | 13.9 |
| RaGOO_A02_89272 | A02 | 18467289 | 1.13E-09 | 0.168 | 104 | 0.692 | 0.831 | 4.04E-06 | 13.9 |
| RaGOO_A02_117685 | A02 | 24798054 | 1.14E-09 | 0.188 | 104 | 0.692 | 0.831 | 4.06E-06 | 13.9 |
| RaGOO_A02_84707 | A02 | 17531844 | 1.14E-09 | 0.173 | 104 | 0.692 | 0.831 | 4.06E-06 | 13.9 |
| RaGOO_A02_100678 | A02 | 20854350 | 1.14E-09 | 0.168 | 104 | 0.692 | 0.831 | 4.06E-06 | 13.9 |
| RaGOO_A02_117683 | A02 | 24797729 | 1.14E-09 | 0.173 | 104 | 0.692 | 0.831 | 4.07E-06 | 13.9 |
| RaGOO_A02_78910 | A02 | 16764797 | 1.15E-09 | 0.144 | 104 | 0.692 | 0.831 | 4.07E-06 | 13.9 |
| RaGOO_A02_91750 | A02 | 18865552 | 1.15E-09 | 0.159 | 104 | 0.692 | 0.831 | 4.07E-06 | 13.9 |
| RaGOO_A02_123134 | A02 | 25684906 | 1.15E-09 | 0.154 | 104 | 0.692 | 0.831 | 4.07E-06 | 13.9 |
| RaGOO_A02_116308 | A02 | 24568822 | 1.15E-09 | 0.154 | 104 | 0.692 | 0.831 | 4.07E-06 | 13.9 |
| RaGOO_A02_76701 | A02 | 16328568 | 1.15E-09 | 0.125 | 104 | 0.692 | 0.831 | 4.08E-06 | 13.9 |
| RaGOO_A02_89758 | A02 | 18555981 | 1.16E-09 | 0.188 | 104 | 0.692 | 0.831 | 4.10E-06 | 13.9 |
| RaGOO_A02_80869 | A02 | 16981530 | 1.16E-09 | 0.183 | 104 | 0.692 | 0.831 | 4.10E-06 | 13.9 |
| RaGOO_A02_100387 | A02 | 20792272 | 1.16E-09 | 0.173 | 104 | 0.692 | 0.831 | 4.10E-06 | 13.9 |
| RaGOO_A02_102994 | A02 | 21218265 | 1.17E-09 | 0.159 | 104 | 0.692 | 0.831 | 4.11E-06 | 13.9 |
| RaGOO_A02_117476 | A02 | 24745889 | 1.17E-09 | 0.154 | 104 | 0.692 | 0.831 | 4.11E-06 | 13.9 |
| RaGOO_A02_113739 | A02 | 24203235 | 1.17E-09 | 0.154 | 104 | 0.692 | 0.831 | 4.11E-06 | 13.9 |
| RaGOO_A02_104774 | A02 | 21646915 | 1.17E-09 | 0.159 | 104 | 0.692 | 0.831 | 4.11E-06 | 13.9 |
| RaGOO_A02_115798 | A02 | 24518101 | 1.18E-09 | 0.163 | 104 | 0.692 | 0.831 | 4.12E-06 | 13.9 |
| RaGOO_A02_83550 | A02 | 17351305 | 1.18E-09 | 0.154 | 104 | 0.692 | 0.831 | 4.12E-06 | 13.9 |
| RaGOO_A02_87316 | A02 | 18049601 | 1.19E-09 | 0.154 | 104 | 0.692 | 0.831 | 4.15E-06 | 13.9 |
| RaGOO_A02_87318 | A02 | 18049639 | 1.19E-09 | 0.154 | 104 | 0.692 | 0.831 | 4.15E-06 | 13.9 |
| RaGOO_A02_91492 | A02 | 18846693 | 1.20E-09 | 0.159 | 104 | 0.692 | 0.831 | 4.18E-06 | 13.9 |
| RaGOO_A02_110976 | A02 | 22913723 | 1.20E-09 | 0.173 | 104 | 0.692 | 0.831 | 4.18E-06 | 13.9 |
| RaGOO_A02_117727 | A02 | 24802170 | 1.21E-09 | 0.178 | 104 | 0.692 | 0.831 | 4.22E-06 | 13.9 |
| RaGOO_A02_92449 | A02 | 18936108 | 1.21E-09 | 0.168 | 104 | 0.692 | 0.831 | 4.22E-06 | 13.9 |
| RaGOO_A02_75268 | A02 | 16171983 | 1.22E-09 | 0.154 | 104 | 0.692 | 0.831 | 4.23E-06 | 13.9 |
| RaGOO_A02_89566 | A02 | 18530939 | 1.23E-09 | 0.173 | 104 | 0.692 | 0.831 | 4.26E-06 | 13.9 |
| RaGOO_A02_101744 | A02 | 21011981 | 1.23E-09 | 0.163 | 104 | 0.692 | 0.831 | 4.26E-06 | 13.9 |
| RaGOO_A02_105929 | A02 | 21908745 | 1.24E-09 | 0.159 | 104 | 0.692 | 0.831 | 4.29E-06 | 13.9 |
| RaGOO_A02_96751 | A02 | 20061982 | 1.24E-09 | 0.168 | 104 | 0.692 | 0.831 | 4.30E-06 | 13.9 |
| RaGOO_A02_92066 | A02 | 18898012 | 1.25E-09 | 0.163 | 104 | 0.692 | 0.831 | 4.32E-06 | 13.8 |
| RaGOO_A02_99022 | A02 | 20475371 | 1.26E-09 | 0.159 | 104 | 0.692 | 0.830 | 4.33E-06 | 13.8 |
| RaGOO_A02_109994 | A02 | 22751508 | 1.26E-09 | 0.159 | 104 | 0.692 | 0.830 | 4.33E-06 | 13.8 |
| RaGOO_A02_84062 | A02 | 17425196 | 1.27E-09 | 0.159 | 104 | 0.692 | 0.830 | 4.36E-06 | 13.8 |
| RaGOO_A02_84063 | A02 | 17425232 | 1.27E-09 | 0.159 | 104 | 0.692 | 0.830 | 4.36E-06 | 13.8 |
| RaGOO_A02_102886 | A02 | 21174806 | 1.27E-09 | 0.149 | 104 | 0.692 | 0.830 | 4.37E-06 | 13.8 |
| RaGOO_A02_98255 | A02 | 20290349 | 1.28E-09 | 0.168 | 104 | 0.692 | 0.830 | 4.39E-06 | 13.8 |
| RaGOO_A02_102701 | A02 | 21153341 | 1.28E-09 | 0.159 | 104 | 0.692 | 0.830 | 4.39E-06 | 13.8 |
| RaGOO_A02_100269 | A02 | 20752162 | 1.29E-09 | 0.173 | 104 | 0.692 | 0.830 | 4.40E-06 | 13.8 |
| RaGOO_A02_88366 | A02 | 18218375 | 1.29E-09 | 0.120 | 104 | 0.692 | 0.830 | 4.40E-06 | 13.8 |
| RaGOO_A02_97810 | A02 | 20217552 | 1.29E-09 | 0.313 | 104 | 0.692 | 0.830 | 4.40E-06 | 13.8 |
| RaGOO_A02_101212 | A02 | 20950552 | 1.30E-09 | 0.154 | 104 | 0.692 | 0.830 | 4.43E-06 | 13.8 |
| RaGOO_A02_89631 | A02 | 18539777 | 1.30E-09 | 0.168 | 104 | 0.692 | 0.830 | 4.43E-06 | 13.8 |
| RaGOO_A02_80168 | A02 | 16919480 | 1.30E-09 | 0.159 | 104 | 0.692 | 0.830 | 4.43E-06 | 13.8 |
| RaGOO_A02_99888 | A02 | 20630602 | 1.30E-09 | 0.159 | 104 | 0.692 | 0.830 | 4.43E-06 | 13.8 |
| RaGOO_A02_102119 | A02 | 21076118 | 1.31E-09 | 0.149 | 104 | 0.692 | 0.830 | 4.43E-06 | 13.8 |
| RaGOO_A02_104313 | A02 | 21564085 | 1.31E-09 | 0.163 | 104 | 0.692 | 0.830 | 4.43E-06 | 13.8 |
| RaGOO_A02_91975 | A02 | 18887683 | 1.31E-09 | 0.178 | 104 | 0.692 | 0.830 | 4.43E-06 | 13.8 |
| RaGOO_A02_79613 | A02 | 16857216 | 1.31E-09 | 0.163 | 104 | 0.692 | 0.830 | 4.43E-06 | 13.8 |
| RaGOO_A02_88705 | A02 | 18274827 | 1.32E-09 | 0.168 | 104 | 0.692 | 0.830 | 4.45E-06 | 13.8 |
| RaGOO_A02_91491 | A02 | 18846685 | 1.32E-09 | 0.168 | 104 | 0.692 | 0.830 | 4.45E-06 | 13.8 |
| RaGOO_A02_111929 | A02 | 23856635 | 1.32E-09 | 0.159 | 104 | 0.692 | 0.830 | 4.45E-06 | 13.8 |
| RaGOO_A02_109205 | A02 | 22583111 | 1.32E-09 | 0.168 | 104 | 0.692 | 0.830 | 4.45E-06 | 13.8 |
| RaGOO_A02_91214 | A02 | 18765843 | 1.32E-09 | 0.163 | 104 | 0.692 | 0.830 | 4.45E-06 | 13.8 |
| RaGOO_A02_92219 | A02 | 18912546 | 1.32E-09 | 0.149 | 104 | 0.692 | 0.830 | 4.45E-06 | 13.8 |
| RaGOO_A02_105428 | A02 | 21820919 | 1.33E-09 | 0.163 | 104 | 0.692 | 0.830 | 4.47E-06 | 13.8 |
| RaGOO_A02_103227 | A02 | 21295133 | 1.33E-09 | 0.159 | 104 | 0.692 | 0.830 | 4.48E-06 | 13.8 |
| RaGOO_A02_87927 | A02 | 18155947 | 1.34E-09 | 0.168 | 104 | 0.692 | 0.830 | 4.48E-06 | 13.8 |

| RaGOO_A02_80115 | A02 | 16914769 | 1.34E-09 | 0.144 | 104 | 0.692 | 0.830 | 4.48E-06 | 13.8 |
| --- | --- | --- | --- | --- | --- | --- | --- | --- | --- |
| RaGOO_A02_102134 | A02 | 21077949 | 1.34E-09 | 0.154 | 104 | 0.692 | 0.830 | 4.50E-06 | 13.8 |
| RaGOO_A02_96389 | A02 | 20000549 | 1.35E-09 | 0.168 | 104 | 0.692 | 0.830 | 4.52E-06 | 13.8 |
| RaGOO_A02_110821 | A02 | 22884607 | 1.36E-09 | 0.173 | 104 | 0.692 | 0.830 | 4.54E-06 | 13.8 |
| RaGOO_A02_90725 | A02 | 18689714 | 1.36E-09 | 0.163 | 104 | 0.692 | 0.830 | 4.54E-06 | 13.8 |
| RaGOO_A02_88937 | A02 | 18301452 | 1.36E-09 | 0.125 | 104 | 0.692 | 0.830 | 4.55E-06 | 13.8 |
| RaGOO_A02_96093 | A02 | 19956564 | 1.37E-09 | 0.163 | 104 | 0.692 | 0.830 | 4.55E-06 | 13.8 |
| RaGOO_A02_112980 | A02 | 24014727 | 1.37E-09 | 0.163 | 104 | 0.692 | 0.830 | 4.55E-06 | 13.8 |
| RaGOO_A02_114635 | A02 | 24366231 | 1.37E-09 | 0.168 | 104 | 0.692 | 0.830 | 4.56E-06 | 13.8 |
| RaGOO_A02_84507 | A02 | 17508269 | 1.39E-09 | 0.178 | 104 | 0.692 | 0.830 | 4.61E-06 | 13.8 |
| RaGOO_A02_117672 | A02 | 24796611 | 1.39E-09 | 0.183 | 104 | 0.692 | 0.830 | 4.61E-06 | 13.8 |
| RaGOO_A02_82299 | A02 | 17220000 | 1.39E-09 | 0.163 | 104 | 0.692 | 0.830 | 4.61E-06 | 13.8 |
| RaGOO_A02_83486 | A02 | 17345658 | 1.40E-09 | 0.163 | 104 | 0.692 | 0.830 | 4.63E-06 | 13.7 |
| RaGOO_A02_99886 | A02 | 20629964 | 1.40E-09 | 0.154 | 104 | 0.692 | 0.829 | 4.63E-06 | 13.7 |
| RaGOO_A02_117716 | A02 | 24801102 | 1.41E-09 | 0.163 | 104 | 0.692 | 0.829 | 4.64E-06 | 13.7 |
| RaGOO_A02_104933 | A02 | 21728007 | 1.41E-09 | 0.168 | 104 | 0.692 | 0.829 | 4.65E-06 | 13.7 |
| RaGOO_A02_102971 | A02 | 21216336 | 1.41E-09 | 0.120 | 104 | 0.692 | 0.829 | 4.65E-06 | 13.7 |
| RaGOO_A02_102485 | A02 | 21129285 | 1.42E-09 | 0.173 | 104 | 0.692 | 0.829 | 4.66E-06 | 13.7 |
| RaGOO_A02_114977 | A02 | 24408667 | 1.42E-09 | 0.168 | 104 | 0.692 | 0.829 | 4.66E-06 | 13.7 |
| RaGOO_A02_97646 | A02 | 20186991 | 1.42E-09 | 0.173 | 104 | 0.692 | 0.829 | 4.66E-06 | 13.7 |
| RaGOO_A02_105933 | A02 | 21909075 | 1.42E-09 | 0.173 | 104 | 0.692 | 0.829 | 4.66E-06 | 13.7 |
| RaGOO_A02_123260 | A02 | 25693766 | 1.43E-09 | 0.154 | 104 | 0.692 | 0.829 | 4.67E-06 | 13.7 |
| RaGOO_A02_91858 | A02 | 18877551 | 1.43E-09 | 0.178 | 104 | 0.692 | 0.829 | 4.67E-06 | 13.7 |
| RaGOO_A02_102577 | A02 | 21141750 | 1.43E-09 | 0.168 | 104 | 0.692 | 0.829 | 4.67E-06 | 13.7 |
| RaGOO_A02_99681 | A02 | 20576932 | 1.43E-09 | 0.154 | 104 | 0.692 | 0.829 | 4.67E-06 | 13.7 |
| RaGOO_A02_89828 | A02 | 18562824 | 1.43E-09 | 0.159 | 104 | 0.692 | 0.829 | 4.67E-06 | 13.7 |
| RaGOO_A02_89829 | A02 | 18562825 | 1.43E-09 | 0.159 | 104 | 0.692 | 0.829 | 4.67E-06 | 13.7 |
| RaGOO_A02_89830 | A02 | 18562827 | 1.43E-09 | 0.159 | 104 | 0.692 | 0.829 | 4.67E-06 | 13.7 |
| RaGOO_A02_89823 | A02 | 18562572 | 1.44E-09 | 0.159 | 104 | 0.692 | 0.829 | 4.67E-06 | 13.7 |
| RaGOO_A02_100883 | A02 | 20895190 | 1.44E-09 | 0.159 | 104 | 0.692 | 0.829 | 4.67E-06 | 13.7 |
| RaGOO_A02_92070 | A02 | 18898307 | 1.44E-09 | 0.159 | 104 | 0.692 | 0.829 | 4.67E-06 | 13.7 |
| RaGOO_A02_79777 | A02 | 16873027 | 1.44E-09 | 0.159 | 104 | 0.692 | 0.829 | 4.67E-06 | 13.7 |
| RaGOO_A02_110218 | A02 | 22781265 | 1.45E-09 | 0.159 | 104 | 0.692 | 0.829 | 4.68E-06 | 13.7 |
| RaGOO_A02_84490 | A02 | 17506133 | 1.45E-09 | 0.159 | 104 | 0.692 | 0.829 | 4.68E-06 | 13.7 |
| RaGOO_A02_99657 | A02 | 20571845 | 1.45E-09 | 0.159 | 104 | 0.692 | 0.829 | 4.69E-06 | 13.7 |
| RaGOO_A02_96018 | A02 | 19947844 | 1.46E-09 | 0.154 | 104 | 0.692 | 0.829 | 4.70E-06 | 13.7 |
| RaGOO_A02_111284 | A02 | 23030010 | 1.46E-09 | 0.159 | 104 | 0.692 | 0.829 | 4.71E-06 | 13.7 |
| RaGOO_A02_89269 | A02 | 18466156 | 1.46E-09 | 0.144 | 104 | 0.692 | 0.829 | 4.71E-06 | 13.7 |
| RaGOO_A02_102653 | A02 | 21148189 | 1.47E-09 | 0.144 | 104 | 0.692 | 0.829 | 4.74E-06 | 13.7 |
| RaGOO_A02_118221 | A02 | 24865601 | 1.48E-09 | 0.159 | 104 | 0.692 | 0.829 | 4.74E-06 | 13.7 |
| RaGOO_A02_110582 | A02 | 22837947 | 1.48E-09 | 0.163 | 104 | 0.692 | 0.829 | 4.74E-06 | 13.7 |
| RaGOO_A02_104272 | A02 | 21558073 | 1.48E-09 | 0.159 | 104 | 0.692 | 0.829 | 4.75E-06 | 13.7 |
| RaGOO_A02_118049 | A02 | 24836339 | 1.49E-09 | 0.163 | 104 | 0.692 | 0.829 | 4.77E-06 | 13.7 |
| RaGOO_A02_99859 | A02 | 20625530 | 1.49E-09 | 0.168 | 104 | 0.692 | 0.829 | 4.77E-06 | 13.7 |
| RaGOO_A02_84487 | A02 | 17505680 | 1.50E-09 | 0.159 | 104 | 0.692 | 0.829 | 4.79E-06 | 13.7 |
| RaGOO_A02_99090 | A02 | 20480200 | 1.50E-09 | 0.159 | 104 | 0.692 | 0.829 | 4.79E-06 | 13.7 |
| RaGOO_A02_99875 | A02 | 20627227 | 1.50E-09 | 0.125 | 104 | 0.692 | 0.829 | 4.79E-06 | 13.7 |
| RaGOO_A02_73178 | A02 | 15739514 | 1.51E-09 | 0.149 | 104 | 0.692 | 0.829 | 4.82E-06 | 13.7 |
| RaGOO_A02_97097 | A02 | 20105282 | 1.52E-09 | 0.168 | 104 | 0.692 | 0.829 | 4.82E-06 | 13.7 |
| RaGOO_A02_98029 | A02 | 20253877 | 1.52E-09 | 0.159 | 104 | 0.692 | 0.829 | 4.82E-06 | 13.7 |
| RaGOO_A02_115775 | A02 | 24516057 | 1.52E-09 | 0.159 | 104 | 0.692 | 0.829 | 4.82E-06 | 13.7 |
| RaGOO_A02_80847 | A02 | 16980339 | 1.53E-09 | 0.154 | 104 | 0.692 | 0.829 | 4.84E-06 | 13.7 |
| RaGOO_A02_107802 | A02 | 22194275 | 1.54E-09 | 0.139 | 104 | 0.692 | 0.829 | 4.87E-06 | 13.7 |
| RaGOO_A02_106460 | A02 | 21972916 | 1.55E-09 | 0.168 | 104 | 0.692 | 0.829 | 4.89E-06 | 13.7 |
| RaGOO_A02_73204 | A02 | 15741463 | 1.55E-09 | 0.154 | 104 | 0.692 | 0.829 | 4.89E-06 | 13.7 |
| RaGOO_A02_73205 | A02 | 15741464 | 1.55E-09 | 0.154 | 104 | 0.692 | 0.829 | 4.89E-06 | 13.7 |
| RaGOO_A02_91635 | A02 | 18857031 | 1.55E-09 | 0.125 | 104 | 0.692 | 0.829 | 4.89E-06 | 13.7 |
| RaGOO_A02_104271 | A02 | 21558055 | 1.55E-09 | 0.159 | 104 | 0.692 | 0.829 | 4.90E-06 | 13.7 |
| RaGOO_A02_111226 | A02 | 22947907 | 1.55E-09 | 0.154 | 104 | 0.692 | 0.829 | 4.90E-06 | 13.7 |
| RaGOO_A02_89565 | A02 | 18530893 | 1.56E-09 | 0.178 | 104 | 0.692 | 0.829 | 4.90E-06 | 13.7 |
| RaGOO_A02_91170 | A02 | 18763452 | 1.56E-09 | 0.144 | 104 | 0.692 | 0.829 | 4.90E-06 | 13.7 |
| RaGOO_A02_102570 | A02 | 21141244 | 1.58E-09 | 0.178 | 104 | 0.692 | 0.828 | 4.96E-06 | 13.6 |
| RaGOO_A02_115760 | A02 | 24514966 | 1.58E-09 | 0.183 | 104 | 0.692 | 0.828 | 4.96E-06 | 13.6 |
| RaGOO_A02_116314 | A02 | 24569205 | 1.58E-09 | 0.173 | 104 | 0.692 | 0.828 | 4.96E-06 | 13.6 |
| RaGOO_A02_91823 | A02 | 18875444 | 1.59E-09 | 0.188 | 104 | 0.692 | 0.828 | 4.97E-06 | 13.6 |
| RaGOO_A02_99667 | A02 | 20573380 | 1.59E-09 | 0.163 | 104 | 0.692 | 0.828 | 4.97E-06 | 13.6 |
| RaGOO_A02_102833 | A02 | 21168906 | 1.59E-09 | 0.168 | 104 | 0.692 | 0.828 | 4.97E-06 | 13.6 |
| RaGOO_A02_80170 | A02 | 16919945 | 1.60E-09 | 0.159 | 104 | 0.692 | 0.828 | 5.01E-06 | 13.6 |
| RaGOO_A02_111075 | A02 | 22919910 | 1.61E-09 | 0.178 | 104 | 0.692 | 0.828 | 5.01E-06 | 13.6 |
| RaGOO_A02_116060 | A02 | 24546720 | 1.61E-09 | 0.144 | 104 | 0.692 | 0.828 | 5.01E-06 | 13.6 |
| RaGOO_A02_116054 | A02 | 24546440 | 1.61E-09 | 0.163 | 104 | 0.692 | 0.828 | 5.02E-06 | 13.6 |
| RaGOO_A02_103881 | A02 | 21500268 | 1.62E-09 | 0.168 | 104 | 0.692 | 0.828 | 5.04E-06 | 13.6 |
| RaGOO_A02_110975 | A02 | 22913711 | 1.62E-09 | 0.163 | 104 | 0.692 | 0.828 | 5.05E-06 | 13.6 |
| RaGOO_A02_118048 | A02 | 24836203 | 1.64E-09 | 0.173 | 104 | 0.692 | 0.828 | 5.07E-06 | 13.6 |
| RaGOO_A02_75395 | A02 | 16183562 | 1.64E-09 | 0.163 | 104 | 0.692 | 0.828 | 5.07E-06 | 13.6 |
| RaGOO_A02_103819 | A02 | 21495844 | 1.64E-09 | 0.168 | 104 | 0.692 | 0.828 | 5.08E-06 | 13.6 |
| RaGOO_A02_100216 | A02 | 20722882 | 1.65E-09 | 0.168 | 104 | 0.692 | 0.828 | 5.10E-06 | 13.6 |
| RaGOO_A02_109352 | A02 | 22607390 | 1.67E-09 | 0.183 | 104 | 0.692 | 0.828 | 5.15E-06 | 13.6 |
| RaGOO_A02_115774 | A02 | 24516055 | 1.67E-09 | 0.163 | 104 | 0.692 | 0.828 | 5.15E-06 | 13.6 |
| RaGOO_A02_108590 | A02 | 22424269 | 1.67E-09 | 0.173 | 104 | 0.692 | 0.828 | 5.15E-06 | 13.6 |
| RaGOO_A02_104276 | A02 | 21558516 | 1.67E-09 | 0.163 | 104 | 0.692 | 0.828 | 5.15E-06 | 13.6 |
| RaGOO_A02_104175 | A02 | 21546455 | 1.68E-09 | 0.173 | 104 | 0.692 | 0.828 | 5.17E-06 | 13.6 |
| RaGOO_A02_113906 | A02 | 24235097 | 1.68E-09 | 0.159 | 104 | 0.692 | 0.828 | 5.17E-06 | 13.6 |
| RaGOO_A02_91889 | A02 | 18879978 | 1.68E-09 | 0.168 | 104 | 0.692 | 0.828 | 5.17E-06 | 13.6 |
| RaGOO_A02_91855 | A02 | 18877474 | 1.69E-09 | 0.188 | 104 | 0.692 | 0.828 | 5.17E-06 | 13.6 |
| RaGOO_A02_106289 | A02 | 21954151 | 1.70E-09 | 0.163 | 104 | 0.692 | 0.828 | 5.19E-06 | 13.6 |
| RaGOO_A02_85746 | A02 | 17726498 | 1.70E-09 | 0.183 | 104 | 0.692 | 0.828 | 5.19E-06 | 13.6 |
| RaGOO_A02_116192 | A02 | 24559435 | 1.70E-09 | 0.178 | 104 | 0.692 | 0.828 | 5.19E-06 | 13.6 |
| RaGOO_A02_110211 | A02 | 22780498 | 1.70E-09 | 0.163 | 104 | 0.692 | 0.828 | 5.20E-06 | 13.6 |
| RaGOO_A02_112047 | A02 | 23878978 | 1.70E-09 | 0.154 | 104 | 0.692 | 0.828 | 5.20E-06 | 13.6 |
| RaGOO_A02_116064 | A02 | 24546904 | 1.71E-09 | 0.154 | 104 | 0.692 | 0.828 | 5.21E-06 | 13.6 |

| RaGOO_A02_103094 | A02 | 21230188 | 1.71E-09 | 0.168 | 104 | 0.692 | 0.828 | 5.22E-06 | 13.6 |
| --- | --- | --- | --- | --- | --- | --- | --- | --- | --- |
| RaGOO_A02_103449 | A02 | 21345147 | 1.72E-09 | 0.183 | 104 | 0.692 | 0.828 | 5.22E-06 | 13.6 |
| RaGOO_A02_76981 | A02 | 16415673 | 1.72E-09 | 0.149 | 104 | 0.692 | 0.828 | 5.22E-06 | 13.6 |
| RaGOO_A02_97630 | A02 | 20186258 | 1.72E-09 | 0.173 | 104 | 0.692 | 0.828 | 5.22E-06 | 13.6 |
| RaGOO_A02_115016 | A02 | 24411989 | 1.73E-09 | 0.178 | 104 | 0.692 | 0.828 | 5.25E-06 | 13.6 |
| RaGOO_A02_82273 | A02 | 17218741 | 1.74E-09 | 0.154 | 104 | 0.692 | 0.828 | 5.26E-06 | 13.6 |
| RaGOO_A02_104771 | A02 | 21646865 | 1.74E-09 | 0.168 | 104 | 0.692 | 0.828 | 5.26E-06 | 13.6 |
| RaGOO_A02_101917 | A02 | 21040847 | 1.74E-09 | 0.168 | 104 | 0.692 | 0.828 | 5.27E-06 | 13.6 |
| RaGOO_A02_97301 | A02 | 20140289 | 1.75E-09 | 0.163 | 104 | 0.692 | 0.828 | 5.29E-06 | 13.6 |
| RaGOO_A02_102592 | A02 | 21142857 | 1.75E-09 | 0.168 | 104 | 0.692 | 0.828 | 5.29E-06 | 13.6 |
| RaGOO_A02_99138 | A02 | 20484595 | 1.75E-09 | 0.159 | 104 | 0.692 | 0.828 | 5.29E-06 | 13.6 |
| RaGOO_A02_78182 | A02 | 16641642 | 1.76E-09 | 0.135 | 104 | 0.692 | 0.827 | 5.31E-06 | 13.5 |
| RaGOO_A02_91794 | A02 | 18873858 | 1.77E-09 | 0.154 | 104 | 0.692 | 0.827 | 5.31E-06 | 13.5 |
| RaGOO_A02_91796 | A02 | 18873862 | 1.77E-09 | 0.154 | 104 | 0.692 | 0.827 | 5.31E-06 | 13.5 |
| RaGOO_A02_91795 | A02 | 18873859 | 1.77E-09 | 0.154 | 104 | 0.692 | 0.827 | 5.31E-06 | 13.5 |
| RaGOO_A02_102089 | A02 | 21072286 | 1.77E-09 | 0.159 | 104 | 0.692 | 0.827 | 5.32E-06 | 13.5 |
| RaGOO_A02_100273 | A02 | 20753741 | 1.77E-09 | 0.168 | 104 | 0.692 | 0.827 | 5.32E-06 | 13.5 |
| RaGOO_A02_90338 | A02 | 18639204 | 1.77E-09 | 0.173 | 104 | 0.692 | 0.827 | 5.32E-06 | 13.5 |
| RaGOO_A02_103235 | A02 | 21295538 | 1.78E-09 | 0.178 | 104 | 0.692 | 0.827 | 5.34E-06 | 13.5 |
| RaGOO_A02_114617 | A02 | 24365486 | 1.79E-09 | 0.173 | 104 | 0.692 | 0.827 | 5.35E-06 | 13.5 |
| RaGOO_A02_80159 | A02 | 16919066 | 1.79E-09 | 0.168 | 104 | 0.692 | 0.827 | 5.36E-06 | 13.5 |
| RaGOO_A02_80158 | A02 | 16919063 | 1.79E-09 | 0.168 | 104 | 0.692 | 0.827 | 5.36E-06 | 13.5 |
| RaGOO_A02_98365 | A02 | 20321219 | 1.81E-09 | 0.149 | 104 | 0.692 | 0.827 | 5.39E-06 | 13.5 |
| RaGOO_A02_97620 | A02 | 20185364 | 1.81E-09 | 0.188 | 104 | 0.692 | 0.827 | 5.40E-06 | 13.5 |
| RaGOO_A02_110790 | A02 | 22881385 | 1.81E-09 | 0.159 | 104 | 0.692 | 0.827 | 5.40E-06 | 13.5 |
| RaGOO_A02_106753 | A02 | 22009152 | 1.82E-09 | 0.159 | 104 | 0.692 | 0.827 | 5.41E-06 | 13.5 |
| RaGOO_A02_118603 | A02 | 24948392 | 1.82E-09 | 0.173 | 104 | 0.692 | 0.827 | 5.41E-06 | 13.5 |
| RaGOO_A02_89683 | A02 | 18546829 | 1.82E-09 | 0.163 | 104 | 0.692 | 0.827 | 5.41E-06 | 13.5 |
| RaGOO_A02_91064 | A02 | 18754644 | 1.84E-09 | 0.159 | 104 | 0.692 | 0.827 | 5.45E-06 | 13.5 |
| RaGOO_A02_114709 | A02 | 24371053 | 1.84E-09 | 0.144 | 104 | 0.692 | 0.827 | 5.46E-06 | 13.5 |
| RaGOO_A02_88142 | A02 | 18197503 | 1.84E-09 | 0.159 | 104 | 0.692 | 0.827 | 5.46E-06 | 13.5 |
| RaGOO_A02_103425 | A02 | 21341592 | 1.85E-09 | 0.125 | 104 | 0.692 | 0.827 | 5.46E-06 | 13.5 |
| RaGOO_A02_113276 | A02 | 24060402 | 1.85E-09 | 0.163 | 104 | 0.692 | 0.827 | 5.47E-06 | 13.5 |
| RaGOO_A02_80834 | A02 | 16979800 | 1.85E-09 | 0.163 | 104 | 0.692 | 0.827 | 5.47E-06 | 13.5 |
| RaGOO_A02_116118 | A02 | 24549916 | 1.86E-09 | 0.163 | 104 | 0.692 | 0.827 | 5.48E-06 | 13.5 |
| RaGOO_A02_116120 | A02 | 24549935 | 1.86E-09 | 0.163 | 104 | 0.692 | 0.827 | 5.48E-06 | 13.5 |
| RaGOO_A02_87524 | A02 | 18098250 | 1.86E-09 | 0.159 | 104 | 0.692 | 0.827 | 5.48E-06 | 13.5 |
| RaGOO_A02_110260 | A02 | 22786184 | 1.87E-09 | 0.159 | 104 | 0.692 | 0.827 | 5.48E-06 | 13.5 |
| RaGOO_A02_106738 | A02 | 22007867 | 1.87E-09 | 0.159 | 104 | 0.692 | 0.827 | 5.48E-06 | 13.5 |
| RaGOO_A02_102282 | A02 | 21103958 | 1.89E-09 | 0.168 | 104 | 0.692 | 0.827 | 5.53E-06 | 13.5 |
| RaGOO_A02_72490 | A02 | 15585349 | 1.91E-09 | 0.149 | 104 | 0.692 | 0.827 | 5.60E-06 | 13.5 |
| RaGOO_A02_98257 | A02 | 20290424 | 1.91E-09 | 0.154 | 104 | 0.692 | 0.827 | 5.60E-06 | 13.5 |
| RaGOO_A02_71351 | A02 | 15351934 | 1.93E-09 | 0.154 | 104 | 0.692 | 0.827 | 5.64E-06 | 13.5 |
| RaGOO_A02_84693 | A02 | 17531042 | 1.93E-09 | 0.159 | 104 | 0.692 | 0.827 | 5.64E-06 | 13.5 |
| RaGOO_A02_101380 | A02 | 20971961 | 1.93E-09 | 0.154 | 104 | 0.692 | 0.827 | 5.64E-06 | 13.5 |
| RaGOO_A02_102170 | A02 | 21085353 | 1.94E-09 | 0.163 | 104 | 0.692 | 0.827 | 5.66E-06 | 13.5 |
| RaGOO_A02_97549 | A02 | 20180390 | 1.94E-09 | 0.154 | 104 | 0.692 | 0.827 | 5.66E-06 | 13.5 |
| RaGOO_A02_91692 | A02 | 18861200 | 1.95E-09 | 0.178 | 104 | 0.692 | 0.827 | 5.67E-06 | 13.5 |
| RaGOO_A02_88682 | A02 | 18273485 | 1.97E-09 | 0.159 | 104 | 0.692 | 0.827 | 5.72E-06 | 13.5 |
| RaGOO_A02_76067 | A02 | 16246563 | 1.98E-09 | 0.173 | 104 | 0.692 | 0.826 | 5.76E-06 | 13.4 |
| RaGOO_A02_105941 | A02 | 21909902 | 1.98E-09 | 0.154 | 104 | 0.692 | 0.826 | 5.76E-06 | 13.4 |
| RaGOO_A02_89869 | A02 | 18568611 | 1.99E-09 | 0.154 | 104 | 0.692 | 0.826 | 5.77E-06 | 13.4 |
| RaGOO_A02_102616 | A02 | 21144832 | 1.99E-09 | 0.173 | 104 | 0.692 | 0.826 | 5.77E-06 | 13.4 |
| RaGOO_A02_111071 | A02 | 22919699 | 1.99E-09 | 0.163 | 104 | 0.692 | 0.826 | 5.77E-06 | 13.4 |
| RaGOO_A02_82514 | A02 | 17232902 | 2.00E-09 | 0.125 | 104 | 0.692 | 0.826 | 5.78E-06 | 13.4 |
| RaGOO_A02_100037 | A02 | 20676342 | 2.00E-09 | 0.168 | 104 | 0.692 | 0.826 | 5.78E-06 | 13.4 |
| RaGOO_A02_92295 | A02 | 18918697 | 2.00E-09 | 0.168 | 104 | 0.692 | 0.826 | 5.79E-06 | 13.4 |
| RaGOO_A02_115882 | A02 | 24530054 | 2.01E-09 | 0.144 | 104 | 0.692 | 0.826 | 5.79E-06 | 13.4 |
| RaGOO_A02_115841 | A02 | 24521087 | 2.01E-09 | 0.159 | 104 | 0.692 | 0.826 | 5.79E-06 | 13.4 |
| RaGOO_A02_118226 | A02 | 24865877 | 2.02E-09 | 0.168 | 104 | 0.692 | 0.826 | 5.83E-06 | 13.4 |
| RaGOO_A02_90371 | A02 | 18641760 | 2.04E-09 | 0.173 | 104 | 0.692 | 0.826 | 5.86E-06 | 13.4 |
| RaGOO_A02_110902 | A02 | 22902101 | 2.05E-09 | 0.159 | 104 | 0.692 | 0.826 | 5.88E-06 | 13.4 |
| RaGOO_A02_102088 | A02 | 21072241 | 2.06E-09 | 0.159 | 104 | 0.692 | 0.826 | 5.90E-06 | 13.4 |
| RaGOO_A02_118437 | A02 | 24910449 | 2.06E-09 | 0.159 | 104 | 0.692 | 0.826 | 5.90E-06 | 13.4 |
| RaGOO_A02_103818 | A02 | 21495839 | 2.06E-09 | 0.168 | 104 | 0.692 | 0.826 | 5.90E-06 | 13.4 |
| RaGOO_A02_118634 | A02 | 24956767 | 2.06E-09 | 0.159 | 104 | 0.692 | 0.826 | 5.90E-06 | 13.4 |
| RaGOO_A02_108798 | A02 | 22463613 | 2.06E-09 | 0.163 | 104 | 0.692 | 0.826 | 5.90E-06 | 13.4 |
| RaGOO_A02_102948 | A02 | 21208548 | 2.07E-09 | 0.115 | 104 | 0.692 | 0.826 | 5.92E-06 | 13.4 |
| RaGOO_A02_72817 | A02 | 15654741 | 2.07E-09 | 0.154 | 104 | 0.692 | 0.826 | 5.92E-06 | 13.4 |
| RaGOO_A02_87806 | A02 | 18143806 | 2.08E-09 | 0.173 | 104 | 0.692 | 0.826 | 5.93E-06 | 13.4 |
| RaGOO_A02_100281 | A02 | 20755916 | 2.08E-09 | 0.159 | 104 | 0.692 | 0.826 | 5.93E-06 | 13.4 |
| RaGOO_A02_106750 | A02 | 22009029 | 2.08E-09 | 0.159 | 104 | 0.692 | 0.826 | 5.93E-06 | 13.4 |
| RaGOO_A02_96826 | A02 | 20074429 | 2.09E-09 | 0.183 | 104 | 0.692 | 0.826 | 5.94E-06 | 13.4 |
| RaGOO_A02_92069 | A02 | 18898190 | 2.09E-09 | 0.159 | 104 | 0.692 | 0.826 | 5.95E-06 | 13.4 |
| RaGOO_A02_73976 | A02 | 15953021 | 2.10E-09 | 0.163 | 104 | 0.692 | 0.826 | 5.96E-06 | 13.4 |
| RaGOO_A02_97776 | A02 | 20203077 | 2.11E-09 | 0.163 | 104 | 0.692 | 0.826 | 5.99E-06 | 13.4 |
| RaGOO_A02_110909 | A02 | 22902817 | 2.13E-09 | 0.163 | 104 | 0.692 | 0.826 | 6.03E-06 | 13.4 |
| RaGOO_A02_77657 | A02 | 16561481 | 2.13E-09 | 0.159 | 104 | 0.692 | 0.826 | 6.04E-06 | 13.4 |
| RaGOO_A02_99803 | A02 | 20608678 | 2.13E-09 | 0.154 | 104 | 0.692 | 0.826 | 6.04E-06 | 13.4 |
| RaGOO_A02_115762 | A02 | 24515093 | 2.14E-09 | 0.163 | 104 | 0.692 | 0.826 | 6.04E-06 | 13.4 |
| RaGOO_A02_91502 | A02 | 18848269 | 2.14E-09 | 0.173 | 104 | 0.692 | 0.826 | 6.04E-06 | 13.4 |
| RaGOO_A02_99864 | A02 | 20626100 | 2.14E-09 | 0.144 | 104 | 0.692 | 0.826 | 6.04E-06 | 13.4 |
| RaGOO_A02_99865 | A02 | 20626102 | 2.14E-09 | 0.144 | 104 | 0.692 | 0.826 | 6.04E-06 | 13.4 |
| RaGOO_A02_73167 | A02 | 15738531 | 2.14E-09 | 0.154 | 104 | 0.692 | 0.826 | 6.04E-06 | 13.4 |
| RaGOO_A02_112528 | A02 | 23949473 | 2.15E-09 | 0.173 | 104 | 0.692 | 0.826 | 6.05E-06 | 13.4 |
| RaGOO_A02_91895 | A02 | 18880616 | 2.15E-09 | 0.168 | 104 | 0.692 | 0.826 | 6.06E-06 | 13.4 |
| RaGOO_A02_115776 | A02 | 24516222 | 2.17E-09 | 0.159 | 104 | 0.692 | 0.826 | 6.10E-06 | 13.4 |
| RaGOO_A02_103858 | A02 | 21497970 | 2.18E-09 | 0.168 | 104 | 0.692 | 0.826 | 6.13E-06 | 13.4 |
| RaGOO_A02_118225 | A02 | 24865830 | 2.19E-09 | 0.173 | 104 | 0.692 | 0.826 | 6.14E-06 | 13.4 |
| RaGOO_A02_102656 | A02 | 21148336 | 2.19E-09 | 0.154 | 104 | 0.692 | 0.826 | 6.14E-06 | 13.4 |
| RaGOO_A02_99035 | A02 | 20475845 | 2.19E-09 | 0.159 | 104 | 0.692 | 0.826 | 6.15E-06 | 13.4 |

| RaGOO_A02_101549 | A02 | 20991300 | 2.21E-09 | 0.168 | 104 | 0.692 | 0.826 | 6.19E-06 | 13.4 |
| --- | --- | --- | --- | --- | --- | --- | --- | --- | --- |
| RaGOO_A02_101550 | A02 | 20991311 | 2.21E-09 | 0.168 | 104 | 0.692 | 0.826 | 6.19E-06 | 13.4 |
| RaGOO_A02_115839 | A02 | 24520830 | 2.22E-09 | 0.154 | 104 | 0.692 | 0.825 | 6.21E-06 | 13.3 |
| RaGOO_A02_77195 | A02 | 16449145 | 2.23E-09 | 0.149 | 104 | 0.692 | 0.825 | 6.22E-06 | 13.3 |
| RaGOO_A02_77000 | A02 | 16416562 | 2.23E-09 | 0.154 | 104 | 0.692 | 0.825 | 6.22E-06 | 13.3 |
| RaGOO_A02_100978 | A02 | 20928712 | 2.24E-09 | 0.159 | 104 | 0.692 | 0.825 | 6.25E-06 | 13.3 |
| RaGOO_A02_102673 | A02 | 21150786 | 2.24E-09 | 0.168 | 104 | 0.692 | 0.825 | 6.25E-06 | 13.3 |
| RaGOO_A02_100189 | A02 | 20711963 | 2.25E-09 | 0.163 | 104 | 0.692 | 0.825 | 6.25E-06 | 13.3 |
| RaGOO_A02_73197 | A02 | 15740915 | 2.26E-09 | 0.154 | 104 | 0.692 | 0.825 | 6.28E-06 | 13.3 |
| RaGOO_A02_112979 | A02 | 24014705 | 2.26E-09 | 0.168 | 104 | 0.692 | 0.825 | 6.28E-06 | 13.3 |
| RaGOO_A02_118297 | A02 | 24878853 | 2.27E-09 | 0.168 | 104 | 0.692 | 0.825 | 6.29E-06 | 13.3 |
| RaGOO_A02_101914 | A02 | 21040641 | 2.27E-09 | 0.178 | 104 | 0.692 | 0.825 | 6.29E-06 | 13.3 |
| RaGOO_A02_116299 | A02 | 24568255 | 2.27E-09 | 0.159 | 104 | 0.692 | 0.825 | 6.29E-06 | 13.3 |
| RaGOO_A02_118227 | A02 | 24865929 | 2.28E-09 | 0.163 | 104 | 0.692 | 0.825 | 6.30E-06 | 13.3 |
| RaGOO_A02_115326 | A02 | 24450140 | 2.28E-09 | 0.168 | 104 | 0.692 | 0.825 | 6.30E-06 | 13.3 |
| RaGOO_A02_97451 | A02 | 20165758 | 2.28E-09 | 0.149 | 104 | 0.692 | 0.825 | 6.30E-06 | 13.3 |
| RaGOO_A02_101154 | A02 | 20945539 | 2.28E-09 | 0.173 | 104 | 0.692 | 0.825 | 6.31E-06 | 13.3 |
| RaGOO_A02_100401 | A02 | 20796320 | 2.30E-09 | 0.154 | 104 | 0.692 | 0.825 | 6.35E-06 | 13.3 |
| RaGOO_A02_73203 | A02 | 15741447 | 2.31E-09 | 0.154 | 104 | 0.692 | 0.825 | 6.36E-06 | 13.3 |
| RaGOO_A02_99157 | A02 | 20486080 | 2.31E-09 | 0.178 | 104 | 0.692 | 0.825 | 6.36E-06 | 13.3 |
| RaGOO_A02_115929 | A02 | 24535614 | 2.32E-09 | 0.168 | 104 | 0.692 | 0.825 | 6.37E-06 | 13.3 |
| RaGOO_A02_117507 | A02 | 24750861 | 2.32E-09 | 0.154 | 104 | 0.692 | 0.825 | 6.38E-06 | 13.3 |
| RaGOO_A02_99669 | A02 | 20573526 | 2.32E-09 | 0.163 | 104 | 0.692 | 0.825 | 6.38E-06 | 13.3 |
| RaGOO_A02_114128 | A02 | 24267847 | 2.33E-09 | 0.159 | 104 | 0.692 | 0.825 | 6.40E-06 | 13.3 |
| RaGOO_A02_99805 | A02 | 20609093 | 2.34E-09 | 0.159 | 104 | 0.692 | 0.825 | 6.40E-06 | 13.3 |
| RaGOO_A02_88686 | A02 | 18273574 | 2.34E-09 | 0.163 | 104 | 0.692 | 0.825 | 6.40E-06 | 13.3 |
| RaGOO_A02_98954 | A02 | 20469584 | 2.34E-09 | 0.159 | 104 | 0.692 | 0.825 | 6.41E-06 | 13.3 |
| RaGOO_A02_89558 | A02 | 18529039 | 2.35E-09 | 0.159 | 104 | 0.692 | 0.825 | 6.44E-06 | 13.3 |
| RaGOO_A02_106108 | A02 | 21926468 | 2.37E-09 | 0.154 | 104 | 0.692 | 0.825 | 6.48E-06 | 13.3 |
| RaGOO_A02_73053 | A02 | 15722863 | 2.39E-09 | 0.149 | 104 | 0.692 | 0.825 | 6.52E-06 | 13.3 |
| RaGOO_A02_118196 | A02 | 24863045 | 2.39E-09 | 0.159 | 104 | 0.692 | 0.825 | 6.52E-06 | 13.3 |
| RaGOO_A02_83547 | A02 | 17351030 | 2.40E-09 | 0.178 | 104 | 0.692 | 0.825 | 6.53E-06 | 13.3 |
| RaGOO_A02_110804 | A02 | 22882923 | 2.40E-09 | 0.154 | 104 | 0.692 | 0.825 | 6.53E-06 | 13.3 |
| RaGOO_A02_91665 | A02 | 18859914 | 2.41E-09 | 0.144 | 104 | 0.692 | 0.825 | 6.54E-06 | 13.3 |
| RaGOO_A02_115853 | A02 | 24522396 | 2.41E-09 | 0.168 | 104 | 0.692 | 0.825 | 6.55E-06 | 13.3 |
| RaGOO_A02_98924 | A02 | 20465590 | 2.41E-09 | 0.173 | 104 | 0.692 | 0.825 | 6.55E-06 | 13.3 |
| RaGOO_A02_107950 | A02 | 22201703 | 2.42E-09 | 0.139 | 104 | 0.692 | 0.825 | 6.56E-06 | 13.3 |
| RaGOO_A02_107951 | A02 | 22201711 | 2.42E-09 | 0.139 | 104 | 0.692 | 0.825 | 6.56E-06 | 13.3 |
| RaGOO_A02_97107 | A02 | 20105975 | 2.42E-09 | 0.163 | 104 | 0.692 | 0.825 | 6.56E-06 | 13.3 |
| RaGOO_A02_101876 | A02 | 21037170 | 2.42E-09 | 0.168 | 104 | 0.692 | 0.825 | 6.56E-06 | 13.3 |
| RaGOO_A02_101911 | A02 | 21040411 | 2.44E-09 | 0.178 | 104 | 0.692 | 0.825 | 6.60E-06 | 13.3 |
| RaGOO_A02_97805 | A02 | 20217164 | 2.44E-09 | 0.173 | 104 | 0.692 | 0.825 | 6.60E-06 | 13.3 |
| RaGOO_A02_100388 | A02 | 20792381 | 2.45E-09 | 0.159 | 104 | 0.692 | 0.825 | 6.61E-06 | 13.3 |
| RaGOO_A02_80505 | A02 | 16954197 | 2.45E-09 | 0.115 | 104 | 0.692 | 0.825 | 6.61E-06 | 13.3 |
| RaGOO_A02_115499 | A02 | 24474325 | 2.45E-09 | 0.159 | 104 | 0.692 | 0.825 | 6.61E-06 | 13.3 |
| RaGOO_A02_104804 | A02 | 21665742 | 2.46E-09 | 0.154 | 104 | 0.692 | 0.825 | 6.64E-06 | 13.3 |
| RaGOO_A02_84471 | A02 | 17503947 | 2.47E-09 | 0.168 | 104 | 0.692 | 0.825 | 6.65E-06 | 13.3 |
| RaGOO_A02_79724 | A02 | 16868215 | 2.48E-09 | 0.163 | 104 | 0.692 | 0.825 | 6.65E-06 | 13.3 |
| RaGOO_A02_103432 | A02 | 21341927 | 2.48E-09 | 0.125 | 104 | 0.692 | 0.825 | 6.65E-06 | 13.3 |
| RaGOO_A02_98056 | A02 | 20256618 | 2.51E-09 | 0.168 | 104 | 0.692 | 0.824 | 6.74E-06 | 13.2 |
| RaGOO_A02_116319 | A02 | 24569725 | 2.54E-09 | 0.159 | 104 | 0.692 | 0.824 | 6.81E-06 | 13.2 |
| RaGOO_A02_111503 | A02 | 23138186 | 2.56E-09 | 0.154 | 104 | 0.692 | 0.824 | 6.85E-06 | 13.2 |
| RaGOO_A02_97450 | A02 | 20165746 | 2.56E-09 | 0.149 | 104 | 0.692 | 0.824 | 6.85E-06 | 13.2 |
| RaGOO_A02_111113 | A02 | 22934133 | 2.57E-09 | 0.154 | 104 | 0.692 | 0.824 | 6.85E-06 | 13.2 |
| RaGOO_A02_111114 | A02 | 22934134 | 2.57E-09 | 0.154 | 104 | 0.692 | 0.824 | 6.85E-06 | 13.2 |
| RaGOO_A02_89298 | A02 | 18473669 | 2.58E-09 | 0.149 | 104 | 0.692 | 0.824 | 6.87E-06 | 13.2 |
| RaGOO_A02_88051 | A02 | 18191605 | 2.58E-09 | 0.159 | 104 | 0.692 | 0.824 | 6.87E-06 | 13.2 |
| RaGOO_A02_88052 | A02 | 18191610 | 2.58E-09 | 0.159 | 104 | 0.692 | 0.824 | 6.87E-06 | 13.2 |
| RaGOO_A02_88053 | A02 | 18191640 | 2.58E-09 | 0.159 | 104 | 0.692 | 0.824 | 6.87E-06 | 13.2 |
| RaGOO_A02_98819 | A02 | 20449952 | 2.58E-09 | 0.154 | 104 | 0.692 | 0.824 | 6.87E-06 | 13.2 |
| RaGOO_A02_71955 | A02 | 15483722 | 2.62E-09 | 0.159 | 104 | 0.692 | 0.824 | 6.97E-06 | 13.2 |
| RaGOO_A02_118228 | A02 | 24865947 | 2.64E-09 | 0.173 | 104 | 0.692 | 0.824 | 7.01E-06 | 13.2 |
| RaGOO_A02_87807 | A02 | 18143815 | 2.64E-09 | 0.183 | 104 | 0.692 | 0.824 | 7.01E-06 | 13.2 |
| RaGOO_A02_107019 | A02 | 22098547 | 2.66E-09 | 0.125 | 104 | 0.692 | 0.824 | 7.04E-06 | 13.2 |
| RaGOO_A02_88540 | A02 | 18261026 | 2.68E-09 | 0.149 | 104 | 0.692 | 0.824 | 7.09E-06 | 13.2 |
| RaGOO_A02_110302 | A02 | 22791011 | 2.68E-09 | 0.154 | 104 | 0.692 | 0.824 | 7.09E-06 | 13.2 |
| RaGOO_A02_107576 | A02 | 22173025 | 2.69E-09 | 0.125 | 104 | 0.692 | 0.824 | 7.09E-06 | 13.2 |
| RaGOO_A02_114975 | A02 | 24408441 | 2.69E-09 | 0.120 | 104 | 0.692 | 0.824 | 7.09E-06 | 13.2 |
| RaGOO_A02_102844 | A02 | 21170194 | 2.72E-09 | 0.163 | 104 | 0.692 | 0.824 | 7.18E-06 | 13.2 |
| RaGOO_A02_102845 | A02 | 21170200 | 2.72E-09 | 0.163 | 104 | 0.692 | 0.824 | 7.18E-06 | 13.2 |
| RaGOO_A02_115826 | A02 | 24519855 | 2.73E-09 | 0.149 | 104 | 0.692 | 0.824 | 7.19E-06 | 13.2 |
| RaGOO_A02_99673 | A02 | 20574486 | 2.74E-09 | 0.159 | 104 | 0.692 | 0.824 | 7.21E-06 | 13.2 |
| RaGOO_A02_105431 | A02 | 21821288 | 2.75E-09 | 0.159 | 104 | 0.692 | 0.824 | 7.22E-06 | 13.2 |
| RaGOO_A02_115481 | A02 | 24472356 | 2.75E-09 | 0.163 | 104 | 0.692 | 0.824 | 7.22E-06 | 13.2 |
| RaGOO_A02_117503 | A02 | 24749811 | 2.75E-09 | 0.183 | 104 | 0.692 | 0.824 | 7.22E-06 | 13.2 |
| RaGOO_A02_80156 | A02 | 16918896 | 2.75E-09 | 0.149 | 104 | 0.692 | 0.824 | 7.22E-06 | 13.2 |
| RaGOO_A02_102654 | A02 | 21148217 | 2.76E-09 | 0.149 | 104 | 0.692 | 0.824 | 7.22E-06 | 13.2 |
| RaGOO_A02_117454 | A02 | 24740457 | 2.76E-09 | 0.163 | 104 | 0.692 | 0.824 | 7.22E-06 | 13.2 |
| RaGOO_A02_105564 | A02 | 21845339 | 2.78E-09 | 0.154 | 104 | 0.692 | 0.824 | 7.27E-06 | 13.2 |
| RaGOO_A02_84488 | A02 | 17505790 | 2.80E-09 | 0.168 | 104 | 0.692 | 0.823 | 7.31E-06 | 13.1 |
| RaGOO_A02_105563 | A02 | 21845151 | 2.81E-09 | 0.149 | 104 | 0.692 | 0.823 | 7.33E-06 | 13.1 |
| RaGOO_A02_99716 | A02 | 20583335 | 2.83E-09 | 0.168 | 104 | 0.692 | 0.823 | 7.39E-06 | 13.1 |
| RaGOO_A02_113882 | A02 | 24234396 | 2.85E-09 | 0.163 | 104 | 0.692 | 0.823 | 7.43E-06 | 13.1 |
| RaGOO_A02_117729 | A02 | 24802247 | 2.87E-09 | 0.154 | 104 | 0.692 | 0.823 | 7.47E-06 | 13.1 |
| RaGOO_A02_91803 | A02 | 18874227 | 2.87E-09 | 0.168 | 104 | 0.692 | 0.823 | 7.48E-06 | 13.1 |
| RaGOO_A02_86739 | A02 | 17933933 | 2.88E-09 | 0.163 | 104 | 0.692 | 0.823 | 7.49E-06 | 13.1 |
| RaGOO_A02_111072 | A02 | 22919753 | 2.88E-09 | 0.159 | 104 | 0.692 | 0.823 | 7.49E-06 | 13.1 |
| RaGOO_A02_88936 | A02 | 18301430 | 2.89E-09 | 0.115 | 104 | 0.692 | 0.823 | 7.50E-06 | 13.1 |
| RaGOO_A02_104279 | A02 | 21558612 | 2.89E-09 | 0.163 | 104 | 0.692 | 0.823 | 7.51E-06 | 13.1 |
| RaGOO_A02_83495 | A02 | 17345977 | 2.91E-09 | 0.139 | 104 | 0.692 | 0.823 | 7.52E-06 | 13.1 |
| RaGOO_A02_83494 | A02 | 17345975 | 2.91E-09 | 0.139 | 104 | 0.692 | 0.823 | 7.52E-06 | 13.1 |

| RaGOO_A02_91702 | A02 | 18861687 | 2.91E-09 | 0.183 | 104 | 0.692 | 0.823 | 7.52E-06 | 13.1 |
| --- | --- | --- | --- | --- | --- | --- | --- | --- | --- |
| RaGOO_A02_91457 | A02 | 18844864 | 2.91E-09 | 0.159 | 104 | 0.692 | 0.823 | 7.54E-06 | 13.1 |
| RaGOO_A02_110828 | A02 | 22886334 | 2.92E-09 | 0.168 | 104 | 0.692 | 0.823 | 7.54E-06 | 13.1 |
| RaGOO_A02_116653 | A02 | 24600941 | 2.92E-09 | 0.168 | 104 | 0.692 | 0.823 | 7.55E-06 | 13.1 |
| RaGOO_A02_99014 | A02 | 20474597 | 2.93E-09 | 0.168 | 104 | 0.692 | 0.823 | 7.55E-06 | 13.1 |
| RaGOO_A02_92448 | A02 | 18936061 | 2.93E-09 | 0.159 | 104 | 0.692 | 0.823 | 7.56E-06 | 13.1 |
| RaGOO_A02_102337 | A02 | 21110282 | 2.96E-09 | 0.163 | 104 | 0.692 | 0.823 | 7.61E-06 | 13.1 |
| RaGOO_A02_84705 | A02 | 17531804 | 2.97E-09 | 0.159 | 104 | 0.692 | 0.823 | 7.64E-06 | 13.1 |
| RaGOO_A02_100490 | A02 | 20825872 | 2.97E-09 | 0.154 | 104 | 0.692 | 0.823 | 7.64E-06 | 13.1 |
| RaGOO_A02_100491 | A02 | 20825887 | 2.97E-09 | 0.154 | 104 | 0.692 | 0.823 | 7.64E-06 | 13.1 |
| RaGOO_A02_110262 | A02 | 22786322 | 3.01E-09 | 0.154 | 104 | 0.692 | 0.823 | 7.72E-06 | 13.1 |
| RaGOO_A02_101898 | A02 | 21039402 | 3.02E-09 | 0.154 | 104 | 0.692 | 0.823 | 7.74E-06 | 13.1 |
| RaGOO_A02_82391 | A02 | 17225454 | 3.05E-09 | 0.168 | 104 | 0.692 | 0.823 | 7.82E-06 | 13.1 |
| RaGOO_A02_113908 | A02 | 24235133 | 3.06E-09 | 0.159 | 104 | 0.692 | 0.823 | 7.82E-06 | 13.1 |
| RaGOO_A02_123299 | A02 | 25697226 | 3.06E-09 | 0.139 | 104 | 0.692 | 0.823 | 7.82E-06 | 13.1 |
| RaGOO_A02_100129 | A02 | 20698048 | 3.06E-09 | 0.163 | 104 | 0.692 | 0.823 | 7.82E-06 | 13.1 |
| RaGOO_A02_98047 | A02 | 20256047 | 3.07E-09 | 0.173 | 104 | 0.692 | 0.823 | 7.82E-06 | 13.1 |
| RaGOO_A02_84708 | A02 | 17531851 | 3.07E-09 | 0.163 | 104 | 0.692 | 0.823 | 7.82E-06 | 13.1 |
| RaGOO_A02_75614 | A02 | 16195008 | 3.08E-09 | 0.163 | 104 | 0.692 | 0.823 | 7.84E-06 | 13.1 |
| RaGOO_A02_86930 | A02 | 17983308 | 3.08E-09 | 0.173 | 104 | 0.692 | 0.823 | 7.84E-06 | 13.1 |
| RaGOO_A02_100984 | A02 | 20929560 | 3.08E-09 | 0.173 | 104 | 0.692 | 0.823 | 7.84E-06 | 13.1 |
| RaGOO_A02_110974 | A02 | 22913655 | 3.09E-09 | 0.168 | 104 | 0.692 | 0.823 | 7.84E-06 | 13.1 |
| RaGOO_A02_82292 | A02 | 17219750 | 3.10E-09 | 0.159 | 104 | 0.692 | 0.823 | 7.87E-06 | 13.1 |
| RaGOO_A02_114640 | A02 | 24366366 | 3.10E-09 | 0.154 | 104 | 0.692 | 0.823 | 7.88E-06 | 13.1 |
| RaGOO_A02_99046 | A02 | 20476546 | 3.11E-09 | 0.178 | 104 | 0.692 | 0.823 | 7.89E-06 | 13.1 |
| RaGOO_A02_75273 | A02 | 16172084 | 3.11E-09 | 0.159 | 104 | 0.692 | 0.823 | 7.89E-06 | 13.1 |
| RaGOO_A02_76752 | A02 | 16331437 | 3.13E-09 | 0.163 | 104 | 0.692 | 0.823 | 7.91E-06 | 13.1 |
| RaGOO_A02_91392 | A02 | 18824584 | 3.15E-09 | 0.159 | 104 | 0.692 | 0.822 | 7.97E-06 | 13.0 |
| RaGOO_A02_80136 | A02 | 16917373 | 3.15E-09 | 0.144 | 104 | 0.692 | 0.822 | 7.97E-06 | 13.0 |
| RaGOO_A02_111115 | A02 | 22934142 | 3.16E-09 | 0.163 | 104 | 0.692 | 0.822 | 7.98E-06 | 13.0 |
| RaGOO_A02_108927 | A02 | 22544647 | 3.17E-09 | 0.154 | 104 | 0.692 | 0.822 | 7.98E-06 | 13.0 |
| RaGOO_A02_107499 | A02 | 22170380 | 3.17E-09 | 0.120 | 104 | 0.692 | 0.822 | 7.98E-06 | 13.0 |
| RaGOO_A02_89941 | A02 | 18581015 | 3.17E-09 | 0.149 | 104 | 0.692 | 0.822 | 7.98E-06 | 13.0 |
| RaGOO_A02_89690 | A02 | 18547639 | 3.17E-09 | 0.149 | 104 | 0.692 | 0.822 | 7.98E-06 | 13.0 |
| RaGOO_A02_84473 | A02 | 17504127 | 3.19E-09 | 0.159 | 104 | 0.692 | 0.822 | 8.02E-06 | 13.0 |
| RaGOO_A02_73179 | A02 | 15739584 | 3.21E-09 | 0.159 | 104 | 0.692 | 0.822 | 8.06E-06 | 13.0 |
| RaGOO_A02_115884 | A02 | 24530140 | 3.21E-09 | 0.149 | 104 | 0.692 | 0.822 | 8.06E-06 | 13.0 |
| RaGOO_A02_115831 | A02 | 24520175 | 3.23E-09 | 0.154 | 104 | 0.692 | 0.822 | 8.09E-06 | 13.0 |
| RaGOO_A02_105938 | A02 | 21909564 | 3.23E-09 | 0.159 | 104 | 0.692 | 0.822 | 8.09E-06 | 13.0 |
| RaGOO_A02_100279 | A02 | 20755656 | 3.23E-09 | 0.163 | 104 | 0.692 | 0.822 | 8.09E-06 | 13.0 |
| RaGOO_A02_89629 | A02 | 18539648 | 3.24E-09 | 0.168 | 104 | 0.692 | 0.822 | 8.09E-06 | 13.0 |
| RaGOO_A02_111225 | A02 | 22947879 | 3.24E-09 | 0.154 | 104 | 0.692 | 0.822 | 8.09E-06 | 13.0 |
| RaGOO_A02_99876 | A02 | 20627243 | 3.24E-09 | 0.106 | 104 | 0.692 | 0.822 | 8.09E-06 | 13.0 |
| RaGOO_A02_119587 | A02 | 25153217 | 3.24E-09 | 0.173 | 104 | 0.692 | 0.822 | 8.09E-06 | 13.0 |
| RaGOO_A02_75274 | A02 | 16172176 | 3.25E-09 | 0.163 | 104 | 0.692 | 0.822 | 8.11E-06 | 13.0 |
| RaGOO_A02_101910 | A02 | 21040368 | 3.26E-09 | 0.178 | 104 | 0.692 | 0.822 | 8.12E-06 | 13.0 |
| RaGOO_A02_108571 | A02 | 22422741 | 3.27E-09 | 0.168 | 104 | 0.692 | 0.822 | 8.14E-06 | 13.0 |
| RaGOO_A02_91722 | A02 | 18863274 | 3.27E-09 | 0.168 | 104 | 0.692 | 0.822 | 8.14E-06 | 13.0 |
| RaGOO_A02_91868 | A02 | 18878319 | 3.28E-09 | 0.168 | 104 | 0.692 | 0.822 | 8.14E-06 | 13.0 |
| RaGOO_A02_86935 | A02 | 17983663 | 3.28E-09 | 0.173 | 104 | 0.692 | 0.822 | 8.14E-06 | 13.0 |
| RaGOO_A02_86934 | A02 | 17983662 | 3.28E-09 | 0.173 | 104 | 0.692 | 0.822 | 8.14E-06 | 13.0 |
| RaGOO_A02_87720 | A02 | 18137524 | 3.31E-09 | 0.111 | 104 | 0.692 | 0.822 | 8.20E-06 | 13.0 |
| RaGOO_A02_114588 | A02 | 24362715 | 3.35E-09 | 0.192 | 104 | 0.692 | 0.822 | 8.30E-06 | 13.0 |
| RaGOO_A02_120774 | A02 | 25408519 | 3.37E-09 | 0.173 | 104 | 0.692 | 0.822 | 8.35E-06 | 13.0 |
| RaGOO_A02_73275 | A02 | 15757737 | 3.37E-09 | 0.159 | 104 | 0.692 | 0.822 | 8.35E-06 | 13.0 |
| RaGOO_A02_80281 | A02 | 16932966 | 3.37E-09 | 0.173 | 104 | 0.692 | 0.822 | 8.35E-06 | 13.0 |
| RaGOO_A02_112384 | A02 | 23925567 | 3.38E-09 | 0.139 | 104 | 0.692 | 0.822 | 8.35E-06 | 13.0 |
| RaGOO_A02_92310 | A02 | 18919403 | 3.38E-09 | 0.163 | 104 | 0.692 | 0.822 | 8.35E-06 | 13.0 |
| RaGOO_A02_91063 | A02 | 18754637 | 3.39E-09 | 0.159 | 104 | 0.692 | 0.822 | 8.36E-06 | 13.0 |
| RaGOO_A02_114494 | A02 | 24332182 | 3.39E-09 | 0.154 | 104 | 0.692 | 0.822 | 8.36E-06 | 13.0 |
| RaGOO_A02_79837 | A02 | 16880927 | 3.40E-09 | 0.159 | 104 | 0.692 | 0.822 | 8.36E-06 | 13.0 |
| RaGOO_A02_107667 | A02 | 22175850 | 3.40E-09 | 0.125 | 104 | 0.692 | 0.822 | 8.36E-06 | 13.0 |
| RaGOO_A02_106737 | A02 | 22007831 | 3.41E-09 | 0.149 | 104 | 0.692 | 0.822 | 8.40E-06 | 13.0 |
| RaGOO_A02_85394 | A02 | 17682688 | 3.43E-09 | 0.154 | 104 | 0.692 | 0.822 | 8.42E-06 | 13.0 |
| RaGOO_A02_116351 | A02 | 24571810 | 3.44E-09 | 0.159 | 104 | 0.692 | 0.822 | 8.44E-06 | 13.0 |
| RaGOO_A02_98044 | A02 | 20255579 | 3.44E-09 | 0.168 | 104 | 0.692 | 0.822 | 8.45E-06 | 13.0 |
| RaGOO_A02_102574 | A02 | 21141467 | 3.46E-09 | 0.178 | 104 | 0.692 | 0.822 | 8.49E-06 | 13.0 |
| RaGOO_A02_102960 | A02 | 21209412 | 3.46E-09 | 0.178 | 104 | 0.692 | 0.822 | 8.49E-06 | 13.0 |
| RaGOO_A02_103192 | A02 | 21293284 | 3.47E-09 | 0.163 | 104 | 0.692 | 0.822 | 8.50E-06 | 13.0 |
| RaGOO_A02_105449 | A02 | 21825076 | 3.47E-09 | 0.159 | 104 | 0.692 | 0.822 | 8.50E-06 | 13.0 |
| RaGOO_A02_102483 | A02 | 21129210 | 3.48E-09 | 0.163 | 104 | 0.692 | 0.822 | 8.52E-06 | 13.0 |
| RaGOO_A02_101057 | A02 | 20936128 | 3.50E-09 | 0.173 | 104 | 0.692 | 0.822 | 8.56E-06 | 13.0 |
| RaGOO_A02_124787 | A02 | 26013559 | 3.53E-09 | 0.216 | 104 | 0.692 | 0.822 | 8.60E-06 | 12.9 |
| RaGOO_A02_103823 | A02 | 21495969 | 3.53E-09 | 0.173 | 104 | 0.692 | 0.822 | 8.60E-06 | 12.9 |
| RaGOO_A02_89397 | A02 | 18498531 | 3.53E-09 | 0.149 | 104 | 0.692 | 0.821 | 8.60E-06 | 12.9 |
| RaGOO_A02_82226 | A02 | 17216622 | 3.54E-09 | 0.163 | 104 | 0.692 | 0.821 | 8.61E-06 | 12.9 |
| RaGOO_A02_82225 | A02 | 17216612 | 3.54E-09 | 0.163 | 104 | 0.692 | 0.821 | 8.61E-06 | 12.9 |
| RaGOO_A02_99664 | A02 | 20572988 | 3.54E-09 | 0.154 | 104 | 0.692 | 0.821 | 8.62E-06 | 12.9 |
| RaGOO_A02_100602 | A02 | 20843124 | 3.56E-09 | 0.154 | 104 | 0.692 | 0.821 | 8.65E-06 | 12.9 |
| RaGOO_A02_100962 | A02 | 20927543 | 3.57E-09 | 0.168 | 104 | 0.692 | 0.821 | 8.66E-06 | 12.9 |
| RaGOO_A02_89630 | A02 | 18539715 | 3.57E-09 | 0.168 | 104 | 0.692 | 0.821 | 8.66E-06 | 12.9 |
| RaGOO_A02_116352 | A02 | 24571822 | 3.57E-09 | 0.159 | 104 | 0.692 | 0.821 | 8.66E-06 | 12.9 |
| RaGOO_A02_115317 | A02 | 24444139 | 3.57E-09 | 0.188 | 104 | 0.692 | 0.821 | 8.66E-06 | 12.9 |
| RaGOO_A02_80842 | A02 | 16980081 | 3.58E-09 | 0.149 | 104 | 0.692 | 0.821 | 8.67E-06 | 12.9 |
| RaGOO_A02_103066 | A02 | 21226458 | 3.58E-09 | 0.163 | 104 | 0.692 | 0.821 | 8.67E-06 | 12.9 |
| RaGOO_A02_84024 | A02 | 17421619 | 3.59E-09 | 0.130 | 104 | 0.692 | 0.821 | 8.67E-06 | 12.9 |
| RaGOO_A02_118630 | A02 | 24956447 | 3.59E-09 | 0.159 | 104 | 0.692 | 0.821 | 8.67E-06 | 12.9 |
| RaGOO_A02_115815 | A02 | 24519275 | 3.60E-09 | 0.149 | 104 | 0.692 | 0.821 | 8.70E-06 | 12.9 |
| RaGOO_A02_103028 | A02 | 21222308 | 3.61E-09 | 0.154 | 104 | 0.692 | 0.821 | 8.71E-06 | 12.9 |
| RaGOO_A02_103014 | A02 | 21220399 | 3.62E-09 | 0.154 | 104 | 0.692 | 0.821 | 8.72E-06 | 12.9 |
| RaGOO_A02_90341 | A02 | 18639475 | 3.64E-09 | 0.163 | 104 | 0.692 | 0.821 | 8.76E-06 | 12.9 |

| RaGOO_A02_77186 | A02 | 16447615 | 3.64E-09 | 0.159 | 104 | 0.692 | 0.821 | 8.76E-06 | 12.9 |
| --- | --- | --- | --- | --- | --- | --- | --- | --- | --- |
| RaGOO_A02_103841 | A02 | 21496996 | 3.64E-09 | 0.159 | 104 | 0.692 | 0.821 | 8.76E-06 | 12.9 |
| RaGOO_A02_88143 | A02 | 18197599 | 3.65E-09 | 0.154 | 104 | 0.692 | 0.821 | 8.76E-06 | 12.9 |
| RaGOO_A02_104254 | A02 | 21554637 | 3.66E-09 | 0.159 | 104 | 0.692 | 0.821 | 8.78E-06 | 12.9 |
| RaGOO_A02_76997 | A02 | 16416448 | 3.68E-09 | 0.159 | 104 | 0.692 | 0.821 | 8.83E-06 | 12.9 |
| RaGOO_A02_100679 | A02 | 20854431 | 3.68E-09 | 0.168 | 104 | 0.692 | 0.821 | 8.83E-06 | 12.9 |
| RaGOO_A02_112529 | A02 | 23949526 | 3.69E-09 | 0.173 | 104 | 0.692 | 0.821 | 8.83E-06 | 12.9 |
| RaGOO_A02_96056 | A02 | 19953191 | 3.69E-09 | 0.154 | 104 | 0.692 | 0.821 | 8.83E-06 | 12.9 |
| RaGOO_A02_92020 | A02 | 18892692 | 3.69E-09 | 0.154 | 104 | 0.692 | 0.821 | 8.83E-06 | 12.9 |
| RaGOO_A02_102822 | A02 | 21168259 | 3.71E-09 | 0.163 | 104 | 0.692 | 0.821 | 8.87E-06 | 12.9 |
| RaGOO_A02_110749 | A02 | 22877214 | 3.73E-09 | 0.154 | 104 | 0.692 | 0.821 | 8.92E-06 | 12.9 |
| RaGOO_A02_105246 | A02 | 21798832 | 3.74E-09 | 0.159 | 104 | 0.692 | 0.821 | 8.92E-06 | 12.9 |
| RaGOO_A02_73677 | A02 | 15821857 | 3.75E-09 | 0.168 | 104 | 0.692 | 0.821 | 8.94E-06 | 12.9 |
| RaGOO_A02_115855 | A02 | 24522438 | 3.76E-09 | 0.178 | 104 | 0.692 | 0.821 | 8.96E-06 | 12.9 |
| RaGOO_A02_73173 | A02 | 15739093 | 3.76E-09 | 0.149 | 104 | 0.692 | 0.821 | 8.96E-06 | 12.9 |
| RaGOO_A02_83432 | A02 | 17330747 | 3.78E-09 | 0.149 | 104 | 0.692 | 0.821 | 8.97E-06 | 12.9 |
| RaGOO_A02_86923 | A02 | 17982958 | 3.78E-09 | 0.154 | 104 | 0.692 | 0.821 | 8.97E-06 | 12.9 |
| RaGOO_A02_86925 | A02 | 17982961 | 3.78E-09 | 0.154 | 104 | 0.692 | 0.821 | 8.97E-06 | 12.9 |
| RaGOO_A02_115697 | A02 | 24501920 | 3.79E-09 | 0.159 | 104 | 0.692 | 0.821 | 9.01E-06 | 12.9 |
| RaGOO_A02_91605 | A02 | 18856238 | 3.81E-09 | 0.183 | 104 | 0.692 | 0.821 | 9.04E-06 | 12.9 |
| RaGOO_A02_73287 | A02 | 15758898 | 3.81E-09 | 0.149 | 104 | 0.692 | 0.821 | 9.04E-06 | 12.9 |
| RaGOO_A02_104253 | A02 | 21554608 | 3.82E-09 | 0.178 | 104 | 0.692 | 0.821 | 9.04E-06 | 12.9 |
| RaGOO_A02_99647 | A02 | 20570376 | 3.82E-09 | 0.159 | 104 | 0.692 | 0.821 | 9.05E-06 | 12.9 |
| RaGOO_A02_80265 | A02 | 16929488 | 3.83E-09 | 0.168 | 104 | 0.692 | 0.821 | 9.05E-06 | 12.9 |
| RaGOO_A02_77697 | A02 | 16564382 | 3.83E-09 | 0.154 | 104 | 0.692 | 0.821 | 9.05E-06 | 12.9 |
| RaGOO_A02_98116 | A02 | 20272048 | 3.83E-09 | 0.178 | 104 | 0.692 | 0.821 | 9.05E-06 | 12.9 |
| RaGOO_A02_100091 | A02 | 20689435 | 3.83E-09 | 0.154 | 104 | 0.692 | 0.821 | 9.05E-06 | 12.9 |
| RaGOO_A02_89125 | A02 | 18422972 | 3.84E-09 | 0.154 | 104 | 0.692 | 0.821 | 9.06E-06 | 12.9 |
| RaGOO_A02_80093 | A02 | 16913125 | 3.84E-09 | 0.149 | 104 | 0.692 | 0.821 | 9.06E-06 | 12.9 |
| RaGOO_A02_89813 | A02 | 18561574 | 3.85E-09 | 0.183 | 104 | 0.692 | 0.821 | 9.08E-06 | 12.9 |
| RaGOO_A02_97370 | A02 | 20153422 | 3.86E-09 | 0.149 | 104 | 0.692 | 0.821 | 9.09E-06 | 12.9 |
| RaGOO_A02_115840 | A02 | 24520916 | 3.88E-09 | 0.173 | 104 | 0.692 | 0.821 | 9.13E-06 | 12.9 |
| RaGOO_A02_97194 | A02 | 20129014 | 3.89E-09 | 0.149 | 104 | 0.692 | 0.821 | 9.13E-06 | 12.9 |
| RaGOO_A02_116068 | A02 | 24547055 | 3.89E-09 | 0.159 | 104 | 0.692 | 0.821 | 9.13E-06 | 12.9 |
| RaGOO_A02_119582 | A02 | 25152924 | 3.89E-09 | 0.163 | 104 | 0.692 | 0.821 | 9.13E-06 | 12.9 |
| RaGOO_A02_102555 | A02 | 21140280 | 3.89E-09 | 0.168 | 104 | 0.692 | 0.821 | 9.13E-06 | 12.9 |
| RaGOO_A02_97811 | A02 | 20217572 | 3.90E-09 | 0.308 | 104 | 0.692 | 0.821 | 9.13E-06 | 12.9 |
| RaGOO_A02_97180 | A02 | 20121266 | 3.90E-09 | 0.159 | 104 | 0.692 | 0.821 | 9.13E-06 | 12.9 |
| RaGOO_A02_89526 | A02 | 18524283 | 3.91E-09 | 0.149 | 104 | 0.692 | 0.821 | 9.14E-06 | 12.9 |
| RaGOO_A02_117728 | A02 | 24802219 | 3.92E-09 | 0.173 | 104 | 0.692 | 0.821 | 9.16E-06 | 12.9 |
| RaGOO_A02_99054 | A02 | 20477840 | 3.92E-09 | 0.154 | 104 | 0.692 | 0.821 | 9.17E-06 | 12.9 |
| RaGOO_A02_82389 | A02 | 17225332 | 3.93E-09 | 0.154 | 104 | 0.692 | 0.821 | 9.17E-06 | 12.9 |
| RaGOO_A02_82562 | A02 | 17234603 | 3.94E-09 | 0.168 | 104 | 0.692 | 0.821 | 9.19E-06 | 12.9 |
| RaGOO_A02_73201 | A02 | 15741406 | 3.95E-09 | 0.149 | 104 | 0.692 | 0.821 | 9.20E-06 | 12.9 |
| RaGOO_A02_99665 | A02 | 20573079 | 3.95E-09 | 0.163 | 104 | 0.692 | 0.821 | 9.20E-06 | 12.9 |
| RaGOO_A02_89760 | A02 | 18556059 | 3.95E-09 | 0.183 | 104 | 0.692 | 0.821 | 9.20E-06 | 12.9 |
| RaGOO_A02_89761 | A02 | 18556061 | 3.95E-09 | 0.183 | 104 | 0.692 | 0.821 | 9.20E-06 | 12.9 |
| RaGOO_A02_118426 | A02 | 24909493 | 3.96E-09 | 0.159 | 104 | 0.692 | 0.820 | 9.22E-06 | 12.8 |
| RaGOO_A02_110210 | A02 | 22780451 | 3.97E-09 | 0.154 | 104 | 0.692 | 0.820 | 9.22E-06 | 12.8 |
| RaGOO_A02_113907 | A02 | 24235107 | 3.98E-09 | 0.163 | 104 | 0.692 | 0.820 | 9.24E-06 | 12.8 |
| RaGOO_A02_82383 | A02 | 17224979 | 3.98E-09 | 0.168 | 104 | 0.692 | 0.820 | 9.24E-06 | 12.8 |
| RaGOO_A02_100247 | A02 | 20740414 | 3.99E-09 | 0.168 | 104 | 0.692 | 0.820 | 9.25E-06 | 12.8 |
| RaGOO_A02_105924 | A02 | 21907998 | 4.00E-09 | 0.159 | 104 | 0.692 | 0.820 | 9.25E-06 | 12.8 |
| RaGOO_A02_113238 | A02 | 24059012 | 4.00E-09 | 0.154 | 104 | 0.692 | 0.820 | 9.25E-06 | 12.8 |
| RaGOO_A02_115870 | A02 | 24528881 | 4.01E-09 | 0.159 | 104 | 0.692 | 0.820 | 9.27E-06 | 12.8 |
| RaGOO_A02_72441 | A02 | 15577066 | 4.02E-09 | 0.149 | 104 | 0.692 | 0.820 | 9.29E-06 | 12.8 |
| RaGOO_A02_91917 | A02 | 18882800 | 4.04E-09 | 0.168 | 104 | 0.692 | 0.820 | 9.33E-06 | 12.8 |
| RaGOO_A02_96055 | A02 | 19953127 | 4.05E-09 | 0.159 | 104 | 0.692 | 0.820 | 9.36E-06 | 12.8 |
| RaGOO_A02_109057 | A02 | 22569338 | 4.06E-09 | 0.159 | 104 | 0.692 | 0.820 | 9.37E-06 | 12.8 |
| RaGOO_A02_100398 | A02 | 20795132 | 4.07E-09 | 0.149 | 104 | 0.692 | 0.820 | 9.38E-06 | 12.8 |
| RaGOO_A02_103542 | A02 | 21377535 | 4.08E-09 | 0.159 | 104 | 0.692 | 0.820 | 9.38E-06 | 12.8 |
| RaGOO_A02_104773 | A02 | 21646882 | 4.08E-09 | 0.163 | 104 | 0.692 | 0.820 | 9.38E-06 | 12.8 |
| RaGOO_A02_104772 | A02 | 21646873 | 4.08E-09 | 0.163 | 104 | 0.692 | 0.820 | 9.38E-06 | 12.8 |
| RaGOO_A02_99050 | A02 | 20477382 | 4.08E-09 | 0.173 | 104 | 0.692 | 0.820 | 9.38E-06 | 12.8 |
| RaGOO_A02_99452 | A02 | 20531889 | 4.09E-09 | 0.159 | 104 | 0.692 | 0.820 | 9.39E-06 | 12.8 |
| RaGOO_A02_114091 | A02 | 24258600 | 4.10E-09 | 0.168 | 104 | 0.692 | 0.820 | 9.42E-06 | 12.8 |
| RaGOO_A02_115813 | A02 | 24519112 | 4.11E-09 | 0.168 | 104 | 0.692 | 0.820 | 9.42E-06 | 12.8 |
| RaGOO_A02_112070 | A02 | 23881652 | 4.12E-09 | 0.154 | 104 | 0.692 | 0.820 | 9.44E-06 | 12.8 |
| RaGOO_A02_80104 | A02 | 16913819 | 4.13E-09 | 0.159 | 104 | 0.692 | 0.820 | 9.47E-06 | 12.8 |
| RaGOO_A02_92427 | A02 | 18934295 | 4.14E-09 | 0.149 | 104 | 0.692 | 0.820 | 9.47E-06 | 12.8 |
| RaGOO_A02_96204 | A02 | 19981495 | 4.14E-09 | 0.173 | 104 | 0.692 | 0.820 | 9.47E-06 | 12.8 |
| RaGOO_A02_89271 | A02 | 18467079 | 4.14E-09 | 0.159 | 104 | 0.692 | 0.820 | 9.47E-06 | 12.8 |
| RaGOO_A02_97179 | A02 | 20121242 | 4.15E-09 | 0.159 | 104 | 0.692 | 0.820 | 9.48E-06 | 12.8 |
| RaGOO_A02_97104 | A02 | 20105846 | 4.16E-09 | 0.168 | 104 | 0.692 | 0.820 | 9.49E-06 | 12.8 |
| RaGOO_A02_100824 | A02 | 20890746 | 4.17E-09 | 0.173 | 104 | 0.692 | 0.820 | 9.51E-06 | 12.8 |
| RaGOO_A02_122339 | A02 | 25569854 | 4.18E-09 | 0.149 | 104 | 0.692 | 0.820 | 9.52E-06 | 12.8 |
| RaGOO_A02_116082 | A02 | 24547867 | 4.18E-09 | 0.163 | 104 | 0.692 | 0.820 | 9.52E-06 | 12.8 |
| RaGOO_A02_92067 | A02 | 18898154 | 4.18E-09 | 0.168 | 104 | 0.692 | 0.820 | 9.52E-06 | 12.8 |
| RaGOO_A02_99304 | A02 | 20506870 | 4.20E-09 | 0.192 | 104 | 0.692 | 0.820 | 9.55E-06 | 12.8 |
| RaGOO_A02_110212 | A02 | 22780505 | 4.20E-09 | 0.154 | 104 | 0.692 | 0.820 | 9.55E-06 | 12.8 |
| RaGOO_A02_80871 | A02 | 16981585 | 4.20E-09 | 0.173 | 104 | 0.692 | 0.820 | 9.55E-06 | 12.8 |
| RaGOO_A02_80137 | A02 | 16917378 | 4.21E-09 | 0.149 | 104 | 0.692 | 0.820 | 9.56E-06 | 12.8 |
| RaGOO_A02_99679 | A02 | 20575731 | 4.22E-09 | 0.163 | 104 | 0.692 | 0.820 | 9.56E-06 | 12.8 |
| RaGOO_A02_102283 | A02 | 21103964 | 4.22E-09 | 0.178 | 104 | 0.692 | 0.820 | 9.56E-06 | 12.8 |
| RaGOO_A02_83555 | A02 | 17352603 | 4.22E-09 | 0.149 | 104 | 0.692 | 0.820 | 9.56E-06 | 12.8 |
| RaGOO_A02_77497 | A02 | 16509571 | 4.23E-09 | 0.159 | 104 | 0.692 | 0.820 | 9.58E-06 | 12.8 |
| RaGOO_A02_87795 | A02 | 18143526 | 4.24E-09 | 0.168 | 104 | 0.692 | 0.820 | 9.60E-06 | 12.8 |
| RaGOO_A02_110427 | A02 | 22814623 | 4.27E-09 | 0.144 | 104 | 0.692 | 0.820 | 9.65E-06 | 12.8 |
| RaGOO_A02_83700 | A02 | 17385625 | 4.27E-09 | 0.135 | 104 | 0.692 | 0.820 | 9.65E-06 | 12.8 |
| RaGOO_A02_77453 | A02 | 16500643 | 4.28E-09 | 0.159 | 104 | 0.692 | 0.820 | 9.66E-06 | 12.8 |
| RaGOO_A02_115770 | A02 | 24515646 | 4.29E-09 | 0.149 | 104 | 0.692 | 0.820 | 9.66E-06 | 12.8 |

| RaGOO_A02_72396 | A02 | 15573252 | 4.29E-09 | 0.144 | 104 | 0.692 | 0.820 | 9.66E-06 | 12.8 |
| --- | --- | --- | --- | --- | --- | --- | --- | --- | --- |
| RaGOO_A02_102284 | A02 | 21103965 | 4.31E-09 | 0.173 | 104 | 0.692 | 0.820 | 9.70E-06 | 12.8 |
| RaGOO_A02_77496 | A02 | 16509570 | 4.32E-09 | 0.163 | 104 | 0.692 | 0.820 | 9.73E-06 | 12.8 |
| RaGOO_A02_85785 | A02 | 17733667 | 4.34E-09 | 0.154 | 104 | 0.692 | 0.820 | 9.77E-06 | 12.8 |
| RaGOO_A02_111116 | A02 | 22934166 | 4.36E-09 | 0.168 | 104 | 0.692 | 0.820 | 9.80E-06 | 12.8 |
| RaGOO_A02_103096 | A02 | 21230220 | 4.38E-09 | 0.154 | 104 | 0.692 | 0.820 | 9.84E-06 | 12.8 |
| RaGOO_A02_100859 | A02 | 20893096 | 4.40E-09 | 0.154 | 104 | 0.692 | 0.820 | 9.87E-06 | 12.8 |
| RaGOO_A02_85739 | A02 | 17725766 | 4.40E-09 | 0.149 | 104 | 0.692 | 0.820 | 9.87E-06 | 12.8 |
| RaGOO_A02_115846 | A02 | 24521686 | 4.40E-09 | 0.159 | 104 | 0.692 | 0.820 | 9.87E-06 | 12.8 |
| RaGOO_A02_110426 | A02 | 22814587 | 4.41E-09 | 0.144 | 104 | 0.692 | 0.820 | 9.89E-06 | 12.8 |
| RaGOO_A02_119571 | A02 | 25152421 | 4.43E-09 | 0.159 | 104 | 0.692 | 0.820 | 9.91E-06 | 12.8 |
| RaGOO_A02_87797 | A02 | 18143562 | 4.44E-09 | 0.183 | 104 | 0.692 | 0.820 | 9.91E-06 | 12.8 |
| RaGOO_A02_117694 | A02 | 24798737 | 4.44E-09 | 0.159 | 104 | 0.692 | 0.820 | 9.91E-06 | 12.8 |
| RaGOO_A02_107888 | A02 | 22200095 | 4.45E-09 | 0.120 | 104 | 0.692 | 0.820 | 9.93E-06 | 12.7 |
| RaGOO_A02_82652 | A02 | 17239557 | 4.46E-09 | 0.178 | 104 | 0.692 | 0.819 | 9.95E-06 | 12.7 |
| RaGOO_A02_80663 | A02 | 16964488 | 4.47E-09 | 0.159 | 104 | 0.692 | 0.819 | 9.96E-06 | 12.7 |
| RaGOO_A02_104935 | A02 | 21728110 | 4.47E-09 | 0.159 | 104 | 0.692 | 0.819 | 9.96E-06 | 12.7 |
| RaGOO_A02_96480 | A02 | 20009904 | 4.48E-09 | 0.154 | 104 | 0.692 | 0.819 | 9.96E-06 | 12.7 |
| RaGOO_A02_114643 | A02 | 24366435 | 4.48E-09 | 0.159 | 104 | 0.692 | 0.819 | 9.96E-06 | 12.7 |
| RaGOO_A02_91563 | A02 | 18853568 | 4.48E-09 | 0.183 | 104 | 0.692 | 0.819 | 9.97E-06 | 12.7 |
| RaGOO_A02_108521 | A02 | 22409568 | 4.50E-09 | 0.139 | 104 | 0.692 | 0.819 | 1.00E-05 | 12.7 |
| RaGOO_A02_91919 | A02 | 18882911 | 4.51E-09 | 0.188 | 104 | 0.692 | 0.819 | 1.00E-05 | 12.7 |
| RaGOO_A02_85745 | A02 | 17726441 | 4.52E-09 | 0.159 | 104 | 0.692 | 0.819 | 1.00E-05 | 12.7 |
| RaGOO_A02_99656 | A02 | 20571741 | 4.53E-09 | 0.159 | 104 | 0.692 | 0.819 | 1.00E-05 | 12.7 |
| RaGOO_A02_108384 | A02 | 22389543 | 4.54E-09 | 0.154 | 104 | 0.692 | 0.819 | 1.01E-05 | 12.7 |
| RaGOO_A02_73242 | A02 | 15748269 | 4.57E-09 | 0.154 | 104 | 0.692 | 0.819 | 1.01E-05 | 12.7 |
| RaGOO_A02_79596 | A02 | 16855286 | 4.57E-09 | 0.163 | 104 | 0.692 | 0.819 | 1.01E-05 | 12.7 |
| RaGOO_A02_98088 | A02 | 20267305 | 4.59E-09 | 0.159 | 104 | 0.692 | 0.819 | 1.02E-05 | 12.7 |
| RaGOO_A02_105470 | A02 | 21828450 | 4.59E-09 | 0.159 | 104 | 0.692 | 0.819 | 1.02E-05 | 12.7 |
| RaGOO_A02_88589 | A02 | 18267308 | 4.62E-09 | 0.154 | 104 | 0.692 | 0.819 | 1.02E-05 | 12.7 |
| RaGOO_A02_72800 | A02 | 15647958 | 4.62E-09 | 0.154 | 104 | 0.692 | 0.819 | 1.02E-05 | 12.7 |
| RaGOO_A02_75271 | A02 | 16172065 | 4.63E-09 | 0.159 | 104 | 0.692 | 0.819 | 1.02E-05 | 12.7 |
| RaGOO_A02_75272 | A02 | 16172066 | 4.63E-09 | 0.159 | 104 | 0.692 | 0.819 | 1.02E-05 | 12.7 |
| RaGOO_A02_110903 | A02 | 22902136 | 4.64E-09 | 0.159 | 104 | 0.692 | 0.819 | 1.02E-05 | 12.7 |
| RaGOO_A02_90895 | A02 | 18718464 | 4.65E-09 | 0.154 | 104 | 0.692 | 0.819 | 1.02E-05 | 12.7 |
| RaGOO_A02_109353 | A02 | 22607420 | 4.65E-09 | 0.168 | 104 | 0.692 | 0.819 | 1.02E-05 | 12.7 |
| RaGOO_A02_121590 | A02 | 25508963 | 4.65E-09 | 0.288 | 104 | 0.692 | 0.819 | 1.02E-05 | 12.7 |
| RaGOO_A02_118416 | A02 | 24909099 | 4.66E-09 | 0.163 | 104 | 0.692 | 0.819 | 1.02E-05 | 12.7 |
| RaGOO_A02_108148 | A02 | 22337806 | 4.66E-09 | 0.159 | 104 | 0.692 | 0.819 | 1.02E-05 | 12.7 |
| RaGOO_A02_101873 | A02 | 21036976 | 4.66E-09 | 0.168 | 104 | 0.692 | 0.819 | 1.02E-05 | 12.7 |
| RaGOO_A02_89719 | A02 | 18552161 | 4.67E-09 | 0.159 | 104 | 0.692 | 0.819 | 1.02E-05 | 12.7 |
| RaGOO_A02_91219 | A02 | 18766238 | 4.67E-09 | 0.159 | 104 | 0.692 | 0.819 | 1.02E-05 | 12.7 |
| RaGOO_A02_105942 | A02 | 21910019 | 4.69E-09 | 0.159 | 104 | 0.692 | 0.819 | 1.03E-05 | 12.7 |
| RaGOO_A02_99021 | A02 | 20475285 | 4.71E-09 | 0.163 | 104 | 0.692 | 0.819 | 1.03E-05 | 12.7 |
| RaGOO_A02_73206 | A02 | 15741481 | 4.71E-09 | 0.149 | 104 | 0.692 | 0.819 | 1.03E-05 | 12.7 |
| RaGOO_A02_97071 | A02 | 20102228 | 4.72E-09 | 0.163 | 104 | 0.692 | 0.819 | 1.03E-05 | 12.7 |
| RaGOO_A02_91168 | A02 | 18763328 | 4.73E-09 | 0.149 | 104 | 0.692 | 0.819 | 1.03E-05 | 12.7 |
| RaGOO_A02_118854 | A02 | 24983670 | 4.73E-09 | 0.168 | 104 | 0.692 | 0.819 | 1.03E-05 | 12.7 |
| RaGOO_A02_82678 | A02 | 17242939 | 4.74E-09 | 0.168 | 104 | 0.692 | 0.819 | 1.03E-05 | 12.7 |
| RaGOO_A02_80776 | A02 | 16976080 | 4.75E-09 | 0.173 | 104 | 0.692 | 0.819 | 1.03E-05 | 12.7 |
| RaGOO_A02_89827 | A02 | 18562726 | 4.75E-09 | 0.159 | 104 | 0.692 | 0.819 | 1.03E-05 | 12.7 |
| RaGOO_A02_73202 | A02 | 15741440 | 4.77E-09 | 0.149 | 104 | 0.692 | 0.819 | 1.04E-05 | 12.7 |
| RaGOO_A02_116058 | A02 | 24546584 | 4.78E-09 | 0.154 | 104 | 0.692 | 0.819 | 1.04E-05 | 12.7 |
| RaGOO_A02_114258 | A02 | 24302951 | 4.78E-09 | 0.159 | 104 | 0.692 | 0.819 | 1.04E-05 | 12.7 |
| RaGOO_A02_106754 | A02 | 22009159 | 4.79E-09 | 0.159 | 104 | 0.692 | 0.819 | 1.04E-05 | 12.7 |
| RaGOO_A02_73187 | A02 | 15740382 | 4.79E-09 | 0.149 | 104 | 0.692 | 0.819 | 1.04E-05 | 12.7 |
| RaGOO_A02_109505 | A02 | 22634815 | 4.80E-09 | 0.168 | 104 | 0.692 | 0.819 | 1.04E-05 | 12.7 |
| RaGOO_A02_80714 | A02 | 16972797 | 4.81E-09 | 0.159 | 104 | 0.692 | 0.819 | 1.04E-05 | 12.7 |
| RaGOO_A02_110588 | A02 | 22838437 | 4.81E-09 | 0.159 | 104 | 0.692 | 0.819 | 1.04E-05 | 12.7 |
| RaGOO_A02_77062 | A02 | 16424326 | 4.82E-09 | 0.159 | 104 | 0.692 | 0.819 | 1.04E-05 | 12.7 |
| RaGOO_A02_119572 | A02 | 25152427 | 4.82E-09 | 0.159 | 104 | 0.692 | 0.819 | 1.04E-05 | 12.7 |
| RaGOO_A02_89387 | A02 | 18496456 | 4.83E-09 | 0.159 | 104 | 0.692 | 0.819 | 1.04E-05 | 12.7 |
| RaGOO_A02_75404 | A02 | 16184239 | 4.83E-09 | 0.154 | 104 | 0.692 | 0.819 | 1.04E-05 | 12.7 |
| RaGOO_A02_85814 | A02 | 17736039 | 4.84E-09 | 0.159 | 104 | 0.692 | 0.819 | 1.04E-05 | 12.7 |
| RaGOO_A02_109669 | A02 | 22658069 | 4.84E-09 | 0.163 | 104 | 0.692 | 0.819 | 1.04E-05 | 12.7 |
| RaGOO_A02_91876 | A02 | 18878841 | 4.84E-09 | 0.159 | 104 | 0.692 | 0.819 | 1.04E-05 | 12.7 |
| RaGOO_A02_112020 | A02 | 23876549 | 4.87E-09 | 0.159 | 104 | 0.692 | 0.819 | 1.05E-05 | 12.7 |
| RaGOO_A02_112019 | A02 | 23876547 | 4.87E-09 | 0.159 | 104 | 0.692 | 0.819 | 1.05E-05 | 12.7 |
| RaGOO_A02_97485 | A02 | 20170339 | 4.87E-09 | 0.159 | 104 | 0.692 | 0.819 | 1.05E-05 | 12.7 |
| RaGOO_A02_82548 | A02 | 17233787 | 4.90E-09 | 0.135 | 104 | 0.692 | 0.819 | 1.05E-05 | 12.7 |
| RaGOO_A02_82935 | A02 | 17281964 | 4.93E-09 | 0.159 | 104 | 0.692 | 0.819 | 1.06E-05 | 12.7 |
| RaGOO_A02_101573 | A02 | 20992103 | 4.93E-09 | 0.154 | 104 | 0.692 | 0.819 | 1.06E-05 | 12.7 |
| RaGOO_A02_98990 | A02 | 20472828 | 4.94E-09 | 0.144 | 104 | 0.692 | 0.819 | 1.06E-05 | 12.7 |
| RaGOO_A02_102065 | A02 | 21069696 | 4.96E-09 | 0.154 | 104 | 0.692 | 0.819 | 1.06E-05 | 12.7 |
| RaGOO_A02_89696 | A02 | 18548928 | 4.97E-09 | 0.144 | 104 | 0.692 | 0.819 | 1.06E-05 | 12.7 |
| RaGOO_A02_110762 | A02 | 22878698 | 4.98E-09 | 0.135 | 104 | 0.692 | 0.819 | 1.07E-05 | 12.7 |
| RaGOO_A02_105686 | A02 | 21873975 | 4.99E-09 | 0.159 | 104 | 0.692 | 0.819 | 1.07E-05 | 12.7 |
| RaGOO_A02_82388 | A02 | 17225257 | 5.01E-09 | 0.154 | 104 | 0.692 | 0.819 | 1.07E-05 | 12.6 |
| RaGOO_A02_110912 | A02 | 22902926 | 5.03E-09 | 0.159 | 104 | 0.692 | 0.818 | 1.07E-05 | 12.6 |
| RaGOO_A02_80280 | A02 | 16932958 | 5.03E-09 | 0.183 | 104 | 0.692 | 0.818 | 1.07E-05 | 12.6 |
| RaGOO_A02_113748 | A02 | 24203659 | 5.03E-09 | 0.168 | 104 | 0.692 | 0.818 | 1.07E-05 | 12.6 |
| RaGOO_A02_116182 | A02 | 24559082 | 5.05E-09 | 0.159 | 104 | 0.692 | 0.818 | 1.08E-05 | 12.6 |
| RaGOO_A02_105930 | A02 | 21908834 | 5.07E-09 | 0.154 | 104 | 0.692 | 0.818 | 1.08E-05 | 12.6 |
| RaGOO_A02_73218 | A02 | 15743229 | 5.07E-09 | 0.168 | 104 | 0.692 | 0.818 | 1.08E-05 | 12.6 |
| RaGOO_A02_105892 | A02 | 21900674 | 5.07E-09 | 0.173 | 104 | 0.692 | 0.818 | 1.08E-05 | 12.6 |
| RaGOO_A02_117924 | A02 | 24824249 | 5.07E-09 | 0.144 | 104 | 0.692 | 0.818 | 1.08E-05 | 12.6 |
| RaGOO_A02_105243 | A02 | 21798505 | 5.09E-09 | 0.163 | 104 | 0.692 | 0.818 | 1.08E-05 | 12.6 |
| RaGOO_A02_98000 | A02 | 20234850 | 5.09E-09 | 0.159 | 104 | 0.692 | 0.818 | 1.08E-05 | 12.6 |
| RaGOO_A02_102117 | A02 | 21076027 | 5.11E-09 | 0.163 | 104 | 0.692 | 0.818 | 1.08E-05 | 12.6 |
| RaGOO_A02_86745 | A02 | 17934108 | 5.14E-09 | 0.135 | 104 | 0.692 | 0.818 | 1.09E-05 | 12.6 |
| RaGOO_A02_77008 | A02 | 16416777 | 5.15E-09 | 0.163 | 104 | 0.692 | 0.818 | 1.09E-05 | 12.6 |

| RaGOO_A02_110748 | A02 | 22877168 | 5.17E-09 | 0.149 | 104 | 0.692 | 0.818 | 1.09E-05 | 12.6 |
| --- | --- | --- | --- | --- | --- | --- | --- | --- | --- |
| RaGOO_A02_105547 | A02 | 21843062 | 5.17E-09 | 0.178 | 104 | 0.692 | 0.818 | 1.09E-05 | 12.6 |
| RaGOO_A02_101899 | A02 | 21039574 | 5.18E-09 | 0.159 | 104 | 0.692 | 0.818 | 1.09E-05 | 12.6 |
| RaGOO_A02_99644 | A02 | 20568097 | 5.18E-09 | 0.149 | 104 | 0.692 | 0.818 | 1.09E-05 | 12.6 |
| RaGOO_A02_114088 | A02 | 24258136 | 5.22E-09 | 0.154 | 104 | 0.692 | 0.818 | 1.10E-05 | 12.6 |
| RaGOO_A02_111230 | A02 | 22948184 | 5.22E-09 | 0.163 | 104 | 0.692 | 0.818 | 1.10E-05 | 12.6 |
| RaGOO_A02_87321 | A02 | 18049905 | 5.22E-09 | 0.154 | 104 | 0.692 | 0.818 | 1.10E-05 | 12.6 |
| RaGOO_A02_102849 | A02 | 21170693 | 5.22E-09 | 0.163 | 104 | 0.692 | 0.818 | 1.10E-05 | 12.6 |
| RaGOO_A02_113599 | A02 | 24177361 | 5.23E-09 | 0.163 | 104 | 0.692 | 0.818 | 1.10E-05 | 12.6 |
| RaGOO_A02_97203 | A02 | 20129887 | 5.24E-09 | 0.163 | 104 | 0.692 | 0.818 | 1.10E-05 | 12.6 |
| RaGOO_A02_82443 | A02 | 17228576 | 5.25E-09 | 0.135 | 104 | 0.692 | 0.818 | 1.10E-05 | 12.6 |
| RaGOO_A02_82445 | A02 | 17228597 | 5.25E-09 | 0.135 | 104 | 0.692 | 0.818 | 1.10E-05 | 12.6 |
| RaGOO_A02_82272 | A02 | 17218659 | 5.26E-09 | 0.149 | 104 | 0.692 | 0.818 | 1.10E-05 | 12.6 |
| RaGOO_A02_80126 | A02 | 16916036 | 5.26E-09 | 0.163 | 104 | 0.692 | 0.818 | 1.10E-05 | 12.6 |
| RaGOO_A02_80633 | A02 | 16962386 | 5.27E-09 | 0.159 | 104 | 0.692 | 0.818 | 1.10E-05 | 12.6 |
| RaGOO_A02_87928 | A02 | 18155949 | 5.33E-09 | 0.163 | 104 | 0.692 | 0.818 | 1.12E-05 | 12.6 |
| RaGOO_A02_82340 | A02 | 17222601 | 5.34E-09 | 0.159 | 104 | 0.692 | 0.818 | 1.12E-05 | 12.6 |
| RaGOO_A02_109955 | A02 | 22696089 | 5.35E-09 | 0.149 | 104 | 0.692 | 0.818 | 1.12E-05 | 12.6 |
| RaGOO_A02_114621 | A02 | 24365660 | 5.35E-09 | 0.159 | 104 | 0.692 | 0.818 | 1.12E-05 | 12.6 |
| RaGOO_A02_98978 | A02 | 20471197 | 5.37E-09 | 0.149 | 104 | 0.692 | 0.818 | 1.12E-05 | 12.6 |
| RaGOO_A02_79303 | A02 | 16815861 | 5.39E-09 | 0.154 | 104 | 0.692 | 0.818 | 1.12E-05 | 12.6 |
| RaGOO_A02_90523 | A02 | 18669254 | 5.39E-09 | 0.139 | 104 | 0.692 | 0.818 | 1.12E-05 | 12.6 |
| RaGOO_A02_99057 | A02 | 20478127 | 5.42E-09 | 0.188 | 104 | 0.692 | 0.818 | 1.13E-05 | 12.6 |
| RaGOO_A02_99056 | A02 | 20478119 | 5.42E-09 | 0.188 | 104 | 0.692 | 0.818 | 1.13E-05 | 12.6 |
| RaGOO_A02_102067 | A02 | 21069806 | 5.43E-09 | 0.183 | 104 | 0.692 | 0.818 | 1.13E-05 | 12.6 |
| RaGOO_A02_100976 | A02 | 20928683 | 5.46E-09 | 0.163 | 104 | 0.692 | 0.818 | 1.13E-05 | 12.6 |
| RaGOO_A02_100802 | A02 | 20871264 | 5.46E-09 | 0.178 | 104 | 0.692 | 0.818 | 1.13E-05 | 12.6 |
| RaGOO_A02_72941 | A02 | 15705499 | 5.50E-09 | 0.149 | 104 | 0.692 | 0.818 | 1.14E-05 | 12.6 |
| RaGOO_A02_73078 | A02 | 15724866 | 5.50E-09 | 0.159 | 104 | 0.692 | 0.818 | 1.14E-05 | 12.6 |
| RaGOO_A02_73855 | A02 | 15870968 | 5.50E-09 | 0.159 | 104 | 0.692 | 0.818 | 1.14E-05 | 12.6 |
| RaGOO_A02_73856 | A02 | 15870969 | 5.50E-09 | 0.159 | 104 | 0.692 | 0.818 | 1.14E-05 | 12.6 |
| RaGOO_A02_112381 | A02 | 23925511 | 5.51E-09 | 0.159 | 104 | 0.692 | 0.818 | 1.14E-05 | 12.6 |
| RaGOO_A02_123671 | A02 | 25726685 | 5.51E-09 | 0.159 | 104 | 0.692 | 0.818 | 1.14E-05 | 12.6 |
| RaGOO_A02_112050 | A02 | 23879097 | 5.53E-09 | 0.159 | 104 | 0.692 | 0.818 | 1.14E-05 | 12.6 |
| RaGOO_A02_107197 | A02 | 22134498 | 5.55E-09 | 0.178 | 104 | 0.692 | 0.818 | 1.15E-05 | 12.6 |
| RaGOO_A02_83608 | A02 | 17372794 | 5.55E-09 | 0.163 | 104 | 0.692 | 0.818 | 1.15E-05 | 12.6 |
| RaGOO_A02_115928 | A02 | 24535599 | 5.57E-09 | 0.163 | 104 | 0.692 | 0.818 | 1.15E-05 | 12.6 |
| RaGOO_A02_123133 | A02 | 25684886 | 5.64E-09 | 0.149 | 104 | 0.692 | 0.817 | 1.16E-05 | 12.5 |
| RaGOO_A02_104448 | A02 | 21583256 | 5.67E-09 | 0.149 | 104 | 0.692 | 0.817 | 1.17E-05 | 12.5 |
| RaGOO_A02_105940 | A02 | 21909888 | 5.68E-09 | 0.154 | 104 | 0.692 | 0.817 | 1.17E-05 | 12.5 |
| RaGOO_A02_118628 | A02 | 24956296 | 5.68E-09 | 0.154 | 104 | 0.692 | 0.817 | 1.17E-05 | 12.5 |
| RaGOO_A02_111053 | A02 | 22918258 | 5.69E-09 | 0.168 | 104 | 0.692 | 0.817 | 1.17E-05 | 12.5 |
| RaGOO_A02_109484 | A02 | 22632495 | 5.73E-09 | 0.168 | 104 | 0.692 | 0.817 | 1.18E-05 | 12.5 |
| RaGOO_A02_80101 | A02 | 16913461 | 5.74E-09 | 0.163 | 104 | 0.692 | 0.817 | 1.18E-05 | 12.5 |
| RaGOO_A02_99624 | A02 | 20561987 | 5.76E-09 | 0.159 | 104 | 0.692 | 0.817 | 1.18E-05 | 12.5 |
| RaGOO_A02_72297 | A02 | 15535167 | 5.77E-09 | 0.163 | 104 | 0.692 | 0.817 | 1.18E-05 | 12.5 |
| RaGOO_A02_108623 | A02 | 22426025 | 5.78E-09 | 0.173 | 104 | 0.692 | 0.817 | 1.18E-05 | 12.5 |
| RaGOO_A02_100843 | A02 | 20891918 | 5.80E-09 | 0.163 | 104 | 0.692 | 0.817 | 1.19E-05 | 12.5 |
| RaGOO_A02_84459 | A02 | 17502140 | 5.83E-09 | 0.144 | 104 | 0.692 | 0.817 | 1.19E-05 | 12.5 |
| RaGOO_A02_97674 | A02 | 20189071 | 5.84E-09 | 0.159 | 104 | 0.692 | 0.817 | 1.19E-05 | 12.5 |
| RaGOO_A02_112400 | A02 | 23929496 | 5.84E-09 | 0.178 | 104 | 0.692 | 0.817 | 1.19E-05 | 12.5 |
| RaGOO_A02_98366 | A02 | 20321421 | 5.85E-09 | 0.173 | 104 | 0.692 | 0.817 | 1.19E-05 | 12.5 |
| RaGOO_A02_96076 | A02 | 19955222 | 5.86E-09 | 0.154 | 104 | 0.692 | 0.817 | 1.19E-05 | 12.5 |
| RaGOO_A02_96077 | A02 | 19955225 | 5.86E-09 | 0.154 | 104 | 0.692 | 0.817 | 1.19E-05 | 12.5 |
| RaGOO_A02_99751 | A02 | 20594772 | 5.86E-09 | 0.149 | 104 | 0.692 | 0.817 | 1.19E-05 | 12.5 |
| RaGOO_A02_79838 | A02 | 16880942 | 5.87E-09 | 0.159 | 104 | 0.692 | 0.817 | 1.20E-05 | 12.5 |
| RaGOO_A02_103541 | A02 | 21377431 | 5.90E-09 | 0.163 | 104 | 0.692 | 0.817 | 1.20E-05 | 12.5 |
| RaGOO_A02_115489 | A02 | 24473270 | 5.91E-09 | 0.163 | 104 | 0.692 | 0.817 | 1.20E-05 | 12.5 |
| RaGOO_A02_109885 | A02 | 22679746 | 5.92E-09 | 0.154 | 104 | 0.692 | 0.817 | 1.20E-05 | 12.5 |
| RaGOO_A02_103817 | A02 | 21495797 | 5.93E-09 | 0.168 | 104 | 0.692 | 0.817 | 1.21E-05 | 12.5 |
| RaGOO_A02_101181 | A02 | 20948397 | 5.94E-09 | 0.168 | 104 | 0.692 | 0.817 | 1.21E-05 | 12.5 |
| RaGOO_A02_89525 | A02 | 18524267 | 5.94E-09 | 0.154 | 104 | 0.692 | 0.817 | 1.21E-05 | 12.5 |
| RaGOO_A02_114995 | A02 | 24409686 | 5.94E-09 | 0.168 | 104 | 0.692 | 0.817 | 1.21E-05 | 12.5 |
| RaGOO_A02_102332 | A02 | 21109926 | 5.95E-09 | 0.163 | 104 | 0.692 | 0.817 | 1.21E-05 | 12.5 |
| RaGOO_A02_97992 | A02 | 20234151 | 5.96E-09 | 0.168 | 104 | 0.692 | 0.817 | 1.21E-05 | 12.5 |
| RaGOO_A02_117872 | A02 | 24819158 | 5.96E-09 | 0.159 | 104 | 0.692 | 0.817 | 1.21E-05 | 12.5 |
| RaGOO_A02_85728 | A02 | 17724934 | 5.97E-09 | 0.149 | 104 | 0.692 | 0.817 | 1.21E-05 | 12.5 |
| RaGOO_A02_110820 | A02 | 22884548 | 5.99E-09 | 0.163 | 104 | 0.692 | 0.817 | 1.21E-05 | 12.5 |
| RaGOO_A02_112382 | A02 | 23925512 | 6.01E-09 | 0.115 | 104 | 0.692 | 0.817 | 1.21E-05 | 12.5 |
| RaGOO_A02_118653 | A02 | 24965153 | 6.03E-09 | 0.154 | 104 | 0.692 | 0.817 | 1.22E-05 | 12.5 |
| RaGOO_A02_101987 | A02 | 21049331 | 6.04E-09 | 0.173 | 104 | 0.692 | 0.817 | 1.22E-05 | 12.5 |
| RaGOO_A02_73147 | A02 | 15737125 | 6.05E-09 | 0.144 | 104 | 0.692 | 0.817 | 1.22E-05 | 12.5 |
| RaGOO_A02_109996 | A02 | 22751564 | 6.05E-09 | 0.163 | 104 | 0.692 | 0.817 | 1.22E-05 | 12.5 |
| RaGOO_A02_109995 | A02 | 22751559 | 6.05E-09 | 0.163 | 104 | 0.692 | 0.817 | 1.22E-05 | 12.5 |
| RaGOO_A02_115797 | A02 | 24517988 | 6.05E-09 | 0.173 | 104 | 0.692 | 0.817 | 1.22E-05 | 12.5 |
| RaGOO_A02_91802 | A02 | 18874184 | 6.05E-09 | 0.173 | 104 | 0.692 | 0.817 | 1.22E-05 | 12.5 |
| RaGOO_A02_102875 | A02 | 21173918 | 6.06E-09 | 0.168 | 104 | 0.692 | 0.817 | 1.22E-05 | 12.5 |
| RaGOO_A02_90343 | A02 | 18639511 | 6.07E-09 | 0.173 | 104 | 0.692 | 0.817 | 1.22E-05 | 12.5 |
| RaGOO_A02_103093 | A02 | 21230115 | 6.11E-09 | 0.178 | 104 | 0.692 | 0.817 | 1.23E-05 | 12.5 |
| RaGOO_A02_109848 | A02 | 22676041 | 6.11E-09 | 0.159 | 104 | 0.692 | 0.817 | 1.23E-05 | 12.5 |
| RaGOO_A02_91798 | A02 | 18873962 | 6.13E-09 | 0.154 | 104 | 0.692 | 0.817 | 1.23E-05 | 12.5 |
| RaGOO_A02_114624 | A02 | 24365974 | 6.13E-09 | 0.188 | 104 | 0.692 | 0.817 | 1.23E-05 | 12.5 |
| RaGOO_A02_79887 | A02 | 16885067 | 6.15E-09 | 0.168 | 104 | 0.692 | 0.817 | 1.23E-05 | 12.5 |
| RaGOO_A02_79025 | A02 | 16782956 | 6.17E-09 | 0.168 | 104 | 0.692 | 0.817 | 1.23E-05 | 12.5 |
| RaGOO_A02_73156 | A02 | 15737744 | 6.20E-09 | 0.168 | 104 | 0.692 | 0.817 | 1.24E-05 | 12.5 |
| RaGOO_A02_100591 | A02 | 20842663 | 6.21E-09 | 0.149 | 104 | 0.692 | 0.817 | 1.24E-05 | 12.5 |
| RaGOO_A02_97619 | A02 | 20185264 | 6.23E-09 | 0.125 | 104 | 0.692 | 0.817 | 1.24E-05 | 12.5 |
| RaGOO_A02_97460 | A02 | 20167117 | 6.25E-09 | 0.149 | 104 | 0.692 | 0.817 | 1.25E-05 | 12.5 |
| RaGOO_A02_105498 | A02 | 21834537 | 6.27E-09 | 0.168 | 104 | 0.692 | 0.817 | 1.25E-05 | 12.5 |
| RaGOO_A02_115811 | A02 | 24519038 | 6.27E-09 | 0.173 | 104 | 0.692 | 0.817 | 1.25E-05 | 12.5 |
| RaGOO_A02_116083 | A02 | 24547903 | 6.31E-09 | 0.173 | 104 | 0.692 | 0.817 | 1.26E-05 | 12.5 |

| RaGOO_A02_98256 | A02 | 20290422 | 6.31E-09 | 0.159 | 104 | 0.692 | 0.817 | 1.26E-05 | 12.5 |
| --- | --- | --- | --- | --- | --- | --- | --- | --- | --- |
| RaGOO_A02_75394 | A02 | 16183544 | 6.31E-09 | 0.154 | 104 | 0.692 | 0.817 | 1.26E-05 | 12.5 |
| RaGOO_A02_116091 | A02 | 24548358 | 6.33E-09 | 0.149 | 104 | 0.692 | 0.817 | 1.26E-05 | 12.4 |
| RaGOO_A02_102606 | A02 | 21143594 | 6.36E-09 | 0.163 | 104 | 0.692 | 0.816 | 1.26E-05 | 12.4 |
| RaGOO_A02_88372 | A02 | 18218420 | 6.36E-09 | 0.135 | 104 | 0.692 | 0.816 | 1.26E-05 | 12.4 |
| RaGOO_A02_100856 | A02 | 20892823 | 6.38E-09 | 0.163 | 104 | 0.692 | 0.816 | 1.27E-05 | 12.4 |
| RaGOO_A02_73990 | A02 | 15953849 | 6.39E-09 | 0.149 | 104 | 0.692 | 0.816 | 1.27E-05 | 12.4 |
| RaGOO_A02_116086 | A02 | 24547991 | 6.39E-09 | 0.159 | 104 | 0.692 | 0.816 | 1.27E-05 | 12.4 |
| RaGOO_A02_75270 | A02 | 16172058 | 6.41E-09 | 0.154 | 104 | 0.692 | 0.816 | 1.27E-05 | 12.4 |
| RaGOO_A02_115812 | A02 | 24519065 | 6.42E-09 | 0.192 | 104 | 0.692 | 0.816 | 1.27E-05 | 12.4 |
| RaGOO_A02_99109 | A02 | 20481701 | 6.42E-09 | 0.163 | 104 | 0.692 | 0.816 | 1.27E-05 | 12.4 |
| RaGOO_A02_108257 | A02 | 22378844 | 6.43E-09 | 0.178 | 104 | 0.692 | 0.816 | 1.27E-05 | 12.4 |
| RaGOO_A02_100290 | A02 | 20759784 | 6.45E-09 | 0.159 | 104 | 0.692 | 0.816 | 1.27E-05 | 12.4 |
| RaGOO_A02_118825 | A02 | 24977523 | 6.50E-09 | 0.168 | 104 | 0.692 | 0.816 | 1.28E-05 | 12.4 |
| RaGOO_A02_103547 | A02 | 21378518 | 6.51E-09 | 0.168 | 104 | 0.692 | 0.816 | 1.28E-05 | 12.4 |
| RaGOO_A02_104252 | A02 | 21554597 | 6.53E-09 | 0.154 | 104 | 0.692 | 0.816 | 1.29E-05 | 12.4 |
| RaGOO_A02_115833 | A02 | 24520223 | 6.55E-09 | 0.163 | 104 | 0.692 | 0.816 | 1.29E-05 | 12.4 |
| RaGOO_A02_119414 | A02 | 25140961 | 6.55E-09 | 0.159 | 104 | 0.692 | 0.816 | 1.29E-05 | 12.4 |
| RaGOO_A02_99728 | A02 | 20586928 | 6.56E-09 | 0.154 | 104 | 0.692 | 0.816 | 1.29E-05 | 12.4 |
| RaGOO_A02_110631 | A02 | 22842215 | 6.57E-09 | 0.159 | 104 | 0.692 | 0.816 | 1.29E-05 | 12.4 |
| RaGOO_A02_117960 | A02 | 24827306 | 6.58E-09 | 0.159 | 104 | 0.692 | 0.816 | 1.29E-05 | 12.4 |
| RaGOO_A02_85829 | A02 | 17737795 | 6.59E-09 | 0.154 | 104 | 0.692 | 0.816 | 1.29E-05 | 12.4 |
| RaGOO_A02_99961 | A02 | 20657232 | 6.59E-09 | 0.163 | 104 | 0.692 | 0.816 | 1.29E-05 | 12.4 |
| RaGOO_A02_96598 | A02 | 20033912 | 6.60E-09 | 0.144 | 104 | 0.692 | 0.816 | 1.30E-05 | 12.4 |
| RaGOO_A02_98034 | A02 | 20254257 | 6.62E-09 | 0.192 | 104 | 0.692 | 0.816 | 1.30E-05 | 12.4 |
| RaGOO_A02_111002 | A02 | 22915556 | 6.63E-09 | 0.163 | 104 | 0.692 | 0.816 | 1.30E-05 | 12.4 |
| RaGOO_A02_115301 | A02 | 24443028 | 6.63E-09 | 0.154 | 104 | 0.692 | 0.816 | 1.30E-05 | 12.4 |
| RaGOO_A02_92062 | A02 | 18897749 | 6.65E-09 | 0.173 | 104 | 0.692 | 0.816 | 1.30E-05 | 12.4 |
| RaGOO_A02_118014 | A02 | 24832534 | 6.67E-09 | 0.154 | 104 | 0.692 | 0.816 | 1.30E-05 | 12.4 |
| RaGOO_A02_98025 | A02 | 20253584 | 6.67E-09 | 0.149 | 104 | 0.692 | 0.816 | 1.30E-05 | 12.4 |
| RaGOO_A02_102872 | A02 | 21173678 | 6.70E-09 | 0.168 | 104 | 0.692 | 0.816 | 1.31E-05 | 12.4 |
| RaGOO_A02_75390 | A02 | 16183241 | 6.72E-09 | 0.149 | 104 | 0.692 | 0.816 | 1.31E-05 | 12.4 |
| RaGOO_A02_103852 | A02 | 21497490 | 6.73E-09 | 0.159 | 104 | 0.692 | 0.816 | 1.31E-05 | 12.4 |
| RaGOO_A02_86936 | A02 | 17983729 | 6.77E-09 | 0.168 | 104 | 0.692 | 0.816 | 1.32E-05 | 12.4 |
| RaGOO_A02_110213 | A02 | 22780526 | 6.78E-09 | 0.159 | 104 | 0.692 | 0.816 | 1.32E-05 | 12.4 |
| RaGOO_A02_115323 | A02 | 24449981 | 6.87E-09 | 0.188 | 104 | 0.692 | 0.816 | 1.34E-05 | 12.4 |
| RaGOO_A02_84477 | A02 | 17504453 | 6.89E-09 | 0.154 | 104 | 0.692 | 0.816 | 1.34E-05 | 12.4 |
| RaGOO_A02_82364 | A02 | 17223966 | 6.89E-09 | 0.154 | 104 | 0.692 | 0.816 | 1.34E-05 | 12.4 |
| RaGOO_A02_100740 | A02 | 20863596 | 6.95E-09 | 0.149 | 104 | 0.692 | 0.816 | 1.35E-05 | 12.4 |
| RaGOO_A02_115782 | A02 | 24516827 | 6.97E-09 | 0.149 | 104 | 0.692 | 0.816 | 1.35E-05 | 12.4 |
| RaGOO_A02_108218 | A02 | 22373114 | 6.97E-09 | 0.159 | 104 | 0.692 | 0.816 | 1.35E-05 | 12.4 |
| RaGOO_A02_103359 | A02 | 21317402 | 6.98E-09 | 0.139 | 104 | 0.692 | 0.816 | 1.35E-05 | 12.4 |
| RaGOO_A02_76979 | A02 | 16415615 | 6.98E-09 | 0.154 | 104 | 0.692 | 0.816 | 1.35E-05 | 12.4 |
| RaGOO_A02_97106 | A02 | 20105957 | 7.02E-09 | 0.178 | 104 | 0.692 | 0.816 | 1.36E-05 | 12.4 |
| RaGOO_A02_110423 | A02 | 22814421 | 7.02E-09 | 0.154 | 104 | 0.692 | 0.816 | 1.36E-05 | 12.4 |
| RaGOO_A02_85690 | A02 | 17720706 | 7.05E-09 | 0.173 | 104 | 0.692 | 0.816 | 1.37E-05 | 12.4 |
| RaGOO_A02_90241 | A02 | 18616070 | 7.07E-09 | 0.188 | 104 | 0.692 | 0.816 | 1.37E-05 | 12.4 |
| RaGOO_A02_99889 | A02 | 20630655 | 7.10E-09 | 0.163 | 104 | 0.692 | 0.816 | 1.37E-05 | 12.4 |
| RaGOO_A02_118677 | A02 | 24966904 | 7.10E-09 | 0.163 | 104 | 0.692 | 0.816 | 1.37E-05 | 12.4 |
| RaGOO_A02_88707 | A02 | 18274835 | 7.13E-09 | 0.159 | 104 | 0.692 | 0.816 | 1.37E-05 | 12.3 |
| RaGOO_A02_91162 | A02 | 18762933 | 7.13E-09 | 0.149 | 104 | 0.692 | 0.815 | 1.37E-05 | 12.3 |
| RaGOO_A02_104270 | A02 | 21558032 | 7.13E-09 | 0.159 | 104 | 0.692 | 0.815 | 1.37E-05 | 12.3 |
| RaGOO_A02_114645 | A02 | 24366468 | 7.14E-09 | 0.154 | 104 | 0.692 | 0.815 | 1.37E-05 | 12.3 |
| RaGOO_A02_99160 | A02 | 20486357 | 7.14E-09 | 0.183 | 104 | 0.692 | 0.815 | 1.37E-05 | 12.3 |
| RaGOO_A02_117723 | A02 | 24801655 | 7.15E-09 | 0.168 | 104 | 0.692 | 0.815 | 1.37E-05 | 12.3 |
| RaGOO_A02_96842 | A02 | 20075700 | 7.15E-09 | 0.154 | 104 | 0.692 | 0.815 | 1.37E-05 | 12.3 |
| RaGOO_A02_123094 | A02 | 25683320 | 7.16E-09 | 0.144 | 104 | 0.692 | 0.815 | 1.38E-05 | 12.3 |
| RaGOO_A02_90233 | A02 | 18615636 | 7.17E-09 | 0.149 | 104 | 0.692 | 0.815 | 1.38E-05 | 12.3 |
| RaGOO_A02_100266 | A02 | 20750794 | 7.19E-09 | 0.178 | 104 | 0.692 | 0.815 | 1.38E-05 | 12.3 |
| RaGOO_A02_109486 | A02 | 22632570 | 7.23E-09 | 0.159 | 104 | 0.692 | 0.815 | 1.39E-05 | 12.3 |
| RaGOO_A02_73982 | A02 | 15953627 | 7.23E-09 | 0.159 | 104 | 0.692 | 0.815 | 1.39E-05 | 12.3 |
| RaGOO_A02_73983 | A02 | 15953629 | 7.23E-09 | 0.159 | 104 | 0.692 | 0.815 | 1.39E-05 | 12.3 |
| RaGOO_A02_109866 | A02 | 22677882 | 7.24E-09 | 0.144 | 104 | 0.692 | 0.815 | 1.39E-05 | 12.3 |
| RaGOO_A02_87296 | A02 | 18048293 | 7.28E-09 | 0.154 | 104 | 0.692 | 0.815 | 1.39E-05 | 12.3 |
| RaGOO_A02_89318 | A02 | 18480073 | 7.33E-09 | 0.159 | 104 | 0.692 | 0.815 | 1.40E-05 | 12.3 |
| RaGOO_A02_117761 | A02 | 24803749 | 7.34E-09 | 0.144 | 104 | 0.692 | 0.815 | 1.40E-05 | 12.3 |
| RaGOO_A02_80835 | A02 | 16979820 | 7.34E-09 | 0.168 | 104 | 0.692 | 0.815 | 1.40E-05 | 12.3 |
| RaGOO_A02_80836 | A02 | 16979824 | 7.34E-09 | 0.168 | 104 | 0.692 | 0.815 | 1.40E-05 | 12.3 |
| RaGOO_A02_109482 | A02 | 22632277 | 7.34E-09 | 0.168 | 104 | 0.692 | 0.815 | 1.40E-05 | 12.3 |
| RaGOO_A02_116037 | A02 | 24545699 | 7.35E-09 | 0.149 | 104 | 0.692 | 0.815 | 1.40E-05 | 12.3 |
| RaGOO_A02_100807 | A02 | 20871522 | 7.36E-09 | 0.159 | 104 | 0.692 | 0.815 | 1.40E-05 | 12.3 |
| RaGOO_A02_104449 | A02 | 21583288 | 7.37E-09 | 0.149 | 104 | 0.692 | 0.815 | 1.40E-05 | 12.3 |
| RaGOO_A02_91742 | A02 | 18865359 | 7.37E-09 | 0.183 | 104 | 0.692 | 0.815 | 1.40E-05 | 12.3 |
| RaGOO_A02_113835 | A02 | 24230650 | 7.37E-09 | 0.159 | 104 | 0.692 | 0.815 | 1.40E-05 | 12.3 |
| RaGOO_A02_90179 | A02 | 18608033 | 7.40E-09 | 0.154 | 104 | 0.692 | 0.815 | 1.41E-05 | 12.3 |
| RaGOO_A02_76448 | A02 | 16295934 | 7.40E-09 | 0.159 | 104 | 0.692 | 0.815 | 1.41E-05 | 12.3 |
| RaGOO_A02_82453 | A02 | 17229033 | 7.41E-09 | 0.115 | 104 | 0.692 | 0.815 | 1.41E-05 | 12.3 |
| RaGOO_A02_73188 | A02 | 15740437 | 7.42E-09 | 0.149 | 104 | 0.692 | 0.815 | 1.41E-05 | 12.3 |
| RaGOO_A02_102303 | A02 | 21106594 | 7.42E-09 | 0.149 | 104 | 0.692 | 0.815 | 1.41E-05 | 12.3 |
| RaGOO_A02_111570 | A02 | 23162557 | 7.43E-09 | 0.168 | 104 | 0.692 | 0.815 | 1.41E-05 | 12.3 |
| RaGOO_A02_97195 | A02 | 20129089 | 7.44E-09 | 0.154 | 104 | 0.692 | 0.815 | 1.41E-05 | 12.3 |
| RaGOO_A02_122971 | A02 | 25668249 | 7.44E-09 | 0.245 | 104 | 0.692 | 0.815 | 1.41E-05 | 12.3 |
| RaGOO_A02_113123 | A02 | 24036721 | 7.44E-09 | 0.173 | 104 | 0.692 | 0.815 | 1.41E-05 | 12.3 |
| RaGOO_A02_115054 | A02 | 24417504 | 7.46E-09 | 0.149 | 104 | 0.692 | 0.815 | 1.41E-05 | 12.3 |
| RaGOO_A02_75613 | A02 | 16194828 | 7.47E-09 | 0.159 | 104 | 0.692 | 0.815 | 1.41E-05 | 12.3 |
| RaGOO_A02_89433 | A02 | 18512621 | 7.49E-09 | 0.168 | 104 | 0.692 | 0.815 | 1.42E-05 | 12.3 |
| RaGOO_A02_96214 | A02 | 19983076 | 7.49E-09 | 0.163 | 104 | 0.692 | 0.815 | 1.42E-05 | 12.3 |
| RaGOO_A02_76976 | A02 | 16415546 | 7.54E-09 | 0.168 | 104 | 0.692 | 0.815 | 1.42E-05 | 12.3 |
| RaGOO_A02_77373 | A02 | 16490318 | 7.56E-09 | 0.168 | 104 | 0.692 | 0.815 | 1.43E-05 | 12.3 |
| RaGOO_A02_99711 | A02 | 20582707 | 7.59E-09 | 0.149 | 104 | 0.692 | 0.815 | 1.43E-05 | 12.3 |
| RaGOO_A02_109322 | A02 | 22603297 | 7.60E-09 | 0.139 | 104 | 0.692 | 0.815 | 1.43E-05 | 12.3 |

| RaGOO_A02_72647 | A02 | 15607707 | 7.62E-09 | 0.139 | 104 | 0.692 | 0.815 | 1.43E-05 | 12.3 |
| --- | --- | --- | --- | --- | --- | --- | --- | --- | --- |
| RaGOO_A02_91728 | A02 | 18863703 | 7.62E-09 | 0.125 | 104 | 0.692 | 0.815 | 1.43E-05 | 12.3 |
| RaGOO_A02_85666 | A02 | 17718445 | 7.63E-09 | 0.168 | 104 | 0.692 | 0.815 | 1.44E-05 | 12.3 |
| RaGOO_A02_80099 | A02 | 16913444 | 7.66E-09 | 0.154 | 104 | 0.692 | 0.815 | 1.44E-05 | 12.3 |
| RaGOO_A02_82374 | A02 | 17224365 | 7.67E-09 | 0.163 | 104 | 0.692 | 0.815 | 1.44E-05 | 12.3 |
| RaGOO_A02_114159 | A02 | 24278770 | 7.67E-09 | 0.168 | 104 | 0.692 | 0.815 | 1.44E-05 | 12.3 |
| RaGOO_A02_92227 | A02 | 18913189 | 7.68E-09 | 0.154 | 104 | 0.692 | 0.815 | 1.44E-05 | 12.3 |
| RaGOO_A02_100621 | A02 | 20844057 | 7.69E-09 | 0.163 | 104 | 0.692 | 0.815 | 1.44E-05 | 12.3 |
| RaGOO_A02_112498 | A02 | 23948376 | 7.75E-09 | 0.183 | 104 | 0.692 | 0.815 | 1.45E-05 | 12.3 |
| RaGOO_A02_105458 | A02 | 21826550 | 7.75E-09 | 0.159 | 104 | 0.692 | 0.815 | 1.45E-05 | 12.3 |
| RaGOO_A02_98051 | A02 | 20256316 | 7.75E-09 | 0.149 | 104 | 0.692 | 0.815 | 1.45E-05 | 12.3 |
| RaGOO_A02_98052 | A02 | 20256318 | 7.75E-09 | 0.149 | 104 | 0.692 | 0.815 | 1.45E-05 | 12.3 |
| RaGOO_A02_100346 | A02 | 20781159 | 7.79E-09 | 0.159 | 104 | 0.692 | 0.815 | 1.46E-05 | 12.3 |
| RaGOO_A02_102956 | A02 | 21209351 | 7.81E-09 | 0.106 | 104 | 0.692 | 0.815 | 1.46E-05 | 12.3 |
| RaGOO_A02_102957 | A02 | 21209352 | 7.81E-09 | 0.106 | 104 | 0.692 | 0.815 | 1.46E-05 | 12.3 |
| RaGOO_A02_108794 | A02 | 22463507 | 7.82E-09 | 0.159 | 104 | 0.692 | 0.815 | 1.46E-05 | 12.3 |
| RaGOO_A02_102116 | A02 | 21076020 | 7.91E-09 | 0.159 | 104 | 0.692 | 0.815 | 1.48E-05 | 12.3 |
| RaGOO_A02_99037 | A02 | 20476025 | 7.95E-09 | 0.168 | 104 | 0.692 | 0.815 | 1.48E-05 | 12.3 |
| RaGOO_A02_119075 | A02 | 25005269 | 7.95E-09 | 0.149 | 104 | 0.692 | 0.815 | 1.48E-05 | 12.3 |
| RaGOO_A02_102945 | A02 | 21208383 | 7.96E-09 | 0.183 | 104 | 0.692 | 0.815 | 1.48E-05 | 12.3 |
| RaGOO_A02_116066 | A02 | 24547023 | 7.98E-09 | 0.154 | 104 | 0.692 | 0.815 | 1.48E-05 | 12.3 |
| RaGOO_A02_116067 | A02 | 24547029 | 7.98E-09 | 0.154 | 104 | 0.692 | 0.815 | 1.48E-05 | 12.3 |
| RaGOO_A02_75399 | A02 | 16183896 | 7.98E-09 | 0.149 | 104 | 0.692 | 0.815 | 1.48E-05 | 12.3 |
| RaGOO_A02_73054 | A02 | 15722879 | 7.98E-09 | 0.154 | 104 | 0.692 | 0.815 | 1.48E-05 | 12.3 |
| RaGOO_A02_100385 | A02 | 20792222 | 8.00E-09 | 0.139 | 104 | 0.692 | 0.815 | 1.48E-05 | 12.3 |
| RaGOO_A02_101745 | A02 | 21012042 | 8.00E-09 | 0.144 | 104 | 0.692 | 0.815 | 1.48E-05 | 12.3 |
| RaGOO_A02_114650 | A02 | 24366806 | 8.00E-09 | 0.159 | 104 | 0.692 | 0.815 | 1.48E-05 | 12.3 |
| RaGOO_A02_82563 | A02 | 17234635 | 8.04E-09 | 0.168 | 104 | 0.692 | 0.814 | 1.49E-05 | 12.2 |
| RaGOO_A02_114984 | A02 | 24408765 | 8.06E-09 | 0.168 | 104 | 0.692 | 0.814 | 1.49E-05 | 12.2 |
| RaGOO_A02_114427 | A02 | 24323465 | 8.06E-09 | 0.154 | 104 | 0.692 | 0.814 | 1.49E-05 | 12.2 |
| RaGOO_A02_92078 | A02 | 18898603 | 8.08E-09 | 0.154 | 104 | 0.692 | 0.814 | 1.49E-05 | 12.2 |
| RaGOO_A02_92079 | A02 | 18898606 | 8.08E-09 | 0.154 | 104 | 0.692 | 0.814 | 1.49E-05 | 12.2 |
| RaGOO_A02_82367 | A02 | 17224171 | 8.10E-09 | 0.154 | 104 | 0.692 | 0.814 | 1.50E-05 | 12.2 |
| RaGOO_A02_119458 | A02 | 25143584 | 8.14E-09 | 0.168 | 104 | 0.692 | 0.814 | 1.50E-05 | 12.2 |
| RaGOO_A02_109507 | A02 | 22634939 | 8.15E-09 | 0.159 | 104 | 0.692 | 0.814 | 1.50E-05 | 12.2 |
| RaGOO_A02_84483 | A02 | 17505474 | 8.18E-09 | 0.154 | 104 | 0.692 | 0.814 | 1.51E-05 | 12.2 |
| RaGOO_A02_105469 | A02 | 21828435 | 8.22E-09 | 0.154 | 104 | 0.692 | 0.814 | 1.51E-05 | 12.2 |
| RaGOO_A02_89710 | A02 | 18551209 | 8.23E-09 | 0.163 | 104 | 0.692 | 0.814 | 1.51E-05 | 12.2 |
| RaGOO_A02_98836 | A02 | 20451159 | 8.23E-09 | 0.173 | 104 | 0.692 | 0.814 | 1.51E-05 | 12.2 |
| RaGOO_A02_113898 | A02 | 24234794 | 8.23E-09 | 0.154 | 104 | 0.692 | 0.814 | 1.51E-05 | 12.2 |
| RaGOO_A02_82390 | A02 | 17225448 | 8.24E-09 | 0.159 | 104 | 0.692 | 0.814 | 1.51E-05 | 12.2 |
| RaGOO_A02_104307 | A02 | 21563485 | 8.27E-09 | 0.154 | 104 | 0.692 | 0.814 | 1.52E-05 | 12.2 |
| RaGOO_A02_89129 | A02 | 18423114 | 8.29E-09 | 0.154 | 104 | 0.692 | 0.814 | 1.52E-05 | 12.2 |
| RaGOO_A02_108564 | A02 | 22422362 | 8.29E-09 | 0.183 | 104 | 0.692 | 0.814 | 1.52E-05 | 12.2 |
| RaGOO_A02_112387 | A02 | 23925676 | 8.31E-09 | 0.159 | 104 | 0.692 | 0.814 | 1.52E-05 | 12.2 |
| RaGOO_A02_119069 | A02 | 25004663 | 8.32E-09 | 0.159 | 104 | 0.692 | 0.814 | 1.52E-05 | 12.2 |
| RaGOO_A02_113111 | A02 | 24036356 | 8.40E-09 | 0.159 | 104 | 0.692 | 0.814 | 1.54E-05 | 12.2 |
| RaGOO_A02_82527 | A02 | 17233271 | 8.40E-09 | 0.120 | 104 | 0.692 | 0.814 | 1.54E-05 | 12.2 |
| RaGOO_A02_118629 | A02 | 24956327 | 8.41E-09 | 0.154 | 104 | 0.692 | 0.814 | 1.54E-05 | 12.2 |
| RaGOO_A02_110608 | A02 | 22839904 | 8.41E-09 | 0.144 | 104 | 0.692 | 0.814 | 1.54E-05 | 12.2 |
| RaGOO_A02_98131 | A02 | 20274453 | 8.44E-09 | 0.154 | 104 | 0.692 | 0.814 | 1.54E-05 | 12.2 |
| RaGOO_A02_113120 | A02 | 24036630 | 8.45E-09 | 0.149 | 104 | 0.692 | 0.814 | 1.54E-05 | 12.2 |
| RaGOO_A02_89701 | A02 | 18549417 | 8.46E-09 | 0.163 | 104 | 0.692 | 0.814 | 1.54E-05 | 12.2 |
| RaGOO_A02_78865 | A02 | 16759163 | 8.46E-09 | 0.178 | 104 | 0.692 | 0.814 | 1.54E-05 | 12.2 |
| RaGOO_A02_91182 | A02 | 18763991 | 8.49E-09 | 0.149 | 104 | 0.692 | 0.814 | 1.55E-05 | 12.2 |
| RaGOO_A02_91854 | A02 | 18877406 | 8.49E-09 | 0.178 | 104 | 0.692 | 0.814 | 1.55E-05 | 12.2 |
| RaGOO_A02_91866 | A02 | 18878246 | 8.50E-09 | 0.183 | 104 | 0.692 | 0.814 | 1.55E-05 | 12.2 |
| RaGOO_A02_115091 | A02 | 24420662 | 8.51E-09 | 0.163 | 104 | 0.692 | 0.814 | 1.55E-05 | 12.2 |
| RaGOO_A02_105324 | A02 | 21803215 | 8.51E-09 | 0.159 | 104 | 0.692 | 0.814 | 1.55E-05 | 12.2 |
| RaGOO_A02_117379 | A02 | 24731159 | 8.51E-09 | 0.159 | 104 | 0.692 | 0.814 | 1.55E-05 | 12.2 |
| RaGOO_A02_122018 | A02 | 25552300 | 8.54E-09 | 0.236 | 104 | 0.692 | 0.814 | 1.55E-05 | 12.2 |
| RaGOO_A02_76055 | A02 | 16246154 | 8.56E-09 | 0.202 | 104 | 0.692 | 0.814 | 1.55E-05 | 12.2 |
| RaGOO_A02_102556 | A02 | 21140471 | 8.56E-09 | 0.163 | 104 | 0.692 | 0.814 | 1.55E-05 | 12.2 |
| RaGOO_A02_115869 | A02 | 24528753 | 8.57E-09 | 0.168 | 104 | 0.692 | 0.814 | 1.56E-05 | 12.2 |
| RaGOO_A02_91330 | A02 | 18818223 | 8.58E-09 | 0.163 | 104 | 0.692 | 0.814 | 1.56E-05 | 12.2 |
| RaGOO_A02_90710 | A02 | 18688410 | 8.59E-09 | 0.178 | 104 | 0.692 | 0.814 | 1.56E-05 | 12.2 |
| RaGOO_A02_101017 | A02 | 20931900 | 8.62E-09 | 0.163 | 104 | 0.692 | 0.814 | 1.56E-05 | 12.2 |
| RaGOO_A02_115930 | A02 | 24535665 | 8.65E-09 | 0.163 | 104 | 0.692 | 0.814 | 1.57E-05 | 12.2 |
| RaGOO_A02_101210 | A02 | 20950517 | 8.66E-09 | 0.154 | 104 | 0.692 | 0.814 | 1.57E-05 | 12.2 |
| RaGOO_A02_101211 | A02 | 20950520 | 8.66E-09 | 0.154 | 104 | 0.692 | 0.814 | 1.57E-05 | 12.2 |
| RaGOO_A02_115852 | A02 | 24522129 | 8.67E-09 | 0.154 | 104 | 0.692 | 0.814 | 1.57E-05 | 12.2 |
| RaGOO_A02_118717 | A02 | 24971278 | 8.67E-09 | 0.159 | 104 | 0.692 | 0.814 | 1.57E-05 | 12.2 |
| RaGOO_A02_97994 | A02 | 20234278 | 8.69E-09 | 0.163 | 104 | 0.692 | 0.814 | 1.57E-05 | 12.2 |
| RaGOO_A02_82385 | A02 | 17225151 | 8.70E-09 | 0.154 | 104 | 0.692 | 0.814 | 1.57E-05 | 12.2 |
| RaGOO_A02_76751 | A02 | 16331410 | 8.71E-09 | 0.163 | 104 | 0.692 | 0.814 | 1.57E-05 | 12.2 |
| RaGOO_A02_111110 | A02 | 22934034 | 8.71E-09 | 0.159 | 104 | 0.692 | 0.814 | 1.57E-05 | 12.2 |
| RaGOO_A02_97832 | A02 | 20220636 | 8.72E-09 | 0.149 | 104 | 0.692 | 0.814 | 1.57E-05 | 12.2 |
| RaGOO_A02_91458 | A02 | 18844957 | 8.73E-09 | 0.163 | 104 | 0.692 | 0.814 | 1.57E-05 | 12.2 |
| RaGOO_A02_80837 | A02 | 16979850 | 8.74E-09 | 0.154 | 104 | 0.692 | 0.814 | 1.57E-05 | 12.2 |
| RaGOO_A02_110714 | A02 | 22853265 | 8.74E-09 | 0.163 | 104 | 0.692 | 0.814 | 1.57E-05 | 12.2 |
| RaGOO_A02_98153 | A02 | 20276554 | 8.75E-09 | 0.163 | 104 | 0.692 | 0.814 | 1.57E-05 | 12.2 |
| RaGOO_A02_91636 | A02 | 18857120 | 8.77E-09 | 0.212 | 104 | 0.692 | 0.814 | 1.57E-05 | 12.2 |
| RaGOO_A02_82230 | A02 | 17216840 | 8.77E-09 | 0.154 | 104 | 0.692 | 0.814 | 1.57E-05 | 12.2 |
| RaGOO_A02_85727 | A02 | 17724827 | 8.78E-09 | 0.154 | 104 | 0.692 | 0.814 | 1.57E-05 | 12.2 |
| RaGOO_A02_113741 | A02 | 24203437 | 8.80E-09 | 0.183 | 104 | 0.692 | 0.814 | 1.58E-05 | 12.2 |
| RaGOO_A02_102168 | A02 | 21085172 | 8.83E-09 | 0.149 | 104 | 0.692 | 0.814 | 1.58E-05 | 12.2 |
| RaGOO_A02_91860 | A02 | 18877821 | 8.84E-09 | 0.159 | 104 | 0.692 | 0.814 | 1.58E-05 | 12.2 |
| RaGOO_A02_120899 | A02 | 25415197 | 8.85E-09 | 0.231 | 104 | 0.692 | 0.814 | 1.58E-05 | 12.2 |
| RaGOO_A02_97182 | A02 | 20121289 | 8.87E-09 | 0.163 | 104 | 0.692 | 0.814 | 1.59E-05 | 12.2 |
| RaGOO_A02_108610 | A02 | 22425340 | 8.88E-09 | 0.178 | 104 | 0.692 | 0.814 | 1.59E-05 | 12.2 |
| RaGOO_A02_116175 | A02 | 24558790 | 8.89E-09 | 0.168 | 104 | 0.692 | 0.814 | 1.59E-05 | 12.2 |

| RaGOO_A02_90270 | A02 | 18617812 | 8.89E-09 | 0.173 | 104 | 0.692 | 0.814 | 1.59E-05 | 12.2 |
| --- | --- | --- | --- | --- | --- | --- | --- | --- | --- |
| RaGOO_A02_115844 | A02 | 24521576 | 8.90E-09 | 0.159 | 104 | 0.692 | 0.814 | 1.59E-05 | 12.2 |
| RaGOO_A02_84470 | A02 | 17503860 | 8.90E-09 | 0.163 | 104 | 0.692 | 0.814 | 1.59E-05 | 12.2 |
| RaGOO_A02_114638 | A02 | 24366296 | 8.91E-09 | 0.188 | 104 | 0.692 | 0.814 | 1.59E-05 | 12.2 |
| RaGOO_A02_99884 | A02 | 20629804 | 8.91E-09 | 0.168 | 104 | 0.692 | 0.814 | 1.59E-05 | 12.2 |
| RaGOO_A02_99096 | A02 | 20480544 | 8.93E-09 | 0.168 | 104 | 0.692 | 0.814 | 1.59E-05 | 12.2 |
| RaGOO_A02_105790 | A02 | 21886191 | 8.94E-09 | 0.159 | 104 | 0.692 | 0.814 | 1.59E-05 | 12.2 |
| RaGOO_A02_73292 | A02 | 15759554 | 8.95E-09 | 0.149 | 104 | 0.692 | 0.814 | 1.59E-05 | 12.2 |
| RaGOO_A02_90248 | A02 | 18616618 | 8.96E-09 | 0.173 | 104 | 0.692 | 0.814 | 1.59E-05 | 12.2 |
| RaGOO_A02_116654 | A02 | 24600964 | 8.97E-09 | 0.159 | 104 | 0.692 | 0.814 | 1.59E-05 | 12.2 |
| RaGOO_A02_104283 | A02 | 21558922 | 8.99E-09 | 0.168 | 104 | 0.692 | 0.814 | 1.60E-05 | 12.2 |
| RaGOO_A02_107502 | A02 | 22170442 | 9.01E-09 | 0.130 | 104 | 0.692 | 0.814 | 1.60E-05 | 12.2 |
| RaGOO_A02_76999 | A02 | 16416493 | 9.05E-09 | 0.149 | 104 | 0.692 | 0.813 | 1.61E-05 | 12.1 |
| RaGOO_A02_102609 | A02 | 21143994 | 9.06E-09 | 0.183 | 104 | 0.692 | 0.813 | 1.61E-05 | 12.1 |
| RaGOO_A02_90249 | A02 | 18616668 | 9.07E-09 | 0.168 | 104 | 0.692 | 0.813 | 1.61E-05 | 12.1 |
| RaGOO_A02_115854 | A02 | 24522397 | 9.07E-09 | 0.163 | 104 | 0.692 | 0.813 | 1.61E-05 | 12.1 |
| RaGOO_A02_117501 | A02 | 24749004 | 9.09E-09 | 0.173 | 104 | 0.692 | 0.813 | 1.61E-05 | 12.1 |
| RaGOO_A02_82224 | A02 | 17216480 | 9.09E-09 | 0.154 | 104 | 0.692 | 0.813 | 1.61E-05 | 12.1 |
| RaGOO_A02_110605 | A02 | 22839657 | 9.14E-09 | 0.154 | 104 | 0.692 | 0.813 | 1.62E-05 | 12.1 |
| RaGOO_A02_100977 | A02 | 20928697 | 9.18E-09 | 0.163 | 104 | 0.692 | 0.813 | 1.62E-05 | 12.1 |
| RaGOO_A02_112521 | A02 | 23949343 | 9.19E-09 | 0.159 | 104 | 0.692 | 0.813 | 1.62E-05 | 12.1 |
| RaGOO_A02_112522 | A02 | 23949356 | 9.19E-09 | 0.159 | 104 | 0.692 | 0.813 | 1.62E-05 | 12.1 |
| RaGOO_A02_98167 | A02 | 20278334 | 9.21E-09 | 0.163 | 104 | 0.692 | 0.813 | 1.62E-05 | 12.1 |
| RaGOO_A02_97072 | A02 | 20102235 | 9.23E-09 | 0.149 | 104 | 0.692 | 0.813 | 1.62E-05 | 12.1 |
| RaGOO_A02_75354 | A02 | 16179308 | 9.24E-09 | 0.154 | 104 | 0.692 | 0.813 | 1.62E-05 | 12.1 |
| RaGOO_A02_100419 | A02 | 20800945 | 9.24E-09 | 0.159 | 104 | 0.692 | 0.813 | 1.62E-05 | 12.1 |
| RaGOO_A02_99023 | A02 | 20475375 | 9.24E-09 | 0.154 | 104 | 0.692 | 0.813 | 1.62E-05 | 12.1 |
| RaGOO_A02_107638 | A02 | 22174991 | 9.24E-09 | 0.125 | 104 | 0.692 | 0.813 | 1.62E-05 | 12.1 |
| RaGOO_A02_105366 | A02 | 21808740 | 9.24E-09 | 0.154 | 104 | 0.692 | 0.813 | 1.62E-05 | 12.1 |
| RaGOO_A02_109483 | A02 | 22632448 | 9.25E-09 | 0.159 | 104 | 0.692 | 0.813 | 1.62E-05 | 12.1 |
| RaGOO_A02_78950 | A02 | 16769355 | 9.25E-09 | 0.149 | 104 | 0.692 | 0.813 | 1.62E-05 | 12.1 |
| RaGOO_A02_107332 | A02 | 22145313 | 9.26E-09 | 0.139 | 104 | 0.692 | 0.813 | 1.62E-05 | 12.1 |
| RaGOO_A02_110604 | A02 | 22839634 | 9.28E-09 | 0.149 | 104 | 0.692 | 0.813 | 1.63E-05 | 12.1 |
| RaGOO_A02_104269 | A02 | 21557927 | 9.29E-09 | 0.144 | 104 | 0.692 | 0.813 | 1.63E-05 | 12.1 |
| RaGOO_A02_107844 | A02 | 22195267 | 9.33E-09 | 0.183 | 104 | 0.692 | 0.813 | 1.63E-05 | 12.1 |
| RaGOO_A02_91723 | A02 | 18863342 | 9.33E-09 | 0.154 | 104 | 0.692 | 0.813 | 1.63E-05 | 12.1 |
| RaGOO_A02_89948 | A02 | 18581545 | 9.34E-09 | 0.159 | 104 | 0.692 | 0.813 | 1.63E-05 | 12.1 |
| RaGOO_A02_115799 | A02 | 24518176 | 9.35E-09 | 0.183 | 104 | 0.692 | 0.813 | 1.64E-05 | 12.1 |
| RaGOO_A02_113696 | A02 | 24196558 | 9.36E-09 | 0.154 | 104 | 0.692 | 0.813 | 1.64E-05 | 12.1 |
| RaGOO_A02_92309 | A02 | 18919344 | 9.36E-09 | 0.163 | 104 | 0.692 | 0.813 | 1.64E-05 | 12.1 |
| RaGOO_A02_117296 | A02 | 24724279 | 9.37E-09 | 0.144 | 104 | 0.692 | 0.813 | 1.64E-05 | 12.1 |
| RaGOO_A02_91797 | A02 | 18873908 | 9.37E-09 | 0.144 | 104 | 0.692 | 0.813 | 1.64E-05 | 12.1 |
| RaGOO_A02_117929 | A02 | 24824633 | 9.40E-09 | 0.163 | 104 | 0.692 | 0.813 | 1.64E-05 | 12.1 |
| RaGOO_A02_102997 | A02 | 21218980 | 9.43E-09 | 0.168 | 104 | 0.692 | 0.813 | 1.64E-05 | 12.1 |
| RaGOO_A02_96952 | A02 | 20086231 | 9.43E-09 | 0.168 | 104 | 0.692 | 0.813 | 1.64E-05 | 12.1 |
| RaGOO_A02_123591 | A02 | 25724170 | 9.43E-09 | 0.154 | 104 | 0.692 | 0.813 | 1.64E-05 | 12.1 |
| RaGOO_A02_99980 | A02 | 20662353 | 9.44E-09 | 0.144 | 104 | 0.692 | 0.813 | 1.64E-05 | 12.1 |
| RaGOO_A02_98981 | A02 | 20471875 | 9.49E-09 | 0.159 | 104 | 0.692 | 0.813 | 1.65E-05 | 12.1 |
| RaGOO_A02_116051 | A02 | 24546159 | 9.50E-09 | 0.159 | 104 | 0.692 | 0.813 | 1.65E-05 | 12.1 |
| RaGOO_A02_89821 | A02 | 18562373 | 9.50E-09 | 0.163 | 104 | 0.692 | 0.813 | 1.65E-05 | 12.1 |
| RaGOO_A02_89822 | A02 | 18562387 | 9.50E-09 | 0.163 | 104 | 0.692 | 0.813 | 1.65E-05 | 12.1 |
| RaGOO_A02_98050 | A02 | 20256260 | 9.51E-09 | 0.149 | 104 | 0.692 | 0.813 | 1.65E-05 | 12.1 |
| RaGOO_A02_116085 | A02 | 24547950 | 9.52E-09 | 0.163 | 104 | 0.692 | 0.813 | 1.65E-05 | 12.1 |
| RaGOO_A02_97969 | A02 | 20232170 | 9.53E-09 | 0.173 | 104 | 0.692 | 0.813 | 1.65E-05 | 12.1 |
| RaGOO_A02_89351 | A02 | 18484578 | 9.56E-09 | 0.168 | 104 | 0.692 | 0.813 | 1.66E-05 | 12.1 |
| RaGOO_A02_113277 | A02 | 24060425 | 9.57E-09 | 0.159 | 104 | 0.692 | 0.813 | 1.66E-05 | 12.1 |
| RaGOO_A02_84207 | A02 | 17445317 | 9.60E-09 | 0.168 | 104 | 0.692 | 0.813 | 1.66E-05 | 12.1 |
| RaGOO_A02_97593 | A02 | 20183730 | 9.64E-09 | 0.168 | 104 | 0.692 | 0.813 | 1.67E-05 | 12.1 |
| RaGOO_A02_85729 | A02 | 17724944 | 9.68E-09 | 0.144 | 104 | 0.692 | 0.813 | 1.67E-05 | 12.1 |
| RaGOO_A02_115404 | A02 | 24465774 | 9.69E-09 | 0.154 | 104 | 0.692 | 0.813 | 1.68E-05 | 12.1 |
| RaGOO_A02_114192 | A02 | 24294204 | 9.70E-09 | 0.163 | 104 | 0.692 | 0.813 | 1.68E-05 | 12.1 |
| RaGOO_A02_78903 | A02 | 16764197 | 9.72E-09 | 0.159 | 104 | 0.692 | 0.813 | 1.68E-05 | 12.1 |
| RaGOO_A02_116300 | A02 | 24568266 | 9.72E-09 | 0.159 | 104 | 0.692 | 0.813 | 1.68E-05 | 12.1 |
| RaGOO_A02_111557 | A02 | 23160070 | 9.72E-09 | 0.173 | 104 | 0.692 | 0.813 | 1.68E-05 | 12.1 |
| RaGOO_A02_115842 | A02 | 24521111 | 9.74E-09 | 0.168 | 104 | 0.692 | 0.813 | 1.68E-05 | 12.1 |
| RaGOO_A02_111664 | A02 | 23173796 | 9.75E-09 | 0.159 | 104 | 0.692 | 0.813 | 1.68E-05 | 12.1 |
| RaGOO_A02_110646 | A02 | 22843430 | 9.77E-09 | 0.154 | 104 | 0.692 | 0.813 | 1.68E-05 | 12.1 |
| RaGOO_A02_77003 | A02 | 16416684 | 9.77E-09 | 0.168 | 104 | 0.692 | 0.813 | 1.68E-05 | 12.1 |
| RaGOO_A02_112091 | A02 | 23883347 | 9.77E-09 | 0.159 | 104 | 0.692 | 0.813 | 1.68E-05 | 12.1 |
| RaGOO_A02_92218 | A02 | 18912519 | 9.79E-09 | 0.139 | 104 | 0.692 | 0.813 | 1.68E-05 | 12.1 |
| RaGOO_A02_89868 | A02 | 18568479 | 9.79E-09 | 0.168 | 104 | 0.692 | 0.813 | 1.68E-05 | 12.1 |
| RaGOO_A02_102690 | A02 | 21151851 | 9.82E-09 | 0.163 | 104 | 0.692 | 0.813 | 1.69E-05 | 12.1 |
| RaGOO_A02_98107 | A02 | 20271010 | 9.83E-09 | 0.159 | 104 | 0.692 | 0.813 | 1.69E-05 | 12.1 |
| RaGOO_A02_114099 | A02 | 24259144 | 9.84E-09 | 0.163 | 104 | 0.692 | 0.813 | 1.69E-05 | 12.1 |
| RaGOO_A02_96825 | A02 | 20074348 | 9.86E-09 | 0.183 | 104 | 0.692 | 0.813 | 1.69E-05 | 12.1 |
| RaGOO_A02_86937 | A02 | 17983767 | 9.90E-09 | 0.163 | 104 | 0.692 | 0.813 | 1.69E-05 | 12.1 |
| RaGOO_A02_121961 | A02 | 25547730 | 9.90E-09 | 0.245 | 104 | 0.692 | 0.813 | 1.69E-05 | 12.1 |
| RaGOO_A02_97488 | A02 | 20170470 | 9.91E-09 | 0.159 | 104 | 0.692 | 0.813 | 1.69E-05 | 12.1 |
| RaGOO_A02_104262 | A02 | 21555932 | 9.91E-09 | 0.149 | 104 | 0.692 | 0.813 | 1.69E-05 | 12.1 |
| RaGOO_A02_97358 | A02 | 20152538 | 9.92E-09 | 0.173 | 104 | 0.692 | 0.813 | 1.69E-05 | 12.1 |
| RaGOO_A02_107317 | A02 | 22144922 | 9.95E-09 | 0.120 | 104 | 0.692 | 0.813 | 1.70E-05 | 12.1 |
| RaGOO_A02_96622 | A02 | 20036323 | 9.95E-09 | 0.178 | 104 | 0.692 | 0.813 | 1.70E-05 | 12.1 |
| RaGOO_A02_105396 | A02 | 21816345 | 9.98E-09 | 0.163 | 104 | 0.692 | 0.813 | 1.70E-05 | 12.1 |
| RaGOO_A02_78866 | A02 | 16759201 | 9.98E-09 | 0.154 | 104 | 0.692 | 0.813 | 1.70E-05 | 12.1 |
| RaGOO_A02_106105 | A02 | 21925772 | 9.99E-09 | 0.159 | 104 | 0.692 | 0.813 | 1.70E-05 | 12.1 |
| RaGOO_A02_89870 | A02 | 18568700 | 1.00E-08 | 0.159 | 104 | 0.692 | 0.813 | 1.71E-05 | 12.1 |
| RaGOO_A02_102118 | A02 | 21076111 | 1.01E-08 | 0.149 | 104 | 0.692 | 0.813 | 1.71E-05 | 12.1 |
| RaGOO_A02_101208 | A02 | 20950369 | 1.01E-08 | 0.168 | 104 | 0.692 | 0.813 | 1.71E-05 | 12.1 |
| RaGOO_A02_99608 | A02 | 20555658 | 1.01E-08 | 0.168 | 104 | 0.692 | 0.813 | 1.71E-05 | 12.1 |
| RaGOO_A02_96068 | A02 | 19954329 | 1.01E-08 | 0.159 | 104 | 0.692 | 0.813 | 1.71E-05 | 12.1 |
| RaGOO_A02_82352 | A02 | 17223451 | 1.01E-08 | 0.159 | 104 | 0.692 | 0.813 | 1.71E-05 | 12.1 |

| RaGOO_A02_89234 | A02 | 18458193 | 1.01E-08 | 0.163 | 104 | 0.692 | 0.813 | 1.71E-05 | 12.1 |
| --- | --- | --- | --- | --- | --- | --- | --- | --- | --- |
| RaGOO_A02_98040 | A02 | 20255250 | 1.01E-08 | 0.163 | 104 | 0.692 | 0.813 | 1.72E-05 | 12.1 |
| RaGOO_A02_111819 | A02 | 23726331 | 1.01E-08 | 0.226 | 104 | 0.692 | 0.813 | 1.72E-05 | 12.1 |
| RaGOO_A02_92060 | A02 | 18897635 | 1.02E-08 | 0.154 | 104 | 0.692 | 0.812 | 1.72E-05 | 12.0 |
| RaGOO_A02_117667 | A02 | 24795723 | 1.02E-08 | 0.159 | 104 | 0.692 | 0.812 | 1.72E-05 | 12.0 |
| RaGOO_A02_99044 | A02 | 20476400 | 1.02E-08 | 0.149 | 104 | 0.692 | 0.812 | 1.73E-05 | 12.0 |
| RaGOO_A02_105863 | A02 | 21896921 | 1.02E-08 | 0.163 | 104 | 0.692 | 0.812 | 1.73E-05 | 12.0 |
| RaGOO_A02_117508 | A02 | 24750867 | 1.02E-08 | 0.149 | 104 | 0.692 | 0.812 | 1.73E-05 | 12.0 |
| RaGOO_A02_96546 | A02 | 20025173 | 1.02E-08 | 0.154 | 104 | 0.692 | 0.812 | 1.73E-05 | 12.0 |
| RaGOO_A02_73209 | A02 | 15741772 | 1.02E-08 | 0.154 | 104 | 0.692 | 0.812 | 1.73E-05 | 12.0 |
| RaGOO_A02_88544 | A02 | 18261213 | 1.03E-08 | 0.168 | 104 | 0.692 | 0.812 | 1.73E-05 | 12.0 |
| RaGOO_A02_97481 | A02 | 20170202 | 1.03E-08 | 0.159 | 104 | 0.692 | 0.812 | 1.74E-05 | 12.0 |
| RaGOO_A02_108577 | A02 | 22423246 | 1.03E-08 | 0.168 | 104 | 0.692 | 0.812 | 1.74E-05 | 12.0 |
| RaGOO_A02_72298 | A02 | 15535183 | 1.03E-08 | 0.178 | 104 | 0.692 | 0.812 | 1.74E-05 | 12.0 |
| RaGOO_A02_111555 | A02 | 23159715 | 1.03E-08 | 0.173 | 104 | 0.692 | 0.812 | 1.74E-05 | 12.0 |
| RaGOO_A02_112536 | A02 | 23949841 | 1.03E-08 | 0.168 | 104 | 0.692 | 0.812 | 1.74E-05 | 12.0 |
| RaGOO_A02_102587 | A02 | 21142268 | 1.03E-08 | 0.149 | 104 | 0.692 | 0.812 | 1.74E-05 | 12.0 |
| RaGOO_A02_79615 | A02 | 16857322 | 1.03E-08 | 0.149 | 104 | 0.692 | 0.812 | 1.74E-05 | 12.0 |
| RaGOO_A02_73028 | A02 | 15715441 | 1.05E-08 | 0.178 | 104 | 0.692 | 0.812 | 1.76E-05 | 12.0 |
| RaGOO_A02_118195 | A02 | 24863018 | 1.05E-08 | 0.168 | 104 | 0.692 | 0.812 | 1.76E-05 | 12.0 |
| RaGOO_A02_99696 | A02 | 20579753 | 1.05E-08 | 0.178 | 104 | 0.692 | 0.812 | 1.76E-05 | 12.0 |
| RaGOO_A02_109992 | A02 | 22751435 | 1.05E-08 | 0.163 | 104 | 0.692 | 0.812 | 1.76E-05 | 12.0 |
| RaGOO_A02_107977 | A02 | 22202592 | 1.05E-08 | 0.168 | 104 | 0.692 | 0.812 | 1.76E-05 | 12.0 |
| RaGOO_A02_116188 | A02 | 24559225 | 1.05E-08 | 0.173 | 104 | 0.692 | 0.812 | 1.77E-05 | 12.0 |
| RaGOO_A02_96456 | A02 | 20006666 | 1.05E-08 | 0.168 | 104 | 0.692 | 0.812 | 1.77E-05 | 12.0 |
| RaGOO_A02_84538 | A02 | 17510670 | 1.06E-08 | 0.159 | 104 | 0.692 | 0.812 | 1.77E-05 | 12.0 |
| RaGOO_A02_80141 | A02 | 16917600 | 1.06E-08 | 0.163 | 104 | 0.692 | 0.812 | 1.78E-05 | 12.0 |
| RaGOO_A02_102182 | A02 | 21091746 | 1.06E-08 | 0.149 | 104 | 0.692 | 0.812 | 1.78E-05 | 12.0 |
| RaGOO_A02_88687 | A02 | 18273600 | 1.06E-08 | 0.159 | 104 | 0.692 | 0.812 | 1.78E-05 | 12.0 |
| RaGOO_A02_98002 | A02 | 20234873 | 1.07E-08 | 0.154 | 104 | 0.692 | 0.812 | 1.78E-05 | 12.0 |
| RaGOO_A02_98001 | A02 | 20234871 | 1.07E-08 | 0.154 | 104 | 0.692 | 0.812 | 1.78E-05 | 12.0 |
| RaGOO_A02_98027 | A02 | 20253802 | 1.07E-08 | 0.154 | 104 | 0.692 | 0.812 | 1.79E-05 | 12.0 |
| RaGOO_A02_80184 | A02 | 16920804 | 1.07E-08 | 0.135 | 104 | 0.692 | 0.812 | 1.79E-05 | 12.0 |
| RaGOO_A02_91834 | A02 | 18876143 | 1.08E-08 | 0.212 | 104 | 0.692 | 0.812 | 1.81E-05 | 12.0 |
| RaGOO_A02_113109 | A02 | 24036311 | 1.08E-08 | 0.163 | 104 | 0.692 | 0.812 | 1.81E-05 | 12.0 |
| RaGOO_A02_108568 | A02 | 22422663 | 1.08E-08 | 0.163 | 104 | 0.692 | 0.812 | 1.81E-05 | 12.0 |
| RaGOO_A02_111665 | A02 | 23173798 | 1.09E-08 | 0.163 | 104 | 0.692 | 0.812 | 1.81E-05 | 12.0 |
| RaGOO_A02_106130 | A02 | 21929300 | 1.09E-08 | 0.163 | 104 | 0.692 | 0.812 | 1.81E-05 | 12.0 |
| RaGOO_A02_82307 | A02 | 17220396 | 1.09E-08 | 0.168 | 104 | 0.692 | 0.812 | 1.81E-05 | 12.0 |
| RaGOO_A02_114844 | A02 | 24387756 | 1.09E-08 | 0.149 | 104 | 0.692 | 0.812 | 1.81E-05 | 12.0 |
| RaGOO_A02_85037 | A02 | 17601376 | 1.09E-08 | 0.149 | 104 | 0.692 | 0.812 | 1.81E-05 | 12.0 |
| RaGOO_A02_82275 | A02 | 17218835 | 1.09E-08 | 0.159 | 104 | 0.692 | 0.812 | 1.81E-05 | 12.0 |
| RaGOO_A02_112519 | A02 | 23949210 | 1.10E-08 | 0.159 | 104 | 0.692 | 0.812 | 1.82E-05 | 12.0 |
| RaGOO_A02_99818 | A02 | 20611029 | 1.10E-08 | 0.154 | 104 | 0.692 | 0.812 | 1.82E-05 | 12.0 |
| RaGOO_A02_80637 | A02 | 16962675 | 1.10E-08 | 0.144 | 104 | 0.692 | 0.812 | 1.82E-05 | 12.0 |
| RaGOO_A02_114705 | A02 | 24370739 | 1.10E-08 | 0.168 | 104 | 0.692 | 0.812 | 1.82E-05 | 12.0 |
| RaGOO_A02_80185 | A02 | 16920871 | 1.10E-08 | 0.149 | 104 | 0.692 | 0.812 | 1.82E-05 | 12.0 |
| RaGOO_A02_105795 | A02 | 21886386 | 1.10E-08 | 0.144 | 104 | 0.692 | 0.812 | 1.82E-05 | 12.0 |
| RaGOO_A02_102990 | A02 | 21217831 | 1.10E-08 | 0.163 | 104 | 0.692 | 0.812 | 1.82E-05 | 12.0 |
| RaGOO_A02_116081 | A02 | 24547826 | 1.11E-08 | 0.173 | 104 | 0.692 | 0.812 | 1.83E-05 | 12.0 |
| RaGOO_A02_80139 | A02 | 16917489 | 1.11E-08 | 0.154 | 104 | 0.692 | 0.812 | 1.84E-05 | 12.0 |
| RaGOO_A02_91564 | A02 | 18853580 | 1.11E-08 | 0.192 | 104 | 0.692 | 0.812 | 1.84E-05 | 12.0 |
| RaGOO_A02_115802 | A02 | 24518351 | 1.12E-08 | 0.183 | 104 | 0.692 | 0.812 | 1.84E-05 | 12.0 |
| RaGOO_A02_82295 | A02 | 17219867 | 1.12E-08 | 0.159 | 104 | 0.692 | 0.812 | 1.84E-05 | 12.0 |
| RaGOO_A02_78199 | A02 | 16650017 | 1.12E-08 | 0.159 | 104 | 0.692 | 0.812 | 1.84E-05 | 12.0 |
| RaGOO_A02_110259 | A02 | 22786058 | 1.12E-08 | 0.154 | 104 | 0.692 | 0.812 | 1.84E-05 | 12.0 |
| RaGOO_A02_114997 | A02 | 24409953 | 1.12E-08 | 0.163 | 104 | 0.692 | 0.812 | 1.84E-05 | 12.0 |
| RaGOO_A02_97754 | A02 | 20200978 | 1.12E-08 | 0.144 | 104 | 0.692 | 0.812 | 1.84E-05 | 12.0 |
| RaGOO_A02_77717 | A02 | 16565241 | 1.12E-08 | 0.139 | 104 | 0.692 | 0.812 | 1.85E-05 | 12.0 |
| RaGOO_A02_113239 | A02 | 24059014 | 1.12E-08 | 0.159 | 104 | 0.692 | 0.812 | 1.85E-05 | 12.0 |
| RaGOO_A02_80630 | A02 | 16962183 | 1.12E-08 | 0.154 | 104 | 0.692 | 0.812 | 1.85E-05 | 12.0 |
| RaGOO_A02_92358 | A02 | 18926049 | 1.13E-08 | 0.159 | 104 | 0.692 | 0.812 | 1.85E-05 | 12.0 |
| RaGOO_A02_117927 | A02 | 24824468 | 1.13E-08 | 0.173 | 104 | 0.692 | 0.812 | 1.85E-05 | 12.0 |
| RaGOO_A02_80773 | A02 | 16975963 | 1.13E-08 | 0.168 | 104 | 0.692 | 0.812 | 1.85E-05 | 12.0 |
| RaGOO_A02_117714 | A02 | 24800981 | 1.13E-08 | 0.159 | 104 | 0.692 | 0.812 | 1.85E-05 | 12.0 |
| RaGOO_A02_110597 | A02 | 22839310 | 1.13E-08 | 0.159 | 104 | 0.692 | 0.812 | 1.85E-05 | 12.0 |
| RaGOO_A02_91922 | A02 | 18883072 | 1.13E-08 | 0.168 | 104 | 0.692 | 0.812 | 1.85E-05 | 12.0 |
| RaGOO_A02_90234 | A02 | 18615653 | 1.13E-08 | 0.159 | 104 | 0.692 | 0.812 | 1.86E-05 | 12.0 |
| RaGOO_A02_77498 | A02 | 16509594 | 1.14E-08 | 0.149 | 104 | 0.692 | 0.812 | 1.87E-05 | 12.0 |
| RaGOO_A02_104244 | A02 | 21553369 | 1.15E-08 | 0.173 | 104 | 0.692 | 0.812 | 1.87E-05 | 11.9 |
| RaGOO_A02_86619 | A02 | 17865299 | 1.15E-08 | 0.154 | 104 | 0.692 | 0.812 | 1.87E-05 | 11.9 |
| RaGOO_A02_102481 | A02 | 21129129 | 1.15E-08 | 0.154 | 104 | 0.692 | 0.812 | 1.87E-05 | 11.9 |
| RaGOO_A02_94531 | A02 | 19289836 | 1.15E-08 | 0.106 | 104 | 0.692 | 0.812 | 1.87E-05 | 11.9 |
| RaGOO_A02_108975 | A02 | 22549386 | 1.15E-08 | 0.159 | 104 | 0.692 | 0.811 | 1.87E-05 | 11.9 |
| RaGOO_A02_99796 | A02 | 20606985 | 1.15E-08 | 0.149 | 104 | 0.692 | 0.811 | 1.87E-05 | 11.9 |
| RaGOO_A02_72261 | A02 | 15531891 | 1.15E-08 | 0.163 | 104 | 0.692 | 0.811 | 1.87E-05 | 11.9 |
| RaGOO_A02_109453 | A02 | 22625877 | 1.15E-08 | 0.159 | 104 | 0.692 | 0.811 | 1.87E-05 | 11.9 |
| RaGOO_A02_73146 | A02 | 15737121 | 1.15E-08 | 0.149 | 104 | 0.692 | 0.811 | 1.87E-05 | 11.9 |
| RaGOO_A02_98045 | A02 | 20255820 | 1.15E-08 | 0.163 | 104 | 0.692 | 0.811 | 1.88E-05 | 11.9 |
| RaGOO_A02_70688 | A02 | 15209406 | 1.15E-08 | 0.159 | 104 | 0.692 | 0.811 | 1.88E-05 | 11.9 |
| RaGOO_A02_75309 | A02 | 16173750 | 1.15E-08 | 0.168 | 104 | 0.692 | 0.811 | 1.88E-05 | 11.9 |
| RaGOO_A02_109668 | A02 | 22658010 | 1.15E-08 | 0.159 | 104 | 0.692 | 0.811 | 1.88E-05 | 11.9 |
| RaGOO_A02_116194 | A02 | 24559591 | 1.16E-08 | 0.173 | 104 | 0.692 | 0.811 | 1.88E-05 | 11.9 |
| RaGOO_A02_105450 | A02 | 21825365 | 1.16E-08 | 0.163 | 104 | 0.692 | 0.811 | 1.88E-05 | 11.9 |
| RaGOO_A02_86122 | A02 | 17792619 | 1.17E-08 | 0.192 | 104 | 0.692 | 0.811 | 1.89E-05 | 11.9 |
| RaGOO_A02_112667 | A02 | 23966612 | 1.17E-08 | 0.163 | 104 | 0.692 | 0.811 | 1.90E-05 | 11.9 |
| RaGOO_A02_114707 | A02 | 24370806 | 1.17E-08 | 0.183 | 104 | 0.692 | 0.811 | 1.90E-05 | 11.9 |
| RaGOO_A02_117713 | A02 | 24800950 | 1.17E-08 | 0.149 | 104 | 0.692 | 0.811 | 1.90E-05 | 11.9 |
| RaGOO_A02_108565 | A02 | 22422567 | 1.18E-08 | 0.159 | 104 | 0.692 | 0.811 | 1.91E-05 | 11.9 |
| RaGOO_A02_110398 | A02 | 22811057 | 1.18E-08 | 0.159 | 104 | 0.692 | 0.811 | 1.91E-05 | 11.9 |
| RaGOO_A02_102871 | A02 | 21173615 | 1.18E-08 | 0.178 | 104 | 0.692 | 0.811 | 1.91E-05 | 11.9 |

| RaGOO_A02_91730 | A02 | 18863977 | 1.18E-08 | 0.173 | 104 | 0.692 | 0.811 | 1.91E-05 | 11.9 |
| --- | --- | --- | --- | --- | --- | --- | --- | --- | --- |
| RaGOO_A02_123249 | A02 | 25693003 | 1.19E-08 | 0.149 | 104 | 0.692 | 0.811 | 1.92E-05 | 11.9 |
| RaGOO_A02_118548 | A02 | 24935330 | 1.19E-08 | 0.159 | 104 | 0.692 | 0.811 | 1.92E-05 | 11.9 |
| RaGOO_A02_89700 | A02 | 18549248 | 1.20E-08 | 0.159 | 104 | 0.692 | 0.811 | 1.93E-05 | 11.9 |
| RaGOO_A02_102333 | A02 | 21109967 | 1.20E-08 | 0.144 | 104 | 0.692 | 0.811 | 1.94E-05 | 11.9 |
| RaGOO_A02_111223 | A02 | 22947638 | 1.20E-08 | 0.154 | 104 | 0.692 | 0.811 | 1.94E-05 | 11.9 |
| RaGOO_A02_110750 | A02 | 22877316 | 1.20E-08 | 0.168 | 104 | 0.692 | 0.811 | 1.94E-05 | 11.9 |
| RaGOO_A02_91979 | A02 | 18888008 | 1.20E-08 | 0.173 | 104 | 0.692 | 0.811 | 1.94E-05 | 11.9 |
| RaGOO_A02_112045 | A02 | 23878829 | 1.20E-08 | 0.173 | 104 | 0.692 | 0.811 | 1.94E-05 | 11.9 |
| RaGOO_A02_97063 | A02 | 20101703 | 1.20E-08 | 0.149 | 104 | 0.692 | 0.811 | 1.94E-05 | 11.9 |
| RaGOO_A02_105945 | A02 | 21910173 | 1.21E-08 | 0.154 | 104 | 0.692 | 0.811 | 1.94E-05 | 11.9 |
| RaGOO_A02_91474 | A02 | 18845800 | 1.21E-08 | 0.163 | 104 | 0.692 | 0.811 | 1.94E-05 | 11.9 |
| RaGOO_A02_83076 | A02 | 17300956 | 1.21E-08 | 0.149 | 104 | 0.692 | 0.811 | 1.94E-05 | 11.9 |
| RaGOO_A02_84512 | A02 | 17508679 | 1.22E-08 | 0.163 | 104 | 0.692 | 0.811 | 1.96E-05 | 11.9 |
| RaGOO_A02_92215 | A02 | 18912309 | 1.22E-08 | 0.149 | 104 | 0.692 | 0.811 | 1.96E-05 | 11.9 |
| RaGOO_A02_98006 | A02 | 20235093 | 1.22E-08 | 0.149 | 104 | 0.692 | 0.811 | 1.96E-05 | 11.9 |
| RaGOO_A02_117678 | A02 | 24797258 | 1.22E-08 | 0.149 | 104 | 0.692 | 0.811 | 1.96E-05 | 11.9 |
| RaGOO_A02_117506 | A02 | 24750843 | 1.22E-08 | 0.188 | 104 | 0.692 | 0.811 | 1.96E-05 | 11.9 |
| RaGOO_A02_97695 | A02 | 20191055 | 1.22E-08 | 0.159 | 104 | 0.692 | 0.811 | 1.96E-05 | 11.9 |
| RaGOO_A02_107885 | A02 | 22200017 | 1.23E-08 | 0.120 | 104 | 0.692 | 0.811 | 1.96E-05 | 11.9 |
| RaGOO_A02_116070 | A02 | 24547095 | 1.23E-08 | 0.149 | 104 | 0.692 | 0.811 | 1.96E-05 | 11.9 |
| RaGOO_A02_115759 | A02 | 24514934 | 1.23E-08 | 0.178 | 104 | 0.692 | 0.811 | 1.96E-05 | 11.9 |
| RaGOO_A02_89550 | A02 | 18527808 | 1.23E-08 | 0.159 | 104 | 0.692 | 0.811 | 1.96E-05 | 11.9 |
| RaGOO_A02_120776 | A02 | 25408684 | 1.23E-08 | 0.159 | 104 | 0.692 | 0.811 | 1.96E-05 | 11.9 |
| RaGOO_A02_108620 | A02 | 22425760 | 1.23E-08 | 0.159 | 104 | 0.692 | 0.811 | 1.97E-05 | 11.9 |
| RaGOO_A02_103095 | A02 | 21230189 | 1.23E-08 | 0.159 | 104 | 0.692 | 0.811 | 1.97E-05 | 11.9 |
| RaGOO_A02_108482 | A02 | 22397129 | 1.23E-08 | 0.168 | 104 | 0.692 | 0.811 | 1.97E-05 | 11.9 |
| RaGOO_A02_100411 | A02 | 20798060 | 1.23E-08 | 0.139 | 104 | 0.692 | 0.811 | 1.97E-05 | 11.9 |
| RaGOO_A02_120439 | A02 | 25350359 | 1.24E-08 | 0.322 | 104 | 0.692 | 0.811 | 1.97E-05 | 11.9 |
| RaGOO_A02_82276 | A02 | 17218837 | 1.24E-08 | 0.154 | 104 | 0.692 | 0.811 | 1.97E-05 | 11.9 |
| RaGOO_A02_117692 | A02 | 24798515 | 1.24E-08 | 0.173 | 104 | 0.692 | 0.811 | 1.98E-05 | 11.9 |
| RaGOO_A02_117739 | A02 | 24802767 | 1.24E-08 | 0.173 | 104 | 0.692 | 0.811 | 1.98E-05 | 11.9 |
| RaGOO_A02_96312 | A02 | 19991621 | 1.25E-08 | 0.168 | 104 | 0.692 | 0.811 | 1.98E-05 | 11.9 |
| RaGOO_A02_108621 | A02 | 22425801 | 1.25E-08 | 0.154 | 104 | 0.692 | 0.811 | 1.98E-05 | 11.9 |
| RaGOO_A02_96070 | A02 | 19954687 | 1.25E-08 | 0.163 | 104 | 0.692 | 0.811 | 1.98E-05 | 11.9 |
| RaGOO_A02_101059 | A02 | 20936200 | 1.25E-08 | 0.168 | 104 | 0.692 | 0.811 | 1.99E-05 | 11.9 |
| RaGOO_A02_99634 | A02 | 20564618 | 1.25E-08 | 0.163 | 104 | 0.692 | 0.811 | 1.99E-05 | 11.9 |
| RaGOO_A02_102277 | A02 | 21103603 | 1.26E-08 | 0.188 | 104 | 0.692 | 0.811 | 1.99E-05 | 11.9 |
| RaGOO_A02_97778 | A02 | 20203465 | 1.26E-08 | 0.149 | 104 | 0.692 | 0.811 | 1.99E-05 | 11.9 |
| RaGOO_A02_78047 | A02 | 16631077 | 1.26E-08 | 0.135 | 104 | 0.692 | 0.811 | 1.99E-05 | 11.9 |
| RaGOO_A02_106739 | A02 | 22007968 | 1.26E-08 | 0.183 | 104 | 0.692 | 0.811 | 2.00E-05 | 11.9 |
| RaGOO_A02_110560 | A02 | 22834689 | 1.26E-08 | 0.173 | 104 | 0.692 | 0.811 | 2.00E-05 | 11.9 |
| RaGOO_A02_101567 | A02 | 20991865 | 1.26E-08 | 0.173 | 104 | 0.692 | 0.811 | 2.00E-05 | 11.9 |
| RaGOO_A02_88808 | A02 | 18285461 | 1.27E-08 | 0.178 | 104 | 0.692 | 0.811 | 2.00E-05 | 11.9 |
| RaGOO_A02_83549 | A02 | 17351231 | 1.27E-08 | 0.168 | 104 | 0.692 | 0.811 | 2.01E-05 | 11.9 |
| RaGOO_A02_73151 | A02 | 15737398 | 1.27E-08 | 0.159 | 104 | 0.692 | 0.811 | 2.01E-05 | 11.9 |
| RaGOO_A02_76659 | A02 | 16326384 | 1.27E-08 | 0.149 | 104 | 0.692 | 0.811 | 2.01E-05 | 11.9 |
| RaGOO_A02_73318 | A02 | 15763654 | 1.28E-08 | 0.139 | 104 | 0.692 | 0.811 | 2.02E-05 | 11.9 |
| RaGOO_A02_104285 | A02 | 21559078 | 1.28E-08 | 0.163 | 104 | 0.692 | 0.811 | 2.02E-05 | 11.9 |
| RaGOO_A02_114498 | A02 | 24332377 | 1.28E-08 | 0.173 | 104 | 0.692 | 0.811 | 2.02E-05 | 11.9 |
| RaGOO_A02_86234 | A02 | 17803093 | 1.28E-08 | 0.163 | 104 | 0.692 | 0.811 | 2.02E-05 | 11.9 |
| RaGOO_A02_115571 | A02 | 24481507 | 1.28E-08 | 0.163 | 104 | 0.692 | 0.811 | 2.02E-05 | 11.9 |
| RaGOO_A02_115794 | A02 | 24517756 | 1.28E-08 | 0.168 | 104 | 0.692 | 0.811 | 2.02E-05 | 11.9 |
| RaGOO_A02_107841 | A02 | 22195206 | 1.28E-08 | 0.149 | 104 | 0.692 | 0.811 | 2.02E-05 | 11.9 |
| RaGOO_A02_75351 | A02 | 16178846 | 1.29E-08 | 0.163 | 104 | 0.692 | 0.811 | 2.03E-05 | 11.9 |
| RaGOO_A02_79244 | A02 | 16809972 | 1.29E-08 | 0.163 | 104 | 0.692 | 0.811 | 2.03E-05 | 11.9 |
| RaGOO_A02_107857 | A02 | 22198649 | 1.29E-08 | 0.139 | 104 | 0.692 | 0.811 | 2.03E-05 | 11.9 |
| RaGOO_A02_90168 | A02 | 18607760 | 1.29E-08 | 0.159 | 104 | 0.692 | 0.811 | 2.03E-05 | 11.9 |
| RaGOO_A02_89699 | A02 | 18549044 | 1.29E-08 | 0.149 | 104 | 0.692 | 0.811 | 2.03E-05 | 11.9 |
| RaGOO_A02_115265 | A02 | 24438747 | 1.29E-08 | 0.188 | 104 | 0.692 | 0.811 | 2.03E-05 | 11.8 |
| RaGOO_A02_114592 | A02 | 24363871 | 1.30E-08 | 0.173 | 104 | 0.692 | 0.810 | 2.04E-05 | 11.8 |
| RaGOO_A02_108147 | A02 | 22337805 | 1.30E-08 | 0.159 | 104 | 0.692 | 0.810 | 2.04E-05 | 11.8 |
| RaGOO_A02_105949 | A02 | 21910626 | 1.30E-08 | 0.154 | 104 | 0.692 | 0.810 | 2.04E-05 | 11.8 |
| RaGOO_A02_99846 | A02 | 20615957 | 1.30E-08 | 0.149 | 104 | 0.692 | 0.810 | 2.04E-05 | 11.8 |
| RaGOO_A02_102324 | A02 | 21109380 | 1.31E-08 | 0.149 | 104 | 0.692 | 0.810 | 2.04E-05 | 11.8 |
| RaGOO_A02_83628 | A02 | 17374443 | 1.31E-08 | 0.154 | 104 | 0.692 | 0.810 | 2.05E-05 | 11.8 |
| RaGOO_A02_114983 | A02 | 24408764 | 1.31E-08 | 0.159 | 104 | 0.692 | 0.810 | 2.06E-05 | 11.8 |
| RaGOO_A02_96203 | A02 | 19981447 | 1.32E-08 | 0.168 | 104 | 0.692 | 0.810 | 2.06E-05 | 11.8 |
| RaGOO_A02_80870 | A02 | 16981536 | 1.32E-08 | 0.173 | 104 | 0.692 | 0.810 | 2.06E-05 | 11.8 |
| RaGOO_A02_110216 | A02 | 22780779 | 1.32E-08 | 0.159 | 104 | 0.692 | 0.810 | 2.07E-05 | 11.8 |
| RaGOO_A02_98827 | A02 | 20450685 | 1.33E-08 | 0.173 | 104 | 0.692 | 0.810 | 2.07E-05 | 11.8 |
| RaGOO_A02_102614 | A02 | 21144376 | 1.33E-08 | 0.178 | 104 | 0.692 | 0.810 | 2.08E-05 | 11.8 |
| RaGOO_A02_97993 | A02 | 20234191 | 1.34E-08 | 0.159 | 104 | 0.692 | 0.810 | 2.09E-05 | 11.8 |
| RaGOO_A02_118926 | A02 | 24993889 | 1.34E-08 | 0.135 | 104 | 0.692 | 0.810 | 2.09E-05 | 11.8 |
| RaGOO_A02_97611 | A02 | 20184975 | 1.34E-08 | 0.168 | 104 | 0.692 | 0.810 | 2.09E-05 | 11.8 |
| RaGOO_A02_96828 | A02 | 20074661 | 1.34E-08 | 0.183 | 104 | 0.692 | 0.810 | 2.09E-05 | 11.8 |
| RaGOO_A02_92065 | A02 | 18897907 | 1.35E-08 | 0.159 | 104 | 0.692 | 0.810 | 2.10E-05 | 11.8 |
| RaGOO_A02_114137 | A02 | 24269654 | 1.35E-08 | 0.197 | 104 | 0.692 | 0.810 | 2.10E-05 | 11.8 |
| RaGOO_A02_100961 | A02 | 20927414 | 1.35E-08 | 0.159 | 104 | 0.692 | 0.810 | 2.10E-05 | 11.8 |
| RaGOO_A02_100242 | A02 | 20736696 | 1.35E-08 | 0.154 | 104 | 0.692 | 0.810 | 2.10E-05 | 11.8 |
| RaGOO_A02_77180 | A02 | 16446914 | 1.35E-08 | 0.144 | 104 | 0.692 | 0.810 | 2.10E-05 | 11.8 |
| RaGOO_A02_77181 | A02 | 16446920 | 1.35E-08 | 0.144 | 104 | 0.692 | 0.810 | 2.10E-05 | 11.8 |
| RaGOO_A02_108562 | A02 | 22422144 | 1.35E-08 | 0.159 | 104 | 0.692 | 0.810 | 2.10E-05 | 11.8 |
| RaGOO_A02_79871 | A02 | 16883883 | 1.36E-08 | 0.154 | 104 | 0.692 | 0.810 | 2.10E-05 | 11.8 |
| RaGOO_A02_114089 | A02 | 24258415 | 1.37E-08 | 0.178 | 104 | 0.692 | 0.810 | 2.12E-05 | 11.8 |
| RaGOO_A02_110428 | A02 | 22814662 | 1.37E-08 | 0.159 | 104 | 0.692 | 0.810 | 2.12E-05 | 11.8 |
| RaGOO_A02_87139 | A02 | 18012224 | 1.37E-08 | 0.154 | 104 | 0.692 | 0.810 | 2.12E-05 | 11.8 |
| RaGOO_A02_87138 | A02 | 18012223 | 1.37E-08 | 0.154 | 104 | 0.692 | 0.810 | 2.12E-05 | 11.8 |
| RaGOO_A02_100718 | A02 | 20861971 | 1.37E-08 | 0.149 | 104 | 0.692 | 0.810 | 2.12E-05 | 11.8 |
| RaGOO_A02_97269 | A02 | 20136591 | 1.37E-08 | 0.168 | 104 | 0.692 | 0.810 | 2.12E-05 | 11.8 |
| RaGOO_A02_99281 | A02 | 20505537 | 1.38E-08 | 0.154 | 104 | 0.692 | 0.810 | 2.13E-05 | 11.8 |

| RaGOO_A02_91711 | A02 | 18862120 | 1.38E-08 | 0.163 | 104 | 0.692 | 0.810 | 2.13E-05 | 11.8 |
| --- | --- | --- | --- | --- | --- | --- | --- | --- | --- |
| RaGOO_A02_105457 | A02 | 21826444 | 1.38E-08 | 0.154 | 104 | 0.692 | 0.810 | 2.13E-05 | 11.8 |
| RaGOO_A02_109327 | A02 | 22603514 | 1.38E-08 | 0.135 | 104 | 0.692 | 0.810 | 2.13E-05 | 11.8 |
| RaGOO_A02_76753 | A02 | 16331442 | 1.38E-08 | 0.159 | 104 | 0.692 | 0.810 | 2.13E-05 | 11.8 |
| RaGOO_A02_73274 | A02 | 15757652 | 1.38E-08 | 0.159 | 104 | 0.692 | 0.810 | 2.13E-05 | 11.8 |
| RaGOO_A02_105951 | A02 | 21911039 | 1.39E-08 | 0.154 | 104 | 0.692 | 0.810 | 2.13E-05 | 11.8 |
| RaGOO_A02_120052 | A02 | 25234058 | 1.39E-08 | 0.144 | 104 | 0.692 | 0.810 | 2.13E-05 | 11.8 |
| RaGOO_A02_123253 | A02 | 25693177 | 1.39E-08 | 0.149 | 104 | 0.692 | 0.810 | 2.13E-05 | 11.8 |
| RaGOO_A02_86233 | A02 | 17803072 | 1.39E-08 | 0.159 | 104 | 0.692 | 0.810 | 2.14E-05 | 11.8 |
| RaGOO_A02_123435 | A02 | 25710902 | 1.39E-08 | 0.173 | 104 | 0.692 | 0.810 | 2.14E-05 | 11.8 |
| RaGOO_A02_117562 | A02 | 24754369 | 1.39E-08 | 0.159 | 104 | 0.692 | 0.810 | 2.14E-05 | 11.8 |
| RaGOO_A02_106126 | A02 | 21928383 | 1.39E-08 | 0.154 | 104 | 0.692 | 0.810 | 2.14E-05 | 11.8 |
| RaGOO_A02_102276 | A02 | 21103602 | 1.40E-08 | 0.168 | 104 | 0.692 | 0.810 | 2.14E-05 | 11.8 |
| RaGOO_A02_114495 | A02 | 24332293 | 1.40E-08 | 0.183 | 104 | 0.692 | 0.810 | 2.15E-05 | 11.8 |
| RaGOO_A02_76449 | A02 | 16295936 | 1.40E-08 | 0.154 | 104 | 0.692 | 0.810 | 2.15E-05 | 11.8 |
| RaGOO_A02_113909 | A02 | 24235159 | 1.40E-08 | 0.163 | 104 | 0.692 | 0.810 | 2.15E-05 | 11.8 |
| RaGOO_A02_118539 | A02 | 24934731 | 1.41E-08 | 0.159 | 104 | 0.692 | 0.810 | 2.16E-05 | 11.8 |
| RaGOO_A02_104703 | A02 | 21640319 | 1.41E-08 | 0.168 | 104 | 0.692 | 0.810 | 2.16E-05 | 11.8 |
| RaGOO_A02_73495 | A02 | 15792459 | 1.41E-08 | 0.173 | 104 | 0.692 | 0.810 | 2.16E-05 | 11.8 |
| RaGOO_A02_116656 | A02 | 24601090 | 1.41E-08 | 0.154 | 104 | 0.692 | 0.810 | 2.17E-05 | 11.8 |
| RaGOO_A02_110817 | A02 | 22883867 | 1.42E-08 | 0.154 | 104 | 0.692 | 0.810 | 2.17E-05 | 11.8 |
| RaGOO_A02_118960 | A02 | 24998112 | 1.42E-08 | 0.130 | 104 | 0.692 | 0.810 | 2.17E-05 | 11.8 |
| RaGOO_A02_118675 | A02 | 24966839 | 1.42E-08 | 0.168 | 104 | 0.692 | 0.810 | 2.17E-05 | 11.8 |
| RaGOO_A02_110584 | A02 | 22838082 | 1.42E-08 | 0.159 | 104 | 0.692 | 0.810 | 2.17E-05 | 11.8 |
| RaGOO_A02_114500 | A02 | 24332491 | 1.43E-08 | 0.173 | 104 | 0.692 | 0.810 | 2.18E-05 | 11.8 |
| RaGOO_A02_78909 | A02 | 16764795 | 1.43E-08 | 0.139 | 104 | 0.692 | 0.810 | 2.18E-05 | 11.8 |
| RaGOO_A02_122726 | A02 | 25607915 | 1.44E-08 | 0.154 | 104 | 0.692 | 0.810 | 2.19E-05 | 11.8 |
| RaGOO_A02_76972 | A02 | 16415257 | 1.44E-08 | 0.168 | 104 | 0.692 | 0.810 | 2.19E-05 | 11.8 |
| RaGOO_A02_107150 | A02 | 22116237 | 1.44E-08 | 0.149 | 104 | 0.692 | 0.810 | 2.19E-05 | 11.8 |
| RaGOO_A02_99678 | A02 | 20575614 | 1.44E-08 | 0.163 | 104 | 0.692 | 0.810 | 2.20E-05 | 11.8 |
| RaGOO_A02_97673 | A02 | 20189051 | 1.45E-08 | 0.149 | 104 | 0.692 | 0.810 | 2.20E-05 | 11.8 |
| RaGOO_A02_78021 | A02 | 16615648 | 1.45E-08 | 0.154 | 104 | 0.692 | 0.810 | 2.20E-05 | 11.8 |
| RaGOO_A02_105248 | A02 | 21798851 | 1.45E-08 | 0.154 | 104 | 0.692 | 0.810 | 2.20E-05 | 11.8 |
| RaGOO_A02_83625 | A02 | 17374213 | 1.45E-08 | 0.154 | 104 | 0.692 | 0.810 | 2.20E-05 | 11.8 |
| RaGOO_A02_89826 | A02 | 18562713 | 1.45E-08 | 0.159 | 104 | 0.692 | 0.810 | 2.20E-05 | 11.8 |
| RaGOO_A02_89825 | A02 | 18562702 | 1.45E-08 | 0.159 | 104 | 0.692 | 0.810 | 2.20E-05 | 11.8 |
| RaGOO_A02_105928 | A02 | 21908360 | 1.45E-08 | 0.139 | 104 | 0.692 | 0.810 | 2.20E-05 | 11.8 |
| RaGOO_A02_101957 | A02 | 21046411 | 1.46E-08 | 0.188 | 104 | 0.692 | 0.810 | 2.21E-05 | 11.7 |
| RaGOO_A02_111918 | A02 | 23853884 | 1.46E-08 | 0.154 | 104 | 0.692 | 0.809 | 2.21E-05 | 11.7 |
| RaGOO_A02_111229 | A02 | 22948137 | 1.46E-08 | 0.168 | 104 | 0.692 | 0.809 | 2.21E-05 | 11.7 |
| RaGOO_A02_100287 | A02 | 20758326 | 1.46E-08 | 0.163 | 104 | 0.692 | 0.809 | 2.21E-05 | 11.7 |
| RaGOO_A02_114620 | A02 | 24365656 | 1.46E-08 | 0.154 | 104 | 0.692 | 0.809 | 2.22E-05 | 11.7 |
| RaGOO_A02_118340 | A02 | 24888023 | 1.47E-08 | 0.159 | 104 | 0.692 | 0.809 | 2.22E-05 | 11.7 |
| RaGOO_A02_72815 | A02 | 15654676 | 1.48E-08 | 0.139 | 104 | 0.692 | 0.809 | 2.23E-05 | 11.7 |
| RaGOO_A02_114486 | A02 | 24331318 | 1.48E-08 | 0.168 | 104 | 0.692 | 0.809 | 2.24E-05 | 11.7 |
| RaGOO_A02_89268 | A02 | 18465980 | 1.49E-08 | 0.159 | 104 | 0.692 | 0.809 | 2.25E-05 | 11.7 |
| RaGOO_A02_97679 | A02 | 20189500 | 1.49E-08 | 0.178 | 104 | 0.692 | 0.809 | 2.26E-05 | 11.7 |
| RaGOO_A02_104281 | A02 | 21558718 | 1.50E-08 | 0.159 | 104 | 0.692 | 0.809 | 2.26E-05 | 11.7 |
| RaGOO_A02_98846 | A02 | 20451992 | 1.50E-08 | 0.154 | 104 | 0.692 | 0.809 | 2.27E-05 | 11.7 |
| RaGOO_A02_107071 | A02 | 22103244 | 1.51E-08 | 0.168 | 104 | 0.692 | 0.809 | 2.27E-05 | 11.7 |
| RaGOO_A02_86553 | A02 | 17850348 | 1.51E-08 | 0.130 | 104 | 0.692 | 0.809 | 2.27E-05 | 11.7 |
| RaGOO_A02_100355 | A02 | 20783658 | 1.51E-08 | 0.159 | 104 | 0.692 | 0.809 | 2.27E-05 | 11.7 |
| RaGOO_A02_117668 | A02 | 24796291 | 1.51E-08 | 0.173 | 104 | 0.692 | 0.809 | 2.27E-05 | 11.7 |
| RaGOO_A02_80208 | A02 | 16923767 | 1.51E-08 | 0.163 | 104 | 0.692 | 0.809 | 2.27E-05 | 11.7 |
| RaGOO_A02_72488 | A02 | 15585324 | 1.51E-08 | 0.149 | 104 | 0.692 | 0.809 | 2.27E-05 | 11.7 |
| RaGOO_A02_115803 | A02 | 24518403 | 1.51E-08 | 0.159 | 104 | 0.692 | 0.809 | 2.27E-05 | 11.7 |
| RaGOO_A02_117241 | A02 | 24722918 | 1.52E-08 | 0.154 | 104 | 0.692 | 0.809 | 2.28E-05 | 11.7 |
| RaGOO_A02_115790 | A02 | 24517443 | 1.53E-08 | 0.154 | 104 | 0.692 | 0.809 | 2.29E-05 | 11.7 |
| RaGOO_A02_91602 | A02 | 18855904 | 1.53E-08 | 0.135 | 104 | 0.692 | 0.809 | 2.29E-05 | 11.7 |
| RaGOO_A02_98955 | A02 | 20469587 | 1.53E-08 | 0.163 | 104 | 0.692 | 0.809 | 2.29E-05 | 11.7 |
| RaGOO_A02_99816 | A02 | 20610945 | 1.53E-08 | 0.154 | 104 | 0.692 | 0.809 | 2.29E-05 | 11.7 |
| RaGOO_A02_102579 | A02 | 21141855 | 1.53E-08 | 0.159 | 104 | 0.692 | 0.809 | 2.29E-05 | 11.7 |
| RaGOO_A02_86241 | A02 | 17803286 | 1.53E-08 | 0.154 | 104 | 0.692 | 0.809 | 2.29E-05 | 11.7 |
| RaGOO_A02_110332 | A02 | 22794276 | 1.54E-08 | 0.149 | 104 | 0.692 | 0.809 | 2.30E-05 | 11.7 |
| RaGOO_A02_85609 | A02 | 17710843 | 1.54E-08 | 0.149 | 104 | 0.692 | 0.809 | 2.30E-05 | 11.7 |
| RaGOO_A02_99165 | A02 | 20486751 | 1.54E-08 | 0.159 | 104 | 0.692 | 0.809 | 2.30E-05 | 11.7 |
| RaGOO_A02_77201 | A02 | 16449636 | 1.54E-08 | 0.163 | 104 | 0.692 | 0.809 | 2.30E-05 | 11.7 |
| RaGOO_A02_116377 | A02 | 24579597 | 1.54E-08 | 0.144 | 104 | 0.692 | 0.809 | 2.30E-05 | 11.7 |
| RaGOO_A02_118549 | A02 | 24935343 | 1.54E-08 | 0.149 | 104 | 0.692 | 0.809 | 2.30E-05 | 11.7 |
| RaGOO_A02_97206 | A02 | 20130320 | 1.55E-08 | 0.154 | 104 | 0.692 | 0.809 | 2.31E-05 | 11.7 |
| RaGOO_A02_79668 | A02 | 16863643 | 1.55E-08 | 0.163 | 104 | 0.692 | 0.809 | 2.32E-05 | 11.7 |
| RaGOO_A02_102578 | A02 | 21141828 | 1.56E-08 | 0.163 | 104 | 0.692 | 0.809 | 2.32E-05 | 11.7 |
| RaGOO_A02_90565 | A02 | 18672409 | 1.56E-08 | 0.173 | 104 | 0.692 | 0.809 | 2.32E-05 | 11.7 |
| RaGOO_A02_106290 | A02 | 21954212 | 1.56E-08 | 0.149 | 104 | 0.692 | 0.809 | 2.32E-05 | 11.7 |
| RaGOO_A02_111647 | A02 | 23172180 | 1.57E-08 | 0.154 | 104 | 0.692 | 0.809 | 2.34E-05 | 11.7 |
| RaGOO_A02_82647 | A02 | 17239306 | 1.58E-08 | 0.163 | 104 | 0.692 | 0.809 | 2.34E-05 | 11.7 |
| RaGOO_A02_113853 | A02 | 24231505 | 1.58E-08 | 0.168 | 104 | 0.692 | 0.809 | 2.34E-05 | 11.7 |
| RaGOO_A02_111694 | A02 | 23178554 | 1.58E-08 | 0.178 | 104 | 0.692 | 0.809 | 2.35E-05 | 11.7 |
| RaGOO_A04_79376 | A04 | 16013298 | 5.83E-10 | 0.115 | 104 | 0.692 | 0.837 | 2.62E-06 | 14.5 |
| RaGOO_A04_79377 | A04 | 16013385 | 1.94E-09 | 0.125 | 104 | 0.692 | 0.827 | 5.66E-06 | 13.5 |
| RaGOO_A05_102706 | A05 | 18538316 | 1.06E-08 | 0.163 | 104 | 0.692 | 0.812 | 1.77E-05 | 12.0 |
| RaGOO_A05_102728 | A05 | 18539050 | 2.01E-10 | 0.178 | 104 | 0.692 | 0.847 | 1.52E-06 | 15.5 |
| RaGOO_A05_102730 | A05 | 18539188 | 5.37E-09 | 0.168 | 104 | 0.692 | 0.818 | 1.12E-05 | 12.6 |
| RaGOO_A05_102731 | A05 | 18539205 | 3.08E-09 | 0.178 | 104 | 0.692 | 0.823 | 7.84E-06 | 13.1 |
| RaGOO_A05_102754 | A05 | 18540072 | 1.69E-09 | 0.168 | 104 | 0.692 | 0.828 | 5.18E-06 | 13.6 |
| RaGOO_A05_102755 | A05 | 18540140 | 8.15E-09 | 0.163 | 104 | 0.692 | 0.814 | 1.50E-05 | 12.2 |
| RaGOO_A05_102762 | A05 | 18540272 | 1.29E-10 | 0.163 | 104 | 0.692 | 0.851 | 1.17E-06 | 15.9 |
| RaGOO_A07_4421 | A07 | 5699569 | 3.72E-10 | 0.163 | 104 | 0.692 | 0.841 | 2.03E-06 | 14.9 |
| RaGOO_A08_2486 | A08 | 931429 | 1.42E-08 | 0.096 | 104 | 0.692 | 0.810 | 2.17E-05 | 11.8 |
| RaGOO_A08_31061 | A08 | 12569789 | 1.42E-10 | 0.130 | 104 | 0.692 | 0.850 | 1.26E-06 | 15.8 |
| RaGOO_A08_31074 | A08 | 12570715 | 9.80E-09 | 0.130 | 104 | 0.692 | 0.813 | 1.68E-05 | 12.1 |

| RaGOO_A08_31091 | A08 | 12571618 | 3.16E-09 | 0.139 | 104 | 0.692 | 0.822 | 7.97E-06 | 13.0 |
| --- | --- | --- | --- | --- | --- | --- | --- | --- | --- |
| RaGOO_A08_46366 | A08 | 17416475 | 7.88E-09 | 0.120 | 104 | 0.692 | 0.815 | 1.47E-05 | 12.3 |
| RaGOO_A08_46368 | A08 | 17416498 | 1.33E-09 | 0.111 | 104 | 0.692 | 0.830 | 4.45E-06 | 13.8 |
| RaGOO_A10_33985 | A10 | 8925359 | 1.38E-08 | 0.154 | 104 | 0.692 | 0.810 | 2.13E-05 | 11.8 |
| RaGOO_C01_219292 | C01 | 44755117 | 1.48E-08 | 0.111 | 104 | 0.692 | 0.809 | 2.23E-05 | 11.7 |
| RaGOO_C01_219295 | C01 | 44755281 | 8.44E-10 | 0.120 | 104 | 0.692 | 0.834 | 3.33E-06 | 14.2 |
| RaGOO_C02_85550 | C02 | 29210197 | 5.70E-09 | 0.139 | 104 | 0.692 | 0.817 | 1.17E-05 | 12.5 |
| RaGOO_C02_85551 | C02 | 29210198 | 5.70E-09 | 0.139 | 104 | 0.692 | 0.817 | 1.17E-05 | 12.5 |
| RaGOO_C02_114462 | C02 | 34924439 | 7.87E-10 | 0.120 | 104 | 0.692 | 0.835 | 3.19E-06 | 14.3 |
| RaGOO_C02_114463 | C02 | 34924462 | 1.16E-09 | 0.125 | 104 | 0.692 | 0.831 | 4.10E-06 | 13.9 |
| RaGOO_C02_114464 | C02 | 34924511 | 1.81E-10 | 0.120 | 104 | 0.692 | 0.848 | 1.44E-06 | 15.6 |
| RaGOO_C02_135349 | C02 | 39050494 | 8.33E-11 | 0.130 | 104 | 0.692 | 0.855 | 9.24E-07 | 16.3 |
| RaGOO_C02_135350 | C02 | 39050506 | 1.04E-08 | 0.139 | 104 | 0.692 | 0.812 | 1.75E-05 | 12.0 |
| RaGOO_C02_135351 | C02 | 39050560 | 6.77E-13 | 0.130 | 104 | 0.692 | 0.902 | 1.58E-07 | 21.0 |
| RaGOO_C02_135352 | C02 | 39050621 | 6.77E-13 | 0.130 | 104 | 0.692 | 0.902 | 1.58E-07 | 21.0 |
| RaGOO_C02_135353 | C02 | 39050687 | 9.74E-09 | 0.130 | 104 | 0.692 | 0.813 | 1.68E-05 | 12.1 |
| RaGOO_C02_150771 | C02 | 69855238 | 5.28E-10 | 0.120 | 104 | 0.692 | 0.838 | 2.51E-06 | 14.6 |
| RaGOO_C02_150772 | C02 | 69855239 | 7.10E-09 | 0.115 | 104 | 0.692 | 0.816 | 1.37E-05 | 12.4 |
| RaGOO_C02_163273 | C02 | 85587972 | 2.33E-09 | 0.120 | 104 | 0.692 | 0.825 | 6.39E-06 | 13.3 |
| RaGOO_C02_163274 | C02 | 85587982 | 1.63E-09 | 0.125 | 104 | 0.692 | 0.828 | 5.07E-06 | 13.6 |
| RaGOO_C02_172139 | C02 | 87428754 | 2.98E-09 | 0.130 | 104 | 0.692 | 0.823 | 7.66E-06 | 13.1 |
| RaGOO_C02_172143 | C02 | 87428929 | 5.38E-14 | 0.144 | 104 | 0.692 | 0.929 | 9.34E-08 | 23.7 |
| RaGOO_C02_172144 | C02 | 87428958 | 8.70E-14 | 0.135 | 104 | 0.692 | 0.923 | 9.34E-08 | 23.1 |
| RaGOO_C02_177806 | C02 | 88262680 | 4.46E-10 | 0.120 | 104 | 0.692 | 0.840 | 2.28E-06 | 14.8 |
| RaGOO_C02_178224 | C02 | 88320963 | 5.04E-09 | 0.111 | 104 | 0.692 | 0.818 | 1.07E-05 | 12.6 |
| RaGOO_C02_178266 | C02 | 88397106 | 1.43E-08 | 0.111 | 104 | 0.692 | 0.810 | 2.18E-05 | 11.8 |
| RaGOO_C02_186105 | C02 | 89813054 | 3.92E-10 | 0.125 | 104 | 0.692 | 0.841 | 2.09E-06 | 14.9 |
| RaGOO_C02_186108 | C02 | 89813248 | 1.27E-09 | 0.130 | 104 | 0.692 | 0.830 | 4.37E-06 | 13.8 |
| RaGOO_C02_186109 | C02 | 89813263 | 8.67E-09 | 0.125 | 104 | 0.692 | 0.814 | 1.57E-05 | 12.2 |
| RaGOO_C02_186154 | C02 | 89816312 | 4.88E-09 | 0.096 | 104 | 0.692 | 0.819 | 1.05E-05 | 12.7 |
| RaGOO_C02_186155 | C02 | 89816323 | 6.22E-09 | 0.096 | 104 | 0.692 | 0.817 | 1.24E-05 | 12.5 |
| RaGOO_C02_186364 | C02 | 90074416 | 2.50E-09 | 0.115 | 104 | 0.692 | 0.824 | 6.72E-06 | 13.2 |
| RaGOO_C06_8439 | C06 | 1644925 | 1.09E-10 | 0.216 | 104 | 0.692 | 0.852 | 1.06E-06 | 16.0 |
| RaGOO_C06_8440 | C06 | 1644948 | 2.67E-09 | 0.216 | 104 | 0.692 | 0.824 | 7.06E-06 | 13.2 |
| RaGOO_C06_8441 | C06 | 1644967 | 1.50E-09 | 0.212 | 104 | 0.692 | 0.829 | 4.79E-06 | 13.7 |
| RaGOO_C06_8442 | C06 | 1645009 | 6.68E-10 | 0.207 | 104 | 0.692 | 0.836 | 2.85E-06 | 14.4 |
| RaGOO_C06_8449 | C06 | 1645213 | 1.56E-08 | 0.212 | 104 | 0.692 | 0.809 | 2.32E-05 | 11.7 |
| RaGOO_C06_8461 | C06 | 1645570 | 1.38E-08 | 0.178 | 104 | 0.692 | 0.810 | 2.13E-05 | 11.8 |
| RaGOO_C06_8544 | C06 | 1647526 | 1.61E-09 | 0.202 | 104 | 0.692 | 0.828 | 5.02E-06 | 13.6 |
| RaGOO_C07_175144 | C07 | 57003035 | 1.01E-08 | 0.183 | 104 | 0.692 | 0.813 | 1.71E-05 | 12.1 |
| RaGOO_C08_67021 | C08 | 21380071 | 5.44E-09 | 0.154 | 104 | 0.692 | 0.818 | 1.13E-05 | 12.6 |
| RaGOO_C08_161973 | C08 | 52402284 | 7.91E-10 | 0.149 | 104 | 0.692 | 0.835 | 3.19E-06 | 14.3 |
| RaGOO_C08_162000 | C08 | 52403244 | 1.24E-08 | 0.139 | 104 | 0.692 | 0.811 | 1.97E-05 | 11.9 |
| RaGOO_C08_162001 | C08 | 52403251 | 1.40E-09 | 0.144 | 104 | 0.692 | 0.829 | 4.63E-06 | 13.7 |
| RaGOO_C08_162002 | C08 | 52403306 | 7.35E-10 | 0.135 | 104 | 0.692 | 0.835 | 3.05E-06 | 14.3 |

FDR=False Discovery rate, PVE=Phenotypic variation explained.

**Table S5.** Genome wide association study (GWAS) in fixed and random model circulating probability unification showing the seven significant SNPs above the GWAS threshold 1.55E-08 in *Brassica napus* cv. Darmor bzh v9.

| **SNP** | **Chromosome** | **Position** | **P-value** | **Minor allele frequency** | **False discovery rate_ P-values** | **Allele effect** |
| --- | --- | --- | --- | --- | --- | --- |
| RaGOO_A02_103419 | A02 | 21340791 | 2.20E-78 | 0.135 | 7.13E-72 | 0.875 |
| RaGOO_C05_5843 | C05 | 1437330 | 2.48E-23 | 0.063 | 4.01E-17 | 0.147 |
| RaGOO_A07_55647 | A07 | 16923737 | 9.80E-20 | 0.053 | 1.06E-13 | -0.121 |
| RaGOO_A03_51052 | A03 | 9882026 | 1.01E-13 | 0.077 | 8.13E-08 | 0.112 |
| RaGOO_A07_77461 | A07 | 20447140 | 3.19E-12 | 0.072 | 2.06E-06 | 0.075 |
| RaGOO_A01_44053 | A01 | 7255093 | 1.81E-11 | 0.053 | 9.74E-06 | -0.079 |
| RaGOO_A09_35515 | A09 | 15311164 | 8.45E-11 | 0.058 | 3.90E-05 | -0.064 |

**Table S6**. Delineation of the other quantitative trait loci (QTL) in this study including number of genes and the resistance gene analogs (RGAs) content using the mixed linear model (MLM) genome-wide association study (GWAS) and its significant single nucleotide polymorphism (SNP) associated with *LepR1* blackleg resistance in *Brassica napus.*

| **QTL name** | **Chromosome (Position (Mb))** | **Associated SNP/s delieneating the QTL** | **Genetic information within the QTL intervals** | |
| --- | --- | --- | --- | --- |
|  |  |  | **Total RGAs** | **Total genes** |
| *LepR1* _mlm2 | A04 (15.91-16.11) | RaGOO_A04_793 76 & 79377  (flanking SNPs) | 0 | 34 |
| *LepR1* _mlm3 | A05 (18.44-18.64) | RaGOO_A05_102 706 & 102762  (flanking SNPs) | 0 | 34 |
| *LepR1* _mlm4 | A07 (16.82-20.55) | RaGOO_A07_442  1 | 0 | 3 |
| *LepR1* _mlm5 | A08 (0.83-1.03) | RaGOO_A08_248  6 | 0 | 23 |
| *LepR1* _mlm6 | A09 (15.21-15.41) | RaGOO_A09_355  15 | 0 | 19 |
| *LepR1* _mlm7 | A10 (8.83-9.03) | RaGOO_A10_339  85 | 0 | 5 |
| *LepR1* _mlm8 | C01 (44.66-44.86) | RaGOO_C01_219 292 & 219295  (flanking SNPs) | 1 | 21 |
| *LepR1* _mlm9 | C02 (29.11-39.15) | RaGOO_C02_855 50 & 135353  (flanking SNPs) | 10 | 1057 |
| *LepR1* _mlm10 | C02 (69.76-69.96) | RaGOO_C02_150 771 & 150772  (flanking SNPs) | 0 | 21 |
| *LepR1* _mlm11 | C02 (85.49-90.17) | RaGOO_C02_163 273 & 186364  (flanking SNPs) | 21 | 527 |
| *LepR1* _mlm12 | C06 (1.54- 1.75) | RaGOO_C06_843  9 & 8544 (flanking SNPs) | 0 | 13 |
| *LepR1* _mlm13 | C07 (56.9- 57.1) | RaGOO_C07_175  144 | 0 | 11 |
| *LepR1* _mlm14 | C08 (21.28-21.48) | RaGOO_C08_670  21 | 0 | 18 |
| *LepR1* _mlm15 | C08 (52.3- 52.5) | RaGOO_C08_161 973 & 162002  (flanking SNPs) | 0 | 34 |

**Table S7**. Resistance gene analogs (RGA) within the *LepR1* quantitative trait loci interval in chromosomes C01, and C02 of *Brassica napus* .

| **Gene** | **RGA class** | **Chromosome** | **Start (bp)** | **End (bp)** |
| --- | --- | --- | --- | --- |
| BnaC01g52450D3 | RLK | C01 | 44719410 | 44722413 |
| BnaC02g38780D3 | RLK | C02 | 30159978 | 30162210 |

| BnaC02g39630D3 | RLK | C02 | 30912907 | 30915137 |
| --- | --- | --- | --- | --- |
| BnaC02g39810D3 | TM-CC | C02 | 31054827 | 31055648 |
| BnaC02g41600D3 | TM-CC | C02 | 32597673 | 32600027 |
| BnaC02g42110D3 | RLK | C02 | 33004104 | 33006555 |
| BnaC02g42170D3 | RLK | C02 | 33022619 | 33024646 |
| BnaC02g44010D3 | RLK | C02 | 34903683 | 34906268 |
| BnaC02g44440D3 | NBS | C02 | 35327059 | 35327505 |
| BnaC02g45210D3 | RLK | C02 | 36125707 | 36126768 |
| BnaC02g47480D3 | RLK | C02 | 38217721 | 38221404 |
| BnaC02g71280D3 | RLK | C02 | 86896277 | 86900082 |
| BnaC02g71430D3 | RLK | C02 | 87036102 | 87039164 |
| BnaC02g71690D3 | TX | C02 | 87212184 | 87213112 |
| BnaC02g71700D3 | TX | C02 | 87213915 | 87214582 |
| BnaC02g71970D3 | RLK | C02 | 87437738 | 87440752 |
| BnaC02g72160D3 | TX | C02 | 87675446 | 87676572 |
| BnaC02g72180D3 | TN | C02 | 87678250 | 87682496 |
| BnaC02g72210D3 | TNL | C02 | 87709143 | 87714608 |
| BnaC02g72220D3 | TX | C02 | 87725414 | 87727019 |
| BnaC02g72250D3 | TNL | C02 | 87766645 | 87771001 |
| BnaC02g72310D3 | TX | C02 | 87834711 | 87835043 |
| BnaC02g72330D3 | NBS | C02 | 87835989 | 87837698 |
| BnaC02g72350D3 | NBS | C02 | 87846772 | 87847110 |
| BnaC02g72360D3 | TX | C02 | 87847470 | 87848010 |
| BnaC02g72370D3 | TX | C02 | 87848601 | 87851415 |
| BnaC02g72380D3 | NL | C02 | 87857316 | 87859510 |
| BnaC02g72400D3 | TNL | C02 | 87869274 | 87887431 |
| BnaC02g72780D3 | NBS | C02 | 88260671 | 88261795 |
| BnaC02g72800D3 | CN | C02 | 88274264 | 88275388 |
| BnaC02g73100D3 | RLK | C02 | 88546122 | 88549298 |
| BnaC02g73170D3 | TM-CC | C02 | 88639348 | 88641394 |

**Table S8**. Top *LepR1* candidates in chromosome A02 quantitative trait loci (QTL) of *Brassica napus* in this study and their single nucleotide polymorphism in the coding region.

| **Gene** | **SNP** | **Position** | **Alleles**  **(major/minor)** | **Reference sequence**  **(aa)** | **Variant type in**  **SNP allele*** |
| --- | --- | --- | --- | --- | --- |
| BnaA02g25110D3 | RaGOO_A02_74351 | 16000253 | A/G | TTG (Leucine) | Missense |
|  | RaGOO_A02_74352 | 16000302 | T/C | CTA (Leucine) | Synonymous |
|  | RaGOO_A02_74353 | 16000455 | C/G | GCG (Alanine) | Synonymous |
|  | RaGOO_A02_74354 | 16000503 | T/C | GTA (Valine) | Synonymous |
|  | RaGOO_A02_74355 | 16000593 | G/A | AGC (Serine) | Missense |
|  | RaGOO_A02_74356 | 16000596 | A/T | ACT (Threonine) | Synonymous |
|  | RaGOO_A02_74357 | 16000629 | C/T | GGG (Glycine) | Missense |
|  | RaGOO_A02_74358 | 16001032 | C/T | AGC (Serine) | Missense |
|  | RaGOO_A02_74359 | 16001136 | C/A | GGG (Glycine) | Missense |
|  | RaGOO_A02_74360 | 16001266 | G/C | GCG (Alanine) | Missense |
|  | RaGOO_A02_74361 | 16001286 | C/T | TTG (Leucine) | Missense |
|  | RaGOO_A02_74362 | 16002125 | C/T | GCT (Alanine) | Missense |
| BnaA02g25290D3 | RaGOO_A02_75076 | 16131851 | T/G | GAG (Glutamate) | Missense |
|  | RaGOO_A02_75077 | 16131870 | T/A | AGA (Arginine) | Nonsense |
|  | RaGOO_A02_75078 | 16131906 | T/G | AGA (Arginine) | Nonsense |
|  | RaGOO_A02_75080 | 16132161 | G/A | CCA (Proline) | Missense |
|  | RaGOO_A02_75081 | 16132186 | C/A | CTG (Leucine) | Synonymous |
|  | RaGOO_A02_75083 | 16132548 | A/T | TGT (Cysteine) | Missense |
|  | RaGOO_A02_75084 | 16132566 | A/G | TGA (STOP) | Missense |
|  | RaGOO_A02_75085 | 16132638 | C/A | GTG (Valine) | Missense |
|  | RaGOO_A02_75086 | 16132682 | G/A | TCG (Serine) | Missense |
|  | RaGOO_A02_75091 | 16132825 | G/T | TGC (Cysteine) | Missense |
|  | RaGOO_A02_75092 | 16132828 | G/A | GGC (Glycine) | Missense |
|  | RaGOO_A02_75093 | 16132831 | G/T | GTC (Valine) | Synonymous |
|  | RaGOO_A02_75094 | 16132907 | G/T | CCC (Proline) | Missense |
|  | RaGOO_A02_75096 | 16133104 | A/T | TCT (Serine) | Synonymous |
|  | RaGOO_A02_75097 | 16133231 | T/G | AAG (Lysine) | Missense |
|  | RaGOO_A02_75098 | 16133265 | T/C | ACG (Threonine) | Missense |
|  | RaGOO_A02_75104 | 16133401 | C/G | GGG (Glycine) | Missense |
|  | RaGOO_A02_75105 | 16133419 | G/A | AAC (Asparagine) | Missense |
|  | RaGOO_A02_75106 | 16133467 | T/C | ACA (Threonine) | Synonymous |
|  | RaGOO_A02_75107 | 16133492 | G/A | GCC (Alanine) | Missense |
|  | RaGOO_A02_75108 | 16133503 | A/G | ATT (Isoleucine) | Synonymous |
|  | RaGOO_A02_75109 | 16133530 | G/C | CGC (Arginine) | Synonymous |
|  | RaGOO_A02_75110 | 16133539 | C/T | GTG (Valine) | Synonymous |
|  | RaGOO_A02_75111 | 16133545 | G/A | GTC (Valine) | Synonymous |
|  | RaGOO_A02_75112 | 16133557 | C/T | GGG (Glycine) | Missense |
|  | RaGOO_A02_75115 | 16133758 | T/G | ACA (Threonine) | Synonymous |
|  | RaGOO_A02_75116 | 16133783 | A/G | CTT (Leucine) | Missense |
|  | RaGOO_A02_75117 | 16133799 | C/T | GCT (Alanine) | Missense |
|  | RaGOO_A02_75118 | 16133859 | T/G | ATG (Methionine) | Missense |
|  | RaGOO_A02_75119 | 16133868 | G/T | CGT (Arginine) | Missense |
|  | RaGOO_A02_75120 | 16133874 | G/C | CTC (Leucine) | Missense |
| BnaA02g26040D3 | RaGOO_A02_77140 | 16441347 | A/G | GGT (Glycine) | Missense |
|  | RaGOO_A02_77149 | 16441884 | C/T | TCG (Serine) | Synonymous |
|  | RaGOO_A02_77150 | 16441911 | G/C | CCC (Proline) | Synonymous |
|  | RaGOO_A02_77151 | 16441920 | A/G | GAT (Aspartate) | Missense |
|  | RaGOO_A02_77152 | 16442846 | G/A | CAC (Histidine) | Missense |
|  | RaGOO_A02_77153 | 16442919 | A/G | CTT (Leucine) | Synonymous |
|  | RaGOO_A02_77154 | 16443132 | A/G | ACT (Threonine) | Synonymous |
|  | RaGOO_A02_77155 | 16444567 | T/C | AAC (Asparagine) | Missense |
| BnaA02g27490D3 | RaGOO_A02_85221 | 17640112 | G/A | CAG (Glutamine) | Synonymous |
|  | RaGOO_A02_85222 | 17640157 | A/G | CAA (Glutamine) | Synonymous |
|  | RaGOO_A02_85223 | 17640166 | C/A | GTC (Valine) | Synonymous |
|  | RaGOO_A02_85224 | 17640206 | C/A | CAC (Histidine) | Synonymous |
|  | RaGOO_A02_85225 | 17640739 | T/C | GTT (Valine) | Synonymous |

| BnaA02g29400D3 | RaGOO_A02_95099 | 19348879 | A/C | ATG (Methionine) | Synonymous |
| --- | --- | --- | --- | --- | --- |
| BnaA02g30580D3 | RaGOO_A02_100624 | 20845266 | G/A | TAC (Tyrosine) | Nonsense |
|  | RaGOO_A02_100625 | 20846372 | A/G | TTG (Leucine) | Missense |
|  | RaGOO_A02_100626 | 20846482 | C/T | AGC (Serine) | Missense |
| BnaA02g30960D3 | RaGOO_A02_103493 | 21358047 | G/T | GAT (Aspartate) | Synonymous |
| BnaA02g31910D3 | RaGOO_A02_107160 | 22121135 | C/T | GCG (Alanine) | Synonymous |
|  | RaGOO_A02_107161 | 22121423 | A/T | TCT (Serine) | Synonymous |
|  | RaGOO_A02_107162 | 22121448 | C/T | AAG (Lysine) | Missense |
|  | RaGOO_A02_107163 | 22121492 | G/A | TTC (Phenylalaline) | Missense |
|  | RaGOO_A02_107164 | 22121654 | T/A | GCA (Alanine) | Synonymous |
|  | RaGOO_A02_107165 | 22121964 | T/C | GAA (Glutamate) | Missense |
|  | RaGOO_A02_107166 | 22122305 | A/G | GCT (Alanine) | Synonymous |
|  | RaGOO_A02_107167 | 22122323 | T/C | ACA (Threonine) | Synonymous |
|  | RaGOO_A02_107168 | 22122494 | G/T | AGC (Serine) | Missense |
|  | RaGOO_A02_107169 | 22122710 | C/T | GAG (Glutamate) | Missense |
|  | RaGOO_A02_107170 | 22123424 | T/G | TTA (Leucine) | Missense |
| BnaA02g33290D3 | RaGOO_A02_113000 | 24025646 | T/C | TGG (Tryptophan) | Synonymous |
|  | RaGOO_A02_113001 | 24025759 | C/A | ATC (Isoleucine) | Synonymous |
|  | RaGOO_A02_113002 | 24025840 | C/T | ATC (Isoleucine) | Synonymous |
|  | RaGOO_A02_113003 | 24025843 | A/G | AAA (Lysine) | Synonymous |
| BnaA02g33300D3 | RaGOO_A02_113034 | 24032005 | G/C | GCA (Alanine) | Missense |
| BnaA02g33310D3 | RaGOO_A02_113193 | 24052356 | A/G | AAA (Lysine) | Synonymous |
|  | RaGOO_A02_113194 | 24052907 | T/A | TAA (STOP) | Synonymous |
|  | RaGOO_A02_113195 | 24052992 | C/T | GCT (Alanine) | Synonymous |
|  | RaGOO_A02_113196 | 24052997 | A/G | AAT (Asparagine) | Synonymous |
|  | RaGOO_A02_113197 | 24053041 | T/G | GCT (Alanine) | Synonymous |
|  | RaGOO_A02_113198 | 24053131 | A/G | CGA (Arginine) | Synonymous |
|  | RaGOO_A02_113199 | 24053306 | T/C | TCT (Serine) | Synonymous |
|  | RaGOO_A02_113200 | 24053333 | C/A | CAT (Histidine) | Synonymous |
|  | RaGOO_A02_113201 | 24053374 | C/G | AAC (Asparagine) | Synonymous |
|  | RaGOO_A02_113202 | 24053382 | G/A | CGT (Arginine) | Synonymous |
|  | RaGOO_A02_113203 | 24053424 | A/C | AAT (Asparagine) | Synonymous |
| BnaA02g33320D3 | RaGOO_A02_113312 | 24074553 | C/T | CGC (Arginine) | Synonymous |
|  | RaGOO_A02_113313 | 24074719 | A/G | CGT (Arginine) | Missense |
|  | RaGOO_A02_113314 | 24074739 | A/C | TCA (Serine) | Synonymous |
|  | RaGOO_A02_113315 | 24074790 | A/G | TTA (Leucine) | Synonymous |
|  | RaGOO_A02_113316 | 24074889 | G/A | TCG (Serine) | Synonymous |
|  | RaGOO_A02_113317 | 24075259 | A/C | ATT (Isoleucine) | Synonymous |
|  | RaGOO_A02_113318 | 24075284 | C/A | TCT (Serine) | Synonymous |
|  | RaGOO_A02_113319 | 24075337 | C/T | CCA (Proline) | Synonymous |
|  | RaGOO_A02_113320 | 24075344 | C/T | TCA (Serine) | Synonymous |
|  | RaGOO_A02_113321 | 24075345 | A/C | TCA (Serine) | Synonymous |
| BnaA02g33740D3 | RaGOO_A02_114355 | 24311281 | G/A | ACG (Threonine) | Synonymous |
| BnaA02g33850D3 | RaGOO_A02_115355 | 24452622 | C/G | TAG (STOP) | Missense |
|  | RaGOO_A02_115357 | 24454985 | C/T | GTT (Valine) | Missense |
| BnaA02g34160D3 | RaGOO_A02_117373 | 24730383 | G/A | TAC (Tyrosine) | Nonsense |
|  | RaGOO_A02_117374 | 24730521 | T/C | ACA (Threonine) | Synonymous |
|  | RaGOO_A02_117378 | 24731068 | T/C | GAC (Aspartate) | Missense |
|  | RaGOO_A02_117379 | 24731159 | G/A | CAA (Glutamine) | Synonymous |
|  | RaGOO_A02_117380 | 24731256 | A/C | AAT (Asparagine) | Missense |
|  | RaGOO_A02_117381 | 24731260 | T/C | CAC (Histidine) | Missense |
|  | RaGOO_A02_117382 | 24731444 | C/T | GTT (Valine) | Missense |
|  | RaGOO_A02_117385 | 24731688 | C/G | TCC (Serine) | Synonymous |
|  | RaGOO_A02_117386 | 24731751 | G/C | CCC (Proline) | Synonymous |
|  | RaGOO_A02_117387 | 24731976 | A/G | GGT (Glycine) | Missense |
|  | RaGOO_A02_117388 | 24732186 | A/T | AAT (Asparagine) | Missense |
| BnaA02g34440D3 | RaGOO_A02_118508 | 24924158 | A/C | AGC (Serine) | Synonymous |
|  | RaGOO_A02_118509 | 24924181 | T/C | CTT (Leucine) | Synonymous |
|  | RaGOO_A02_118510 | 24924207 | T/C | ATG (Methionine) | Synonymous |
|  | RaGOO_A02_118511 | 24924214 | T/C | TTT (Phenylalaline) | Synonymous |
| BnaA02g35420D3 | RaGOO_A02_124045 | 25786043 | A/G | GTT (Valine) | Missense |
|  | RaGOO_A02_124060 | 25787016 | C/G | GAA (Glutamate) | Missense |

*based on the most frequent allele

**Table S9.** Results of molecular testing of markers and their subsequent sequencing analysis

| **Candidate genes** | **Band detection using markers** | **Remarks based on MiSeq sequencing results** |
| --- | --- | --- |
| BnaA02g25030D3* | successful | Inconsistent sequence within resistant alleles and so as the susceptible alleles |
| BnaA02g25110D3 | unsuccessful | - |
| BnaA02g25290D3 | unsuccessful | - |
| BnaA02g26040D3 | unsuccessful | - |
| BnaA02g27490D3 | unsuccessful | - |
| BnaA02g30580D3 | unsuccessful | - |
| BnaA02g31910D3 | unsuccessful | - |
| BnaA02g33290D3 | unsuccessful | - |
| BnaA02g33310D3 | successful | Different sequence between resistant and susceptible materials |
| BnaA02g33320D3 | unsuccessful | - |
| BnaA02g33850D3 | successful | Similar sequence of resistant and susceptible alleles |
| BnaA02g34160D3 | unsuccessful | - |
| BnaA02g34440D3 | successful | Similar sequence of resistant and susceptible alleles |
| BnaA02g35420D3 | unsuccessful | - |
| BnaA02g35480D3* | unsuccessful | - |

*flanking genes nearest to the identified *LepR1* candidates.

**Table S10.** Marker details (gtG8, resistant and non-specific markers) for the candidate BnaA02g33310D3 that can

detect *LepR1* resistant and susceptible alleles in *Brassica napus* cultivars.

| **Primer** | **Type** | **Sequences** | **Size (bp)** |
| --- | --- | --- | --- |
| gtG8* | Forward | TTACCCCACACCATTGAC | 4178 |
|  | Reverse | GGAGCGTCGGAACAGAT |  |
| Resistant-specific | Forward | AGGTGTCGTCGCTCCC | 1143 |
|  | Reverse | ATCACAACCTTTGACACAATGA |  |
| Non-specific | Forward | CTCGCACAAAGTCCAAGC | 299 |
|  | Reverse | CGCTCGGATAATTTCATGAT |  |

Marker name.

**Figures**


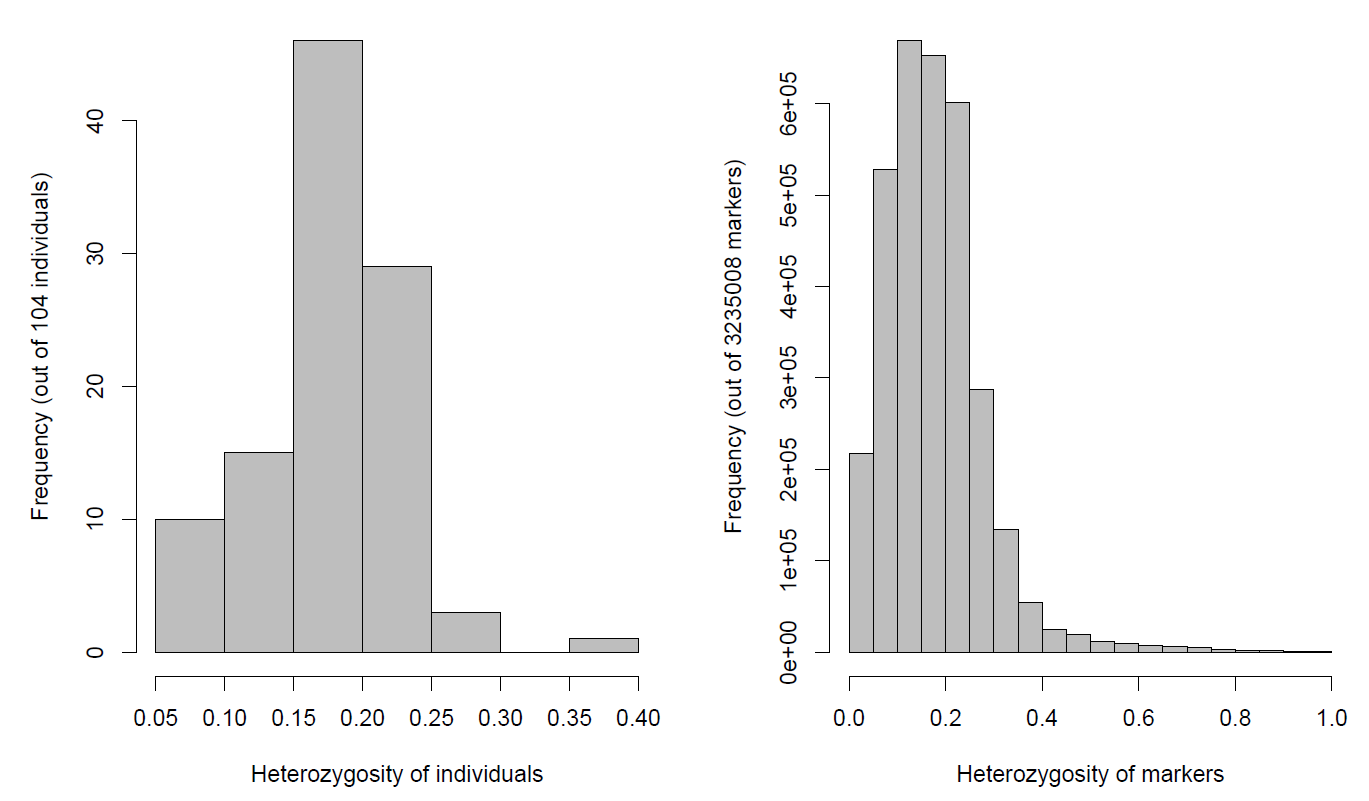


**Figure S1.** Heterozygosity information in the 104 *Brassica napus* genotypes and the 3,235,008 quality filtered single nucleotide polymorphisms used in this study.


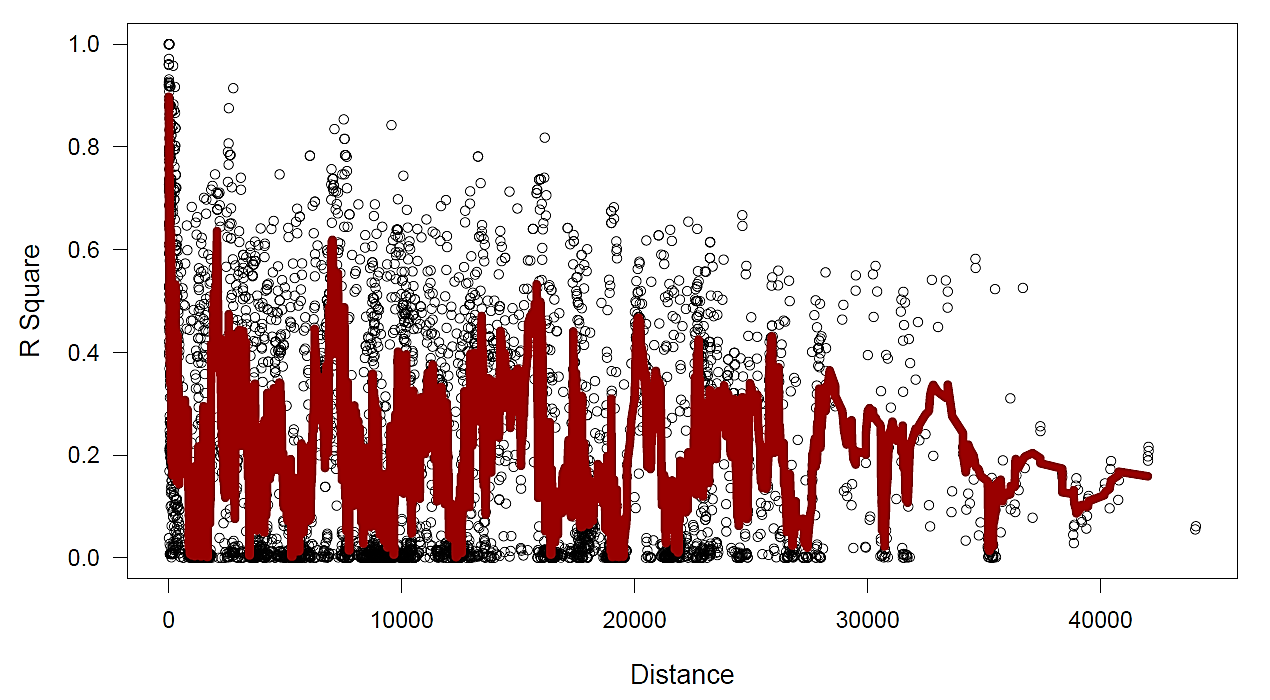


**Figure S2.** Linkage disequilibrium, represented by R^2^, decay rate in *B. napus* genome between single nucleotide polymorphism (SNP) pairs versus the physical distance. Black circles are for individual SNP pairs while red lines filling the black circles are the average R^2^ at a given physical distance.


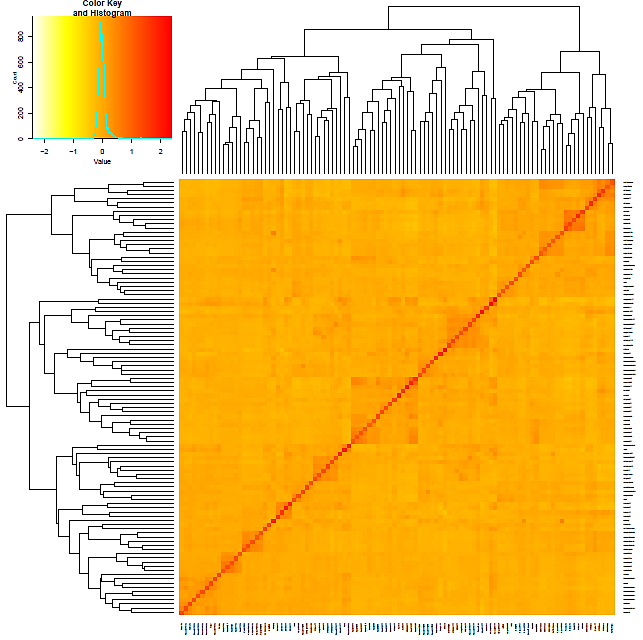


**Figure S3.** Kinship results of 104 *Brassica napus* cultivars showing the genetic distance of the 104 *B. napus* genotypes.


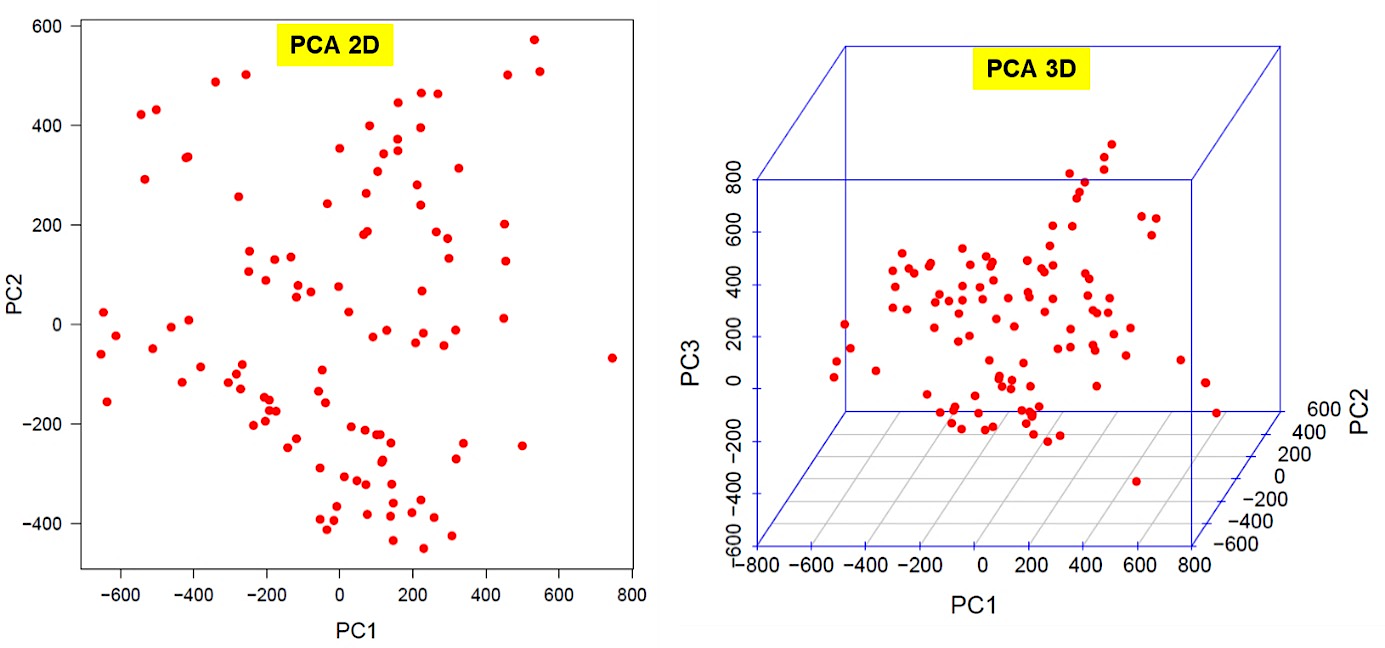


**Figure S4**. Principal component analysis (PCA) showing the structure of 104 *Brassica napus* genotypes.


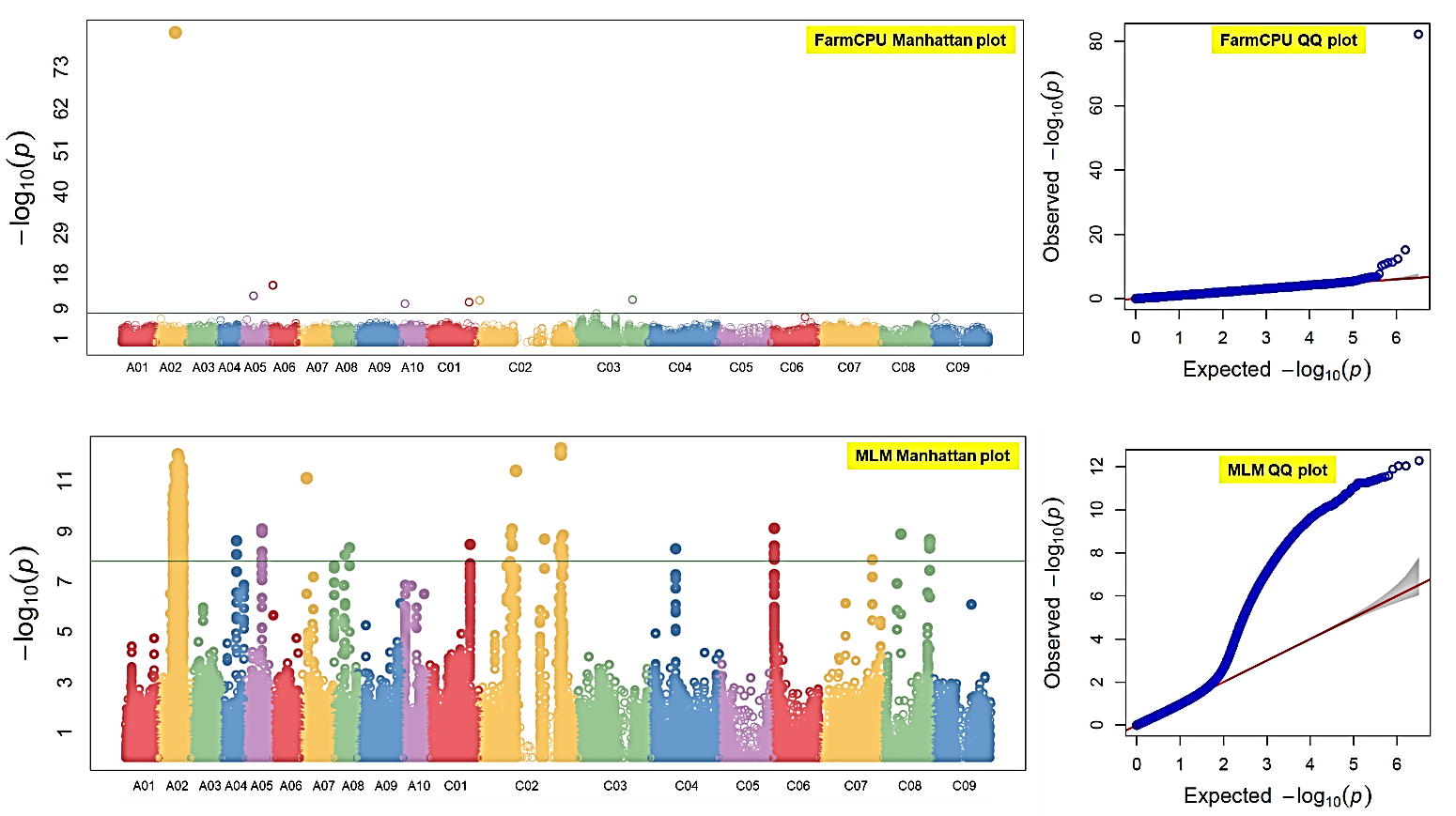


**Figure S5.** Manhattan and QQ plot showing the significant SNPs above the threshold line (green) in fixed and random model circulating probability unification (FarmCPU) of genome wide association study in *Brassica napus* cv. Darmor bzh v9.

**
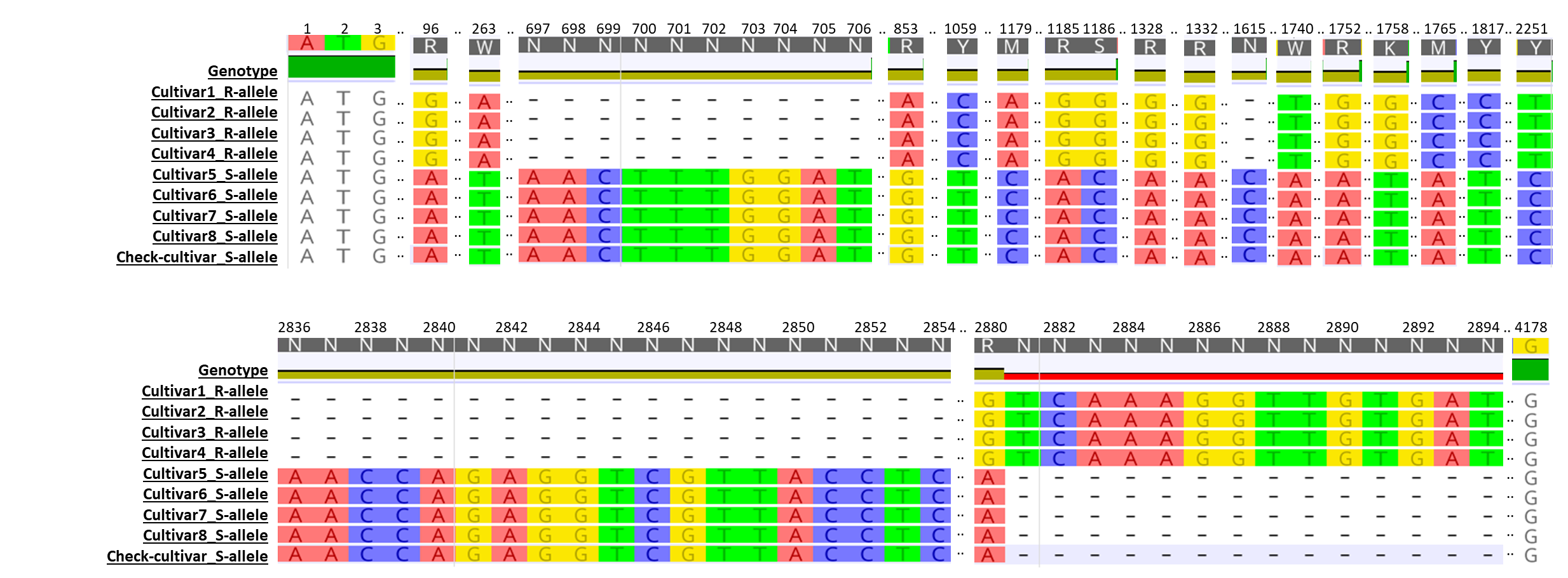
**

**Figure S6**. MiSeq sequencing results between the *LepR1* resistant (R-allele) and susceptible (S-allele) *Brassica napus* cultivars with *BnaA02g33310D3* as the reference captured by the marker gtG8 showing the selected positions having single nucleotide polymorpshim.


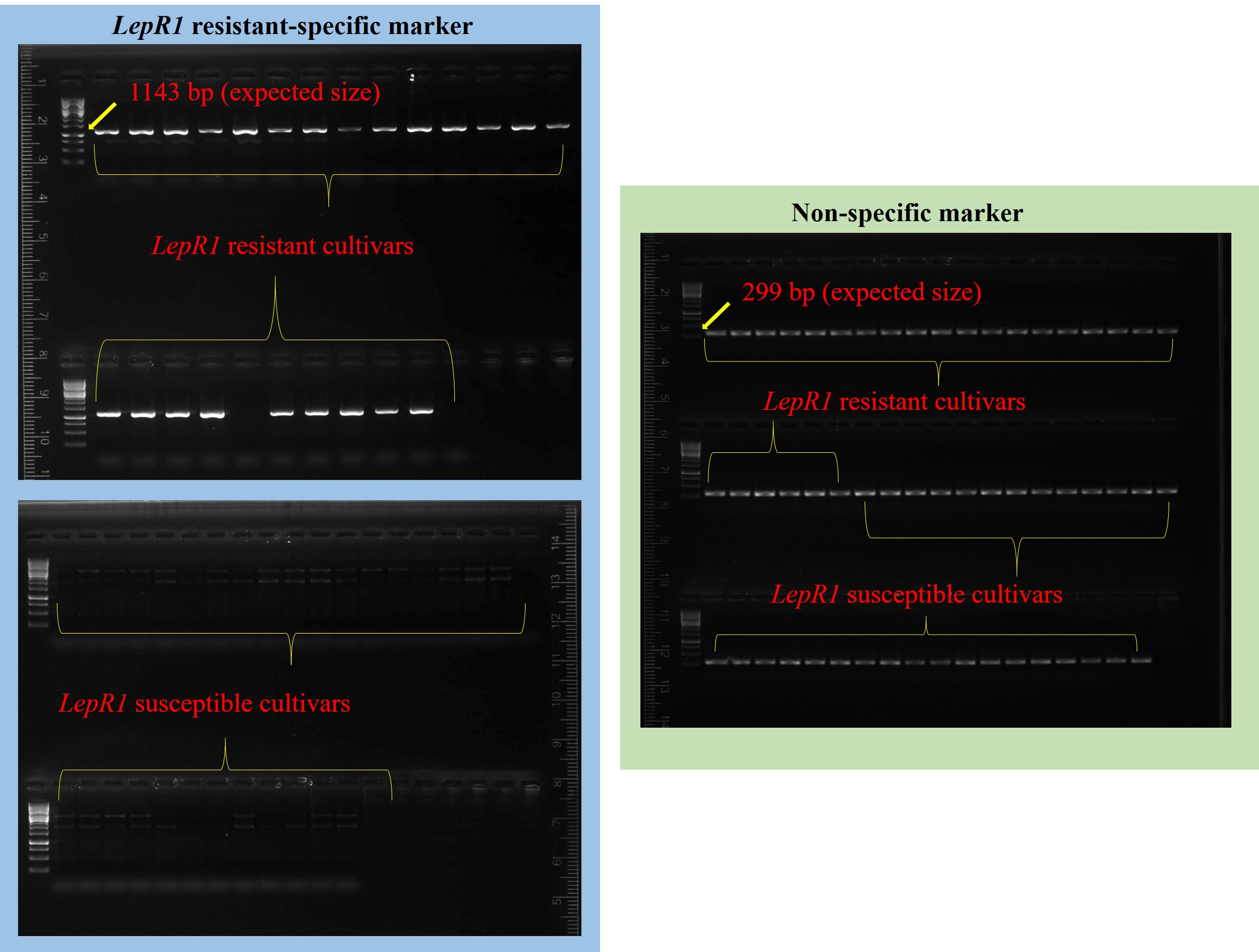


**Figure S7.** Resistant-specific and non-specific markers with their bands for the *LepR1* resistant and susceptible cultivars.
